# Ene-Allenes: Access from Dialkynes by an Intramolecular *anti*-Carbopalladation Cascade

**DOI:** 10.1021/acs.orglett.5c03990

**Published:** 2025-11-12

**Authors:** Pedram Kalvani, Catherine Vanbel, Heinrich F. von Köller, Daniel B. Werz

**Affiliations:** † 9174 Albert-Ludwigs-Universität Freiburg, Institute of Organic Chemistry, Albertstr. 21, 79104 Freiburg, Germany; ‡ 70493 Vrije Universiteit Brussel, Organic Chemistry Research Group, Pleinlaan 2, 1050 Brussels, Belgium

## Abstract

Herein, an intramolecular palladium-catalyzed cascade reaction is reported consisting of both formal *anti*-carbopalladation and *syn*-carbopalladation of internal alkynes. This process leads to two new carbon–carbon bonds across the first double bond formed. The cascade is terminated by β-hydride elimination from the emerging vinyl Pd­(II) complex. As a result, di-, tri-, and tetrasubstituted ene-allenes embedded in an oligo­(hetero)­cyclic ring system are synthesized in yields of up to 73%.

## Graphical abstract

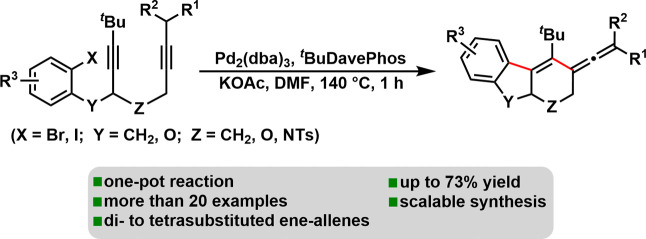

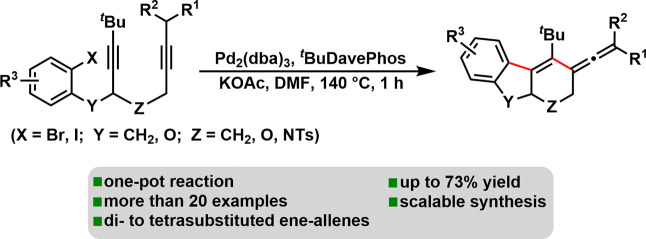

Cascade (or domino) reactions have become a very powerful approach to convert simple precursors to complex compounds in a one-pot process compared to traditional multistep methods.^1^ Among various cascade methods, palladium-catalyzed domino reactions have proven considerably successful over the last few decades. While domino reactions involving C–C double bonds are common, those involving C–C triple bonds have been gaining renewed attention during the last two decades.^2^ For a long time, the fundamental type of carbopalladations of alkynes was restricted to *syn*-carbopalladations, where additions occur on the same side of the emerging double bond.^3^ This method has enabled the synthesis of interesting structures such as molecular switches,^4^ chromans,^5^ fenestranes,^6^ biphenyls,^7^ cyclooctatetraenes,^8^ oligoene-based π-helicenes,^9^ and other heterocyclic systems.^10^

In 2015, our group developed a system for the formal *anti*-carbopalladation of internal alkynes.^11^ In the same year, the Lautens group also achieved *anti*-carbopalladation reactions of alkynes. They realized this challenge via intramolecular carbohalogenation reactions, which involved a *cis*-*trans* isomerization.^12^ To avoid undesired reactions during this transformation, particularly β-hydride elimination after the initial *syn*-attack of the organopalladium species, we installed a *tert*-butyl group at the alkyne unit. In addition, monodentate bulky phosphine ligands and elevated temperatures allowed the generation of a 14 VE (valence electron) Pd species that is able to isomerize from one double-bond geometry to another through an η^2^-vinyl palladium transition state. As a result, a *cis*-*trans*-isomerization takes place in the coordination sphere of the metal. This crucial step was also corroborated by DFT analyses.^13^

Having set the stage for the design of such *anti*-carbopalladations, over the past decade, we combined this crucial step with several different terminating reactions. Inter alia, there were Heck reactions (Scheme 1a),^14^ Stille cross-couplings,^15^ C–H activation,^16^ and intramolecular Suzuki cross-coupling reactions.^17^ We have successfully generated enamines, furans, and tetrasubstituted enol ethers by using suitable heteroatom nucleophiles that are able to attack the vinyl Pd intermediate.^18^ In our previous work, we reported alkyne systems that underwent *anti*-carbopalladation terminated intermolecularly by either Suzuki or Sonogashira cross-coupling reactions to afford complex oligocyclic compounds (Scheme 1b).^19^ During these studies, we realized that, in the case of aliphatic groups attached to the second alkyne unit, other products could be obtained as the result of a β-hydride elimination. Careful analysis revealed that the products of this cascade are ene-allenes (Scheme 1e). Although some methods have already been established for the synthesis of ene-allenes, such as the rearrangement of skipped enynes,^20^ ring-closing metathesis reactions involving 1,*n*-allenynes,^21^ and metal-catalyzed substitution of propargylic alcohol derivatives with nucleophilic reagents (Scheme 1c,d),^22^ we sought to investigate the access of such allenes by our carbopalladation method.

**Scheme 1:**
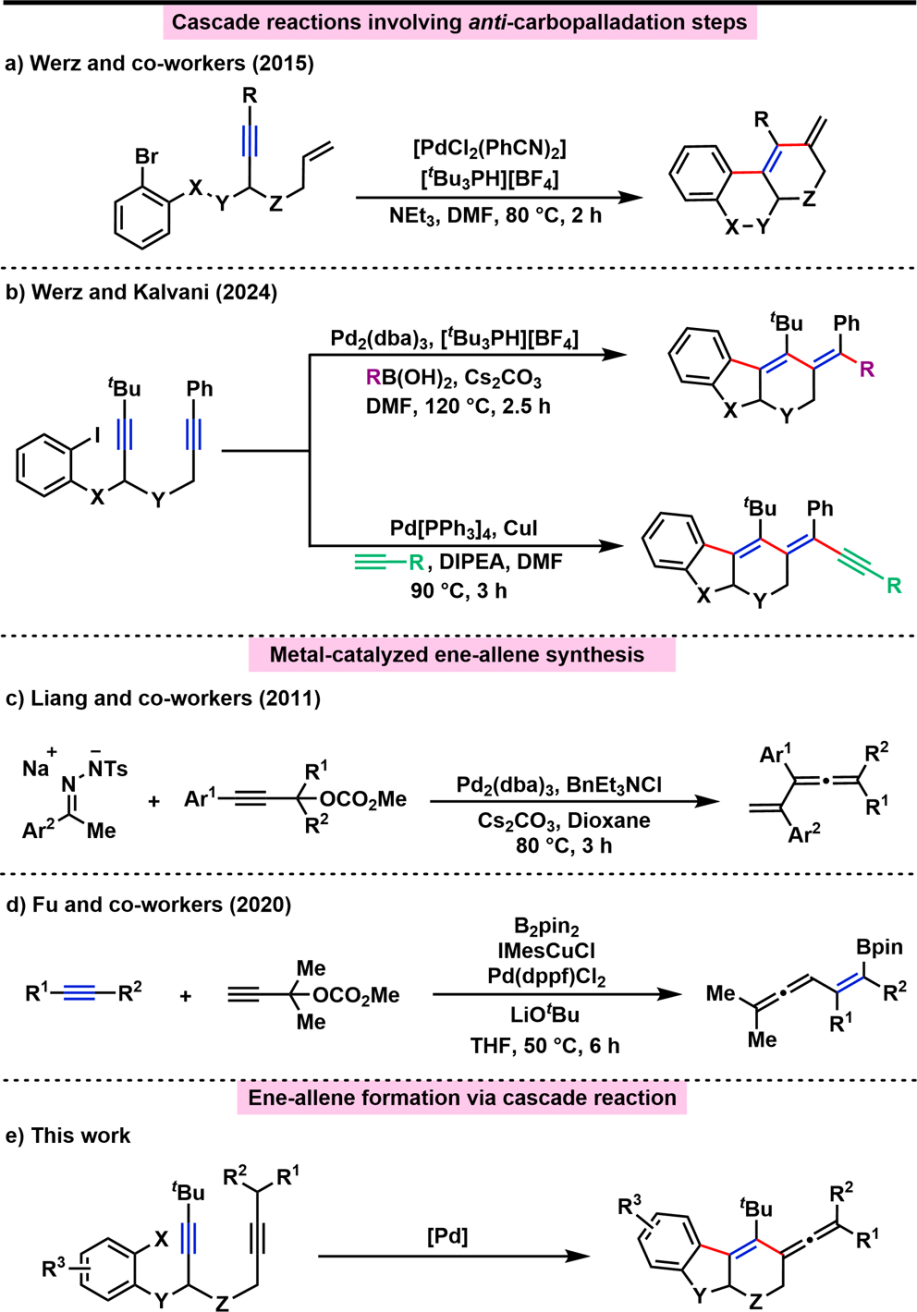
Overview of Previous Results and This Work

At the outset of our studies, compound **1a**, equipped with an aryl iodide and two alkynes capped by *tert*-butyl and methyl groups, was chosen as a model substrate to find optimized reaction conditions that pave the way for yielding ene-allenes. Our earlier investigations showed that aprotic polar solvents, such as DMF or DMA, elevated temperature, and bulky monodentate phosphine ligands are of great importance for the *anti*-carbopalladation step. Therefore, we began our investigations using a catalytic system consisting of Pd_2_(dba)_3_, [^*t*^Bu_3_PH]­[BF_4_], KOAc, and DMF at 90 °C. After 3 h, GC-MS showed the mass of the desired product, and 95% conversion of **1a** was observed with an NMR yield of 47% (see Tables S1–S3 for more information).

After thorough and systematic investigation of reaction conditions, we found the optimal conditions when Pd_2_(dba)_3_ (5 mol %), ^*t*^Bu-DavePhos (10 mol %), and KOAc (7.5 equiv) were used in DMF at 140 °C. The corresponding ene-allene is obtained in 79% NMR yield (71% isolated yield, Table 1, entry 1). Variation of the standard experiment demonstrated that the reaction would not occur in the absence of ligand or base, and working under inert gas (Ar) is crucial (entries 2–3). A broad range of organic and inorganic bases were tried; K_2_CO_3_ like other carbonate bases led to trace product formation, and CsOAc resulted in decreased yields (entries 4–5). In addition, changing the amount of base to 3.0 equiv resulted in only 58% of the allene (entry 6). As expected from earlier *anti*-carbopalladation procedures, the use of toluene as solvent resulted in no product being obtained (entry 7). The transformation needs to be conducted in highly diluted reaction mixtures; using higher concentrations, such as 25 and 100 mM, has a negative effect on the cascade process (entry 8 and Table S2). Entry 10 illustrates that stirring for more than 1 h does not affect the yield of desired ene-allenes. Furthermore, a wide range of other catalytic systems was screened; employing Pd­[PPh_3_]_4_ drastically decreased the yield to 34% (entry 11). Several Pd­(II) sources such as Pd­(OAc)_2_ along with monodentate phosphine ligands like [^*t*^Bu_3_PH]­[BF_4_] were analyzed and formed the target ene-allene in 64% and 71% yield, respectively (entries 12–13).

**Table 1:**
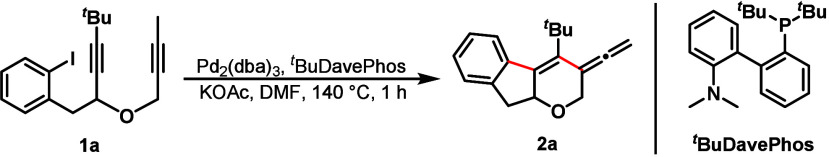
Optimization of Reaction Condition^a^

entry	variation from the standard conditions	yield^b^
1	none	79%^c^
2	no ligand, no base	n.p.^d^
3	open to air	15%
4	K_2_CO_3_ instead of KOAc	trace
5	CsOAc instead of KOAc	31%
6	3.0 equiv of base	58%
7	toluene instead of DMF	n.p.
8	25 mM DMF	62%
9	temperature dropped to 120 °C	56%
10	time increase to 3 h	79%
11^e^	Pd[PPh_3_]_4_ as catalyst	34%
12	Pd(OAc)_2_ instead of Pd_2_(dba)_3_	64%
13	TTBP·HBF_4_ instead of ^*t*^Bu-DavePhos	71%

a The reaction is conducted by using **1a** (50 μmol), precatalyst (5 mol %), ligand (10 mol %), base (7.5 equiv), and solvent (*c* = 12 mM) under inert gas.

b NMR yields were determined by ^1^H NMR analysis of the crude reaction mixture using 1,3,5-trimethoxybenzene as the internal standard.

c Isolated yield of 71%.

d n.p. = no product.

e 10 mol % of catalyst.

With the optimized reaction conditions in hand, the substrate scope of the formal *anti*-carbopalladation cascade was examined (Scheme 2). We began to investigate the effect of different halogens at the ring where the cascade starts. The corresponding domino product (**2a**) was obtained in good yield, while the iodide gave slightly better results for the transformation. Moreover, the domino process can be performed on a large scale; as an example, **2a** is collected in 67% yield on a scale of 1.0 mmol. The successful formation of tri- and tetrasubstituted ene-allenes was observed upon the change from a methyl-capped alkyne to ethyl- and isopropyl-capped alkynes (**2b**–**2c**). In addition, a tosyl-protected nitrogen was embedded in the tether between two alkynes to afford product **2d** in 62% yield. The developed approach is compatible with a range of substituents at the aryl residue, including both electron-donating and electron-withdrawing ones (**2e**–**2k**). Naphthaline- and anthracene-containing vinyl allenes (**2l** and **2m**) were also generated in 59% and 61% yield, respectively. In contrast, other previously published *anti*-carbopalladation cascade silyl groups instead of the shielding *tert*-butyl groups did not lead to product formation (see the Supporting Information).

**Scheme 2:**
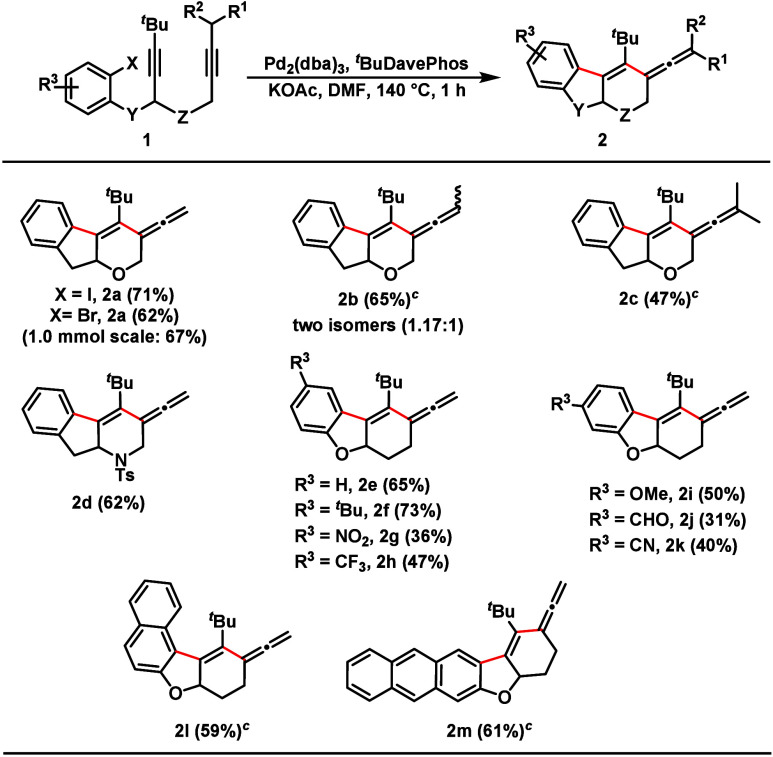
Scope of the Ene-Allene Synthesis^a^ ^,^ ^b^

a Cascade reaction conditions: **1** (1.0 equiv), Pd_2_(dba)_3_ (5 mol %), ^*t*^BuDavePhos (10 mol %), KOAc (7.5 equiv), DMF (12 mM).
b The precursor with X = I was used unless otherwise stated.
c The precursor with X = Br was used.

In order to further explore the scope of the reaction, our next step was to prepare domino substrates which would lead to larger ring sizes (Scheme 3). We showed that the transformation is not limited to the formation of the six/five/six-membered ring pattern. Precursors with longer chains between ring and alkyne moiety were prepared, leading to six/six/six-membered rings with an ene-allene moiety (**2n**). Such a scaffold was obtained in 56% and 44% yield, respectively. In addition, ene-allenes containing a seven-membered ring were obtained by our methodology (**2o**–**2r**). Using naphthaline-containing dialkyne, we were able to access a larger scaffold in good yield (**2s**). Furthermore, six/five/seven-membered ring tri- and tetrasubsituted ene-allenes were successfully synthesized in yields of about 60% (**2t** and **2u**).

**Scheme 3:**
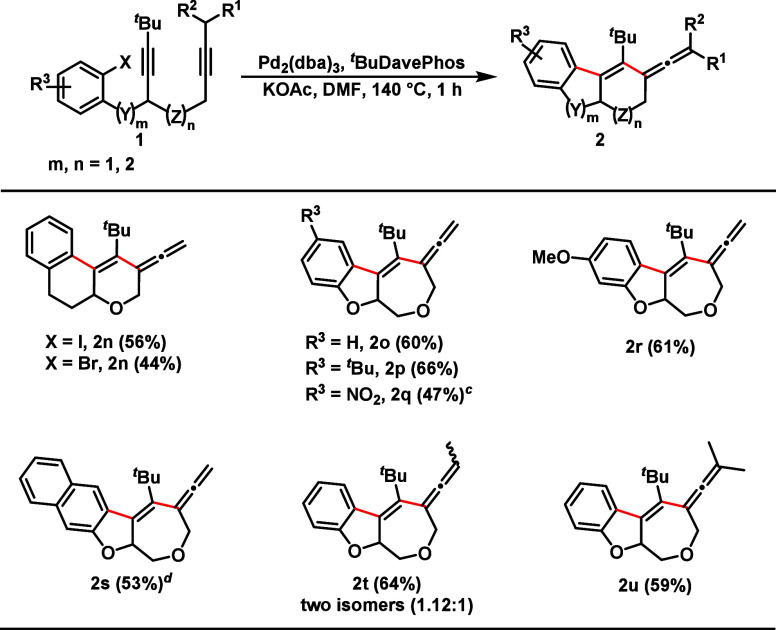
Scope of the Ene-Allene Synthesis: Larger Scaffolds^a^ ^,^ ^b^

a Cascade reaction conditions: **1** (1.0 equiv), Pd_2_(dba)_3_ (5 mol %), ^*t*^BuDavePhos (10 mol %), KOAc (7.5 equiv), DMF (12 mM).
b The precursor with X = I was used unless otherwise stated.
c The reaction was completed in 2 h.
d The precursor with X = Br was used.

To obtain deeper insights into the reaction mechanism of the palladium-catalyzed cascade reaction, we performed DFT computations. Key reaction intermediates and transition states were elucidated by theoretical means (Scheme 4; see computational details in the Supporting Information). The process begins with the oxidative addition of precursor **1a** to the Pd(0) complex (L = *^t^* Bu_3_P). This step is favorable, releasing 4.0 kcal/mol energy, and requires an energy input of 20.4 kcal/mol to overcome the activation barrier, ultimately leading to **IM1**. After the dissociation of one phosphine ligand from the metal complex, which is endothermic by 11.0 kcal/mol, the newly formed intermediate **IM2** undergoes the first *syn*-carbopalladation process to afford **IM3**. The key isomerization step via the η^2^-vinyl transition state results in a more stable 14 VE vinyl palladium species **IM4** with an energy barrier of 27.9 kcal/mol. The difference in the energy level of *syn*- and *anti*-intermediates is consistent with our previous computational studies.^11^ The second intramolecular *syn*-carbopalladation of the adjacent triple bond to afford the six-membered ring requires a low free energy barrier of 17.7 kcal/mol compared to 27.9 kcal/mol for the first same process. Subsequently, the ligand exchange in the presence of KOAc occurred in an endergonic manner. To complete the cascade reaction, ligand exchange happens, and the resulting **IM6** then undergoes β-hydride elimination through a four-membered ring transition state (**TS6**) to form allene-coordinated complex **IM7**. The calculations reveal the transformation is 4.6 kcal/mol exergonic with small barrier energy of 7.8 kcal/mol. Ultimately, PdL_2_ coordinated to the desired ene-allene is restored by ligand exchange between the acetate and *tert*-butyl phosphine; thus, the Pd(0) species is regenerated for the next cycle. The final step releases an energy of 30.7 kcal/mol, indicating that the complete cascade process is thermodynamically favorable. Our DFT calculations indicate that the *syn*-*anti* isomerization is the rate-determining step with an overall energy barrier of 27.9 kcal/mol. Also, considering the relatively weak Pd–I bond, the exchange of iodide and acetate might take place after the formation of **IM1**. If this is the case, the bidentate coordination ability of the acetate ligand could facilitate the dissociation of one phosphine ligand and thereby assist the subsequent carbopalladation step. Nevertheless, the computations for this alternative pathway reveal that this step is unfavorable since the *syn*-*anti* isomerization step requires the overall energy barrier of 36.1 kcal/mol, which is an increase by 8.2 kcal/mol compared to our first proposed pathway (See computational details in the Supporting Information).

**Scheme 4:**
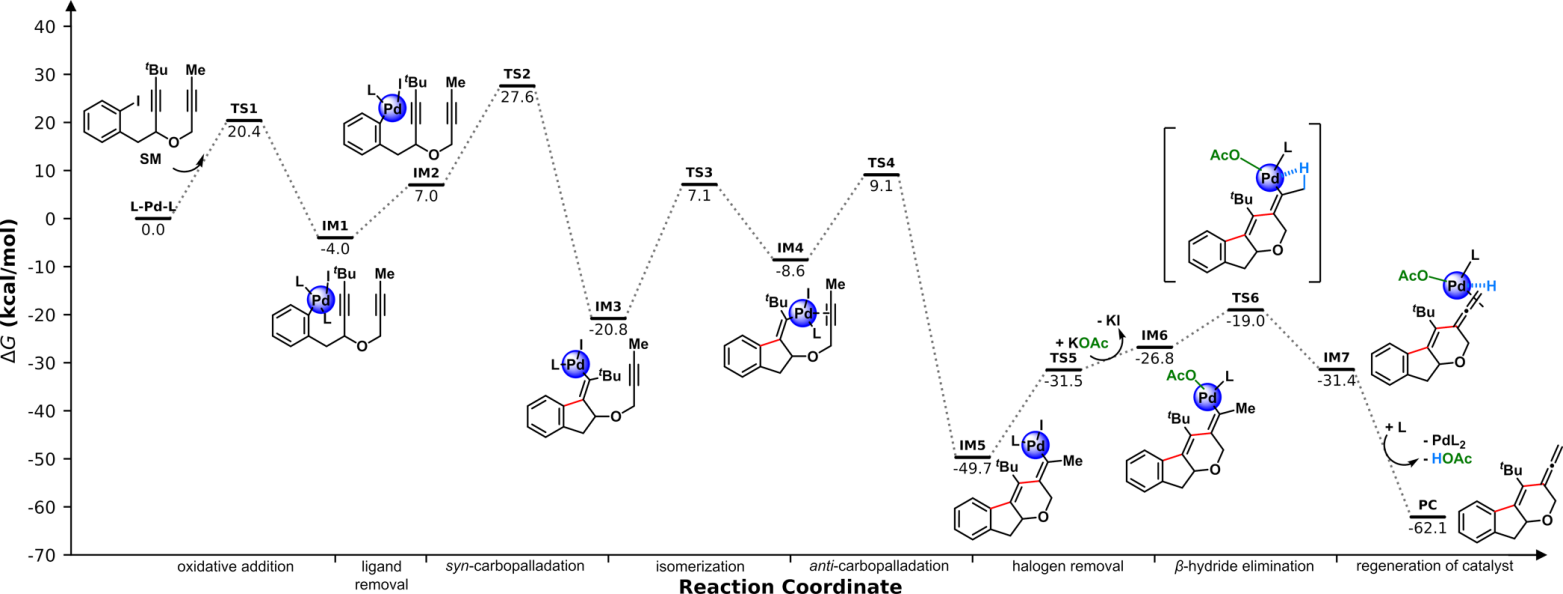
Energy Profiles and Geometries of Key Species for the Formation of Ene-Allenes

In summary, we developed a new approach to afford highly substituted ene-allenes embedded in oligocyclic ring systems in good yields. The designed alkyne systems undergo a novel palladium-catalyzed cascade process consisting of two carbopalladations, one being a formal *anti*-carbopalladation and the other being a *syn*-carbopalladation. The cascade is terminated intramolecularly by β-hydride elimination. Through the transformation, two carbon–carbon bonds are formed in an *anti*-fashion across the newly generated double bond. Moreover, a broad range of functional groups is well tolerated. To gain insight into the reaction mechanism, DFT calculations were carried out. Based on these computational studies, the key step is ligand exchange and the catalytic cycle terminates through β-hydride elimination.

## Supplementary Material



## Data Availability

The data underlying this study are available in the published article and its Supporting Information.

## Notes

Detailed experimental procedures, DFT calculations, analytical data, and ^1^H-, ^13^C-, and ^19^F-NMR spectra of all new compounds (PDF)
