# Supplementary material for: Ene-Allenes: Access from Dialkynes by an Intramolecular anti-Carbopalladation Cascade
Source: Org Lett. 2025 Nov 12;27(46):12769–73. doi: 10.1021/acs.orglett.5c03990 (PMC12645570; doi:10.1021/acs.orglett.5c03990)
Supplement: Supplementary file 1 [file ol5c03990_si_001.pdf]

Ene-Allenes: Access from Dialkynes by an Intramolecular *anti*-Carbopalladation Cascade

Pedram Kalvani,[a] Catherine Vanbel,[b] Heinrich F. von. Köller,[a] Daniel B. Werz*[a]

[a]Albert-Ludwigs-Universität Freiburg, Institute of Organic Chemistry, Albertstr. 21, 79104, Freiburg, Germany

[b]Vrije Universiteit Brussel, Organic Chemistry Research Group, Pleinlaan 2, 1050 Brussels, Belgium

(*corresponding author: daniel.werz@chemie.uni-freiburg.de)

Table of Contents

1. General Experimental S2
2. General Procedures S3
3. Optimization of the Allene Synthesis S5
4. Starting Material Synthesis and Characterization S8
5. The Formation and Characterization of Cascade Products S69
6. Supplementary Data for DFT Calculations S115
7. References S158

1. General Experimental

All air- and moisture-sensitive reactions were carried out in oven-dried or flame-dried glassware, septum-capped under atmospheric pressure of dry argon. Catalytic reactions were performed in Schlenk tubes placed in vial slots of an aluminum heating block with silicon oil in. For work-up and isolation of the products, standard techniques on the bench top were used. All reaction solvents were purchased as dry or extra dry in a bottle with molecular sieves. Solvents (*n*-pentane and ethyl acetate) for column chromatography were distilled before use. In addition, DMF used in allene formation reactions was degassed via three freeze-pump-thaw cycles daily. Commercially available compounds were used as received without further purification unless otherwise stated. $Pd_2(dba)_3$ was purchased from Sigma-Aldrich and *tert*-Bu-DavePhos from ABCR. KOAc applied in catalytic reactions kept in an argon-filled Schlenk tube and dried overnight under high vacuum before used. Reactions were monitored using thin layer chromatography (TLC). Thin-layer chromatography was carried out on Silica gel 60 F_{254} aluminum sheets from Merck. The visualization of spots was achieved by UV light at a wavelength of λ = 254 nm or $KMNO_4$ stain. For all allene purifications, either automated or manual flash column chromatography were performed. The automated flash column chromatography was conducted on CombiFlash Rf 200 via FlashPure EcoFlex Silica 12 g (50 µm, irregular particles) from BUCHI. For manual flash column chromatography, silica gel 60 (40 – 63 µm pore size) from Macherey-Nagel was used.

Proton (^{1}H), carbon (^{13}C), and fluorine (^{19}F) NMR spectra were recorded on 500 MHz Bruker DRX 500 or 400 MHz Bruker Advance II 400 Instrument using the residual signals from $CHCl_3$, δ = 7.26 ppm and δ = 77.16 ppm as internal reference for ^{1}H and ^{13}C chemical shifts, respectively. Additionally, tetramethylsilane (TMS; δ= 0.00 ppm; 0.03%) was added to NMR samples. The following abbreviations were used for ^{1}H, ^{13}C, and ^{19}F NMR chemical shifts: s = singlet, d = doublet, t = triplet, q = quartet, m = multiplet. The chemical shift δ is given in ppm. HRMS spectra were carried out on an Agilent 7890B GC System coupled to a 6545 LC/Q-TOF mass spectrometer (APCI measurements with Q-TOF analyzer). ATR-FTIR spectroscopy was carried out on a Spectrum Two FT-IR Spectrometer from Perkin Elmer. Samples were measured neat on a diamond ATR crystal. Transmission-bands are given in cm^{-1}. Melting points of solid products were measured on a Schorpp MPM-HV2 using the open capillary method. Exact reaction conditions are given in the following procedures.

2. General Procedures

GP1: Addition of an Alkyne to an Aldehyde

To a solution of alkyne (20.0-35.0 mmol, 2.0-3.5 eq.) in THF (50-75 mL, 0.4 M), *n*-BuLi (2.5 M in hexane, 1.5-2.5 eq.) was added dropwise at −78 °C. The mixture was stirred for 1 h at −78 °C before a solution of aldehyde (10.0 mmol, 1.0 eq.) in THF (35 mL, 0.3 M) was added dropwise. The mixture was slowly warmed up to r.t. overnight. Afterwards sat. aq. NH_4Cl-solution was added and the layers were separated. The aqueous layer was extracted three times with EtOAc. The combined organic layers were dried over Na_2SO_4, filtered, and concentrated *in vacuo*. The residue was purified by silica gel column chromatography.

GP2: Nucleophilic Substitution

The corresponding alcohol/amine (1.0 mmol, 1.0 eq.), the bromide derivative (1.1-1.5 mmol, 1.1-1.5 eq.) and TBAI (0.05-0.20 mmol, 5.0-20.0 mol%) were dissolved in DMF (20 mL, 0.05 M) and the solution was cooled to 0 °C. NaH (1.3-1.6 mmol, 1.3-1.6 eq.) was added and the solution was allowed to stir overnight at ambient temperature. Then, sat. aq. NH_4Cl-solution was added to the mixture and the aqueous layer was extracted three times with EtOAc. The combined organic layers were washed with sat. aq. NaCl solution, dried over Na_2SO_4, filtered, and concentrated *in vacuo*. The residue was purified by silica gel column chromatography.

GP3: Mitsunobu Reaction

The corresponding alcohol (1.2 mmol, 1.0 eq.) was dissolved in THF (12 mL, 0.1 M), and the mixture was cooled down to 0 °C. Afterwards, halophenol derivatives (1.2 mmol, 1.2 eq.) and PPh_3 (1.4-1.7 mmol, 1.4-1.7 eq.) were added. DIAD (1.6-1.8 mmol, 1.6-1.8 eq.) was added dropwise, and the mixture was stirred for 0.5 h at 0 °C and then at r.t. until TLC showed full conversion of the starting material. After removal of the solvent *in vacuo*, the residue was purified by silica gel column chromatography.

GP4: Silyl Deprotection

The TBDMS-protected alcohol (1.56 mmol, 1.0 eq.) was dissolved in a mixture of CH_2Cl_2:MeOH (each 1.5 mL, 1.0 M). At 0 °C, acetyl chloride (6.55-9.36 mmol, 4.2-6.0 eq.) was added dropwise. The mixture was stirred for 1 h at 0 °C and afterwards at r.t. until TLC showed full conversion of the starting material. The mixture was quenched with sat. aq. $NaHCO_3$ solution and the layers

were separated. The aqueous layer was extracted three times with EtOAc. The combined organic layers were washed with sat. aq. NaCl solution, dried over Na_2SO_4, filtered, and concentrated *in vacuo*. The residue was purified by silica gel column chromatography.

GP5: *anti*-Carbopalladation/*β*-Hydride Elimination Cascade

The corresponding domino precursor (0.1 mmol, 1.0 eq.), $Pd_2(dba)_3$ (5 µmol, 5.0 mol%), *tert*-Bu-DavePhos (10 µmol, 10.0 mol%), and KOAc (0.75 mmol, 7.5 eq.) were sequentially added to an oven-dried Schlenk tube. Then, the tube was sealed with a rubber septum, evacuated and backfilled with argon three times using Schlenk line. Next, DMF (8 mL, 12 mM) was added under an argon atmosphere. Then, tube placed in an aluminum heating block preheated to 140 °C. The reaction mixture was stirred at this temperature for 1 h with the stirring set to 600 rpm. After cooling down to r.t., the reaction was quenched with sat. aq. NH_4Cl-solution. The aqueous phase was extracted three times with EtOAc. The organic phase was washed with H_2O and sat. aq. NaCl-solution, dried over Na_2SO_4, filtered, and concentrated *in vacuo*. The residue was purified by automated or manual flash column chromatography.

Note: For purification of final domino compounds, mostly two flash column chromatography must be performed.

3. Optimization of the Allene Synthesis

Table S1. The effect of base[a]

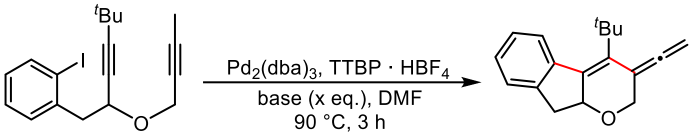

1a 2a

Entry	Base	X eq. of base	Conversion [%][b]	Yield [%][b]
1	Cs_2CO_3	5	100	5
2	K_2CO_3	5	100	trace
3	Na_2CO_3	5	93	trace
4	Ag_2CO_3	5	100	trace
5	CsOAc	5	100	27
6	KOAc	5	93	47
**7**	**KOAc**	**7.5**	**100**	**51**
8	KOAc	3	95	47
9	KOAc	1.2	95	26
10	NaOAc	5	79	17
11	TBAOAc[c]	5	100	28
12	NaO*t*Bu	5	100	n.p.[d]
13	K_3PO_4	5	88	5
14	KOPiv	5	100	22
15	TEA	5	98	15

[a]Reaction scale: 0.05 mmol, $Pd_2(dba)_3$ (10.0 mol%), TTBP · HBF_4 (20.0 mol%), DMF (0.025 M). [b]The conversion and yield was determined by integrating the appropriate peaks in the crude ^1H NMR using 1,3,5- trimethoxybenzene as an internal standard. [c]TBAOAc = tetrabutylammonium acetate. [d]n.p. = no product is formed.

Table S2. The effect of temperature, time, solvent and concentration[a]

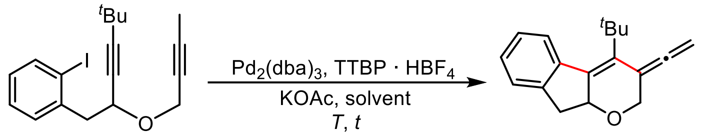

1a **2a**

Entry	Solvent	T (°C)	Conversion [%][b]	Yield [%][b]
1	DMF	70	74	20
2	DMF	110	100	55
3	DMF	120	100	56
4	DMF	140	99	62
5	DMF	160	100	52
6	Toluene	140	100	n.p.[c]
7	MeCN	140	100	24
8	PhCl	140	94	n.p.
9	o-xylene	140	90	n.p.
10	THF	140	100	n.p.
11	DMF (0.1 M)	140	100	46
12	DMF (0.012 M)	140	100	67
**13[d]**	**DMF (0.012 M)**	**140**	**100**	**67 (57)**[e]
14[f]	DMF (0.012 M)	140	100	63

[a]Reaction scale: 0.05 mmol, $Pd_2(dba)_3$ (10.0 mol%), TTBP · HBF_4 (20.0 mol%), KOAc (7.5 eq.), solvent (0.025 M), 3 h. [b]The conversion and yield was determined by integrating the appropriate peaks in the crude ^1H NMR using 1,3,5- trimethoxybenzene as an internal standard. [c]n.p. = no product is formed. [d]Reaction time decreased to 1 h. [e]Isolated yield. [f]Reaction time increased to 18 h.

Table S3. The effect of catalyst and ligand[a]

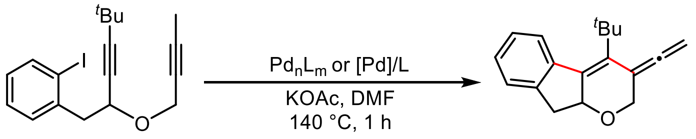

1a **2a**

Entry	Pd_nL_m or [Pd]/L	Conversion [%][b]	Yield [%][b]
1	$Pd_2(dba)_3$ (10 mol%)/*tert*-Bu-XPhos (20 mol%)	86	60
2	$Pd_2(dba)_3$ (10 mol%)/JackiePhos (20 mol%)	100	62
3	$Pd_2(dba)_3$ (10 mol%)/QPhos (20 mol%)	100	58
4	$Pd_2(dba)_3$ (10 mol%)/Ipr[c] (20 mol%)	100	48
5	$Pd_2(dba)_3$ (10 mol%)/DavePhos (20 mol%)	100	67
6	$Pd(OAc)_2$ (10 mol%)/DavePhos (20 mol%)	100	61
7	$Pd[PPh_3]_4$ (10 mol%)	99	34
8	QPhos Pd G3 (10 mol%)	100	66
9	$Pd_2(dba)_3$ (5 mol%)/DavePhos (10 mol%)	100	67
10	$Pd_2(dba)_3$ (5 mol%)/*tert*-Bu-DavePhos (10 mol%)	100	**79 (71)**[d]
11	$Pd(OAc)_2$ (5 mol%)/ *tert*-Bu-DavePhos (10 mol%)	100	64
12	$Pd_2(dba)_3$ (2.5 mol%)/*tert*-Bu-DavePhos (5 mol%)	100	75

[a]Reaction scale: 0.05 mmol, KOAc (7.5 eq.), DMF (0.012 M). [b]The conversion and yield was determined by integrating the appropriate peaks in the crude ^1H NMR using 1,3,5-trimethoxybenzene as an internal standard. [c]Ipr = 1,3-Bis(2,6-diisopropylphenyl)-1,3-dihydro-2*H*-imidazol-2-ylidene. [d]Isolated yield.

4. Starting Material Synthesis and Characterization

1-(2-halophenyl)-5,5-dimethylhex-3-yn-2-ol (S4-S5)

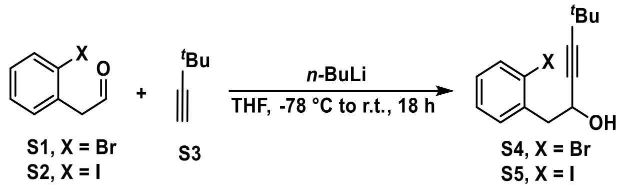

2-(2-bromophenyl)acetaldehyde **S1**[1] (2.23 g, 11.2 mmol, 1.0 eq.) in THF (39 mL, 0.3 M), 3,3-dimethylbut-1-yne **S3** (3.2 g, 4.7 mL, 39.2 mmol, 3.5 eq.) in THF (98 mL, 0.4 M), and *n*-BuLi (2.5 M in hexane, 11.2 mL, 28.01 mmol, 2.5 eq.) were reacted according to **GP1**. The reaction mixture was stirred at r.t. for 18 h. Silica gel column chromatography (*n*-pentane:EtOAc = 10:1) afforded desired compound **S4** (1.84 g, 6.54 mmol, 58%) as yellow oil. The characterization data are consistent with the literature.[2]

2-(2-Iodophenyl)acetaldehyde **S2**[3] (2.00 g, 8.13 mmol, 1.0 eq.) in THF (28.5 mL, 0.3 M), 3,3-dimethylbut-1-yne **S3** (2.30 g, 3.5 mL, 28.46 mmol, 3.5 eq.) in THF (71 mL, 0.4 M), and *n*-BuLi (2.5 M in hexane, 8.1 mL, 20.33 mmol, 2.5 eq.) were reacted according to **GP1**. The reaction mixture was stirred at r.t. for 18 h. Silica gel column chromatography (*n*-pentane:EtOAc = 20:1) afforded desired compound **S5** (1.85 g, 5.64 mmol, 69%) as light-yellow oil. The characterization data are consistent with the literature.[4]

1-bromo-2-(2-(but-2-yn-1-yloxy)-5,5-dimethylhex-3-yn-1-yl)benzene (1a)

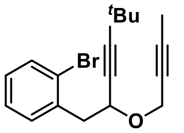

The alcohol **S4** (700 mg, 2.49 mmol, 1.0 eq.), 1-bromobut-2-yne[5] (380.7 mg, 250 µL, 2.86 mmol, 1.1 eq.), TBAI (184 mg, 0.50 mmol, 20.0 mol%), and NaH (60% on mineral oil, 149.4 mg, 3.73 mmol, 1.5 eq.) in DMF (50 mL, 0.05 M) were reacted according to **GP2**. The reaction mixture was stirred at r.t. for 15 h. Silica gel column chromatography (*n*-pentane:EtOAc = 30:1) afforded domino precursor **1a** (700 mg, 2.1 mmol, 84%) as colorless oil.

R_f = 0.6 (*n*-pentane:EtOAc = 30:1).

^{1}H-NMR (500 MHz, $CDCl_3$): δ = 7.52 (dd, J = 8.0, 1.3 Hz, 1H), 7.35 (dd, J = 7.6, 1.8 Hz, 1H), 7.23 (td, J = 7.5, 1.3 Hz, 2H), 7.08 (ddd, J = 7.9, 7.3, 1.7 Hz, 1H), 4.55 (dd, J = 8.0, 6.2 Hz, 1H), 4.30 (dq, J = 15.0, 2.4 Hz, 1H), 4.16 (dq, J = 15.0, 2.3 Hz, 1H), 3.28 (dd, J = 13.3, 6.2 Hz, 1H), 3.02 (dd, J = 13.3, 8.0 Hz, 1H), 1.84 (t, J = 2.3 Hz, 3H), 1.16 (s, 9H).

^{13}C-NMR (126 MHz, $CDCl_3$): δ = 136.9, 132.6, 132.6, 128.3, 127.1, 125.1, 96.3, 82.5, 76.0, 75.1, 67.8, 56.4, 42.4, 31.0, 27.5, 3.8.

IR (ATR): $\tilde{\nu}$ (cm^{-1}) = 2968, 1723, 1473, 1440, 1390, 1362, 1337, 1262, 1203, 1157, 1138, 1064, 1027.

HRMS (APCI, Q-TOF): calculated for $C_{18}H_{22}BrO^+$ $[M+H]^+$: 333.0849, found: 333.0847.

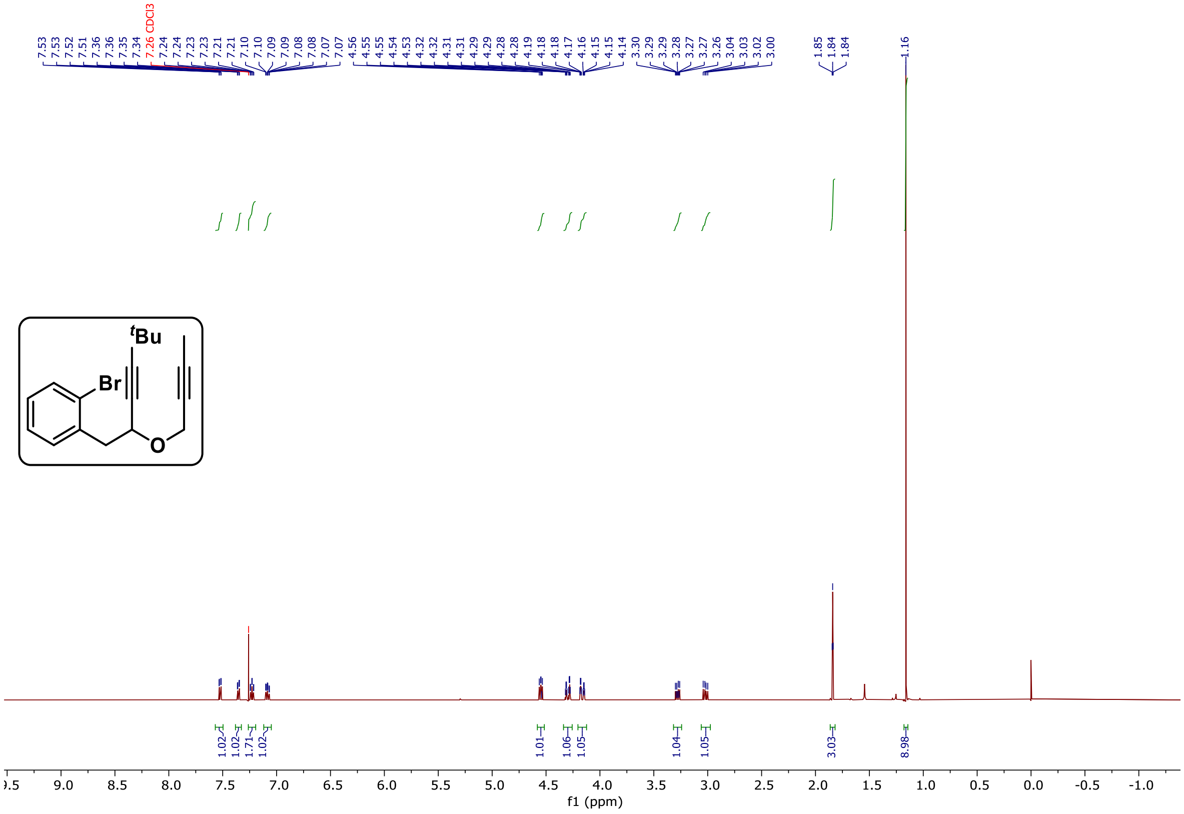

^{1}H-NMR (500 MHz, $CDCl_3$)

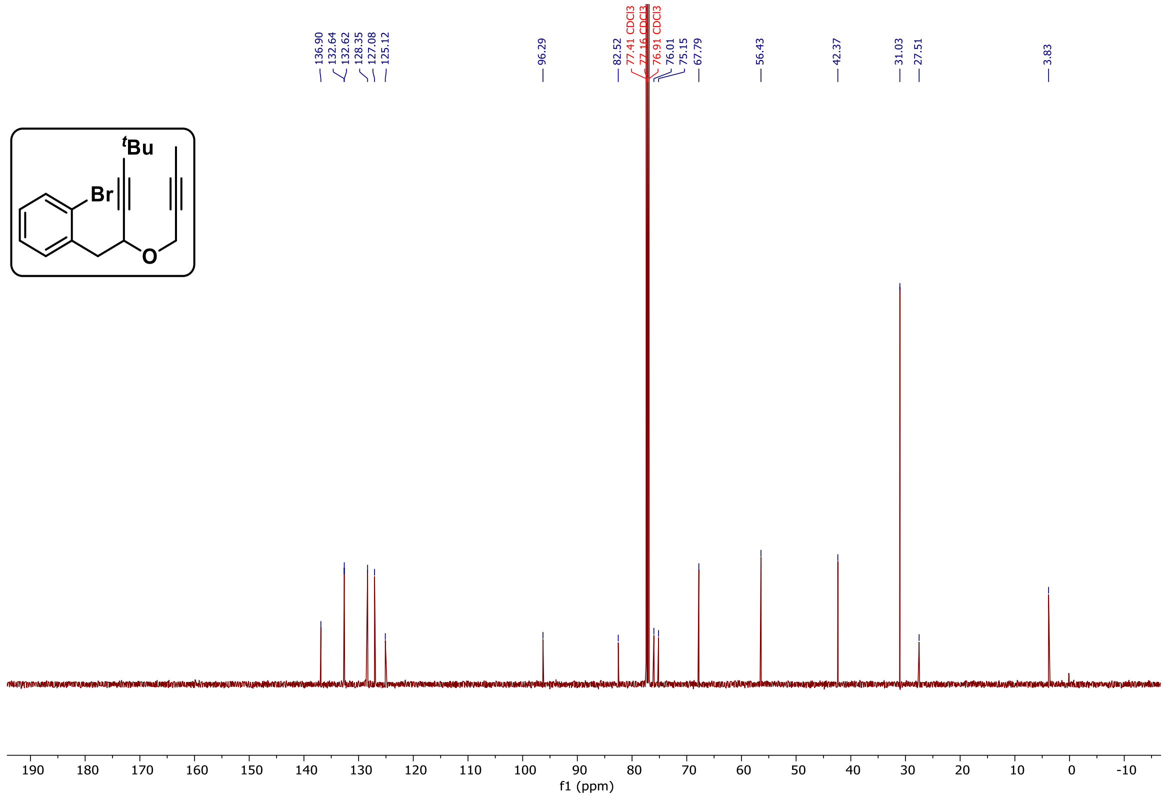

^{13}C-NMR (126 MHz, $CDCl_3$)

1-(2-(but-2-yn-1-yloxy)-5,5-dimethylhex-3-yn-1-yl)-2-iodobenzene (1a')

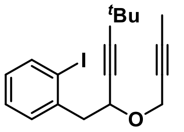

The alcohol **S5** (680 mg, 2.07 mmol, 1.0 eq.), 1-bromobut-2-yne (351 mg, 230 µL, 2.64 mmol, 1.3 eq.), TBAI (153 mg, 0.41 mmol, 20.0 mol%), and NaH (60% on mineral oil, 132 mg, 3.3 mmol, 1.6 eq.) in DMF (41.5 mL, 0.05 M) were reacted according to **GP2**. The reaction mixture was stirred at r.t. for 15 h. Silica gel column chromatography (*n*-pentane:EtOAc = 50:1) afforded domino precursor **1a'** (550 mg, 1.44 mmol, 70%) as colorless oil.

$\mathbf{R_f}$ = 0.32 (*n*-pentane:EtOAc = 50:1).

^{1}H-NMR (400 MHz, $CDCl_3$): δ = 7.81 (dd, J = 8.0, 1.2 Hz, 1H), 7.35 (ddd, J = 7.7, 1.8, 0.4 Hz, 1H), 7.30 – 7.22 (m, 1H), 6.91 (ddd, J = 7.9, 7.3, 1.8 Hz, 1H), 4.53 (dd, J = 8.2, 6.1 Hz, 1H), 4.30 (dq, J = 15.1, 2.4 Hz, 1H), 4.17 (dq, J = 15.0, 2.3 Hz, 1H), 3.24 (dd, J = 13.4, 6.1 Hz, 1H), 3.03 (dd, J = 13.4, 8.1 Hz, 1H), 1.84 (t, J = 2.3 Hz, 3H), 1.17 (s, 9H).

^{13}C-NMR (101 MHz, $CDCl_3$): δ = 140.2, 139.3, 131.8, 128.4, 127.9, 101.3, 96.3, 82.5, 75.9, 75.1, 67.9, 56.4, 46.6, 31.0, 27.5, 3.8.

IR (ATR): $\tilde{v}$ (cm^{-1}) = 2967, 1467, 1436, 1361, 1336, 1262, 1203, 1138, 1064, 1012.

HRMS (APCI, Q-TOF): calculated for $C_{18}H_{25}ONI^+$ $[M+NH_4]^+$: 398.0975, found: 398.0976.

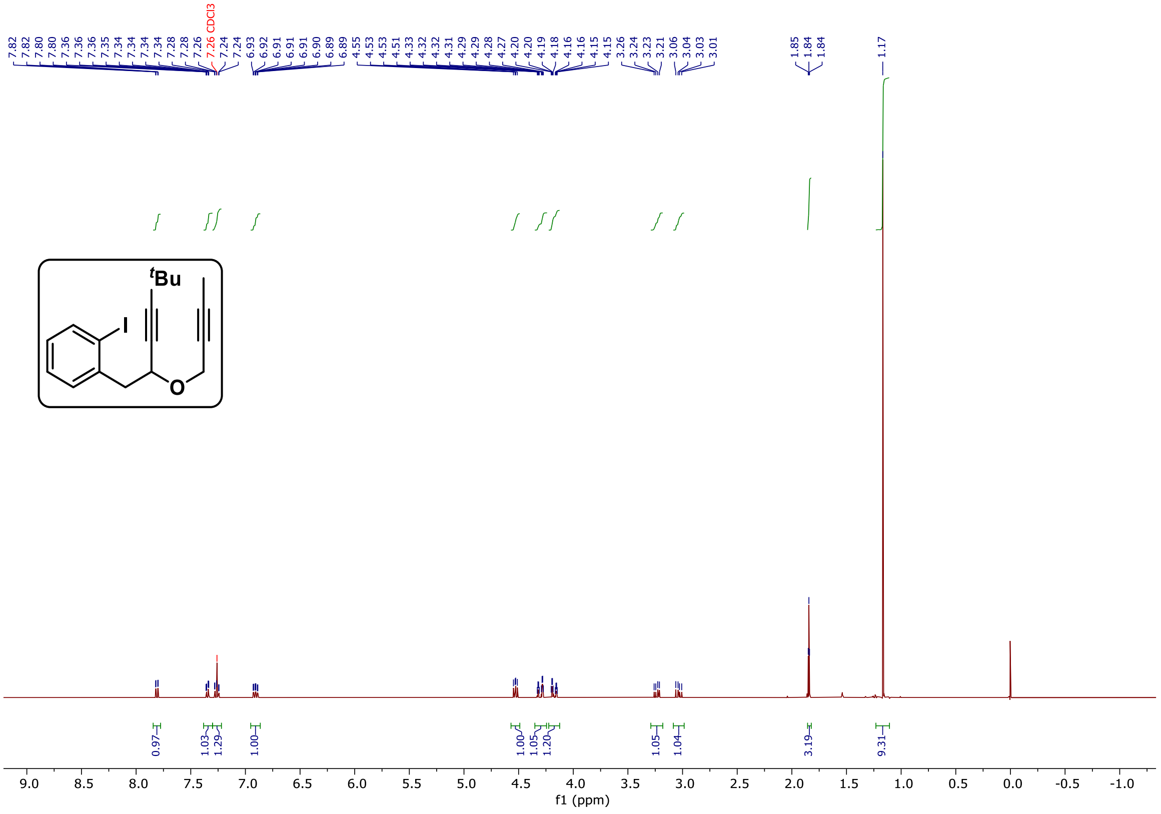

^{1}H-NMR (400 MHz, $CDCl_3$)

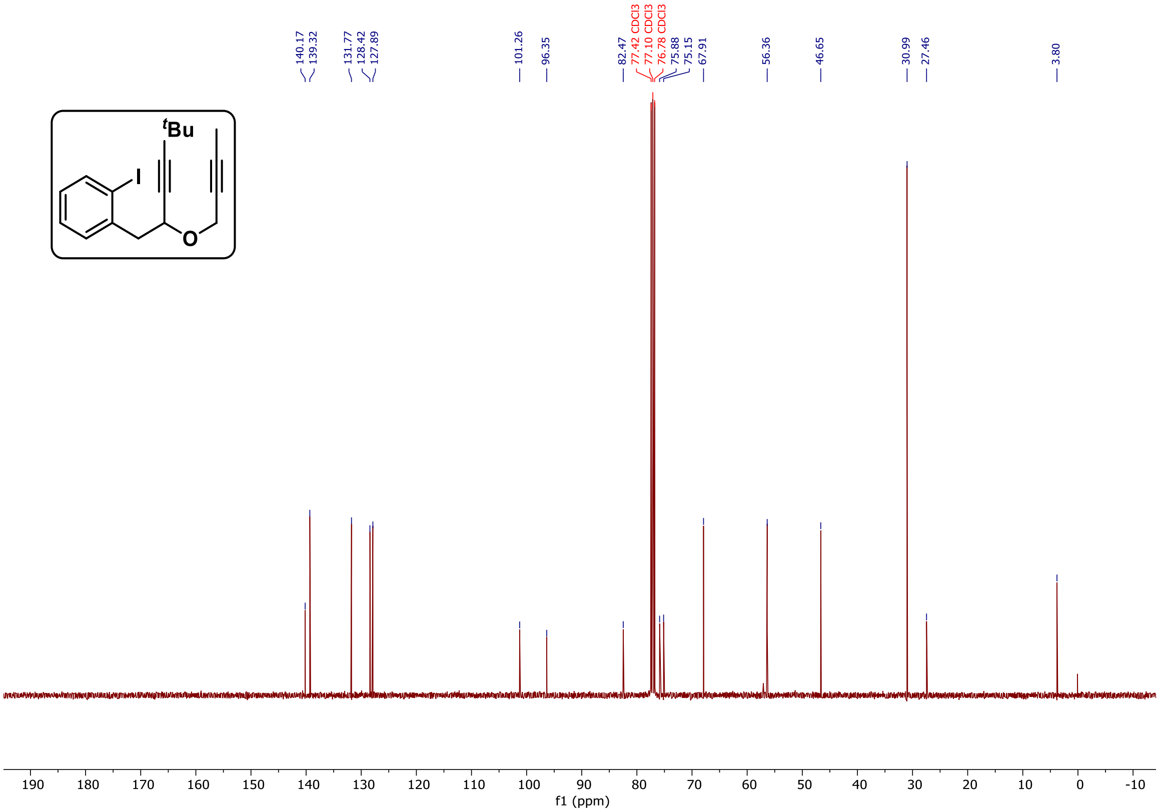

^{13}C-NMR (101 MHz, $CDCl_3$)

1-bromo-2-(5,5-dimethyl-2-(pent-2-yn-1-yloxy)hex-3-yn-1-yl)benzene (1b)

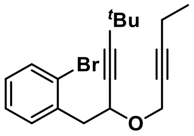

The alcohol **S4** (281.2 mg, 1.0 mmol, 1.0 eq.), 1-bromopent-2-yne (176.4 mg, 123 µL, 1.2 mmol, 1.2 eq.), TBAI (73.9 mg, 0.2 mmol, 20.0 mol%), and NaH (60% on mineral oil, 60 mg, 1.5 mmol, 1.5 eq.) in DMF (20 mL, 0.05 M) were reacted according to **GP2**. The reaction mixture was stirred at r.t. for 16 h. Silica gel column chromatography (*n*-pentane:EtOAc = 40:1) afforded domino precursor **1b** (220 mg, 0.63 mmol, 63%) as yellow oil.

$\mathbf{R_f}$ = 0.33 (*n*-pentane:EtOAc = 40:1).

^{1}H-NMR (500 MHz, $CDCl_3$): δ = 7.52 (dd, J = 8.0, 1.3 Hz, 1H), 7.36 (dd, J = 7.6, 1.8 Hz, 1H), 7.23 (td, J = 7.5, 1.3 Hz, 2H), 7.08 (ddd, J = 7.9, 7.3, 1.8 Hz, 1H), 4.56 (dd, J = 8.0, 6.3 Hz, 1H), 4.31 (dt, J = 15.1, 2.2 Hz, 1H), 4.19 (dt, J = 15.1, 2.1 Hz, 1H), 3.28 (dd, J = 13.3, 6.3 Hz, 1H), 3.02 (dd, J = 13.3, 8.0 Hz, 1H), 2.21 (qt, J = 7.5, 2.2 Hz, 2H), 1.16 (s, 9H), 1.13 (t, J = 7.5 Hz, 3H).

^{13}C-NMR (126 MHz, $CDCl_3$): δ = 136.9, 132.6, 128.3, 127.0, 125.0, 96.1, 88.3, 76.0, 75.2, 67.6, 56.3, 42.3, 31.0, 27.4, 13.9, 12.6.

IR (ATR): $\tilde{\nu}$ (cm^{-1}) = 2970, 2234, 1723, 1568, 1473, 1441, 1362, 1336, 1319, 1262, 1204, 1136, 1065, 1028.

HRMS (APCI, Q-TOF): calculated for $C_{19}H_{24}BrO^+$ $[M+H]^+$: 347.1005, found: 347.1006.

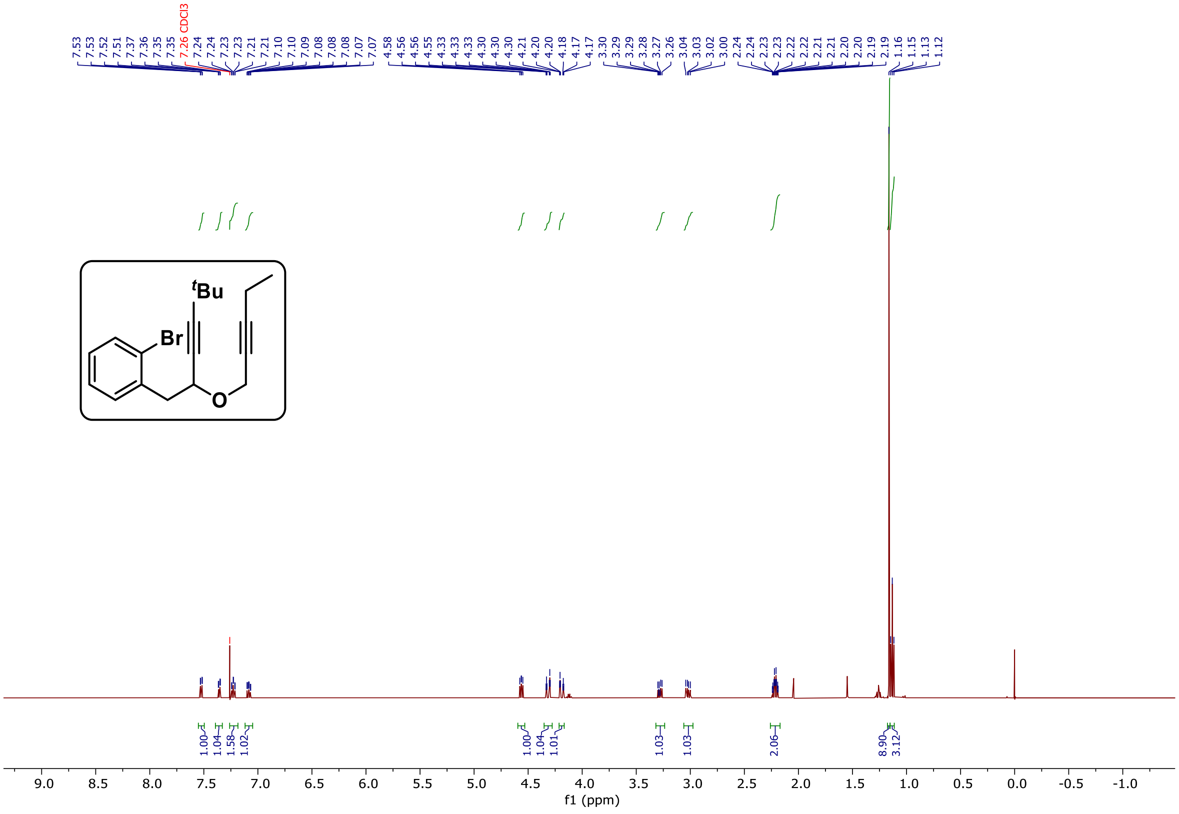

^{1}H-NMR (500 MHz, $CDCl_3$)

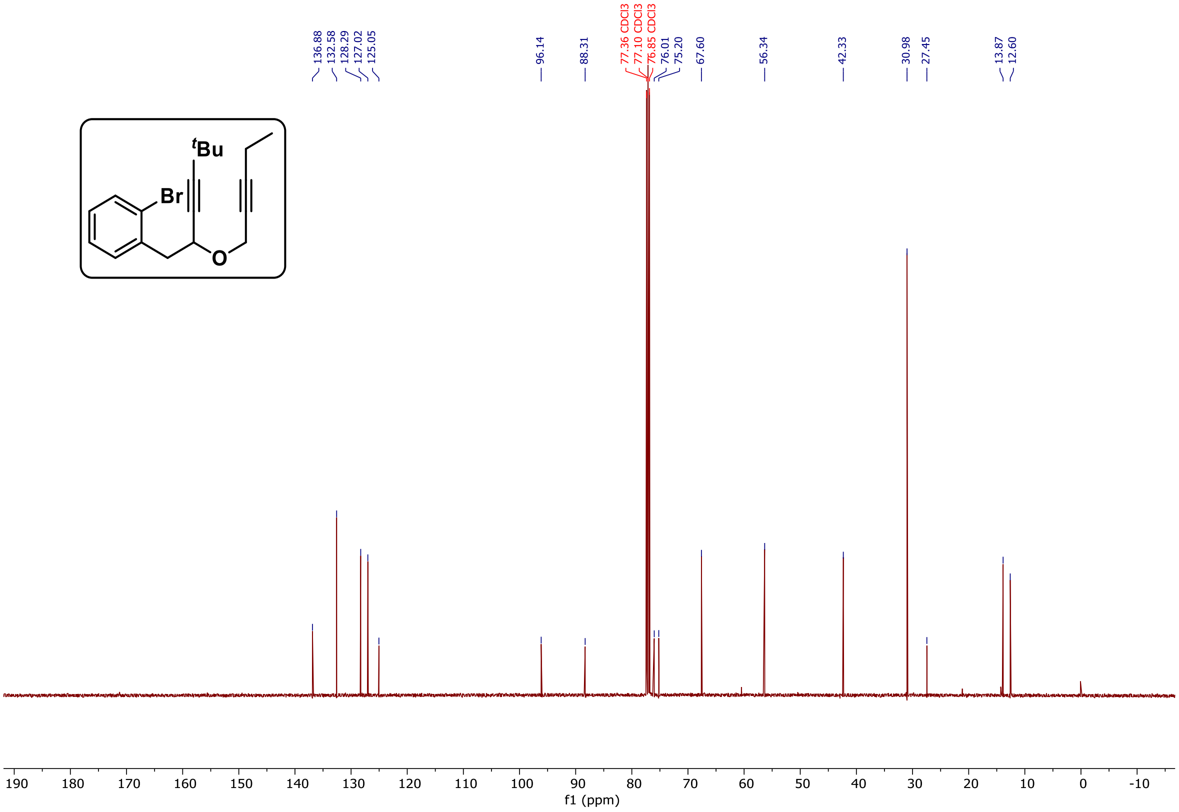

^{13}C-NMR (126 MHz, $CDCl_3$)

1-bromo-2-(5,5-dimethyl-2-((4-methylpent-2-yn-1-yl)oxy)hex-3-yn-1-yl)benzene (1c)

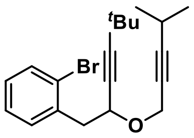

The alcohol **S4** (281.2 mg, 1.0 mmol, 1.0 eq.), 1-bromo-4-methylpent-2-yne[6] (240 mg, 1.49 mmol, 1.5 eq.), TBAI (73.9 mg, 0.2 mmol, 20.0 mol%), and NaH (60% on mineral oil, 60 mg, 1.5 mmol, 1.5 eq.) in DMF (20 mL, 0.05 M) were reacted according to **GP2**. The reaction mixture was stirred at r.t. for 17 h. Silica gel column chromatography (*n*-pentane:EtOAc = 40:1) afforded domino precursor **1c** (150 mg, 0.41 mmol, 42%) as yellow oil.

$\mathbf{R_f}$ = 0.36 (*n*-pentane:EtOAc = 40:1).

^{1}H-NMR (400 MHz, $CDCl_3$): δ = 7.52 (dd, *J* = 8.0, 1.3 Hz, 1H), 7.36 (dd, *J* = 7.6, 1.7 Hz, 1H), 7.23 (td, *J* = 7.5, 1.3 Hz, 1H), 7.10 – 7.05 (m, 1H), 4.57 (dd, *J* = 7.8, 6.4 Hz, 1H), 4.31 (dd, *J* = 15.2, 2.1 Hz, 1H), 4.22 – 4.18 (m, 1H), 3.28 (dd, *J* = 13.4, 6.4 Hz, 1H), 3.02 (dd, *J* = 13.4, 7.8 Hz, 1H), 2.58 (ddtt, *J* = 13.8, 8.9, 6.9, 2.0 Hz, 1H), 1.17 (s, 9H), 1.16 (d, *J* = 1.1 Hz, 4H), 1.15 (d, *J* = 1.0 Hz, 3H).

^{13}C-NMR (101 MHz, $CDCl_3$): δ = 137.0, 132.6, 132.6, 128.3, 127.1, 125.1, 96.1, 92.5, 76.2, 75.1, 67.5, 56.3, 42.4, 31.1, 27.5, 23.1, 20.7.

IR (ATR): $\tilde{\nu}$ (cm^{-1}) = 2969, 2932, 2869, 1724, 1568, 1471, 1441, 1362, 1319, 1262, 1203, 1184, 1159, 1124, 1072, 1029.

HRMS (APCI, Q-TOF): calculated for $C_{20}H_{26}BrO^+$ $[M+H]^+$: 361.1161, found: 361.1162.

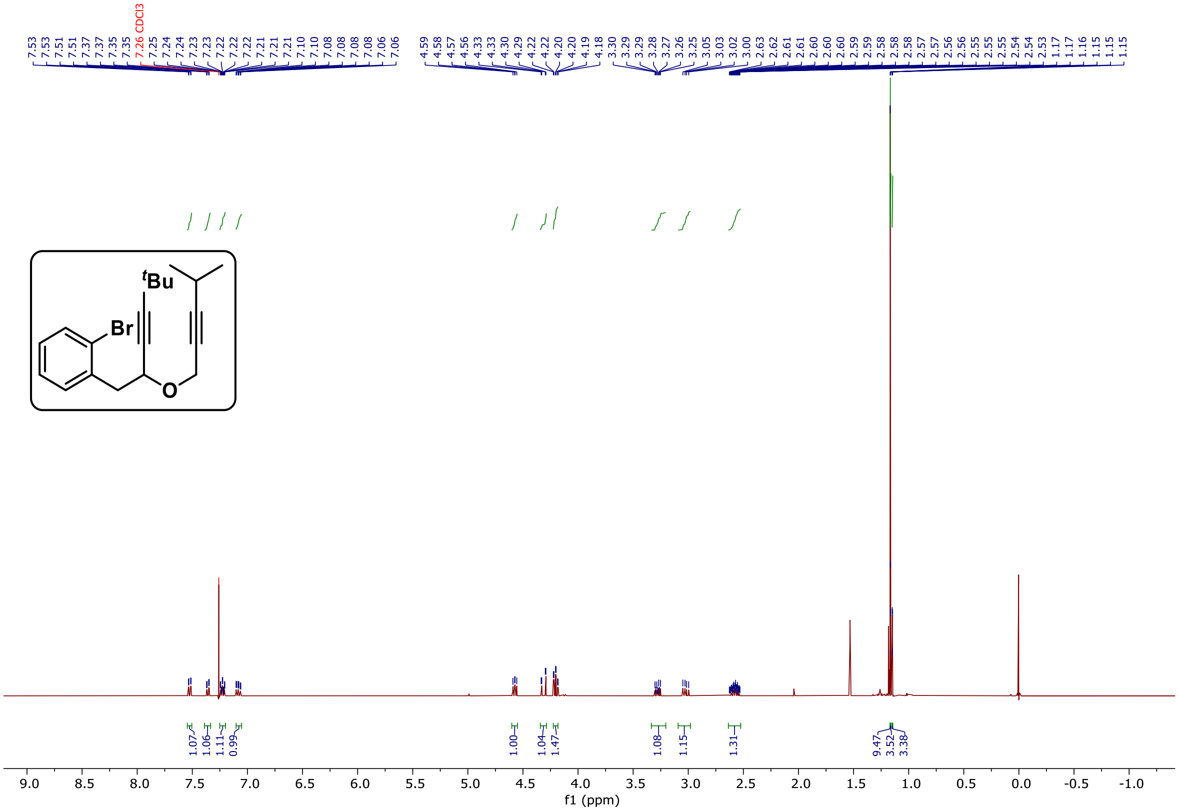

^{1}H-NMR (400 MHz, $CDCl_3$)

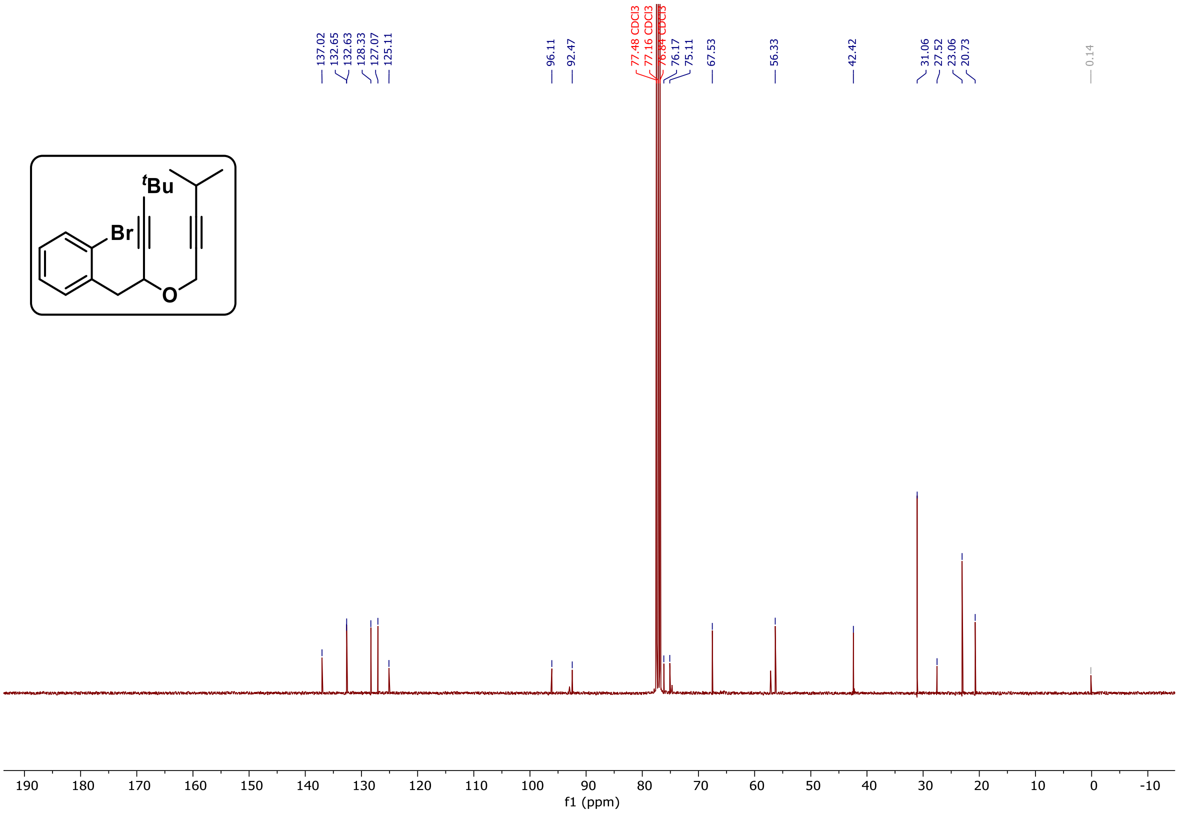

^{13}C-NMR (101 MHz, $CDCl_3$)

N-(but-2-yn-1-yl)-N-(1-(2-iodophenyl)-5,5-dimethylhex-3-yn-2-yl)-4-methylbenzenesulfonamide (1d)

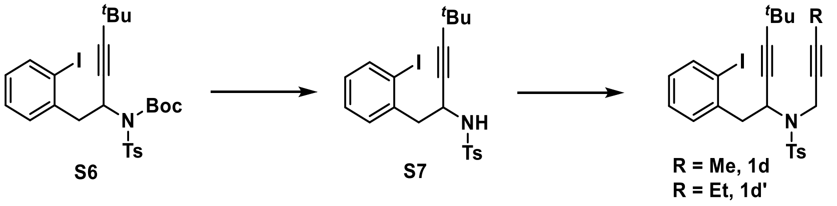

The boc-deprotected amine **S7**[7] (560 mg, 1.16 mmol, 1.0 eq.), 1-bromobut-2-yne (185.6 mg, 122 µL, 1.40 mmol, 1.2 eq.), TBAI (85.9 mg, 0.23 mmol, 20.0 mol%), and NaH (60% on mineral oil, 69.8 mg, 1.74 mmol, 1.5 eq.) in DMF (23 mL, 0.05 M) were reacted according to **GP2**. The reaction mixture was stirred at r.t. for 22 h. Silica gel column chromatography (*n*-pentane:EtOAc = 40:1) afforded domino precursor **1d** (530 mg, 0.99 mmol, 85%) as highly viscous brown oil.

R_f = 0.29 (*n*-pentane:EtOAc = 40:1).

^{1}H-NMR (500 MHz, $CDCl_3$): δ = 7.81 (dd, J = 7.9, 1.3 Hz, 1H), 7.77 – 7.71 (m, 2H), 7.33 (dd, J = 7.6, 1.8 Hz, 1H), 7.30 – 7.21 (m, 3H), 6.91 (ddd, J = 7.9, 7.3, 1.8 Hz, 1H), 5.01 (t, J = 7.9 Hz, 1H), 4.21 – 4.13 (m, 1H), 4.01 (dq, J = 17.9, 2.4 Hz, 1H), 3.28 (d, J = 7.9 Hz, 2H), 2.39 (s, 3H), 1.77 (d, J = 4.8 Hz, 3H), 0.96 (s, 9H).

^{13}C-NMR (126 MHz, $CDCl_3$): δ = 143.3, 140.0, 139.5, 136.6, 131.5, 129.4, 128.6, 127.9, 127.9, 101.3, 96.3, 80.4, 77.4, 77.1, 76.8, 75.3, 73.5, 51.2, 46.2, 34.7, 30.6, 27.2, 21.5, 3.8.

IR (ATR): $\tilde{\nu}$ (cm^{-1}) = 1433, 1359, 1336, 1265, 1163, 1149, 1093, 1052, 1012.

HRMS (APCI, Q-TOF): calculated for $C_{25}H_{28}O_2NINaS^+$ $[M+Na]^+$: 556.0778, found: 556.0779.

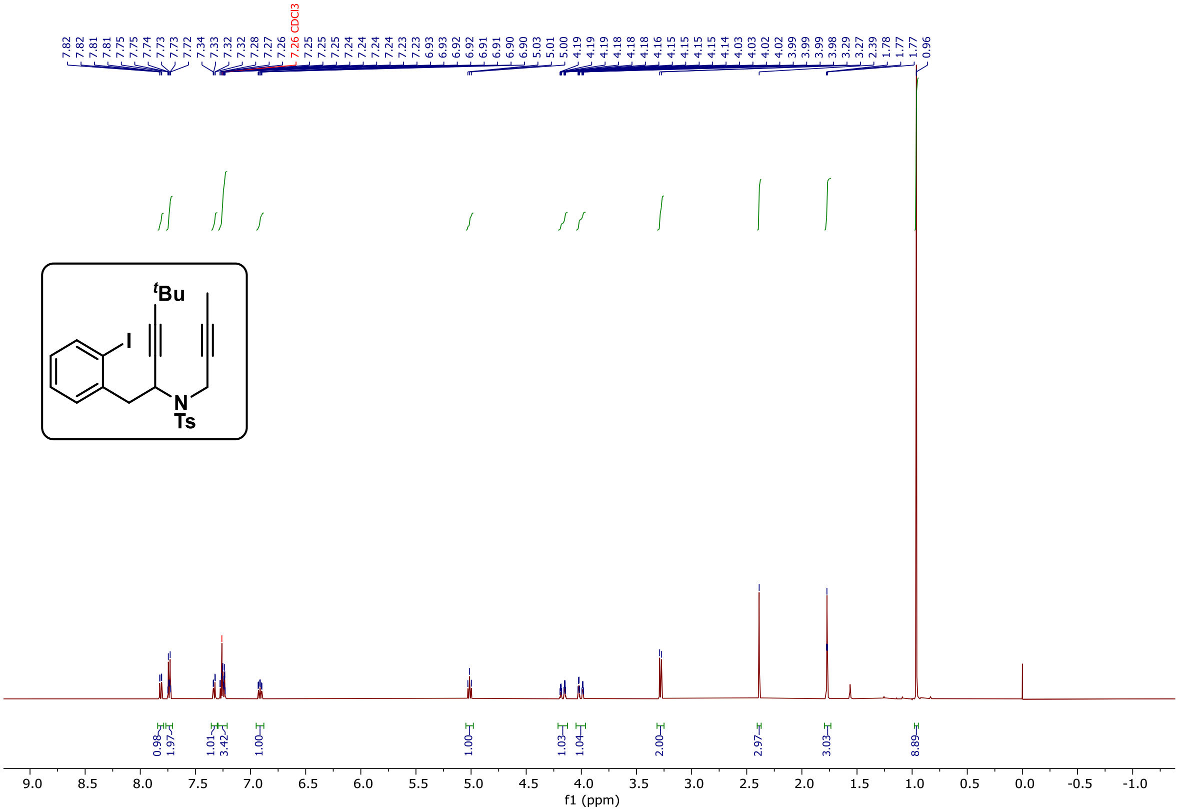

^{1}H-NMR (500 MHz, $CDCl_3$)

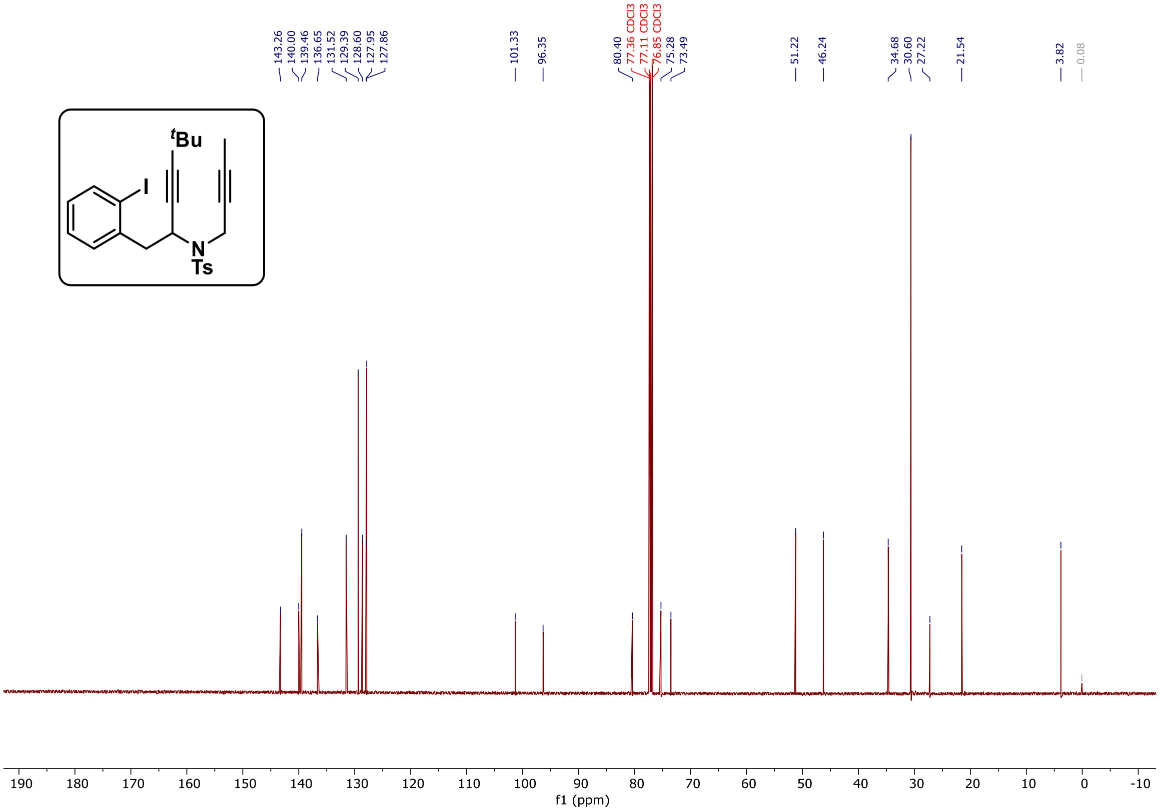

^{13}C-NMR (126 MHz, $CDCl_3$)

_N_-(1-(2-iodophenyl)-5,5-dimethylhex-3-yn-2-yl)-4-methyl-_N_-(pent-2-yn-1-yl)benzenesulfonamide (1d')

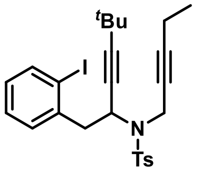

The boc-deprotected amine **S7** (179 mg, 0.37 mmol, 1.0 eq.), 1-bromopent-2-yne (66 mg, 43 µL, 0.45 mmol, 1.2 eq.), TBAI (28 mg, 0.07 mmol, 19.0 mol%), and NaH (60% on mineral oil, 22.4 mg, 0.56 mmol, 1.5 eq.) in DMF (7.5 mL, 0.05 M) were reacted according to **GP2**. The reaction mixture was stirred at r.t. for 22 h. Silica gel column chromatography (*n*-pentane:EtOAc = 40:1) afforded domino precursor **1d'** (151 mg, 0.28 mmol, 74%) as highly viscous colorless oil.

$\mathbf{R_f}$ = 0.4 (*n*-pentane:EtOAc = 40:1).

^{1}H-NMR (500 MHz, $CDCl_3$): δ = 7.82 – 7.80 (dd, *J* = 7.9, 1.3 Hz, 1H), 7.75 – 7.73 (m, 2H), 7.35 – 7.33 (dd, *J* = 7.7, 1.8 Hz, 1H), 7.26 – 7.22 (m, 3H), 6.93 – 6.90 (ddd, *J* = 7.9, 7.3, 1.8 Hz, 1H), 5.03 – 4.99 (dd, *J* = 9.0, 6.9 Hz, 1H), 4.25 – 4.24 (dtd, *J* = 17.9, 2.3, 0.6 Hz, 1H), 4.04 – 4.00 (dt, *J* = 17.9, 2.3 Hz, 1H), 3.33 – 3.25 (m, 2H), 2.39 (s, 3H), 2.19 – 2.13 (qt, *J* = 7.5, 2.3 Hz, 2H), 1.11 – 1.08 (t, *J* = 7.5 Hz, 3H), 0.97 (s, 9H).

^{13}C-NMR (126 MHz, $CDCl_3$): δ = 12.7, 13.7, 21.6, 27.3, 30.7, 34.8, 46.3, 51.4, 73.6, 75.6, 86.2, 96.4, 101.4, 127.9, 128.0, 128.6, 129.5, 131.6, 136.8, 139.5, 140.1, 143.3.

IR (ATR): $\tilde{\nu}$ (cm^{-1}) = 1357, 1338, 1158, 1115, 1091, 1052, 1012.

HRMS (APCI, Q-TOF): calculated for $C_{26}H_{31}INO_2S^+$ $[M+H]^+$: 548.1115, found 548.1121.

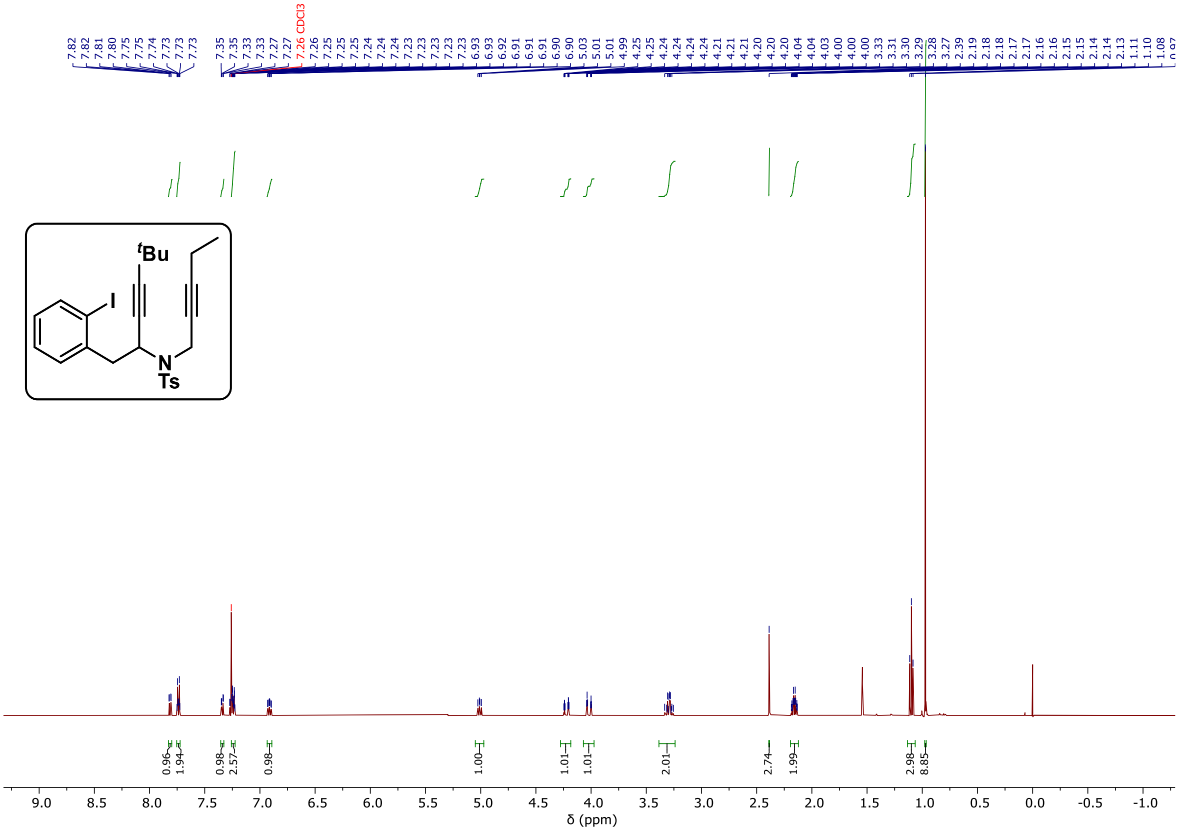

^{1}H-NMR (500 MHz, $CDCl_3$)

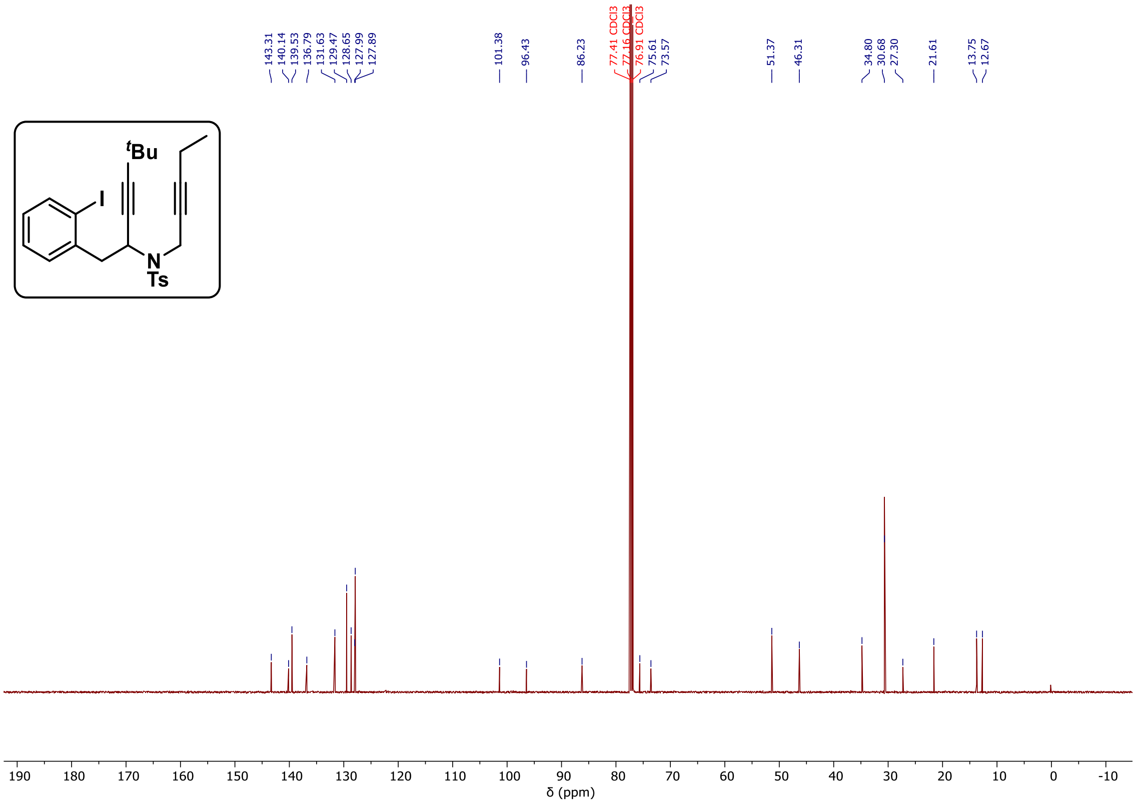

^{13}C-NMR (126 MHz, $CDCl_3$)

2,2-dimethyldeca-3,8-diyn-5-ol (S11)

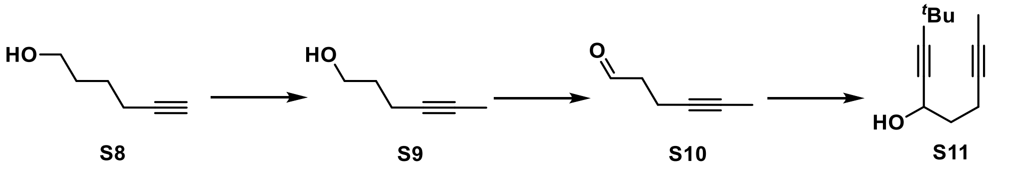

Hex-5-yn-1-ol **S8** (2.7 g, 3 mL, 27.5 mmol, 1.0 eq) was dissolved in DMSO (96 mL, 0.3 M), followed by the addition of KOt-Bu (6.17 g, 55 mmol, 2.0 eq.). The mixture was stirred at room temperature for 3 h before being quenched with 2 M HCl and diluted with water at 0 °C (the reaction color changes from dark-brown to yellow). The resulting mixture was extracted three times with ethyl acetate, and the combined organic layers were dried over Na_2SO_4, filtered, and concentrated *in vacuo*. Silica gel column chromatography (*n*-pentane:EtOAc = 5:1) afforded the desired product **S9** (1.86 g, 18.95 mmol, 69%) as colorless oil. The analytical data were consistent with literature.[8]

Hex-4-yn-1-ol **S9** (1.50 g, 15.28 mmol, 1.0 eq.) was dissolved in CH_2Cl_2 (24 mL, 0.6 M). The resulting solution was slowly added to pyridinium dichromate (11.50 g, 30.57 mmol, 2.0 eq) dissolved in CH_2Cl_2 (52 mL, 0.3 M). After stirring for 5 h, the residue was filtered through a small silica column. The solvent was removed *in vacuo*, and the crude product was purified by silica gel column chromatography (*n*-pentane:Et_2O = 5:1) to furnish hex-4-ynal **S10** (1.0 g, 10.4 mmol, 68%) as colorless liquid. The analytical data were consistent with literature.[9]

Hex-4-ynal **S10** (1.0 g, 10.4 mmol, 1.0 eq.) in THF (36.5 mL, 0.3 M), 3,3-dimethylbut-1-yne (1.71 g, 2.48 mL, 20.81 mmol, 2.0 eq.) in THF (52.0 mL, 0.4 M), and *n*-BuLi (2.5 M in hexane, 6.2 mL, 15.60 mmol, 1.5 eq.) were reacted according to **GP1**. The reaction mixture was stirred at r.t. for 17 h. Silica gel column chromatography (*n*-pentane:EtOAc = 10:1) afforded dialkynes unit **S11** (0.8 g, 4.49 mmol, 43%) as colorless oil.

$\mathbf{R_f}$ = 0.29 (*n*-pentane:EtOAc = 10:1).

^{1}H-NMR (500 MHz, $CDCl_3$): δ = 4.48 (d, J = 7.0 Hz, 1H), 2.38 – 2.21 (m, 2H), 1.86 – 1.81 (m, 2H), 1.77 (t, J = 2.6 Hz, 3H), 1.21 (s, 10H).

^{13}C-NMR (126 MHz, $CDCl_3$): δ = 94.4, 79.1, 78.4, 76.4, 61.9, 37.4, 31.1, 27.5, 15.0, 3.6.

IR (ATR): $\tilde{\nu}$ (cm^{-1}) = 3358, 2967, 2924, 2866, 2235, 1476, 1443, 1362, 1263, 1204, 1061, 1013.

HRMS (APCI, Q-TOF): calculated for $C_{12}H_{18}NO_2^+$ $[M+NO]^+$: 208.1332, found: 208.1332.

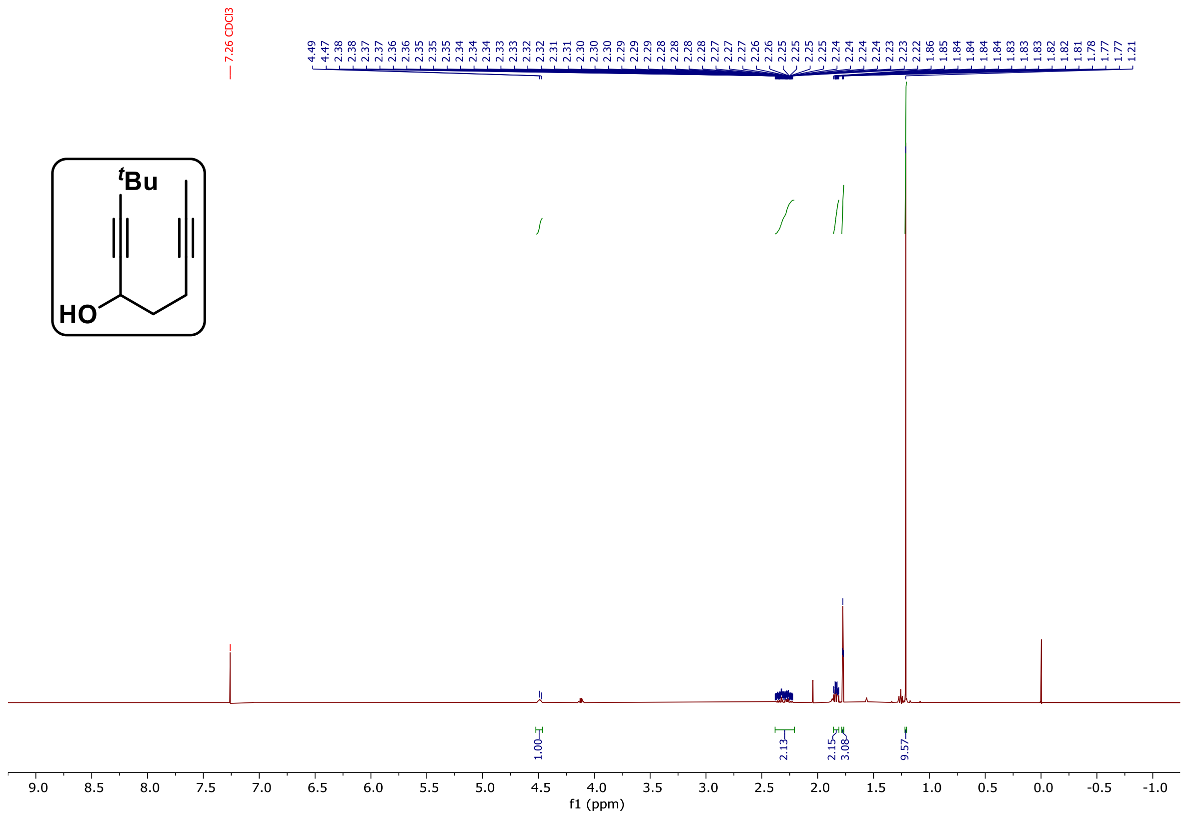

¹H-NMR (500 MHz, CDCl₃)

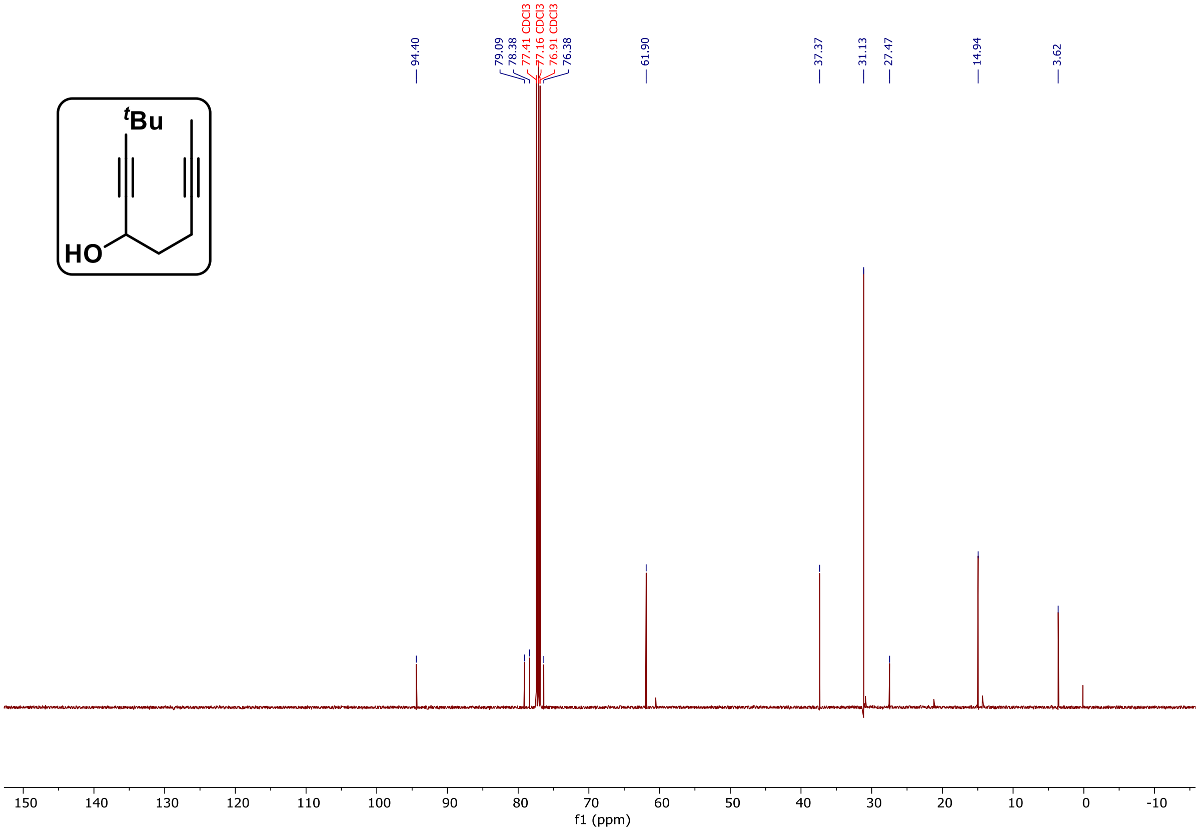

¹³C-NMR (126 MHz, CDCl₃)

1-((2,2-dimethyldeca-3,8-diyn-5-yl)oxy)-2-iodobenzene (1e)

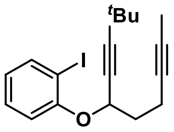

Alcohol **S11** (340 mg, 1.91 mmol, 1.0 eq.) in THF (19 mL, 0.1 M), 2-idodophenol (503.5 mg, 2.29 mmol, 1.2 eq.), PPh_3 (750.4 mg, 2.86 mmol, 1.5 eq.), and DIAD (655.6 mg, 637 µL, 3.24 mmol, 1.7 eq.) were reacted according to **GP3**. The reaction mixture was stirred at r.t. for 21 h. Silica gel column chromatography (*n*-pentane:EtOAc = 40:1) afforded the domino precursor **1e** (200 mg, 0.53 mmol, 69%) as yellow oil.

R_f = 0.5 (*n*-pentane:EtOAc = 40:1).

^{1}H-NMR (500 MHz, $CDCl_3$): δ = 7.76 (dd, *J* = 7.8, 1.6 Hz, 1H), 7.28 (ddd, *J* = 8.2, 7.3, 1.6 Hz, 1H), 7.11 (dd, *J* = 8.2, 1.4 Hz, 1H), 6.74 – 6.70 (m, 1H), 4.83 (dd, *J* = 7.2, 5.3 Hz, 1H), 2.55 – 2.40 (m, 2H), 2.22 – 2.15 (m, 1H), 2.11 – 2.04 (m, 1H), 1.77 (t, *J* = 2.5 Hz, 3H), 1.17 (s, 9H).

^{13}C-NMR (126 MHz, $CDCl_3$): δ = 156.8, 139.3, 129.1, 123.0, 115.0, 96.7, 87.6, 78.2, 76.2, 76.0, 68.8, 35.8, 30.8, 27.5, 15.2, 3.6.

IR (ATR): $\tilde{\nu}$ (cm^{-1}) = 2967, 2917, 1580, 1570, 1469, 1439, 1362, 1339, 1275, 1263, 1237, 1203, 1119, 1056, 1018, 1000.

HRMS (APCI, Q-TOF): calculated for $C_{18}H_{22}IO^+$ [M+H]$^+$: 381.0710, found: 381.0704.

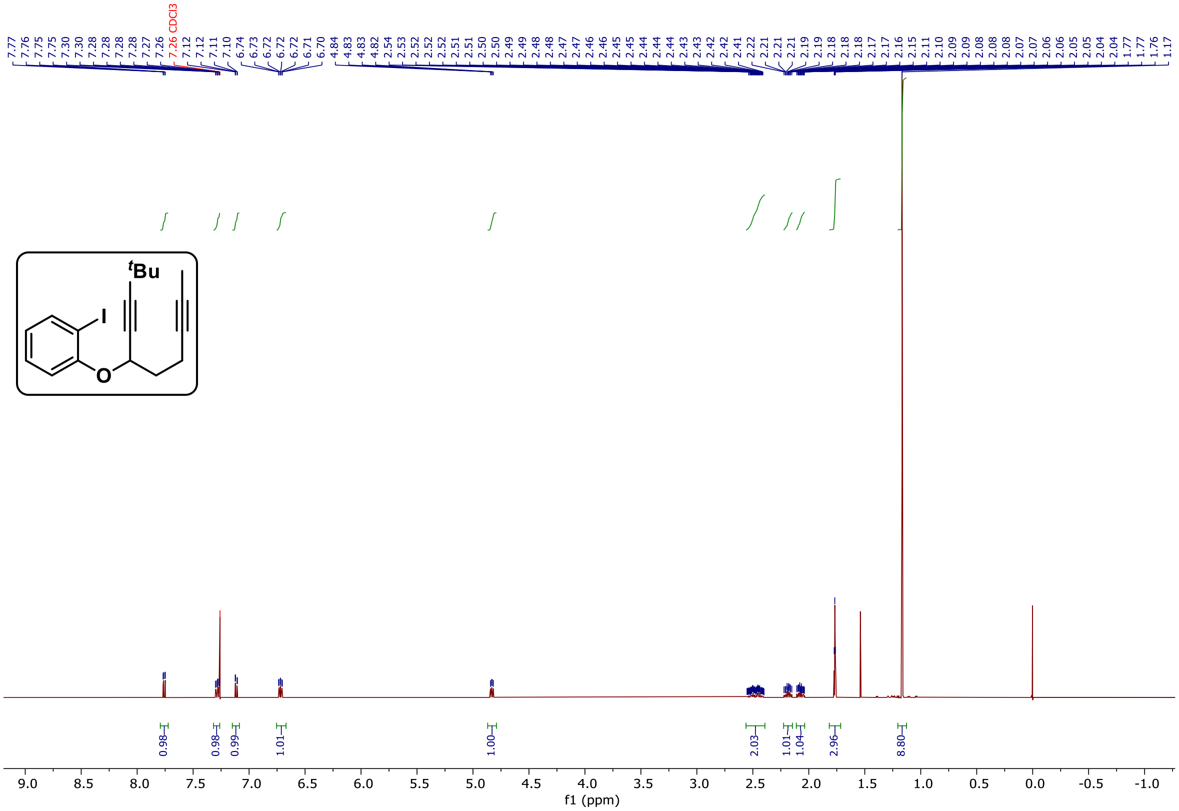

^{1}H-NMR (500 MHz, $CDCl_3$)

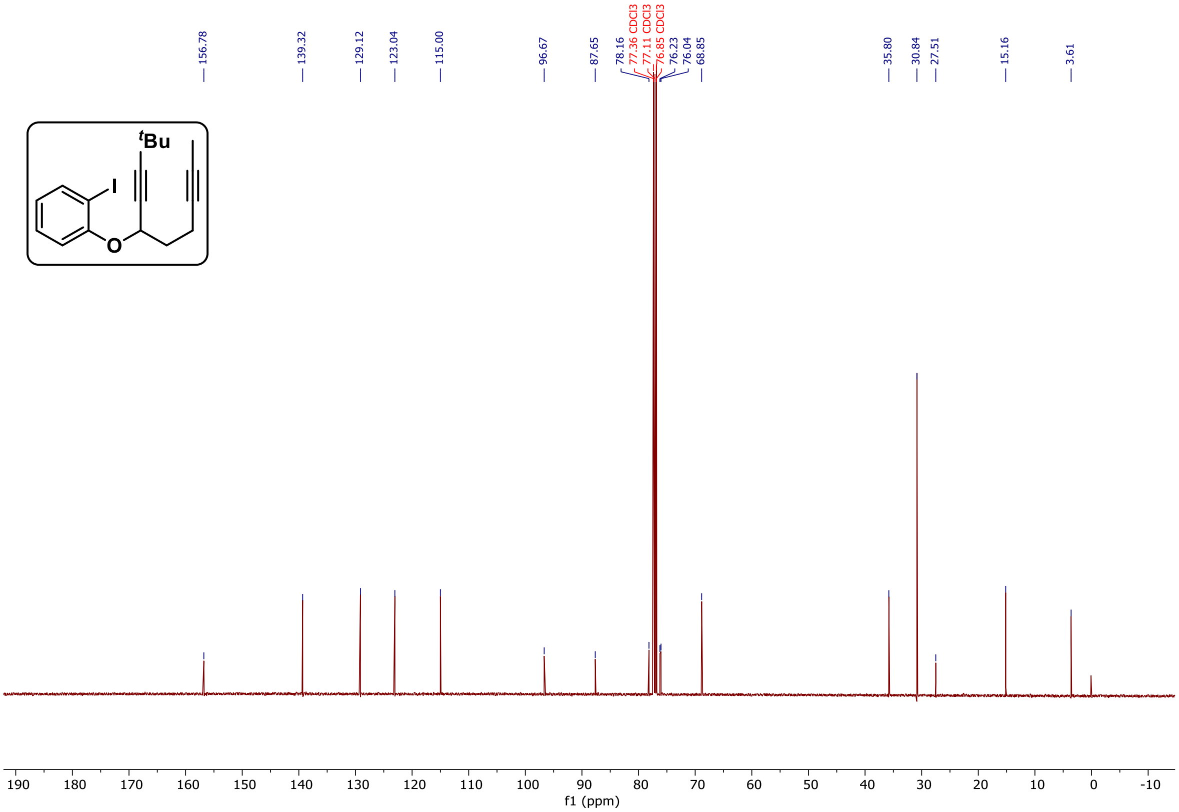

^{13}C-NMR (126 MHz, $CDCl_3$)

4-(*tert*-butyl)-1-((2,2-dimethyldeca-3,8-diyn-5-yl)oxy)-2-iodobenzene (1f)

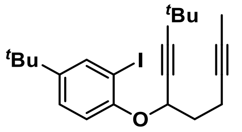

Alcohol **S11** (213.9 mg, 1.20 mmol, 1.0 eq.) in THF (12 mL, 0.1 M), 4-(*tert*-Butyl)-2-iodophenol (397.6 mg, 1.44 mmol, 1.2 eq.), PPh_3 (472.1 mg, 1.80 mmol, 1.5 eq), and DIAD (412.5 mg, 400 µL, 2.04 mmol, 1.7 eq.) were reacted according to **GP3**. The reaction mixture was stirred at r.t. for 16 h. Silica gel column chromatography (*n*-pentane:EtOAc = 100:1) afforded the domino precursor **1f** (244 mg, 0.56 mmol, 47%) as yellow oil.

$\mathbf{R_f}$ = 0.27 (*n*-pentane:EtOAc = 100:1).

^{1}H-NMR (500 MHz, $CDCl_3$): δ = 7.75 (d, *J* = 2.4 Hz, 1H), 7.29 (dd, *J* = 8.6, 2.4 Hz, 1H), 7.04 (d, *J* = 8.6 Hz, 1H), 4.78 (dd, *J* = 7.2, 5.4 Hz, 1H), 2.55 – 2.37 (m, 2H), 2.23 – 2.11 (m, 1H), 2.11 – 2.00 (m, 1H), 1.77 (t, *J* = 2.5 Hz, 3H), 1.29 (s, 9H), 1.17 (s, 9H).

^{13}C-NMR (126 MHz, $CDCl_3$): δ = 154.7, 146.2, 136.3, 126.1, 114.6, 96.5, 87.6, 78.3, 76.3, 76.2, 69.1, 35.9, 34.2, 31.5, 30.9, 15.2, 3.6.

IR (ATR): $\tilde{\nu}$ (cm^{-1}) = 2960, 1491, 1479, 1446, 1361, 1343, 1281, 1257, 1242, 1203, 1190, 1061, 1030, 1000, 979, 917.

HRMS (APCI, Q-TOF): calculated for $C_{22}H_{30}IO^+$ $[M+H]^+$: 437.1336, found: 437.1339.

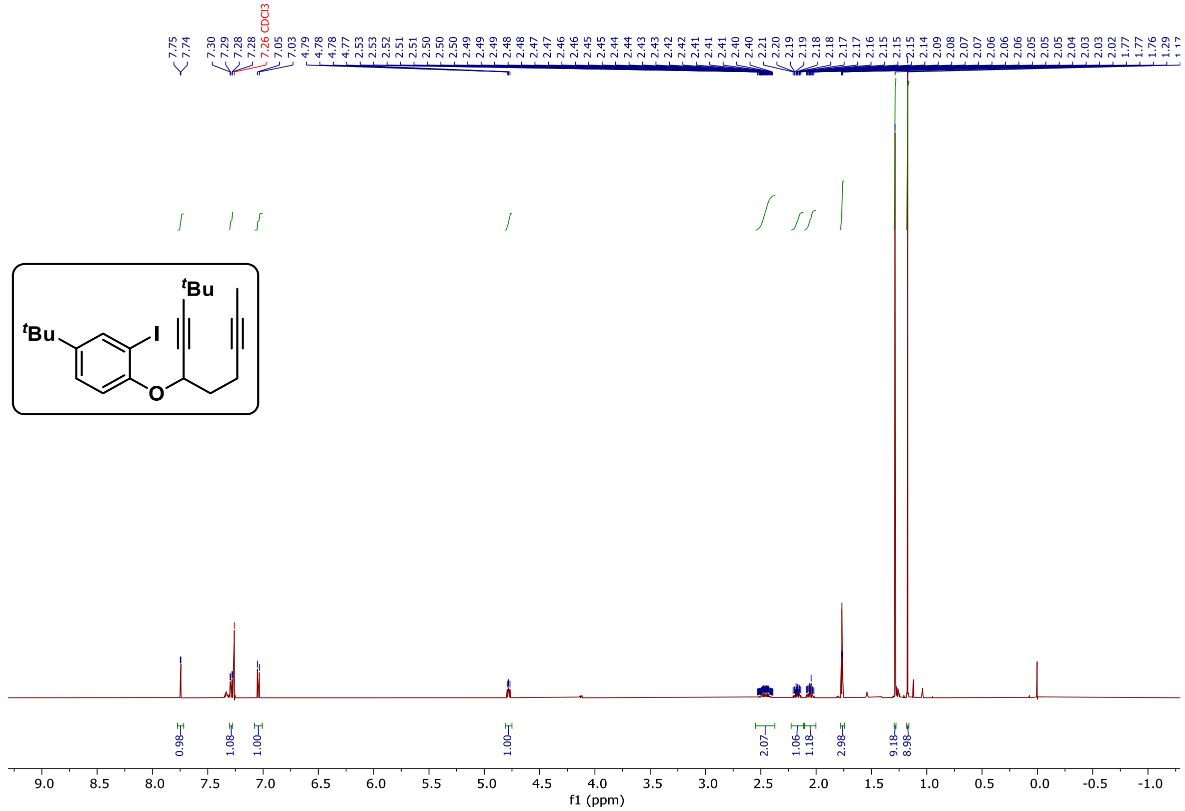

^{1}H-NMR (500 MHz, $CDCl_3$)

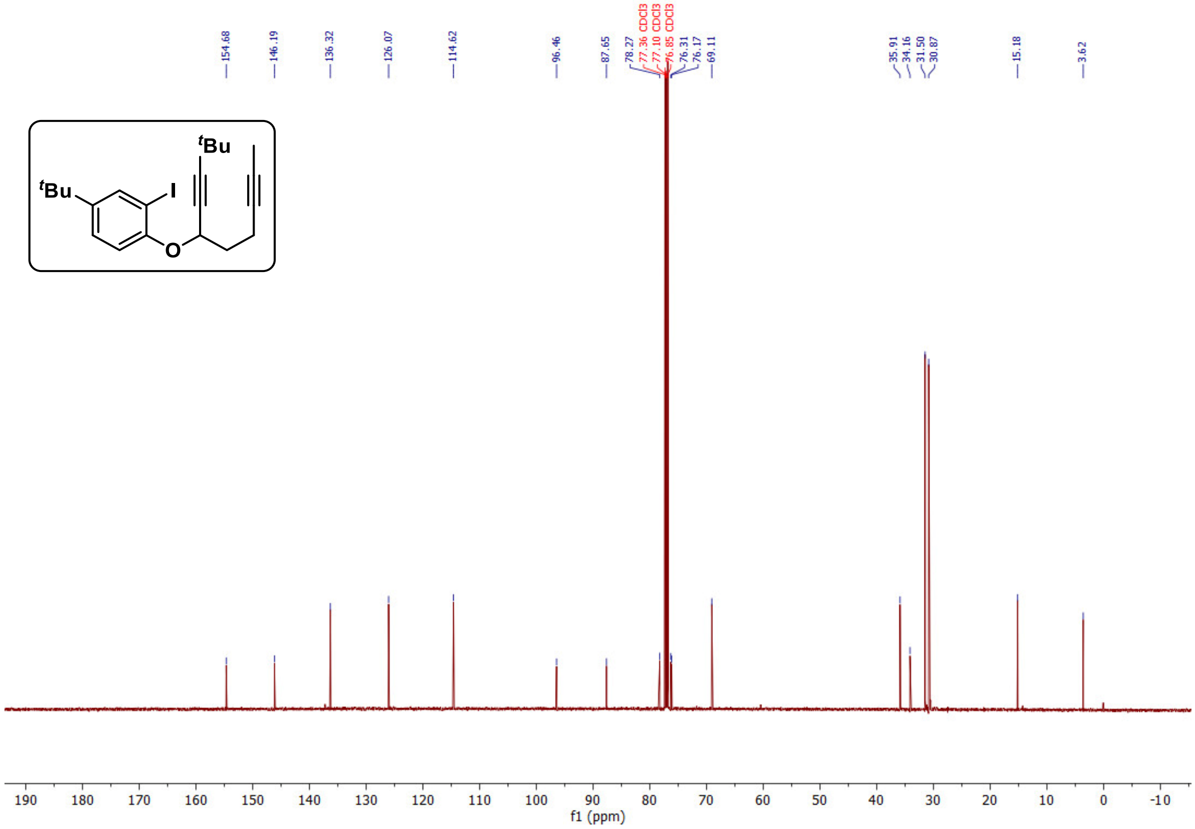

^{13}C-NMR (126 MHz, $CDCl_3$)

1-((2,2-dimethyldeca-3,8-diyn-5-yl)oxy)-2-iodo-4-nitrobenzene (1g)

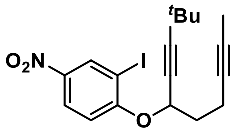

Alcohol **S11** (241.0 mg, 1.35 mmol, 1.0 eq.) in THF (13.5 mL, 0.1 M), 2-iodo-4-nitrophenol (446.0 mg, 1.68 mmol, 1.2 eq.), PPh_3 (551.7 mg, 2.10 mmol, 1.6 eq.), and DIAD (482.1 mg, 468 µL, 2.38 mmol, 1.8 eq.) were reacted according to **GP3**. The reaction mixture was stirred at r.t. for 7 h. Silica gel column chromatography (*n*-pentane:EtOAc = 60:1) afforded the domino precursor **1g** (360 mg, 0.85 mmol, 63%) as yellow oil.

$\mathbf{R_f}$ = 0.24 (*n*-pentane:EtOAc = 60:1).

^{1}**H-NMR** (400 MHz, $CDCl_3$): δ = 8.66 (d, *J* = 2.7 Hz, 1H), 8.23 (dd, *J* = 9.1, 2.7 Hz, 1H), 7.15 (d, *J* = 9.3 Hz, 1H), 4.96 (dd, *J* = 7.4, 5.2 Hz, 1H), 2.55 – 2.38 (m, 2H), 2.28 – 2.17 (m, 1H), 2.11 (dtd, *J* = 13.7, 7.6, 5.2 Hz, 1H), 1.76 (t, *J* = 2.6 Hz, 3H), 1.18 (s, 9H).

^{13}**C-NMR** (101 MHz, $CDCl_3$): δ = 161.7, 142.2, 135.1, 125.3, 112.7, 98.1, 86.2, 77.5, 76.8, 74.7, 69.5, 35.5, 30.8, 27.7, 15.1, 3.6.

IR (ATR): $\tilde{\nu}$ (cm^{-1}) = 2968, 1576, 1516, 1470, 1363, 1338, 1260, 1247, 1203, 1145, 1115, 1055, 1029.

HRMS (APCI, Q-TOF): calculated for $C_{18}H_{21}INO_3^+$ $[M+H]^+$: 426.0561, found: 426.0559.

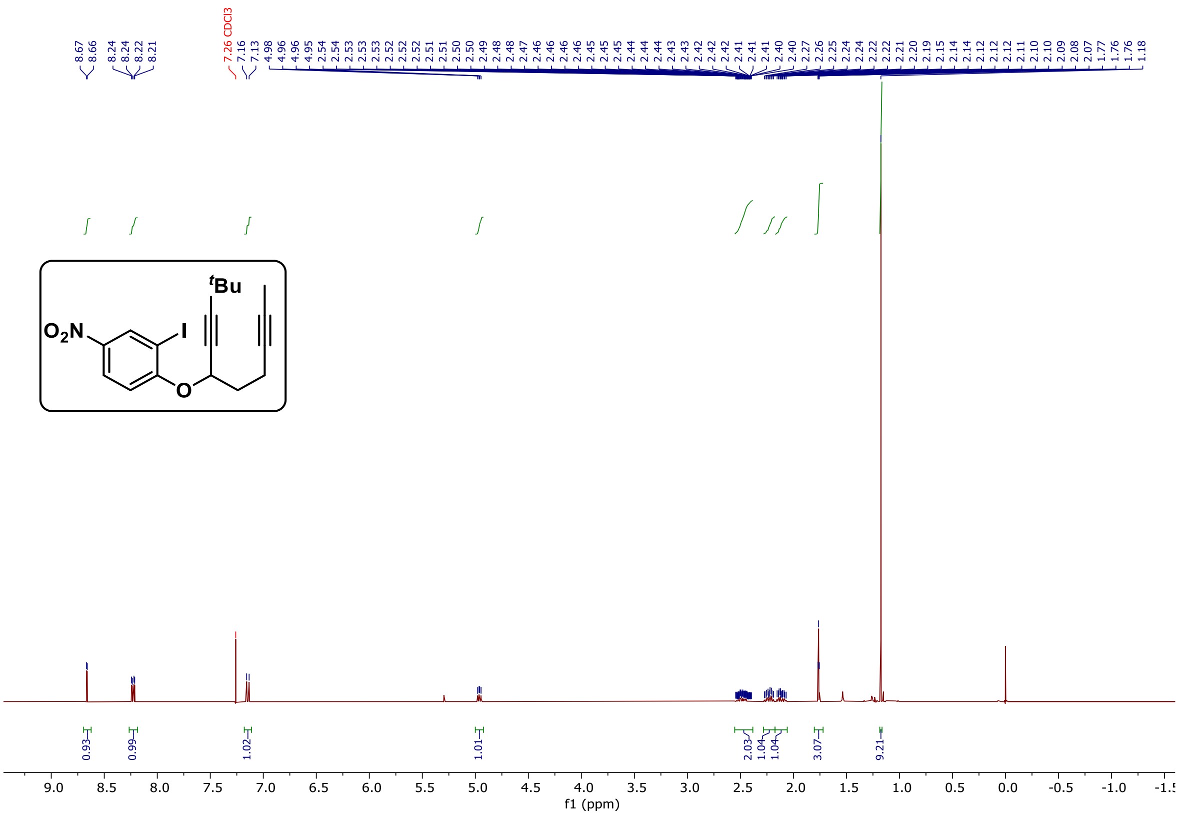

^{1}H-NMR (400 MHz, $CDCl_3$)

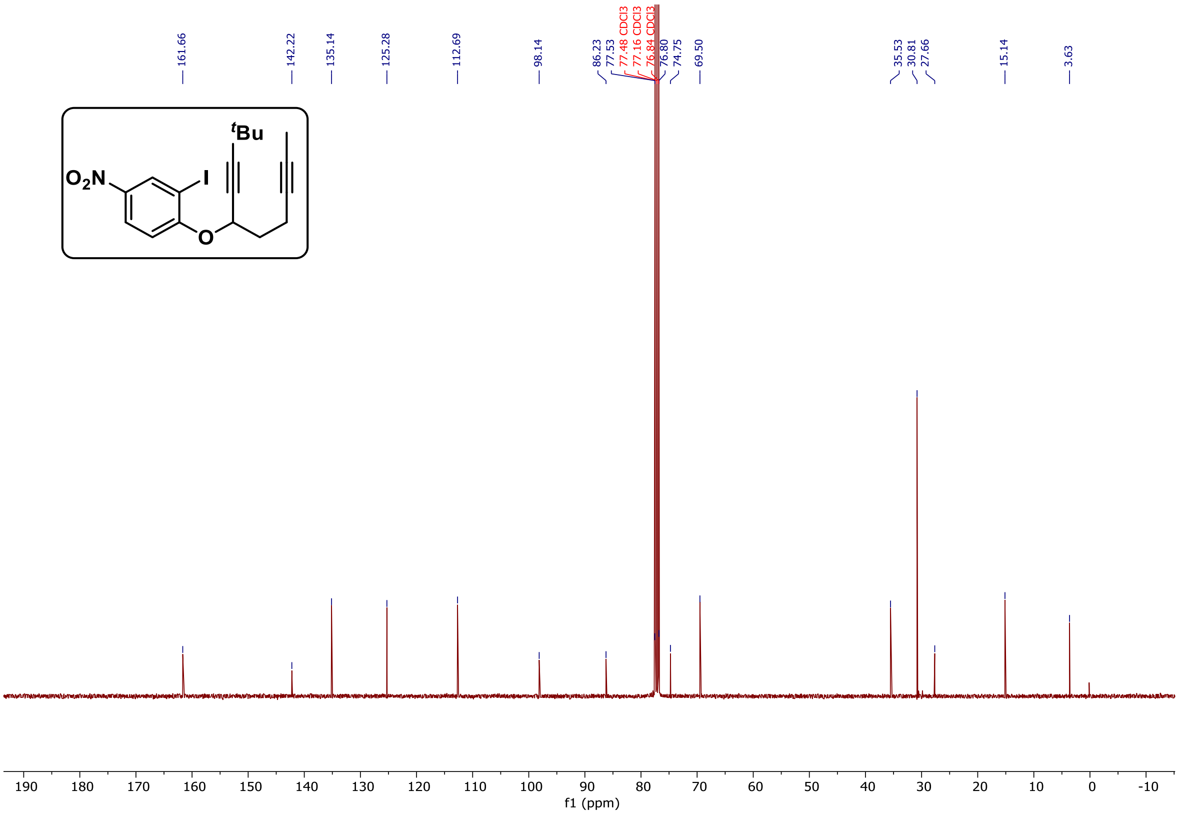

^{13}C-NMR (101 MHz, $CDCl_3$)

1-((2,2-dimethyldeca-3,8-diyn-5-yl)oxy)-2-iodo-4-(trifluoromethyl)benzene (1h)

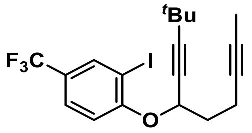

Alcohol **S11** (250 mg, 1.40 mmol, 1.0 eq.) in THF (14 mL, 0.1 M), 2-iodo-4-(trifluoromethyl)phenol (484.8 mg, 1.68 mmol, 1.2 eq.), PPh_3 (551 mg, 2.10 mmol, 1.5 eq.), and DIAD (482 mg, 470 µL, 2.38 mmol, 1.7 eq.) were reacted according to **GP3**. The solution was allowed to stir at r.t. for 19 h. Silica gel column chromatography (*n*-pentane:EtOAc = 100:1) afforded the domino precursor **1h** (357 mg, 0.80 mmol, 57%) as a yellow oil.

$\mathbf{R_f}$ = 0.45 (*n*-pentane:EtOAc = 100:1).

^{1}**H-NMR** (500 MHz, $CDCl_3$): δ = 8.01 – 8.00 (dd, *J* = 2.3, 0.8 Hz, 1H), 7.57 – 7.55 (ddd, *J* = 8.7, 2.3, 0.8 Hz, 1H), 7.15 – 7.14 (d, *J* = 8.0 Hz, 1H), 4.90 – 4.88 (dd, *J* = 7.4, 5.2 Hz, 1H), 2.55 – 2.40 (m, 2H), 2.25 – 2.04 (m, 2H), 1.76 (t, *J* = 2.5 Hz, 3H), 1.17 (s, 9H).

^{13}**C-NMR** (126 MHz, $CDCl_3$): δ = 3.6, 15.2, 27.6, 30.8, 35.7, 69.0, 75.3, 76.6, 77.8, 86.9, 97.5, 113.6, 123.5 (q, *J* = 271.9 Hz), 124.9 (q, *J* = 33.2 Hz), 126.6 (q, *J* = 3.8 Hz), 136.5 (q, *J* = 3.8 Hz), 159.2.

^{19}**F-NMR** (471 MHz, $CDCl_3$): δ = -61.6.

IR (ATR): $\tilde{\nu}$ (cm^{-1}) = 2969, 2924, 2868, 1574, 1476, 1455, 1444, 1400, 1363, 1203.

HRMS (APCI, Q-TOF): calculated for $C_{19}H_{21}F_3IO^+$ $[M+H]^+$: 449.0584, found: 449.0583.

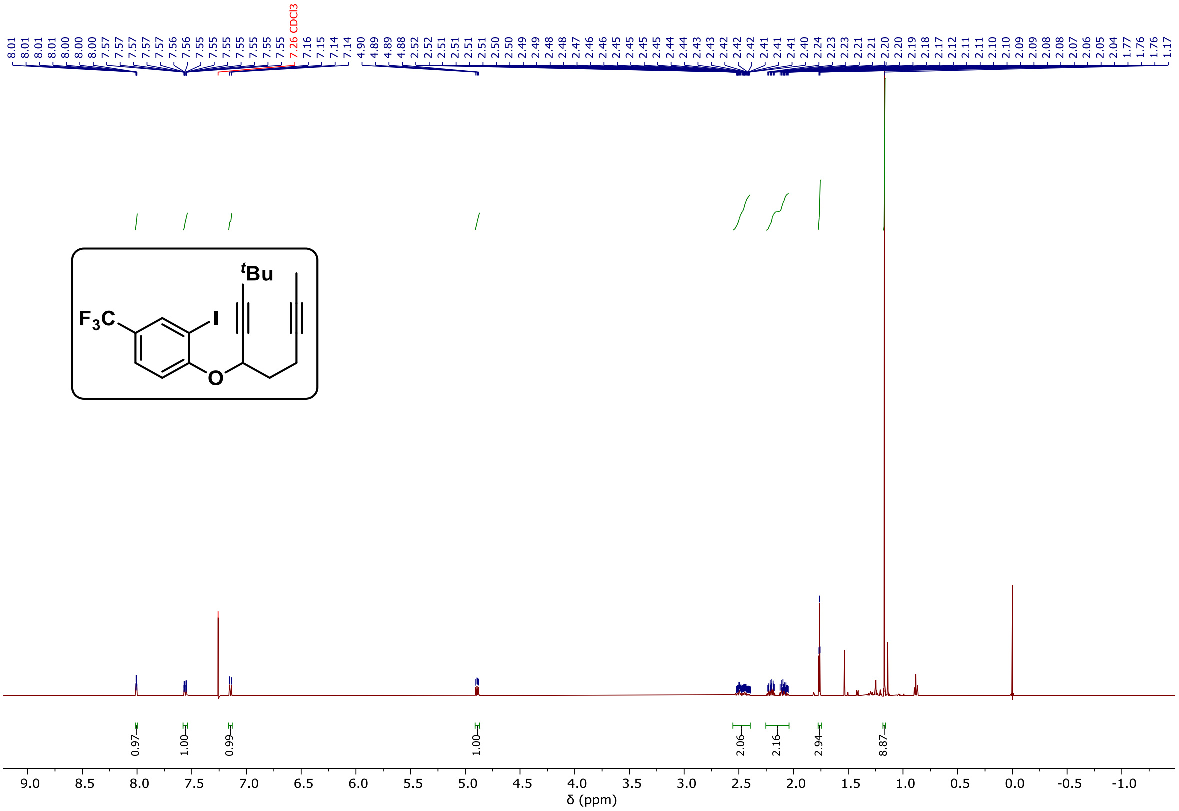

^{1}H-NMR (500 MHz, $CDCl_3$)

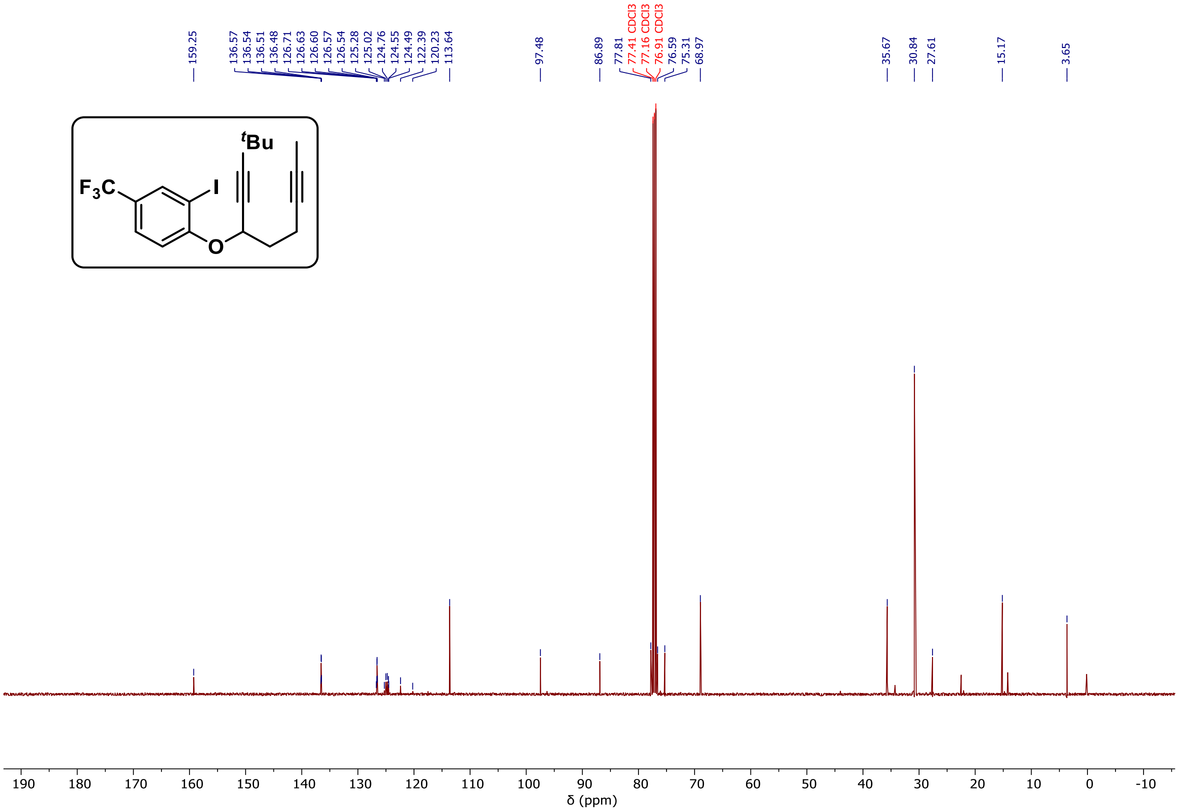

^{13}C-NMR (126 MHz, $CDCl_3$)

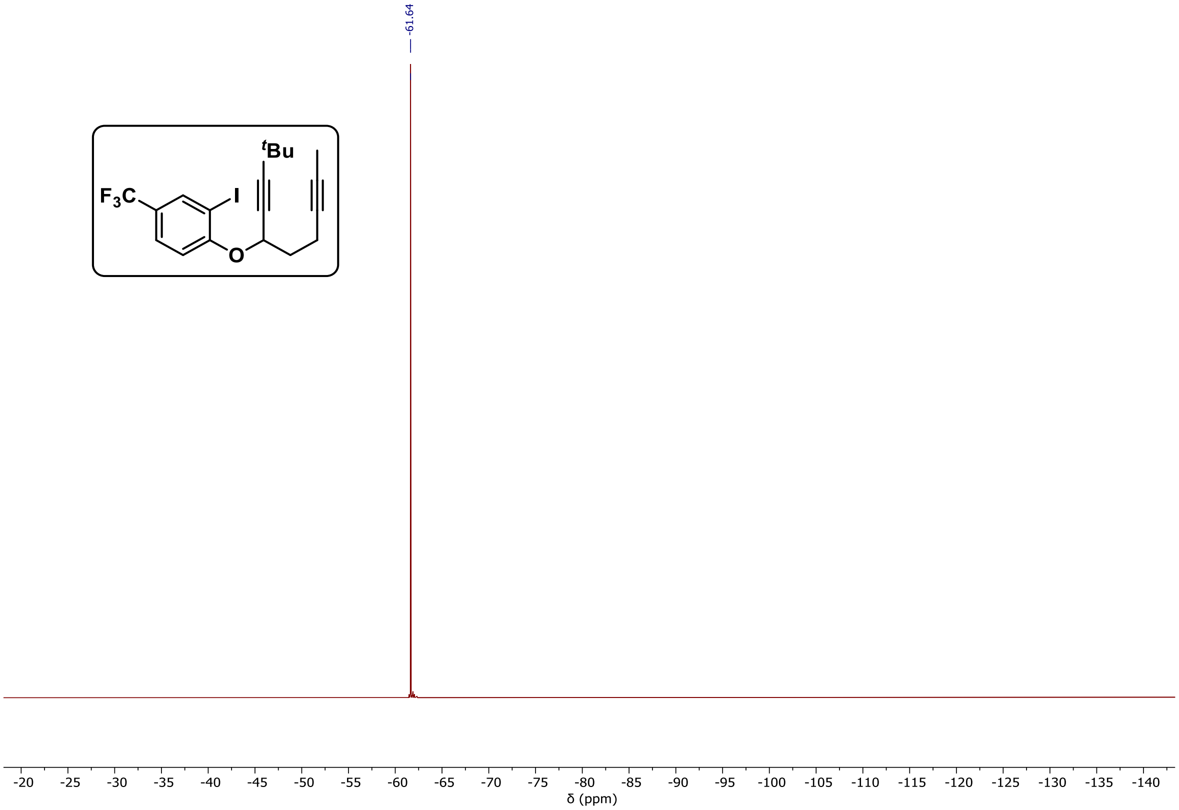

^{19}F-NMR (471 MHz, $CDCl_3$)

2-((2,2-dimethyldeca-3,8-diyn-5-yl)oxy)-1-iodo-4-methoxybenzene (1i)

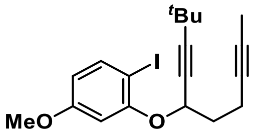

Alcohol **S11** (150 mg, 0.84 mmol, 1.0 eq.) in THF (8.5 mL, 0.1 M), 2-iodo-5-methoxyphenol (252 mg, 1.01 mmol, 1.2 eq.), PPh_3 (331 mg, 1.26 mmol, 1.5 eq.), and DIAD (289 mg, 280 µL, 1.43 mmol, 1.7 eq.) were reacted according to **GP3**. The solution was allowed to stir at r.t. for 15 h. Silica gel column chromatography (*n*-pentane:EtOAc = 100:1) afforded the domino precursor **1i** (141 mg, 0.34 mmol, 41%) as a yellow oil.

$\mathbf{R_f}$ = 0.37 (*n*-pentane:EtOAc = 100:1).

^{1}H-NMR (400 MHz, $CDCl_3$): δ = 7.61 – 7.58 (d, J = 8.7 Hz, 1H), 6.78 – 6.77 (d, J = 2.7 Hz, 1H), 6.37 – 6.34 (dd, J = 8.7, 2.7 Hz, 1H), 4.81 – 4.78 (dd, J = 7.2, 5.4 Hz, 1H), 3.80 (s, 3H), 2.49 – 2.45 (m, 2H), 2.21 – 2.04 (m, 2H), 1.78 – 1.76 (t, J = 2.6 Hz, 3H), 1.18 (s, 9H).

^{13}C-NMR (101 MHz, $CDCl_3$): δ = 3.7, 15.2, 27.6, 30.9, 35.9, 55.6, 69.0, 76.1, 76.3, 77.4, 78.2, 96.8, 102.3, 108.8, 139.0, 157.7, 161.2.

IR (ATR): $\tilde{\nu}$ (cm^{-1}) = 2965, 2925, 2861, 1580, 1473, 1442, 1419, 1405, 1379, 1363, 1338, 1299, 1277, 1260, 1201, 1166, 1132, 1117, 1057, 1030, 1014, 981.

HRMS (APCI, Q-TOF): calculated for $C_{19}H_{24}IO_2^+$ $[M+H]^+$: 411.0815, found: 411.0817.

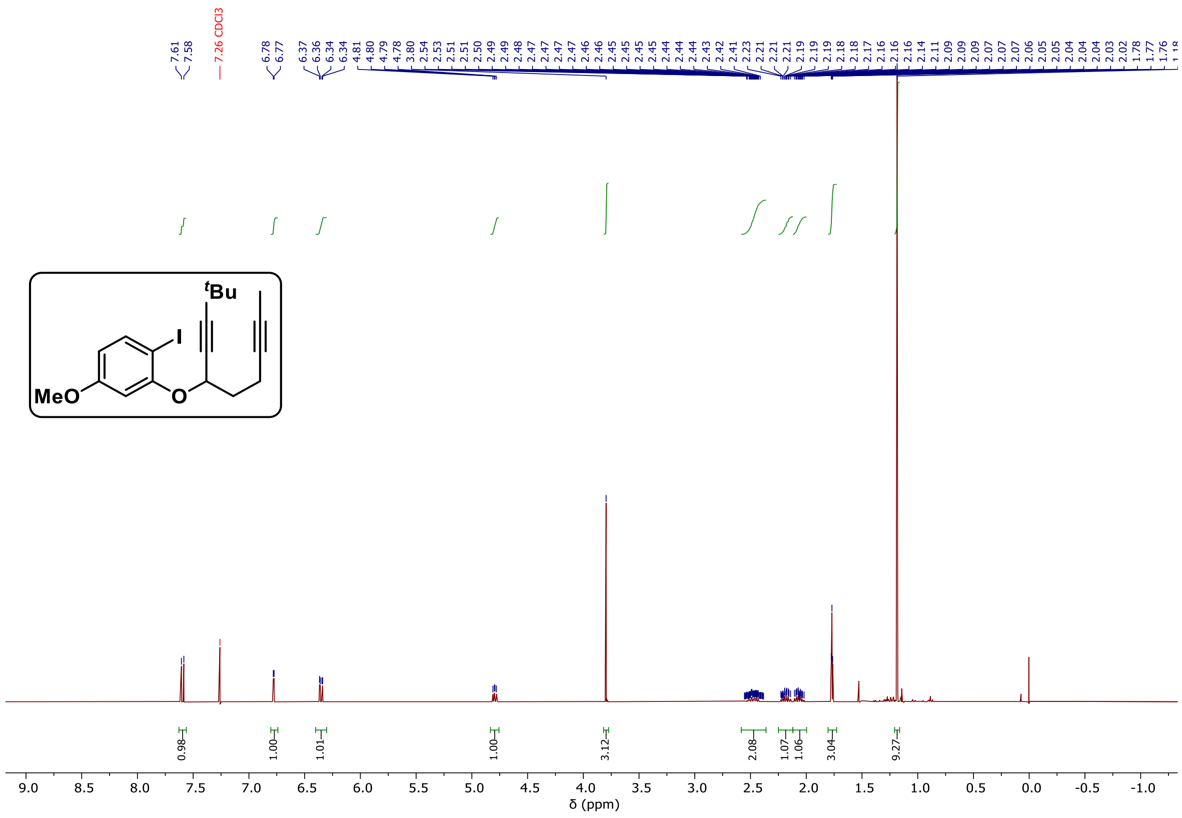

^{1}H-NMR (400 MHz, $CDCl_3$)

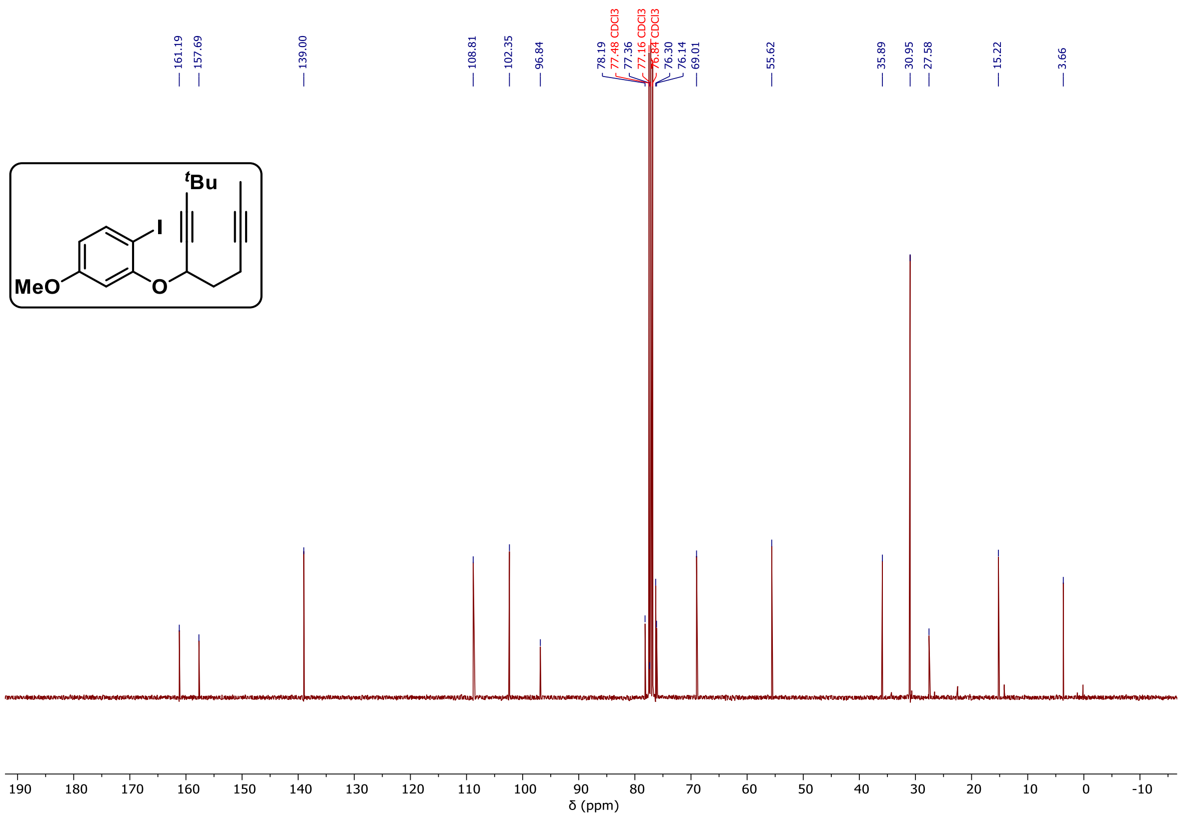

^{13}C-NMR (101 MHz, $CDCl_3$)

(3-((2,2-dimethyldeca-3,8-diyn-5-yl)oxy)-4-iodobenzonitrile (1j)

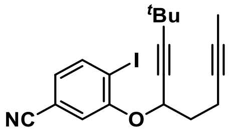

Alcohol **S11** (214 mg, 1.20 mmol, 1.0 eq.) in THF (12 mL, 0.1 M), 3-hydroxy-4-iodobenzonitrile (353 mg, 1.44 mmol, 1.2 eq.), PPh_3 (472 mg, 1.80 mmol, 1.5 eq.), and DIAD (412 mg, 400 µL, 2.04 mmol, 1.7 eq.) were reacted according to **GP3**. The solution was allowed to at r.t. for 23 h. Silica gel column chromatography (*n*-pentane:EtOAc = 80:1) afforded the domino precursor **1j** (258 mg, 0.64 mmol, 53%) as a highly viscous colorless oil.

$\mathbf{R_f}$ = 0.38 (*n*-pentane:EtOAc = 80:1).

^{1}H-NMR (700 MHz, $CDCl_3$): δ = 7.88 – 7.87 (d, *J* = 8.0 Hz, 1H), 7.34 (d, *J* = 1.8 Hz, 1H), 7.00 – 6.99 (dd, *J* = 8.0, 1.7 Hz, 1H), 4.89 – 4.87 (dd, *J* = 7.4, 5.2 Hz, 1H), 2.52 – 2.48 (dddt, *J* = 16.8, 7.5, 5.0, 2.6 Hz, 1H), 2.46 – 2.40 (m, 1H), 2.22 – 2.18 (dtd, *J* = 13.4, 7.4, 5.9 Hz, 1H), 2.11 – 2.06 (m, 1H), 1.78 – 1.77 (t, *J* = 2.5 Hz, 3H), 1.18 (s, 9H).

^{13}C-NMR (176 MHz, $CDCl_3$): δ = 3.7, 15.1, 27.6, 30.8, 35.5, 69.3, 74.9, 76.8, 77.6, 94.3, 98.2, 112.8, 117.2, 118.5, 126.0, 140.4, 157.0.

IR (ATR): $\tilde{\nu}$ (cm^{-1}) = 3082, 2968, 2927, 2867, 2231, 1721, 1624, 1581, 1558, 1468, 1445, 1400, 1363, 1339, 1277, 1253, 1203, 1162, 1135,1119, 1055, 1018, 1004.

HRMS (APCI, Q-TOF): calculated for $C_{19}H_{19}INO^-$ $[M-H]^-$:404.0517, found: 404.0522.

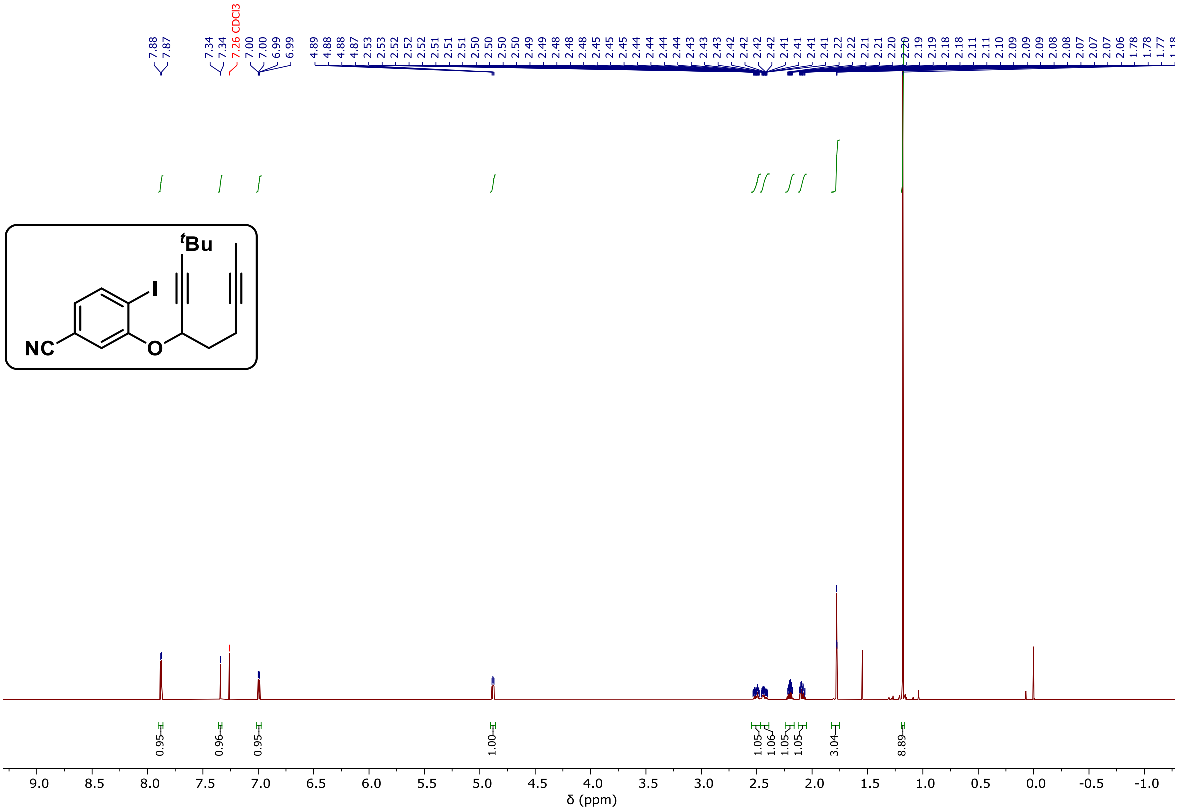

^{1}H-NMR (700 MHz, $CDCl_3$)

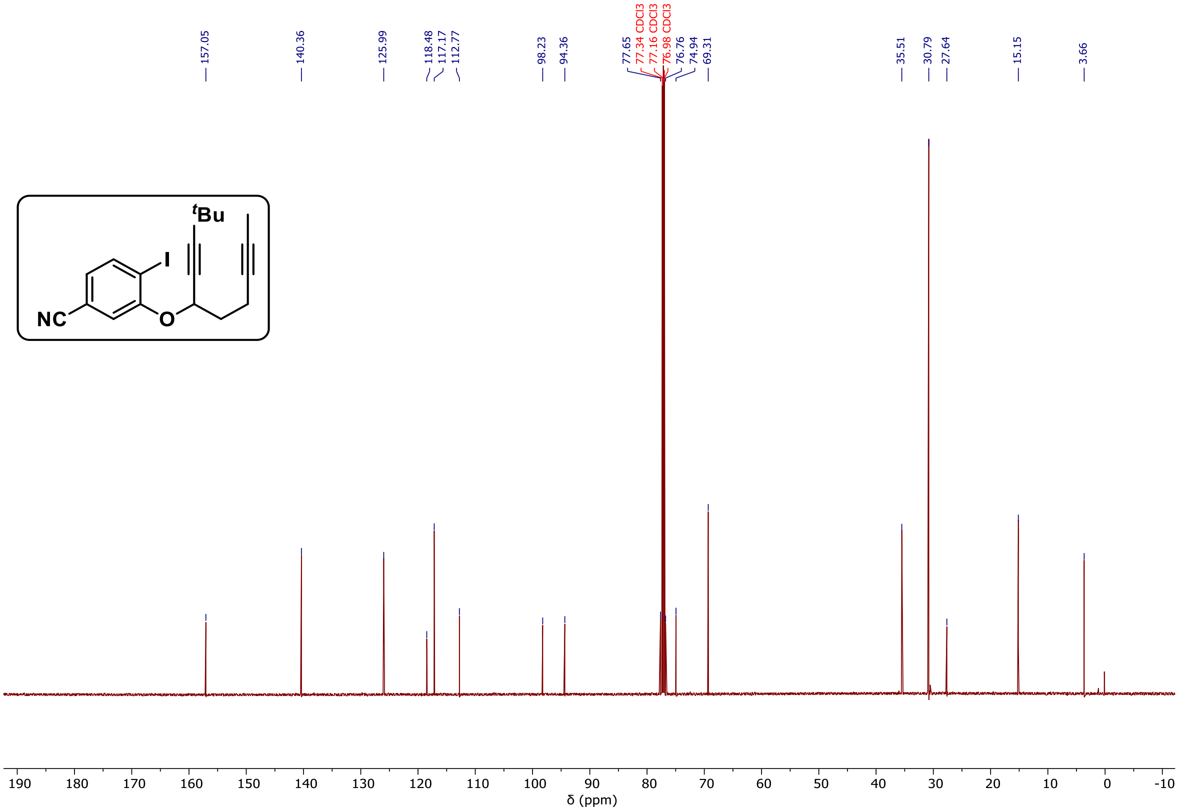

^{13}C-NMR (176 MHz, $CDCl_3$)

3-((2,2-dimethyldeca-3,8-diyn-5-yl)oxy)-4-iodobenzaldehyde (1k)

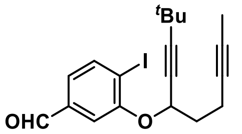

Alcohol **S11** (128.5 mg, 0.72 mmol, 1.0 eq.) in THF (7.5 mL, 0.1 M), 3-hydroxy-4-iodobenzaldehyde (214.3 mg, 0.86 mmol, 1.2 eq.), PPh_3 (283.5 mg, 1.09 mmol, 1.5 eq.), and DIAD (247.5 mg, 240 µL, 1.22 mmol, 1.7 eq.) were reacted according to **GP3**. The solution was allowed to stir at r.t. for 18.5 h. Silica gel column chromatography (*n*-pentane:EtOAc = 80:1) afforded the domino precursor **1k** (130.5 mg, 0.32 mmol, 44%) as a yellow oil.

$\mathbf{R_f}$ = 0.31 (*n*-pentane:EtOAc = 80:1).

^{1}**H-NMR** (700 MHz, $CDCl_3$): δ = 9.94 (s, 1H), 7.96 (d, *J* = 7.9 Hz, 1H), 7.61 (d, *J* = 1.8 Hz, 1H), 7.19 (dd, *J* = 7.9, 1.7 Hz, 1H), 4.94 (dd, *J* = 7.3, 5.2 Hz, 1H), 2.51 (dddt, *J* = 16.8, 7.6, 5.1, 2.6 Hz, 1H), 2.47 – 2.39 (m, 1H), 2.20 (dtd, *J* = 13.5, 7.5, 5.9 Hz, 1H), 2.13 – 2.05 (m, 1H), 1.76 (t, *J* = 2.5 Hz, 3H), 1.15 (s, 9H).

^{13}**C-NMR** (176 MHz, CDCl3): δ = 191.4, 191.4, 157.4, 140.1, 137.6, 124.7, 113.3, 97.8, 96.6, 77.9, 76.6, 75.4, 69.0, 35.5, 30.8, 27.6, 15.2, 3.6.

IR (ATR): $\tilde{\nu}$ (cm^{-1}) = 2967, 1597, 1575, 1468, 1417, 1384, 1364, 1338, 1297, 1263, 1243, 1203, 1160, 1057, 1018.

HRMS (APCI, Q-TOF): calculated for $C_{19}H_{22}IO_2^+$ $[M+H]^+$: 409.0659, found: 409.0660.

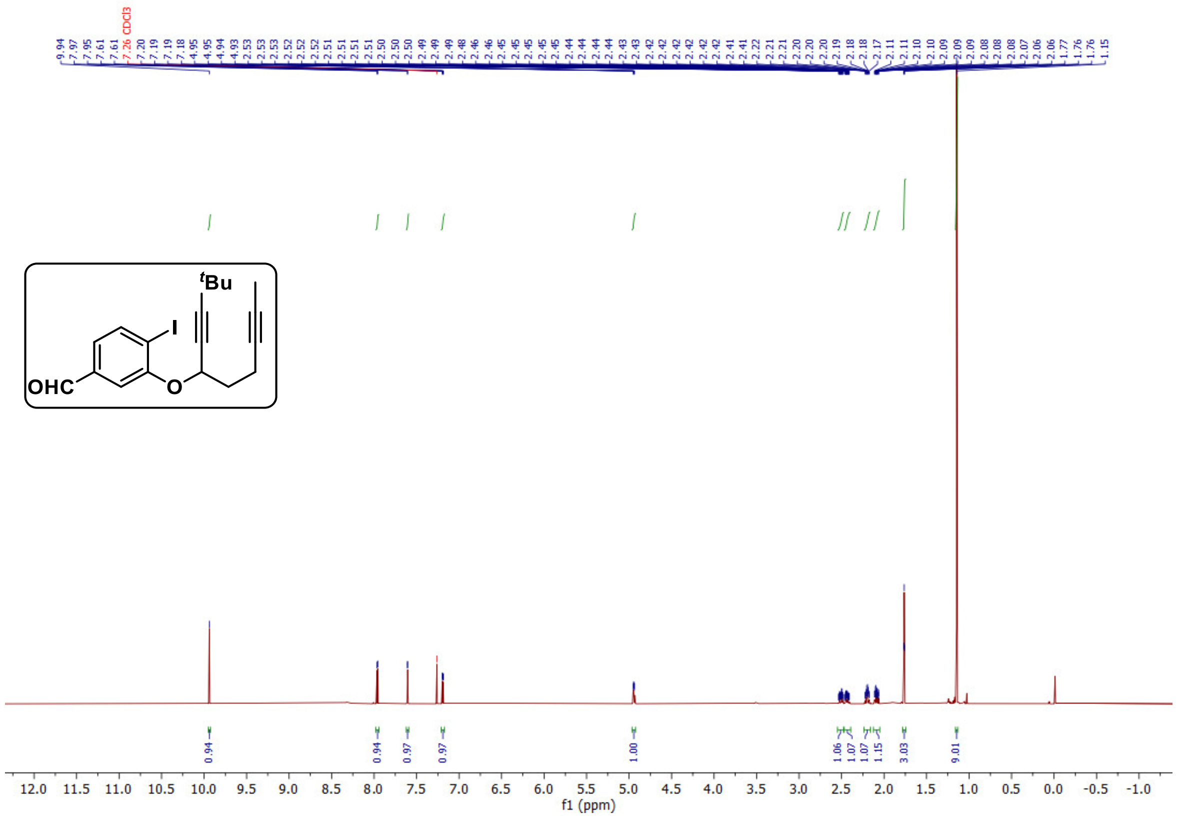

^{1}H-NMR (700 MHz, $CDCl_3$)

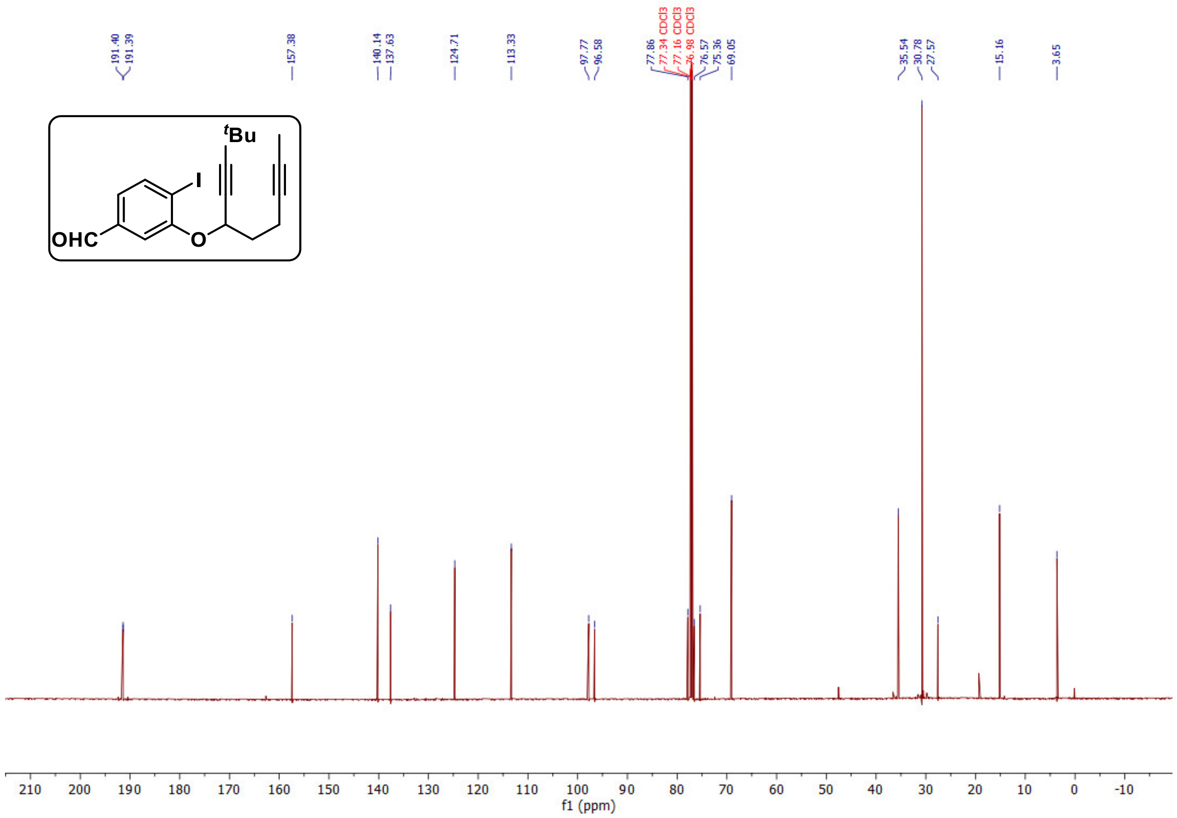

^{13}C-NMR (176 MHz, $CDCl_3$)

1-bromo-2-((2,2-dimethyldeca-3,8-diyn-5-yl)oxy)naphthalene (1l)

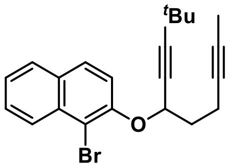

Alcohol **S11** (467.7 mg, 1.22 mmol, 1.0 eq.) in THF (12 mL, 0.1 M), 1-bromonaphthalen-2-ol (321.2 mg, 1.44 mmol, 1.2 eq.), PPh_3 (472.1 mg, 1.8 mmol, 1.5 eq.), and DIAD (412.5 mg, 400 µL, 2.04 mmol, 1.7 eq.) were reacted according to **GP3**. The reaction mixture was stirred at r.t. for 16 h. Silica gel column chromatography (*n*-pentane:EtOAc = 80:1) afforded the domino precursor **1l** (213.0 mg, 0.56 mmol, 46%) as yellow oil.

$\mathbf{R_f}$ = 0.34 (*n*-pentane:EtOAc = 80:1).

^{1}H-NMR (500 MHz, $CDCl_3$): δ = 8.24 (dq, J = 8.5, 0.8 Hz, 1H), 7.80 – 7.77 (m, 2H), 7.58 – 7.54 (m, 1H), 7.51 (d, J = 9.0 Hz, 1H), 7.41 (ddd, J = 8.1, 6.8, 1.2 Hz, 1H), 5.00 (dd, J = 7.1, 5.4 Hz, 1H), 2.63 – 2.53 (m, 1H), 2.47 (dddd, J = 16.6, 8.3, 5.9, 2.6 Hz, 1H), 2.30 – 2.20 (m, 1H), 2.13 (dddd, J = 13.5, 8.0, 7.3, 5.5 Hz, 1H), 1.77 (t, J = 2.5 Hz, 3H), 1.15 (s, 9H).

^{13}C-NMR (126 MHz, $CDCl_3$): δ = 152.7, 133.2, 130.5, 128.4, 128.1, 127.6, 126.6, 124.7, 118.0, 111.1, 96.9, 78.2, 76.4, 76.3, 69.9, 35.9, 30.9, 27.6, 15.1, 3.7.

IR (ATR): $\tilde{\nu}$ (cm^{-1}) = 2967, 1594, 1502, 1462, 1361, 1354, 1343, 1331, 1259, 1240, 1204, 1058, 1039, 1020, 1003, 958.

HRMS (APCI, Q-TOF): calculated for $C_{22}H_{24}BrO^+$ $[M+H]^+$: 383.1005, found: 383.1005.

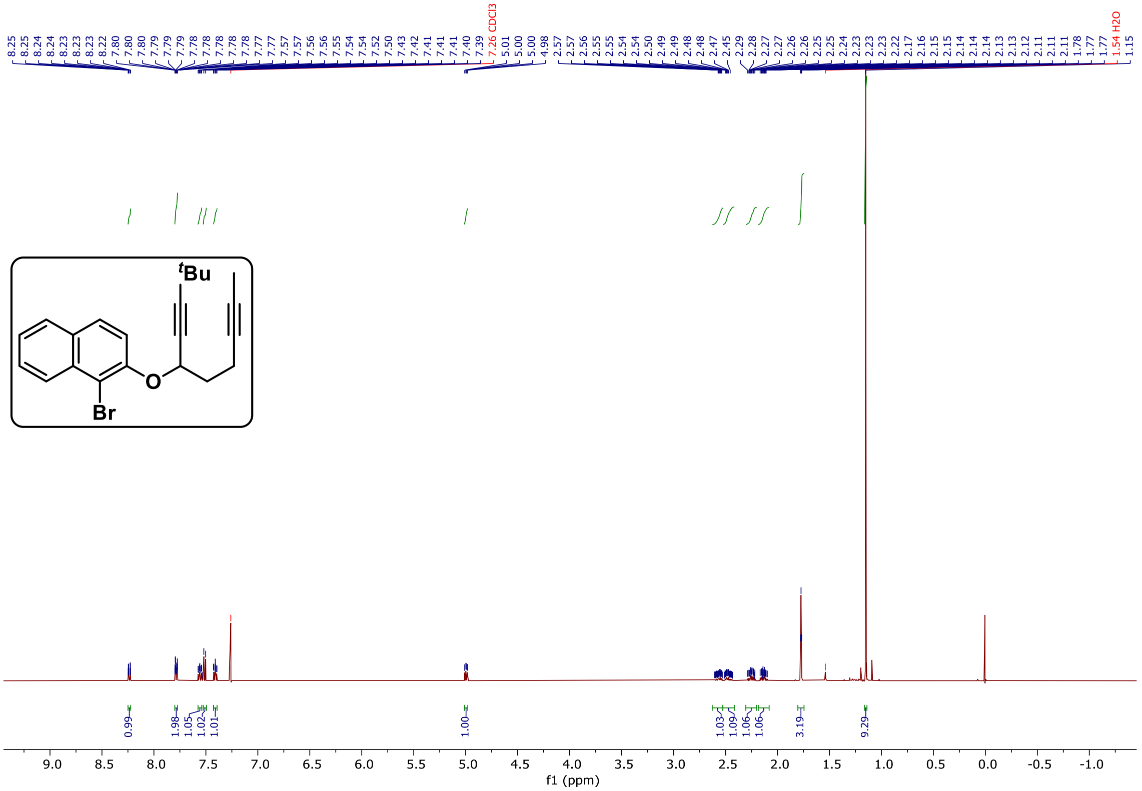

^{1}**H-NMR** (500 MHz, $CDCl_3$)

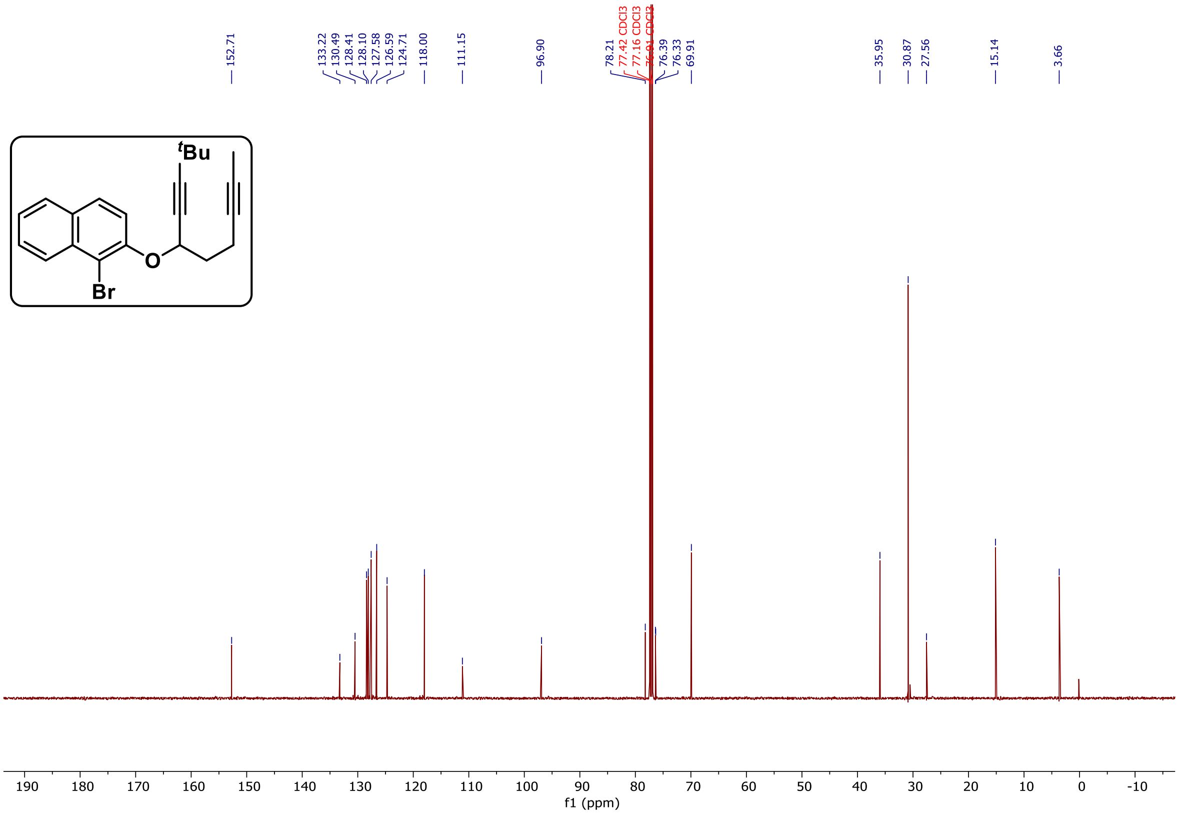

^{13}**C-NMR** (126 MHz, $CDCl_3$)

2-bromo-3-((2,2-dimethyldeca-3,8-diyn-5-yl)oxy)anthracene (1m)

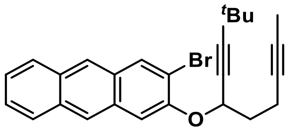

Alcohol **S11** (110.0 mg, 0.62 mmol, 1.0 eq.) in THF (6 mL, 0.1 M), 3-bromoanthracen-2-ol (207.6 mg, 0.76 mmol, 1.2 eq.), PPh_3 (258.9 mg, 0.99 mmol, 1.6 eq.), and DIAD (212.1 mg, 206 µL, 1.05 mmol, 1.7 eq.) were reacted according to **GP3**. The reaction mixture was stirred at r.t. for 16 h. Silica gel column chromatography (*n*-pentane:EtOAc = 80:1) afforded the domino precursor **1m** (150 mg, 0.35 mmol, 56%) as yellow oil.

R_f = 0.29 (*n*-pentane:EtOAc = 60:1).

^{1}H-NMR (700 MHz, $CDCl_3$): δ = 8.26 (t, *J* = 1.7 Hz, 2H), 8.23 (s, 1H), 7.98 – 7.93 (m, 2H), 7.59 (s, 1H), 7.47 – 7.41 (m, 2H), 5.02 (dd, *J* = 7.3, 5.3 Hz, 1H), 2.60 – 2.44 (m, 2H), 2.28 (dtd, *J* = 13.5, 7.6, 6.0 Hz, 1H), 2.21 – 2.12 (m, 1H), 1.78 (d, *J* = 5.1 Hz, 3H), 1.20 (s, 9H).

^{13}C-NMR (176 MHz, $CDCl_3$): δ = 151.2, 132.2, 132.2, 131.6, 130.9, 128.7, 128.3, 127.8, 125.8, 125.2, 125.1, 124.6, 115.8, 108.9, 97.1, 78.1, 76.3, 75.9, 68.6, 35.7, 30.9, 27.6, 15.1, 3.6.

IR (ATR): $\tilde{\nu}$ (cm^{-1}) = 2967, 2923, 1619, 1576, 1446, 1362, 1334, 1295, 1265, 1206, 1179, 1121, 1057, 1014.

HRMS (APCI, Q-TOF): calculated for $C_{26}H_{24}BrO^-$ $[M-H]^-$: 431.1016, found: 431.1019.

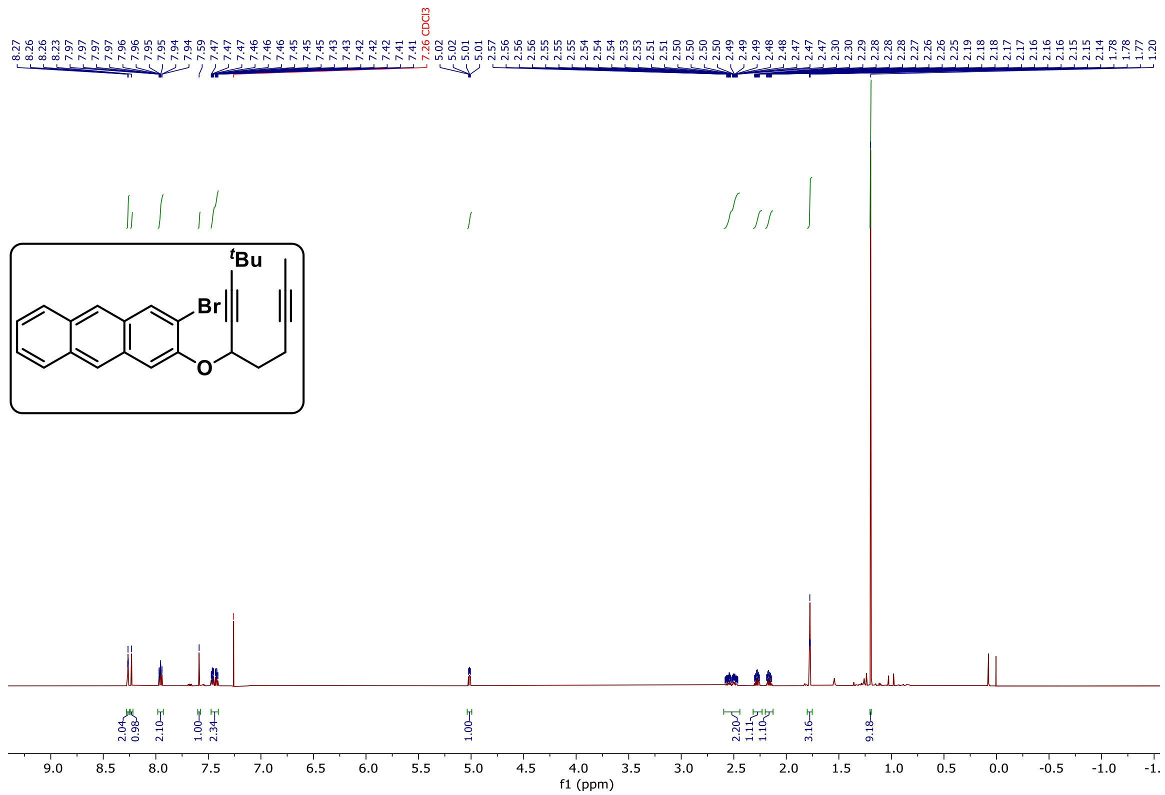

^{1}H-NMR (700 MHz, $CDCl_3$)

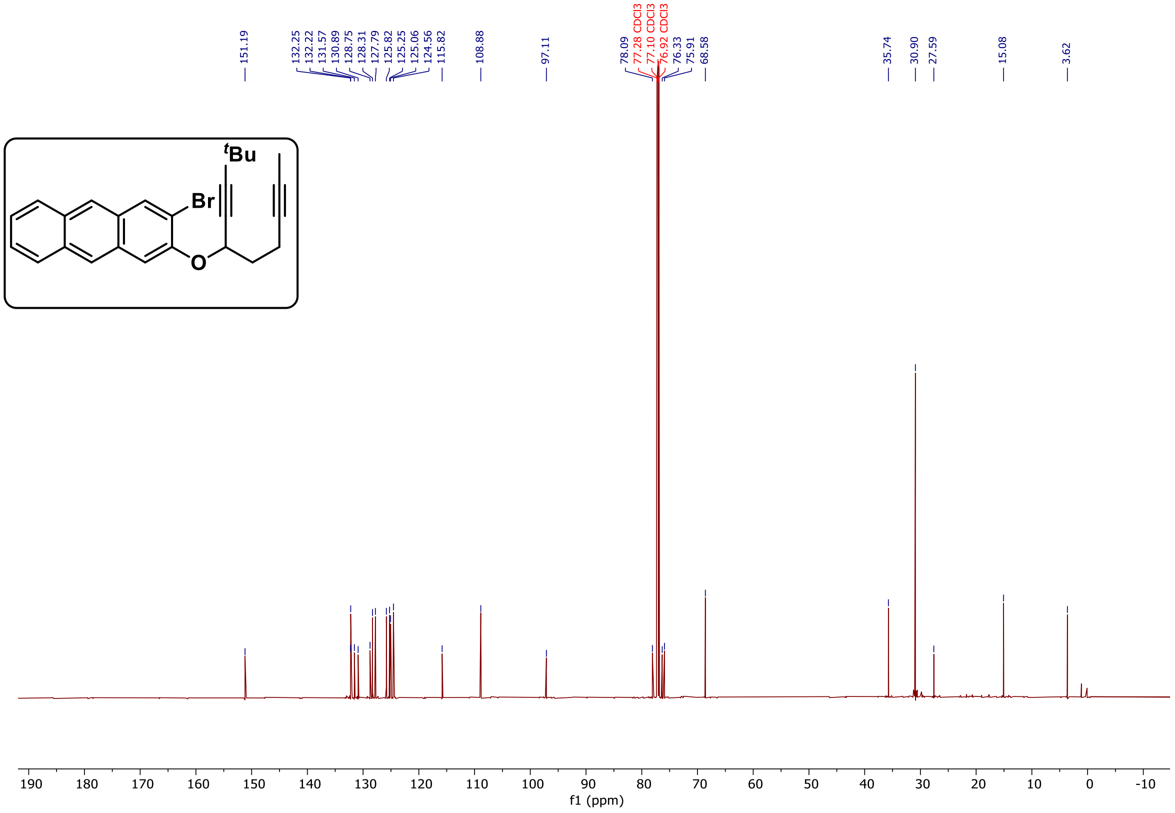

^{13}C-NMR (176 MHz, $CDCl_3$)

1-bromo-2-(3-(but-2-yn-1-yloxy)-6,6-dimethylhept-4-yn-1-yl)benzene (1n)

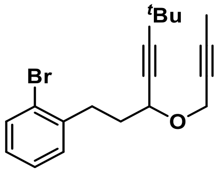

1-(2-bromophenyl)-6,6-dimethylhept-4-yn-3-ol[10] (450 mg, 1.52 mmol, 1.0 eq.), 1-bromobut-2-yne (243.3 mg, 160 µL, 1.83 mmol, 1.2 eq.), TBAI (56.4 mg, 0.15 mmol, 10.0 mol%), and NaH (60% on mineral oil, 91.4 mg, 2.29 mmol, 1.5 eq.) in DMF (30.5 mL, 0.05 M) were reacted according to **GP2**. The reaction mixture was stirred at r.t. for 15.5 h. The crude product was purified using silica gel column chromatography (*n*-pentane:EtOAc = 50:1) to afford the domino precursor **1n** (410 mg, 1.18 mmol, 77%) as yellow oil.

R_f = 0.28 (*n*-pentane:EtOAc = 50:1).

^{1}H-NMR (500 MHz, $CDCl_3$): δ = 7.52 (dd, *J* = 8.0, 1.3 Hz, 1H), 7.27 – 7.20 (m, 3H), 7.05 (ddd, *J* = 7.9, 7.2, 1.9 Hz, 1H), 4.31 (dq, *J* = 15.0, 2.3 Hz, 1H), 4.25 (t, *J* = 6.4 Hz, 1H), 4.17 (dq, *J* = 15.0, 2.3 Hz, 1H), 2.98 – 2.84 (m, 2H), 2.09 – 1.93 (m, 2H), 1.86 (d, *J* = 4.7 Hz, 3H), 1.24 (s, 9H).

^{13}C-NMR (126 MHz, $CDCl_3$): δ = 141.1, 132.9, 130.7, 127.7, 127.4, 124.6, 95.7, 82.3, 76.4, 75.3, 67.7, 56.2, 35.8, 32.0, 31.1, 27.5, 3.8.

IR (ATR): $\tilde{\nu}$ (cm^{-1}) = 2967, 1717, 1471, 1454, 1440, 1362, 1335, 1262, 1203, 1156, 1137, 1067, 1023, 962.

HRMS (APCI, Q-TOF): calculated for $C_{19}H_{24}BrO^+$ $[M+H]^+$: 347.1005, found: 347.1004.

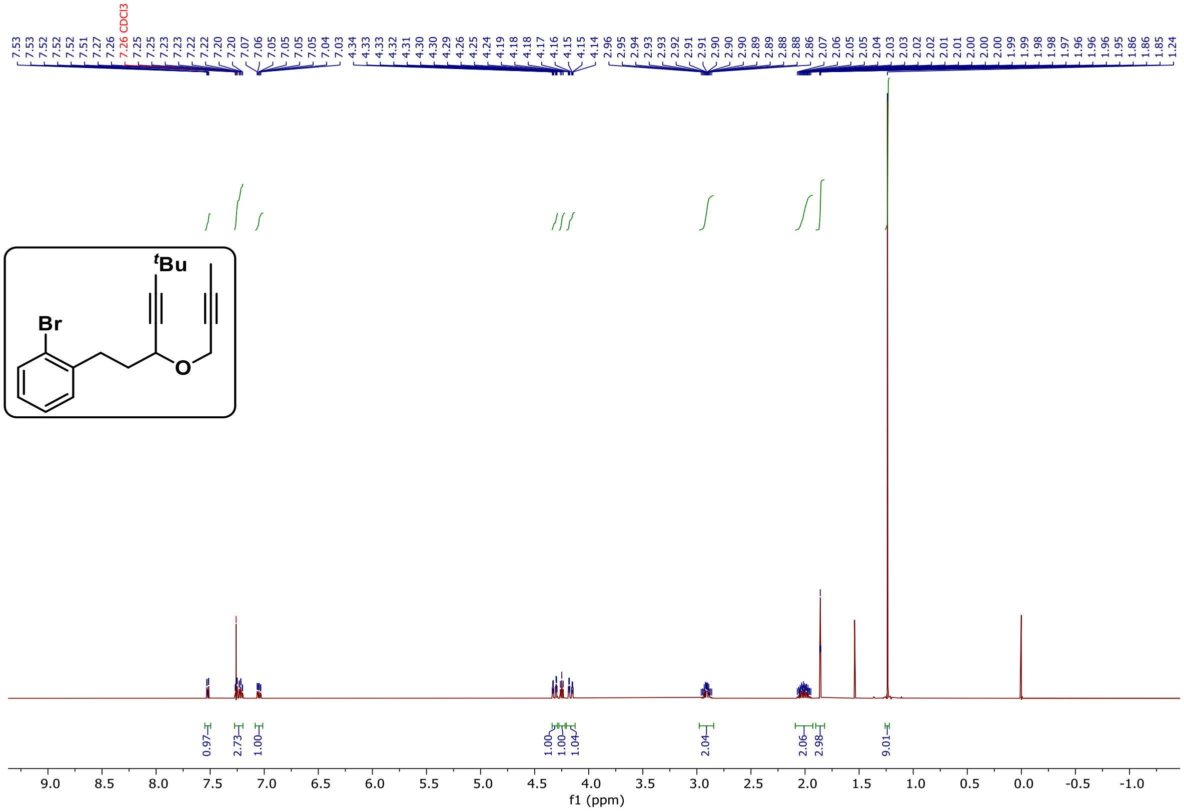

^{1}H-NMR (500 MHz, $CDCl_3$)

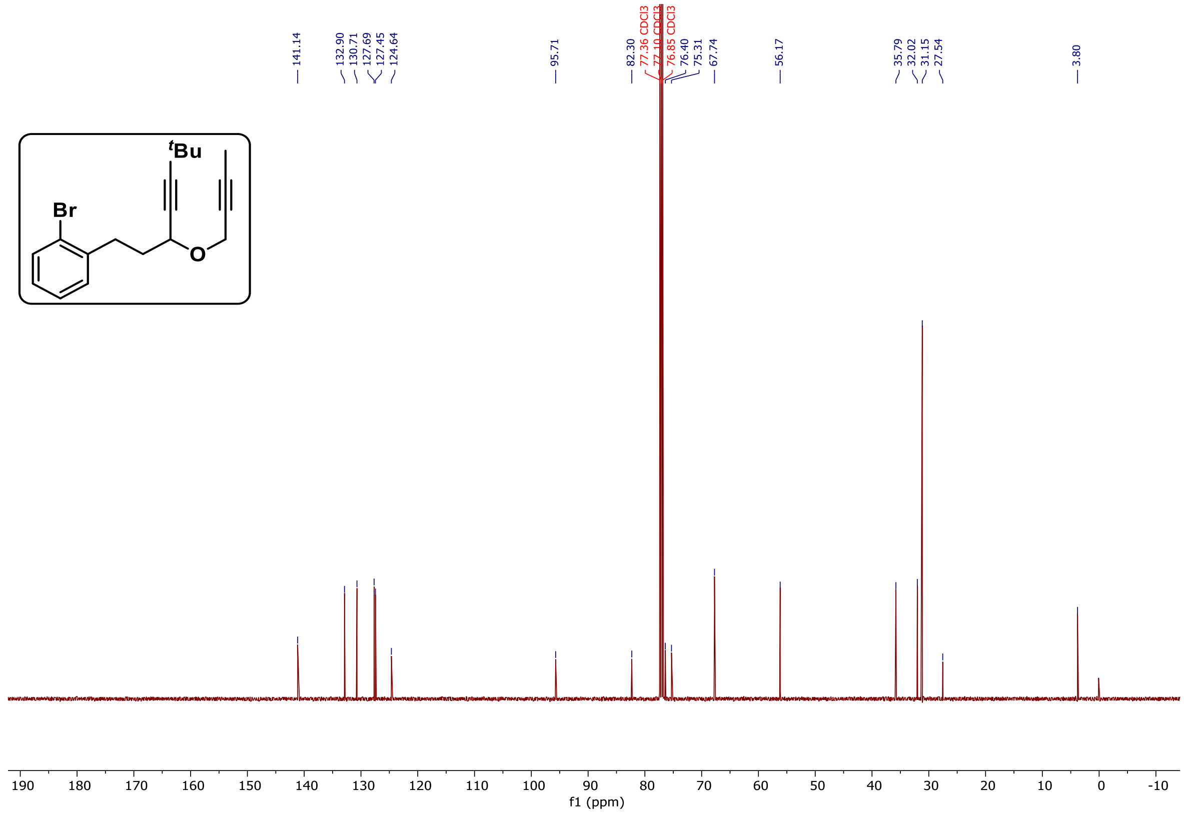

^{13}C-NMR (126 MHz, $CDCl_3$)

1-(3-(but-2-yn-1-yloxy)-6,6-dimethylhept-4-yn-1-yl)-2-iodobenzene (1n')

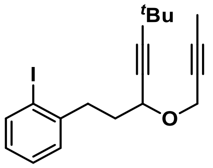

1-(2-iodophenyl)-6,6-dimethylhept-4-yn-3-ol[7] (450 mg, 1.31 mmol, 1.0 eq.), 1-bromobut-2-yne (262.3 mg, 173 µL, 1.97 mmol, 1.5 eq.), TBAI (24.3 mg, 0.07 mmol, 5.0 mol%), and NaH (60% on mineral oil, 68.1 mg, 1.7 mmol, 1.3 eq.) in DMF (26 mL, 0.05 M) were reacted according to **GP2**. The reaction mixture was stirred at r.t. for 16 h. The crude product was purified using silica gel column chromatography (*n*-pentane:EtOAc = 40:1) to afford the domino precursor **1n'** (350 mg, 0.89 mmol, 68%) as colorless oil.

R_f = 0.25 (*n*-pentane:EtOAc = 40:1).

^{1}H-NMR (400 MHz, $CDCl_3$): δ = 7.80 (dt, J = 7.9, 0.9 Hz, 1H), 7.26 – 7.24 (m, 2H), 6.92 – 6.82 (m, 1H), 4.32 (dq, J = 15.1, 2.4 Hz, 1H), 4.26 (t, J = 6.4 Hz, 1H), 4.17 (dq, J = 15.0, 2.3 Hz, 1H), 2.89 (t, J = 8.0 Hz, 2H), 2.06 – 1.92 (m, 2H), 1.86 (t, J = 2.3 Hz, 3H), 1.24 (s, 9H).

^{13}C-NMR (101 MHz, $CDCl_3$): δ = 144.5, 139.7, 129.8, 128.4, 127.9, 100.7, 95.8, 82.3, 76.5, 75.4, 67.7, 56.2, 36.7, 36.1, 31.2, 27.6, 3.8.

IR (ATR): $\tilde{\nu}$ (cm^{-1}) = 2966, 2862, 2234, 1727, 1587, 1562, 1466, 1435, 1362, 1334, 1262, 1203, 1157, 1137, 1066, 1009.

HRMS (APCI, Q-TOF): calculated for $C_{19}H_{24}IO^+$ $[M+H]^+$: 395.0866, found: 395.0872.

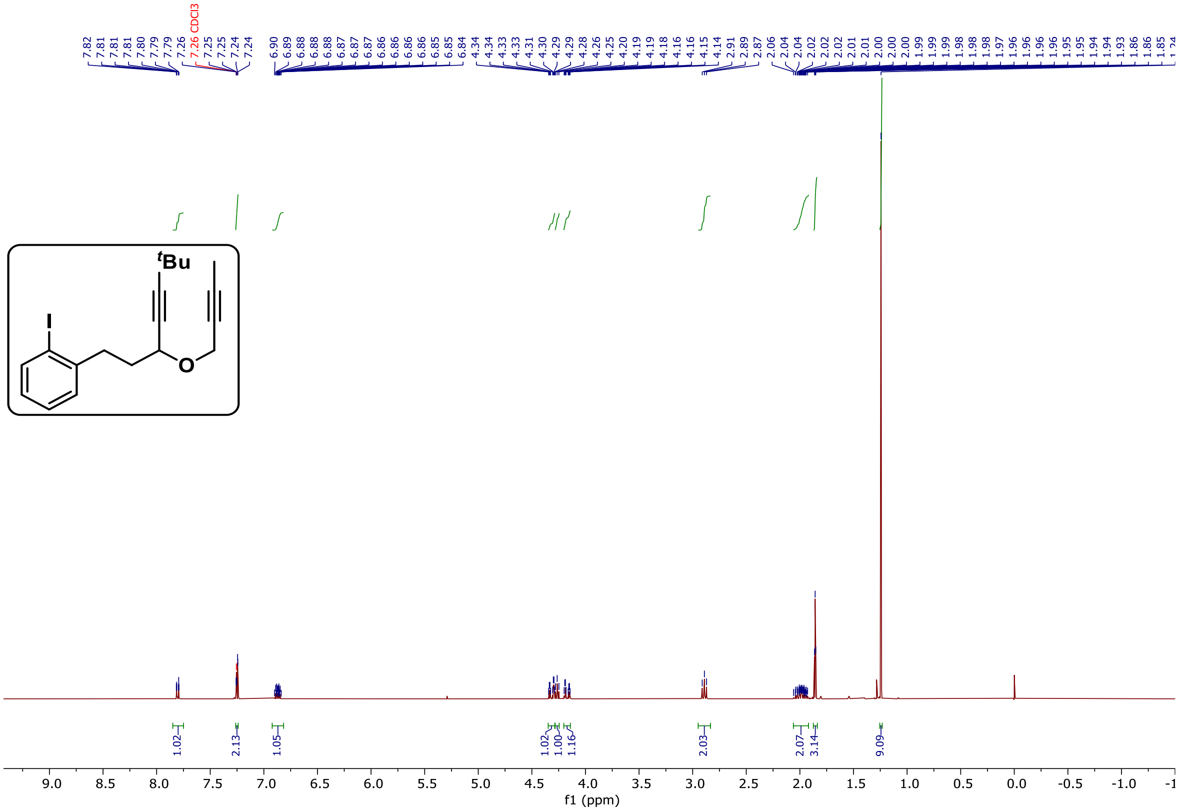

¹H-NMR (400 MHz, CDCl₃)

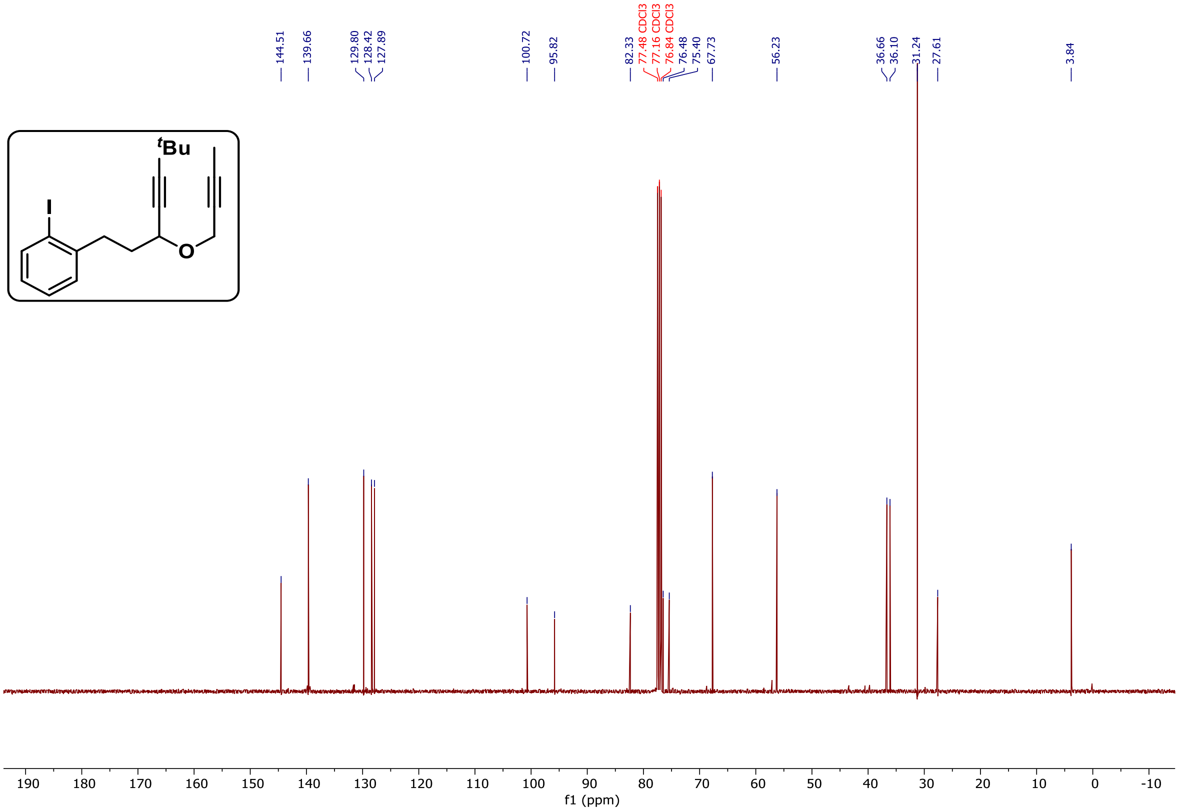

¹³C-NMR (101 MHz, CDCl₃)

Synthesis of 1o-1s

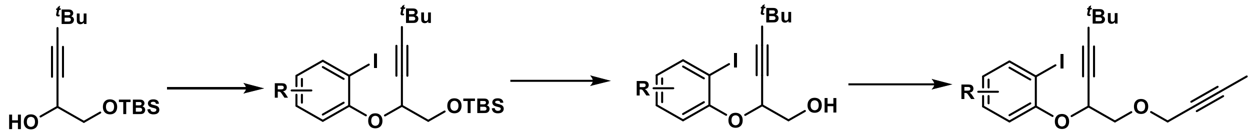

S12 S13-17 S18-22 1o-1s

2-(2-iodophenoxy)-5,5-dimethylhex-3-yn-1-ol (S18)

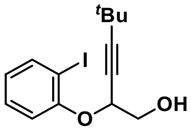

The protected alcohol (**S12**) (400.0 mg, 1.56 mmol, 1.0 eq.) in THF (15.5 mL, 0.1 M), 2-iodophenol (411.9 mg, 1.87 mmol, 1.2 eq.), PPh_3 (593 mg, 2.62 mmol, 1.7 eq.), and DIAD (505.0 mg, 490 µL, 2.50 mmol, 1.6 eq.) were reacted according to **GP3**. The reaction mixture was stirred at r.t. for 16 h. The crude product was used for the deprotection step without further purification. The TBS-protected crude **S13** was reacted with acetyl chloride (719.0 mg, 0.65 mL, 9.16 mmol, 6.0 eq.) in a mixture of CH_2Cl_2:MeOH (each 1.6 mL, 1.0 M) according to **GP4**. The reaction mixture was stirred at r.t. for 3 h. The residue was purified by silica gel column chromatography (*n*-pentane:EtOAc = 10:1) to afford **S18** (165 mg, 0.48 mmol, 37%) as a colorless oil. The characterization data are consistent with our recent literature.[7]

1-((1-(but-2-yn-1-yloxy)-5,5-dimethylhex-3-yn-2-yl)oxy)-2-iodobenzene (1o)

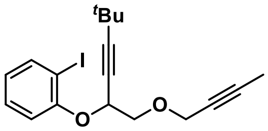

The alcohol **S18** (165 mg, 0.48 mmol, 1.0 eq.), 1-bromobut-2-yne (76.5 mg, 50 µL, 0.57 mmol, 1.2 eq.), TBAI (35.4 mg, 0.1 mmol, 20.0 mol%), and NaH (60% on mineral oil, 17.2 mg, 0.72 mmol, 1.5 eq.) in DMF (9.5 mL, 0.05 M) were reacted according to **GP2**. The reaction mixture was stirred at r.t. for 17.5 h. The crude product was purified using silica gel column chromatography (*n*-pentane:EtOAc = 80:1) to afford the domino precursor **1o** (153 mg, 0.39 mmol, 81%) as a colorless oil.

R_f = 0.36 (*n*-pentane:EtOAc = 80:1).

^{1}H-NMR (700 MHz, $CDCl_3$): δ = 7.77 – 7.76 (ddd, *J* = 7.8, 1.7, 0.6 Hz, 1H), 7.29 – 7.26 (dddd, *J* = 8.1, 7.3, 1.7, 0.6 Hz, 1H), 7.08 – 7.07 (dd, *J* = 8.2, 1.4 Hz, 1H), 6.74 – 6.72 (tdd, *J* = 7.3, 1.4,

0.6 Hz, 1H), 4.93 – 4.91 (m, 1H), 4.39 – 4.30 (m, 2H), 3.96 – 3.90 (m, 2H), 1.86 (td, *J* = 2.3, 0.6 Hz, 3H), 1.16 (s, 9H).

^{13}C-NMR (176 MHz, $CDCl_3$): δ = 3.8, 27.6, 30.8, 60.0, 70.4, 72.3, 73.7, 75.3, 82.8, 87.9, 97.6, 115.4, 123.3, 129.2, 139.5, 156.9.

IR (ATR): $\tilde{\nu}$ (cm^{-1}) = 2968, 2922, 2865, 2238, 1580, 1570, 1469, 1439, 1391, 1360, 1341, 1276, 1262, 1236, 1204, 1157, 1141, 1120, 1093, 1044, 1018.

HRMS (APCI, Q-TOF): calculated for $C_{18}H_{22}IO_2^+$ $[M+H]^+$: 397.0659, found 397.0660.

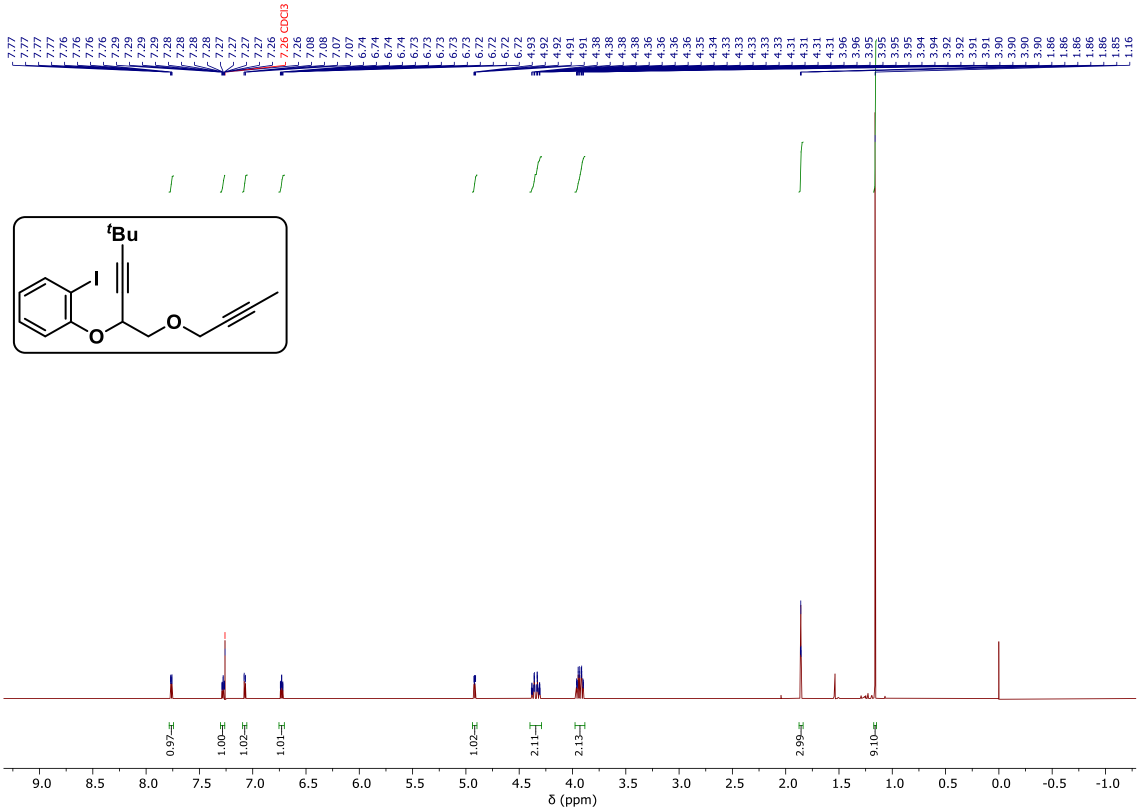

^{1}H-NMR (700 MHz, $CDCl_3$)

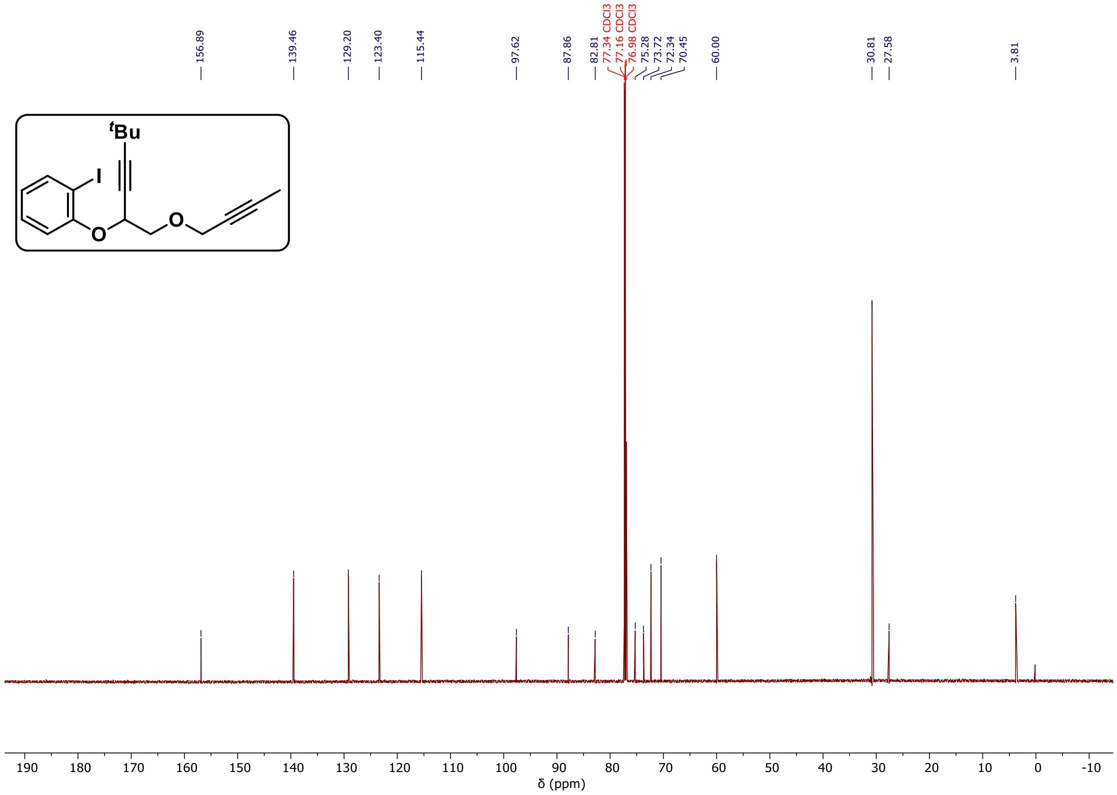

^{13}C-NMR (176 MHz, $CDCl_3$)

2-(4-(*tert*-butyl)-2-iodophenoxy)-5,5-dimethylhex-3-yn-1-ol (S19)

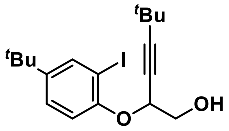

The protected alcohol (**S12**) (400 mg, 1.56 mmol, 1.0 eq.) in THF (15.5 mL, 0.1 M), 4-*tert*-butyl-2-iodophenol (503 mg, 1.82 mmol, 1.2 eq.), PPh_3 (593 mg, 2.62 mmol, 1.7 eq.), and DIAD (505 mg, 490 µL, 2.50 mmol, 1.6 eq.) were reacted according to **GP3**. The reaction mixture was stirred at r.t. for 5 h. The crude product was used for the deprotection step without further purification. The TBS-protected crude **S14** was reacted with acetyl chloride (547 mg, 0.50 mL, 6.97 mmol, 4.5 eq.) in a mixture of CH_2Cl_2:MeOH (each 1.6 mL, 1.0 M) according to **GP4**. The reaction mixture was stirred at r.t. for 4 h. The residue was purified by silica gel column chromatography (*n*-pentane:EtOAc = 10:1) to afford **S19** (262 mg, 0.65 mmol, 66%) as a colorless oil.

$\mathbf{R_f}$ = 0.44 (*n*-pentane:EtOAc = 10:1).

^{1}H-NMR (500 MHz, $CDCl_3$): δ = 7.75 (d, *J* = 2.4 Hz, 1H), 7.33 – 7.31 (dd, *J* = 8.6, 2.4 Hz, 1H), 7.12 – 7.10 (d, *J* = 8.7 Hz, 1H), 4.73 – 4.71 (dd, *J* = 8.3, 3.6 Hz, 1H), 3.99 – 3.94 (m, 1H), 3.84 – 3.79 (ddd, *J* = 11.9, 10.2, 3.6 Hz, 1H), 2.50 – 2.47 (dd, *J* = 10.2, 3.9 Hz, 1H), 1.29 (s, 9H), 1.19 (s, 9H).

^{13}C-NMR (126 MHz, $CDCl_3$): δ = 27.6, 30.8, 31.5, 34.3, 65.8, 72.4, 73.6, 88.0, 98.1, 115.2, 126.5, 136.2, 147.1, 154.4.

IR (ATR): $\tilde{\nu}$ (cm^{-1}) = 2963, 1492, 1482, 1458, 1390, 1362, 1288, 1258, 1245, 1204, 1067, 1051, 1037, 1015.

HRMS (APCI, Q-TOF): calculated for $C_{18}H_{26}IO_2^+$ $[M+H]^+$: 401.0972, found 401.0974.

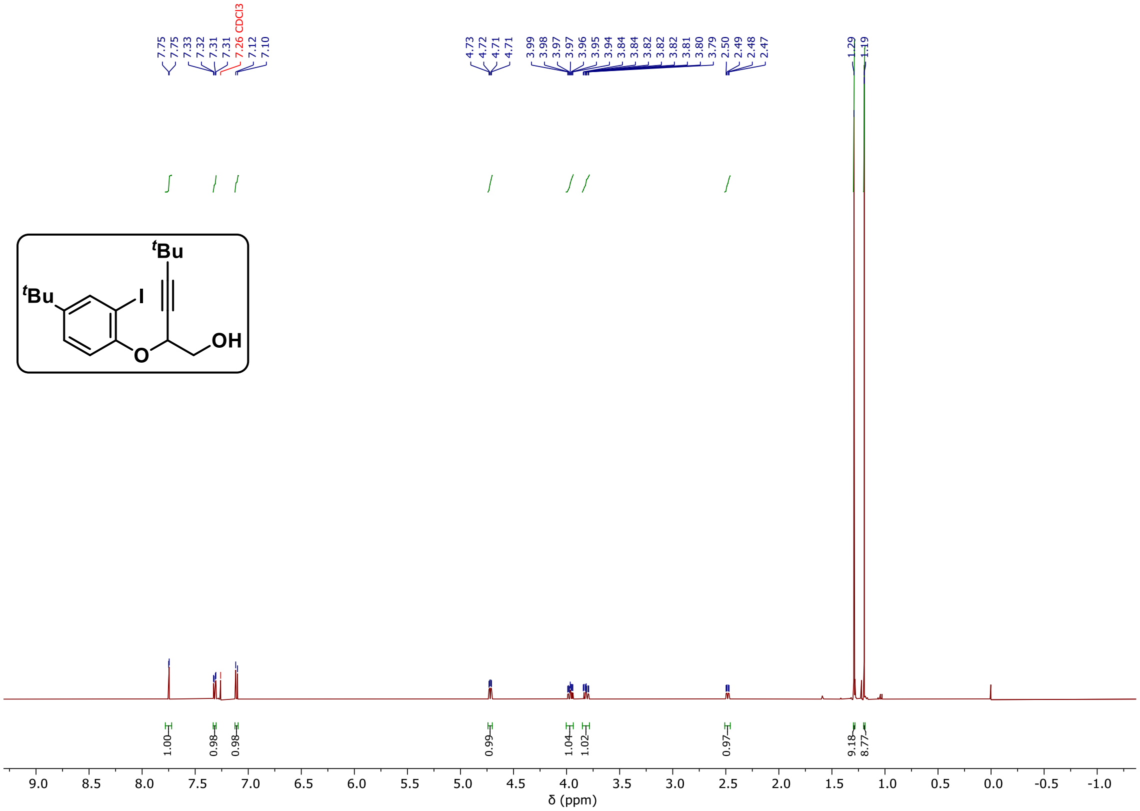

^{1}H-NMR (500 MHz, $CDCl_3$)

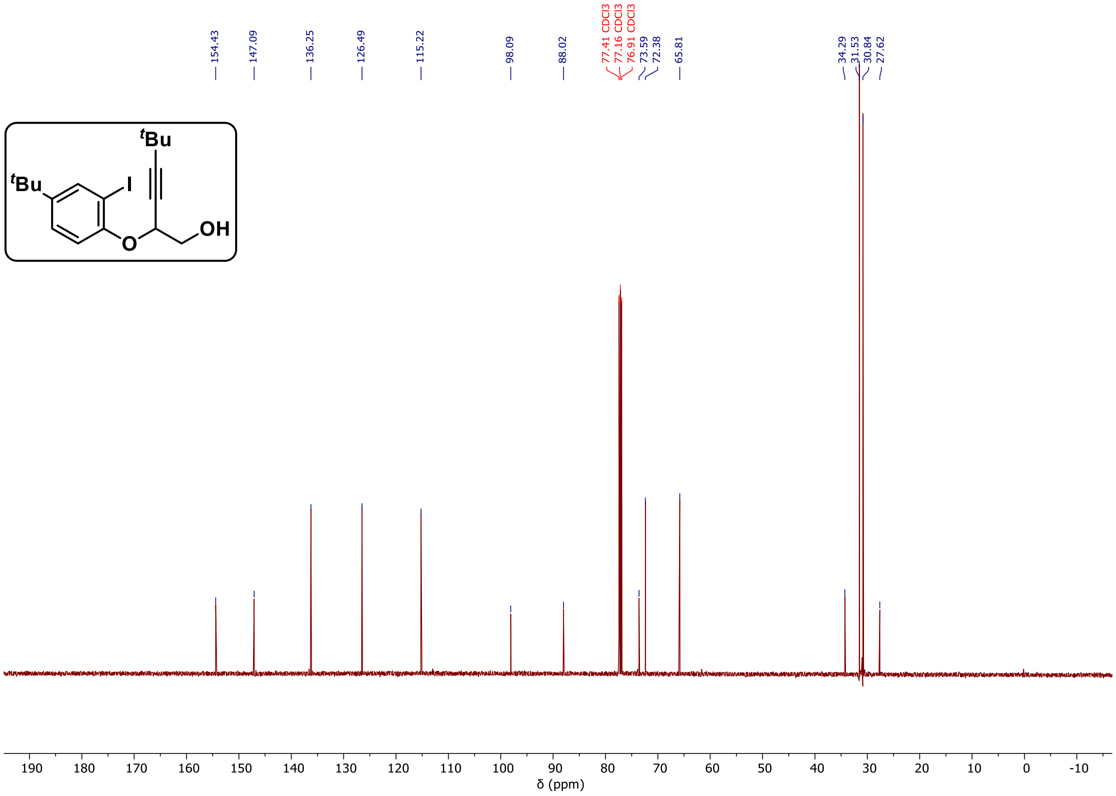

^{13}C-NMR (126 MHz, $CDCl_3$)

1-((1-(but-2-yn-1-yloxy)-5,5-dimethylhex-3-yn-2-yl)oxy)-4-(*tert*-butyl)-2-iodobenzene (1p)

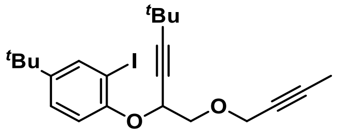

The alcohol **S19** (220 mg, 0.55 mmol, 1.0 eq.), 1-bromobut-2-yne (87.7 mg, 58 µL, 0.66 mmol, 1.2 eq.), TBAI (41.0 mg, 0.11 mmol, 20.0 mol%), and NaH (60% on mineral oil, 19.8 mg, 0.82 mmol, 1.5 eq.) in DMF (11 mL, 0.05 M) were reacted according to **GP2**. The reaction mixture was stirred at r.t. for 16 h. The crude product was purified using silica gel column chromatography (*n*-pentane:EtOAc = 80:1) to afford the domino precursor **1p** (169 mg, 0.37 mmol, 68%) as a colorless oil.

R_f = 0.52 (*n*-pentane:EtOAc = 40:1).

^{1}H-NMR (500 MHz, $CDCl_3$): δ = 7.75 – 7.74 (d, *J* = 2.3 Hz, 1H), 7.29 – 7.27 (dd, *J* = 8.6, 2.4 Hz, 1H), 7.01 – 6.99 (d, *J* = 8.6 Hz, 1H), 4.88 – 4.86 (dd, *J* = 7.5, 4.0 Hz, 1H), 4.39 – 4.29 (m, 2H), 3.95 – 3.87 (m, 2H), 1.86 – 1.85 (t, *J* = 2.3 Hz ,3H), 1.28 (s, 9H), 1.16 (s, 9H).

^{13}C-NMR (126 MHz, $CDCl_3$): δ = 3.8, 27.6, 30.8, 31.5, 34.2, 60.0, 70.7, 72.4, 74.0, 75.3, 82.7, 87.8, 97.4, 115.1, 126.2, 136.4, 146.6, 154.8.

IR (ATR): $\tilde{\nu}$ (cm^{-1}) = 2963, 2922, 2903, 2866, 1593, 1490, 1483, 1459, 1390, 1361, 1340, 1288, 1258, 1247, 1203, 1157, 1141, 1092, 1038, 1007.

HRMS (APCI, Q-TOF): calculated for $C_{22}H_{30}IO_2^+$ [M+H]$^+$: 453.1285, found 453.1289.

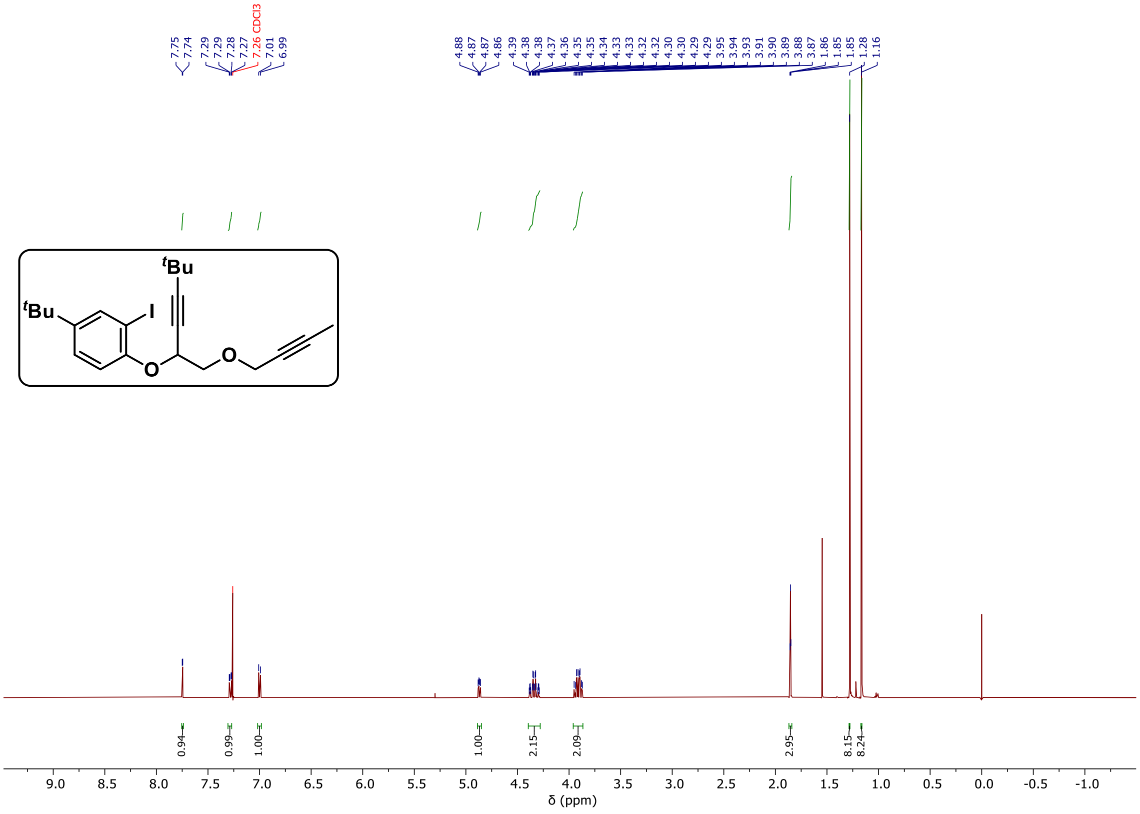

^{1}H-NMR (500 MHz, $CDCl_3$)

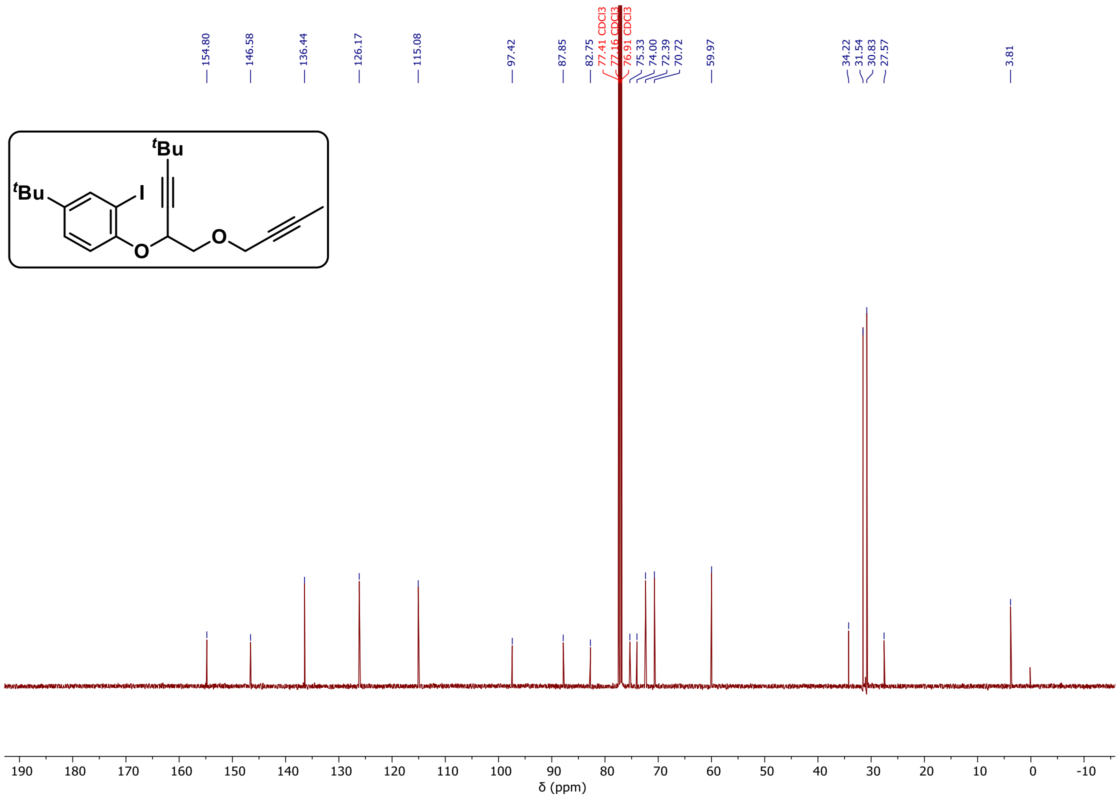

^{13}C-NMR (126 MHz, $CDCl_3$)

2-(2-iodo-4-nitrophenoxy)-5,5-dimethylhex-3-yn-1-ol (S20)

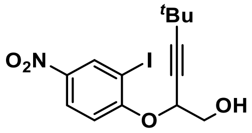

The protected alcohol (**S12**) (306 mg, 1.19 mmol, 1.0 eq.) in THF (12 mL, 0.1 M), 2-iodo-4-nitrophenol (379 mg, 1.43 mmol, 1.2 eq.), PPh_3 (454 mg, 1.73 mmol, 1.4 eq.), and DIAD (386 mg, 380 µL, 1.91 mmol, 1.6 eq.) were reacted according to **GP3**. The reaction mixture was stirred at r.t. for 16 h. The crude product was used for the deprotection step without further purification. The TBS-protected crude **S15** was reacted with acetyl chloride (458 mg, 420 µL, 5.84 mmol, 4.9 eq.) in a mixture of CH_2Cl_2:MeOH (each 1.2 mL, 1.0 M) according to **GP4**. The residue was purified by silica gel column chromatography (*n*-pentane:EtOAc =10:1) to afford **S20** (236 mg, 0.61 mmol, 73%) as a colorless oil.

$\mathbf{R_f}$ = 0.44 (*n*-pentane:EtOAc = 10:1).

^{1}H-NMR (500 MHz, $CDCl_3$): δ = 8.67 (d, *J* = 2.7 Hz, 1H), 8.25 (dd, *J* = 9.1, 2.7 Hz, 1H), 7.17 (d, *J* = 9.1 Hz, 1H), 4.89 (dd, *J* = 8.0, 3.5 Hz, 1H), 4.08 – 3.89 (m, 2H), 1.23 (s, 1H), 1.19 (s, 9H).

^{13}C-NMR (126 MHz, $CDCl_3$): δ = 27.7, 30.7, 65.5, 72.3, 77.4, 86.5, 99.7, 113.0, 125.5, 135.1, 142.6, 161.2.

IR (ATR): $\tilde{\nu}$ (cm^{-1}) = 2968, 2928, 1594, 1577, 1514, 1470, 1392, 1363, 1336, 1297, 1267, 1259, 1248, 1205, 1146, 1115, 1064, 1033, 1011.

HRMS (APCI, Q-TOF): calculated for $C_{14}H_{17}INO_4^+$ $[M+H]^+$: 390.0197, found 390.0206.

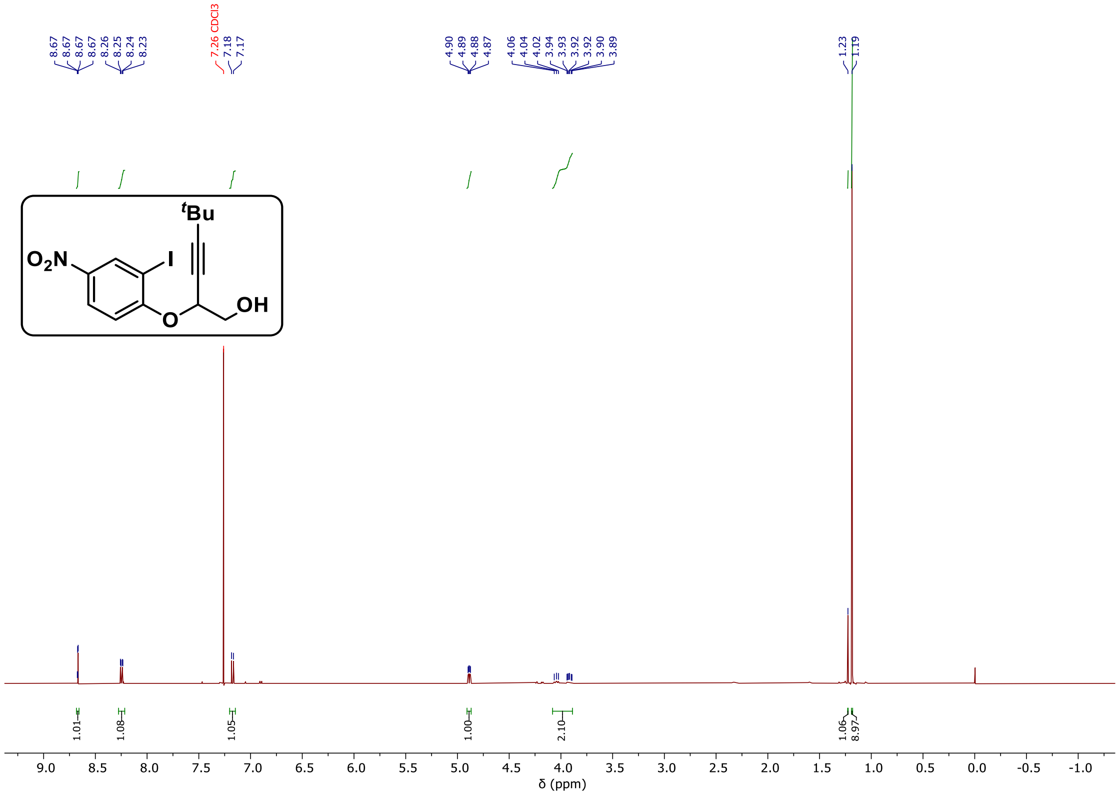

^{1}H-NMR (500 MHz, CDCl$_3$)

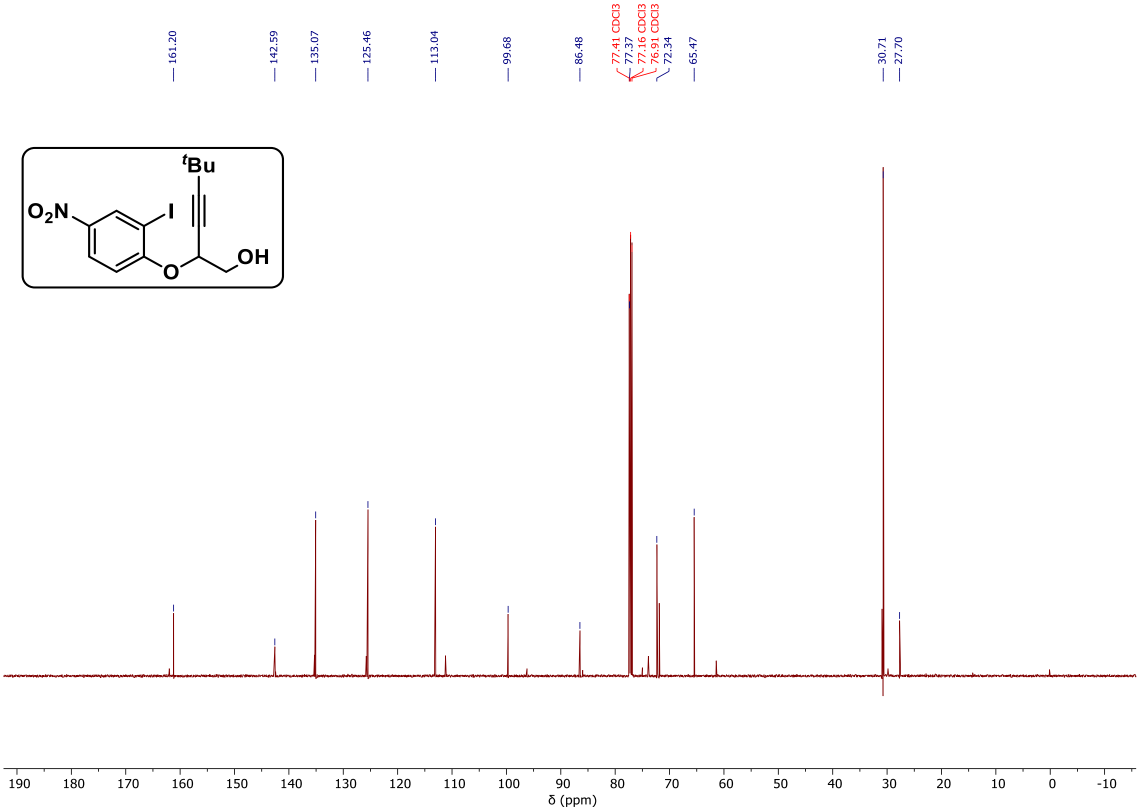

^{13}C-NMR (126 MHz, CDCl$_3$)

1-((1-(but-2-yn-1-yloxy)-5,5-dimethylhex-3-yn-2-yl)oxy)-2-iodo-4-nitrobenzene (1q)

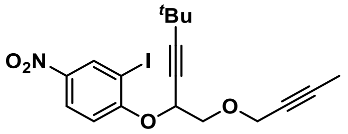

The alcohol **S20** (236 mg, 0.61 mmol, 1.0 eq.), 1-bromobut-2-yne (97 mg, 64 µL, 0.73 mmol, 1.2 eq.), TBAI (44.8 mg, 0.12 mmol, 20.0 mol%), and NaH (60% on mineral oil, 21.8 mg, 0.91 mmol, 1.5 eq.) in DMF (12 mL, 0.05 M) were reacted according to **GP2**. The reaction mixture was stirred at r.t. overnight. The crude product was purified using silica gel column chromatography (*n*-pentane:EtOAc = 80:1) to afford the domino precursor **1q** (88 mg, 0.20 mmol, 33%) as a colorless oil.

$\mathbf{R_f}$ = 0.36 (*n*-pentane:EtOAc = 80:1).

^{1}H-NMR (700 MHz, $CDCl_3$): δ = 8.67 – 8.66 (t, *J* = 2.4 Hz, 1H), 8.23 – 8.21 (dt, *J* = 8.9, 2.1 Hz, 1H), 6.91 – 6.89 (d, *J* = 9.1 Hz, 1H), 4.75 – 4.74 (ddd, *J* = 7.2, 4.2, 0.9 Hz, 1H), 4.39 – 4.22 (m, 4H), 1.85 (s, 3H), 1.23 (s, 9H).

^{13}C-NMR (176 MHz, $CDCl_3$): δ = 3.9, 27.6, 31.0, 57.0, 67.0, 72.4, 73.1, 74.7, 83.1, 85.8, 97.3, 111.2, 125.6, 135.3, 142.2, 162.5.

IR (ATR): $\tilde{\nu}$ (cm^{-1}) = 3521, 3332, 2927, 2857, 2778, 2510, 2328, 2045, 1956, 1727, 1665, 1499, 1438, 1405, 1385, 1255, 1151, 1089, 1063.

HRMS (APCI, Q-TOF): calculated for $C_{18}H_{21}INO_4^+$ $[M+H]^+$: 442.0510, found 442.0506.

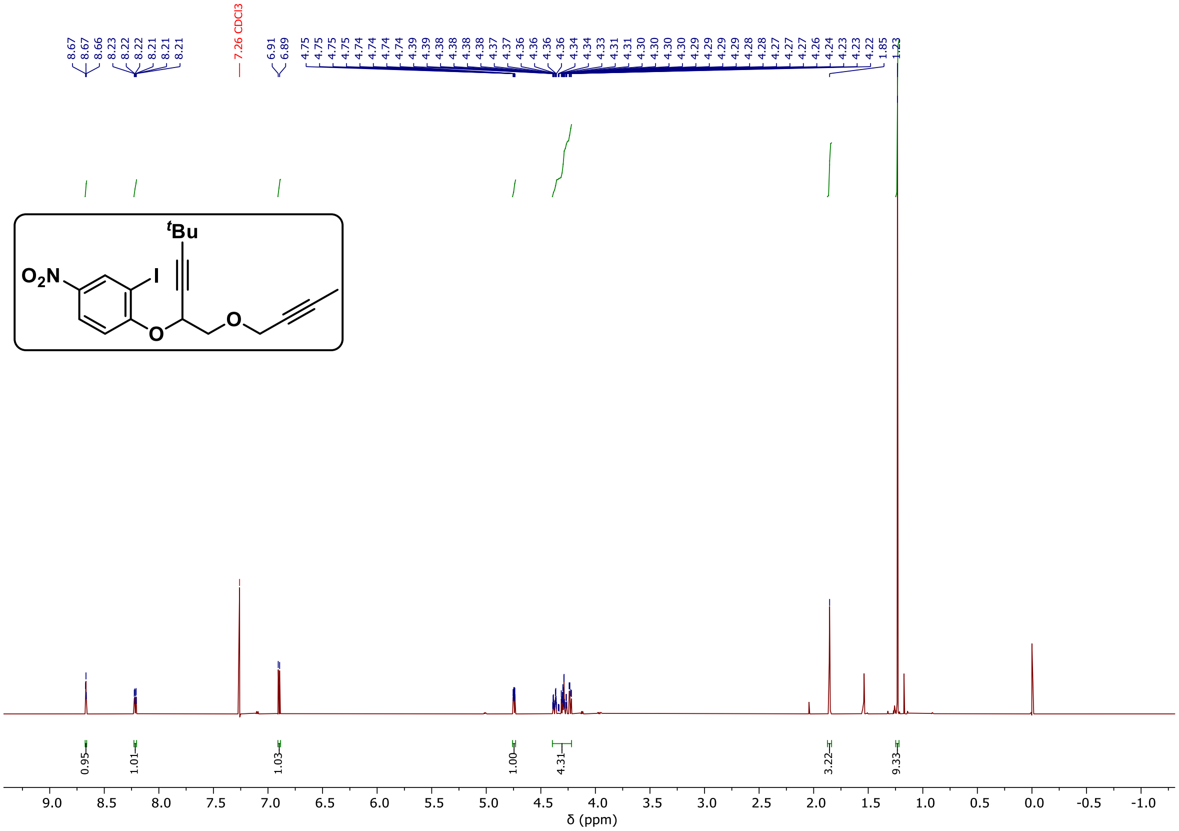

^{1}H-NMR (700 MHz, $CDCl_3$)

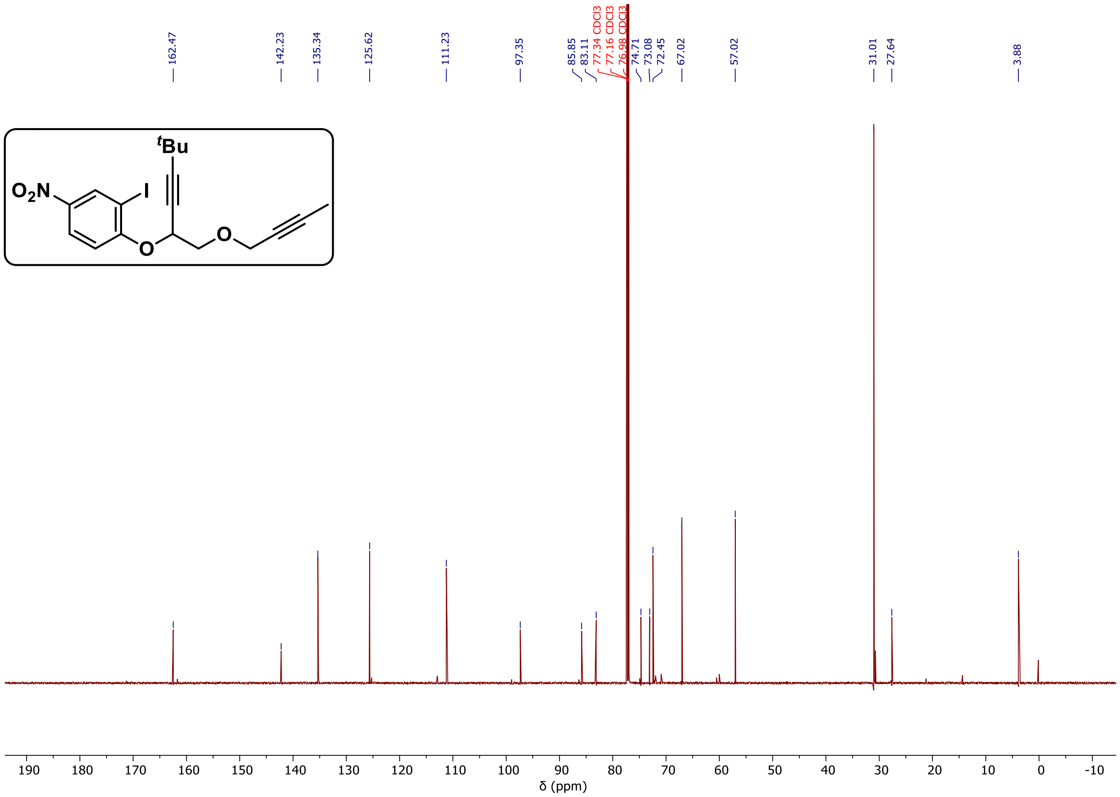

^{13}C-NMR (176 MHz, $CDCl_3$)

2-(2-iodo-5-methoxyphenoxy)-5,5-dimethylhex-3-yn-1-ol (S21)

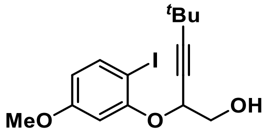

The protected alcohol (**S12**) (500 mg, 1.95 mmol, 1.0 eq.) in THF (19.5 mL, 0.1 M), 2-iodo-5-methoxyphenol (585 mg, 2.34 mmol, 1.2 eq.), PPh_3 (741 mg, 2.82 mmol, 1.4 eq.), and DIAD (630 mg, 0.61 mL, 3.12 mmol, 1.6 eq.) were reacted according to **GP3**. The reaction mixture was stirred at r.t. for 17.5 h. The crude product was used for the deprotection step without further purification. The TBS-protected crude **S16** was reacted with acetyl chloride (649.0 mg, 590 µL, 8.29 mmol, 4.2 eq.) in a mixture of CH_2Cl_2:MeOH (each 2.0 mL, 1.0 M) according to **GP4**. The residue was purified by silica gel column chromatography (*n*-pentane:EtOAc = 10:1) to afford **S21** (133 mg, 0.36 mmol, 43%) as a colorless oil.

R_f = 0.44 (*n*-pentane:EtOAc = 10:1).

^{1}H-NMR (500 MHz, $CDCl_3$): δ = 7.61 – 7.59 (d, *J* = 8.6 Hz, 1H), 6.84 – 6.83 (d, *J* = 2.7 Hz, 1H), 6.41 – 6.39 (dd, *J* = 8.7, 2.7 Hz, 1H), 4.74 – 4.72 (dd, *J* = 8.3, 3.5 Hz, 1H), 4.01 – 3.95 (m, 1H), 3.85– 3.82 (ddd, *J* = 11.9, 10.2, 3.5 Hz, 1H), 3.80 (s, 3H), 2.48 – 2.45 (dd, *J* = 10.3, 4.0 Hz, 1H), 1.20 (s, 9H).

^{13}C-NMR (126 MHz, $CDCl_3$): δ = 27.6, 30.9, 55.6, 65.7, 72.2, 73.3, 76.4, 98.4, 102.7, 109.6, 138.9, 157.3, 161.3.

IR (ATR): $\tilde{\nu}$ (cm^{-1}) = 3414, 2967, 2930, 2902, 2868, 2836, 1581, 1474, 1442, 1419, 1406, 1362, 1334, 1299, 1278, 1258, 1200, 1166, 1133, 1117, 1065, 1051, 1013.

HRMS (APCI, Q-TOF): calculated for $C_{15}H_{20}IO_3^+$ $[M+H]^+$: 375.0452, found 375.0455.

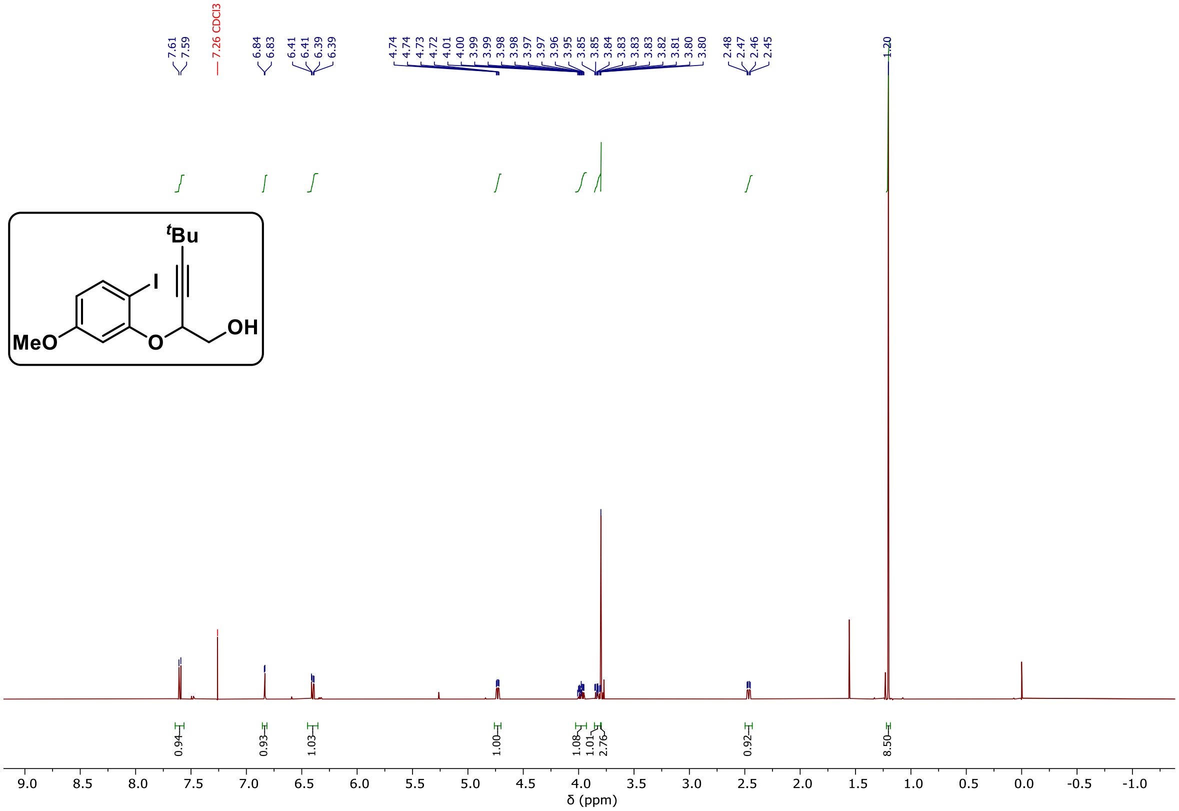

^{1}H-NMR (500 MHz, $CDCl_3$)

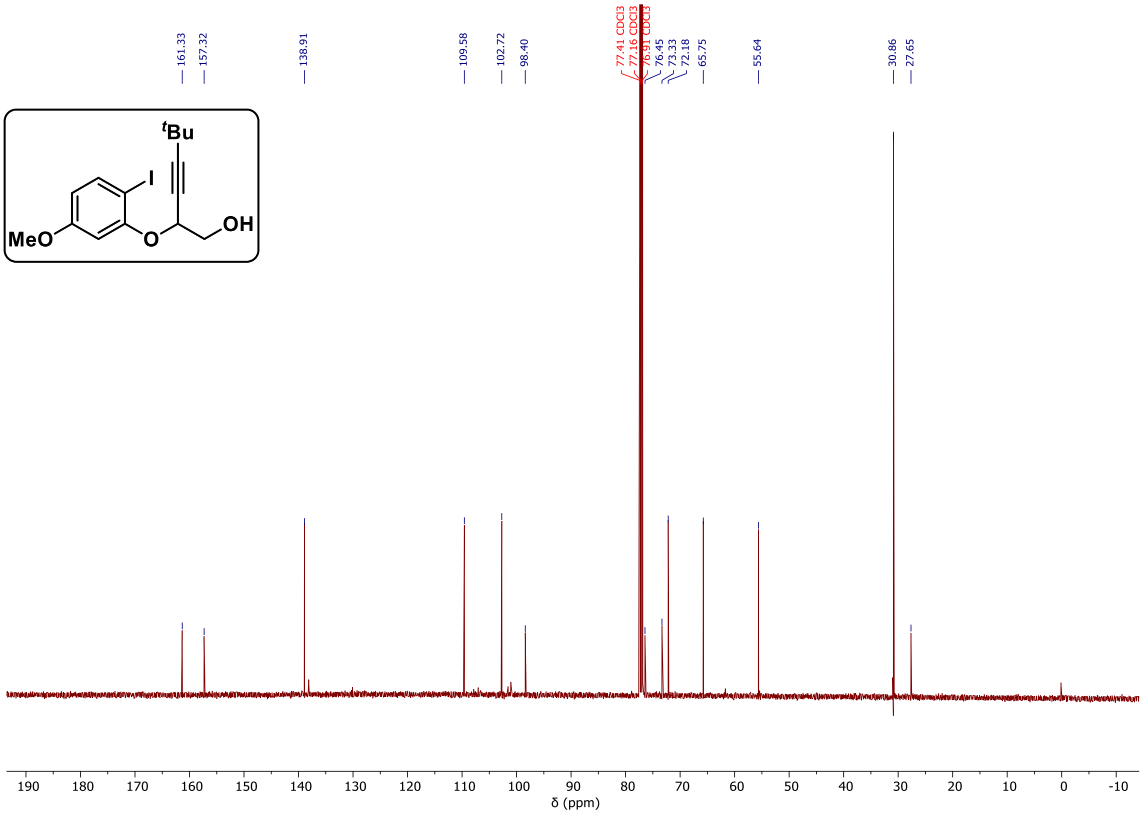

^{13}C-NMR (126 MHz, $CDCl_3$)

2-((1-(but-2-yn-1-yloxy)-5,5-dimethylhex-3-yn-2-yl)oxy)-1-iodo-4-methoxybenzene (1r)

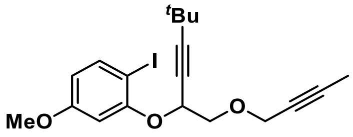

The alcohol **S21** (133 mg, 0.36 mmol, 1.0 eq.), 1-bromobut-2-yne (56.7 mg, 37 µL, 0.43 mmol, 1.2 eq.), TBAI (26.3 mg, 0.07 mmol, 20.0 mol%), and NaH (60% on mineral oil,14 mg, 0.53 mmol, 1.5 eq.) in DMF (7 mL, 0.05 M) were reacted according to **GP2**. The reaction mixture was stirred at r.t. for 16.5 h. The crude product was purified using silica gel column chromatography (*n*-pentane:EtOAc = 80:1) to afford the domino precursor **1r** (100 mg, 0.23 mmol, 66%) as a colorless oil.

R$_f$ = 0.36 (*n*-pentane:EtOAc = 80:1).

^{1}H-NMR (500 MHz, $CDCl_3$): δ = 7.61 – 7.59 (d, *J* = 8.6 Hz, 1H), 6.73 – 6.72 (d, *J* = 2.7 Hz, 1H), 6.36 (dd, *J* = 8.8, 2.7 Hz, 1H), 4.89 – 4.87 (dd, *J* = 7.4, 3.9 Hz, 1H), 4.39 – 4.29 (m, 2H), 3.96 – 3.88 (m, 2H), 3.78 (s, 3H), 1.86 – 1.85 (t, *J* = 2.3 Hz, 3H), 1.18 (s, 9H).

^{13}C-NMR (126 MHz, $CDCl_3$): δ = 3.8, 27.6, 30.8, 55.6, 60.0, 70.5, 72.3, 73.7, 75.3, 76.4, 82.8, 97.7, 102.6, 109.1, 139.1, 157.7, 161.1.

IR (ATR): $\tilde{\nu}$ (cm^{-1}) = 2967, 2924, 2865, 2238, 1718, 1581, 1476, 1441, 1419, 1406, 1361, 1340, 1299, 1278, 1259, 1200, 1167, 1139, 1118, 1091, 1048, 1012.

HRMS (APCI, Q-TOF): calculated for $C_{19}H_{24}IO_3^+$ [M+H]$^+$: 427.0765, found 427.0762.

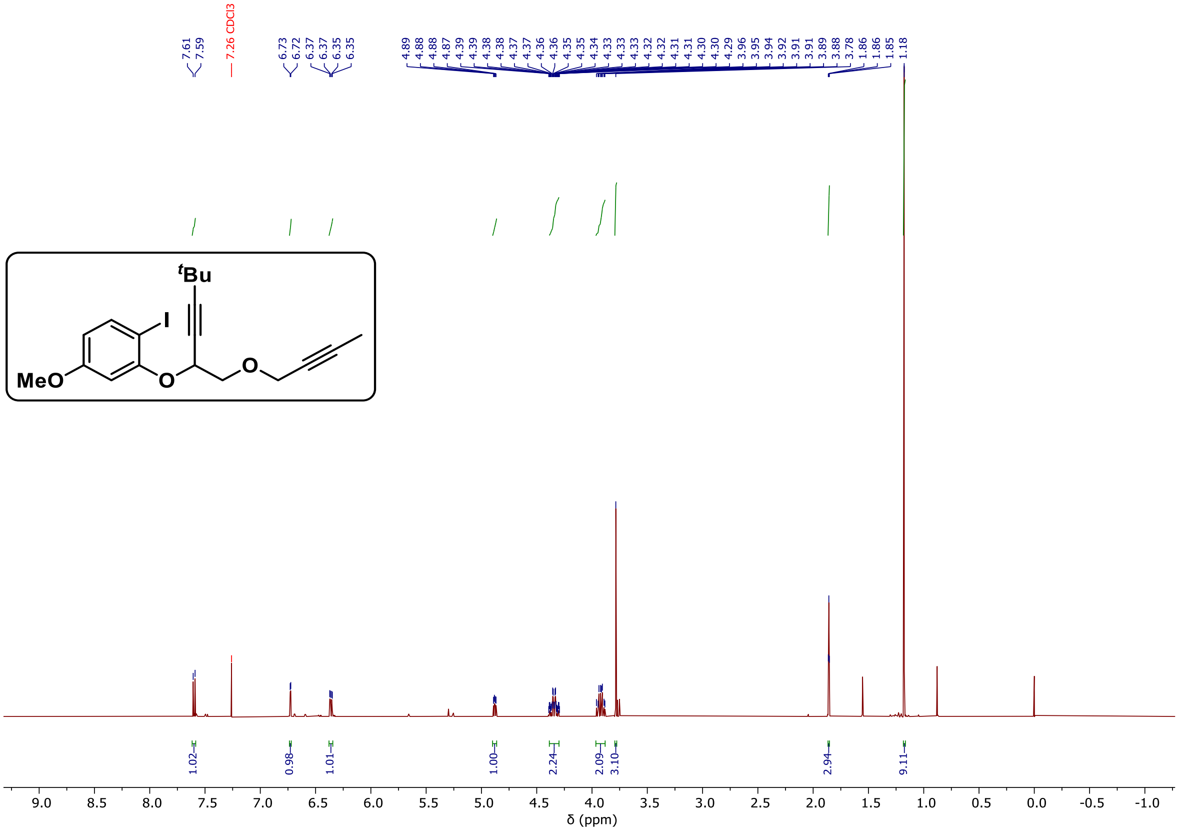

^{1}H-NMR (500 MHz, $CDCl_3$)

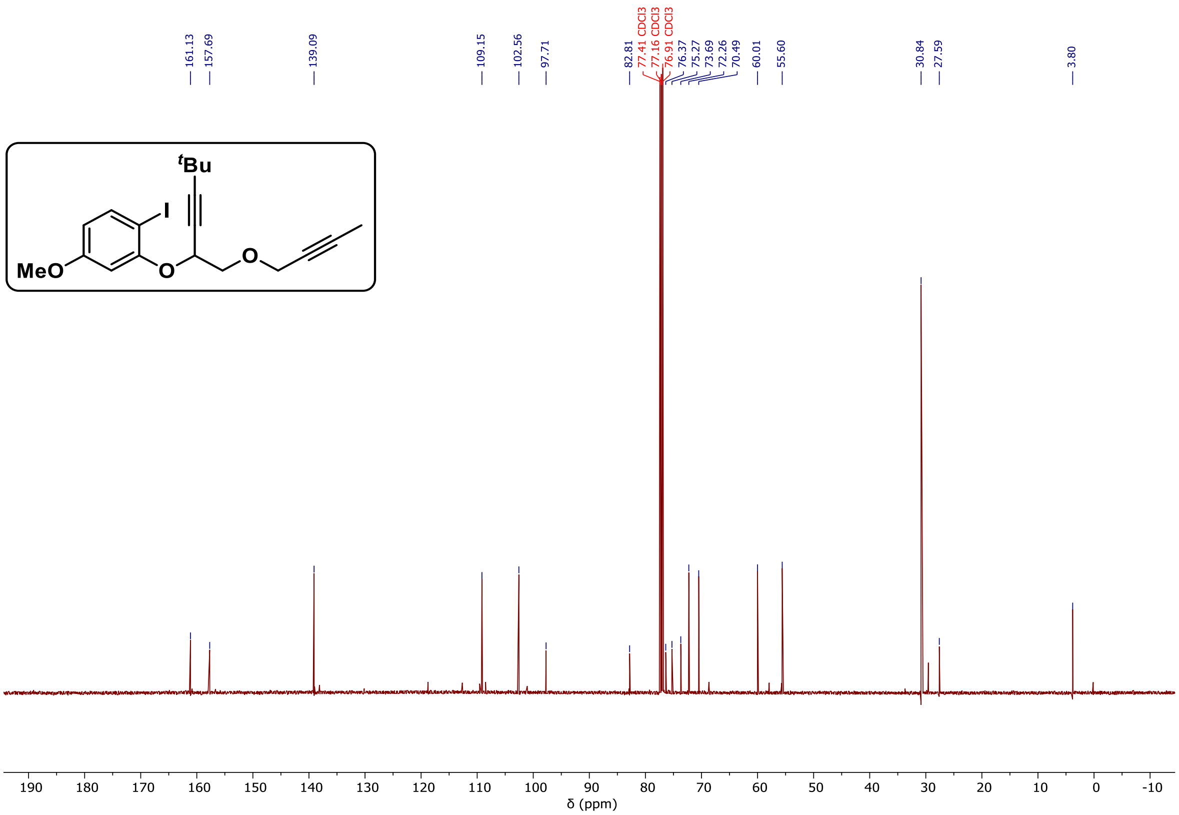

^{13}C-NMR (126 MHz, $CDCl_3$)

2-((3-bromonaphthalen-2-yl)oxy)-5,5-dimethylhex-3-yn-1-ol (S22)

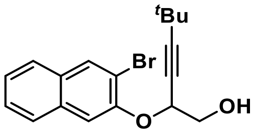

The protected alcohol (**S12**) (400 mg, 1.56 mmol, 1.0 eq.) in THF (15.5 mL, 0.1 M), 3-bromonaphtalen-2-ol (417.5 mg, 1.87 mmol, 1.2 eq.), PPh_3 (593.2 mg, 2.26 mmol, 1.4 eq.), and DIAD (504.6 mg, 490 µL, 2.50 mmol, 1.6 eq.) were reacted according to **GP3**. The reaction mixture was stirred at r.t. for 22 h. The crude product was used for the deprotection step without further purification. The TBS-protected **S17** crude was reacted with acetyl chloride (522 mg, 474 µL, 6.64 mmol, 4.3 eq.) in a mixture of CH_2Cl_2:MeOH (each 1.5 mL, 1.0 M) according **GP4**. The residue was purified by silica gel column chromatography (*n*-pentane:EtOAc = 10:1) to afford **S22** (204 mg, 0.59 mmol, 62%) as a colorless oil.

R_f = 0.36 (*n*-pentane:EtOAc = 10:1).

^{1}H-NMR (700 MHz, $CDCl_3$): δ = 8.07 (s, 1H), 7.74 – 7.70 (ddtd, *J* = 15.7, 8.2, 1.3, 0.6 Hz, 2H), 7.56 (s, 1H), 7.48 – 7.46 (ddd, *J* = 8.2, 6.9, 1.3 Hz, 1H), 7.40 – 7.38 (ddd, *J* = 8.1, 6.9, 1.2 Hz, 1H), 4.92 – 4.90 (dd, *J* = 8.2, 3.6 Hz, 1H), 4.06 – 4.03 (ddd, *J* = 12.2, 8.2, 4.1 Hz, 1H), 3.94 – 3.90 (ddd, *J* = 11.9, 10.0, 3.6 Hz, 1H), 2.44 – 2.42 (ddd, *J* = 10.0, 4.2, 1.7 Hz, 1H), 1.20 (s, 9H).

^{13}C-NMR (176 MHz, $CDCl_3$): δ = 27.7, 30.8, 65.7, 71.9, 73.2, 98.6, 111.7, 114.4, 125.1, 126.8, 126.9, 127.0, 130.2, 132.2, 133.5, 151.6.

IR (ATR): $\tilde{\nu}$ (cm^{-1}) = 2967, 2927, 1587, 1496, 1454, 1384, 1359, 1324, 1262, 1244, 1215, 1182, 1148, 1133, 1067, 1050, 1016, 1003.

HRMS (APCI, Q-TOF): calculated for $C_{18}H_{20}BrO_2^+$ $[M+H]^+$: 347.0641, found 347.0640.

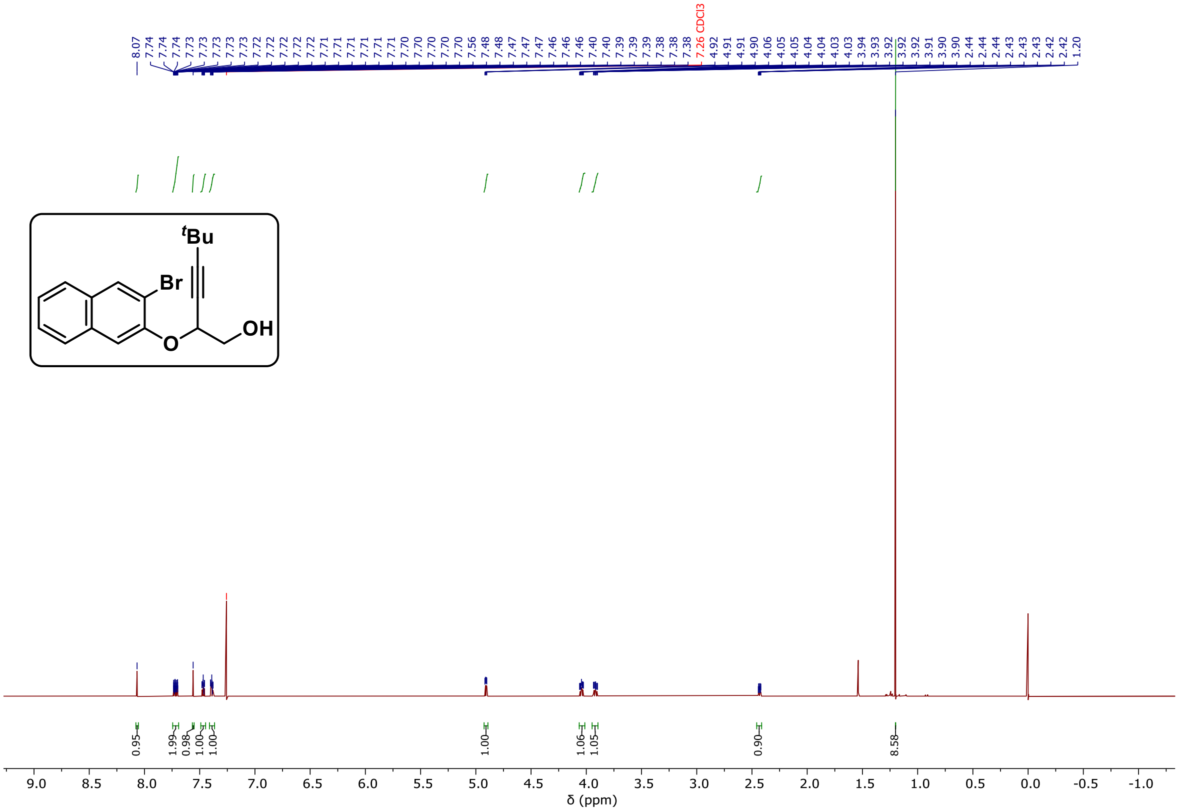

^{1}H-NMR (700 MHz, $CDCl_3$)

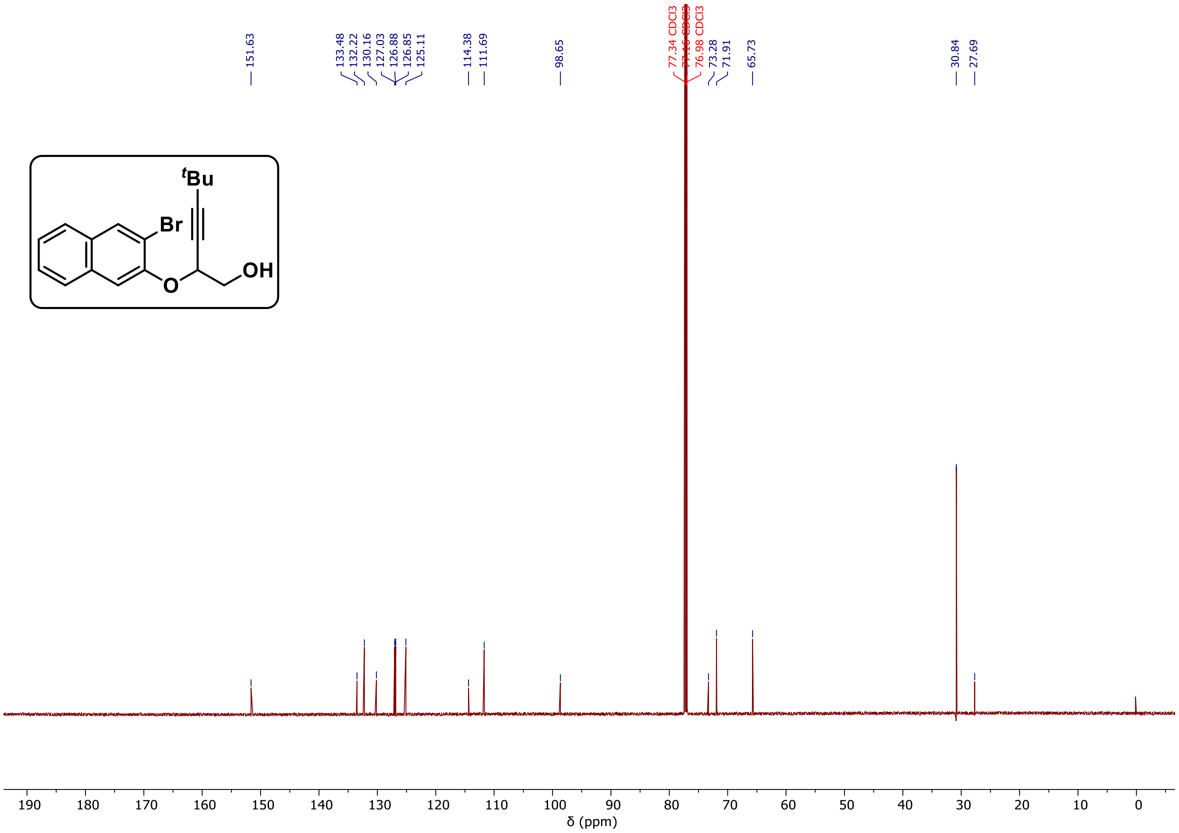

^{13}C-NMR (176 MHz, $CDCl_3$)

2-bromo-3-((1-(but-2-yn-1-yloxy)-5,5-dimethylhex-3-yn-2-yl)oxy)naphthalene (1s)

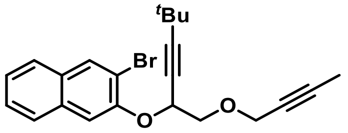

The alcohol **S22** (204 mg, 0.59 mmol, 1.0 eq.), 1-bromobut-2-yne (93.7 mg, 62 µL, 0.71 mmol, 1.2 eq.), TBAI (43.4 mg, 0.12 mmol, 20.0 mol%), and NaH (60% on mineral oil, 21.1 mg, 0.88 mmol, 1.5 eq.) in DMF (12 mL, 0.05 M) were reacted according to **GP2**. The reaction mixture was stirred at r.t. for 16 h. The crude product was purified using two silica gel column chromatography (*n*-pentane:EtOAc = 80:1 and *n*-pentane: CH_2Cl_2 = 2:1) to afford the domino precursor **1s** (141 mg, 0.35 mmol, 60%) as a colorless oil.

$\mathbf{R_f}$ = 0.33 (*n*-pentane:CH_2Cl_2 = 2:1).

^{1}H-NMR (700 MHz, $CDCl_3$): δ = 8.06 (s, 1H), 7.73 – 7.68 (m, 2H), 7.48 (s, 1H), 7.46 – 7.44 (ddd, *J* = 8.2, 6.8, 1.2 Hz, 1H), 7.38 – 7.36 (ddd, *J* = 8.1, 6.8, 1.2 Hz, 1H), 5.07 – 5.06 (dd, *J* = 7.6, 3.6 Hz, 1H), 4.42 – 4.34 (m, 2H), 4.04 – 3.97 (m, 2H), 1.86 (t, *J* = 2.3 Hz, 3H), 1.18 (s, 9H).

^{13}C-NMR (176 MHz, $CDCl_3$): δ = 3.8, 27.6, 30.8, 59.9, 70.3, 72.1, 73.6, 75.3, 82,8, 98.0, 111.2, 114.4, 124.8, 126.6, 126.8, 126.9, 129.9, 132.2, 133.4, 151.8.

IR (ATR): $\tilde{\nu}$ (cm^{-1}) = 1454, 1357, 1325, 1261, 1243, 1215, 1183, 1140, 1133, 1091, 1044, 1010.

HRMS (APCI, Q-TOF): calculated for $C_{22}H_{24}BrO_2^+$ $[M+H]^+$: 399.0954, found 399.0957.

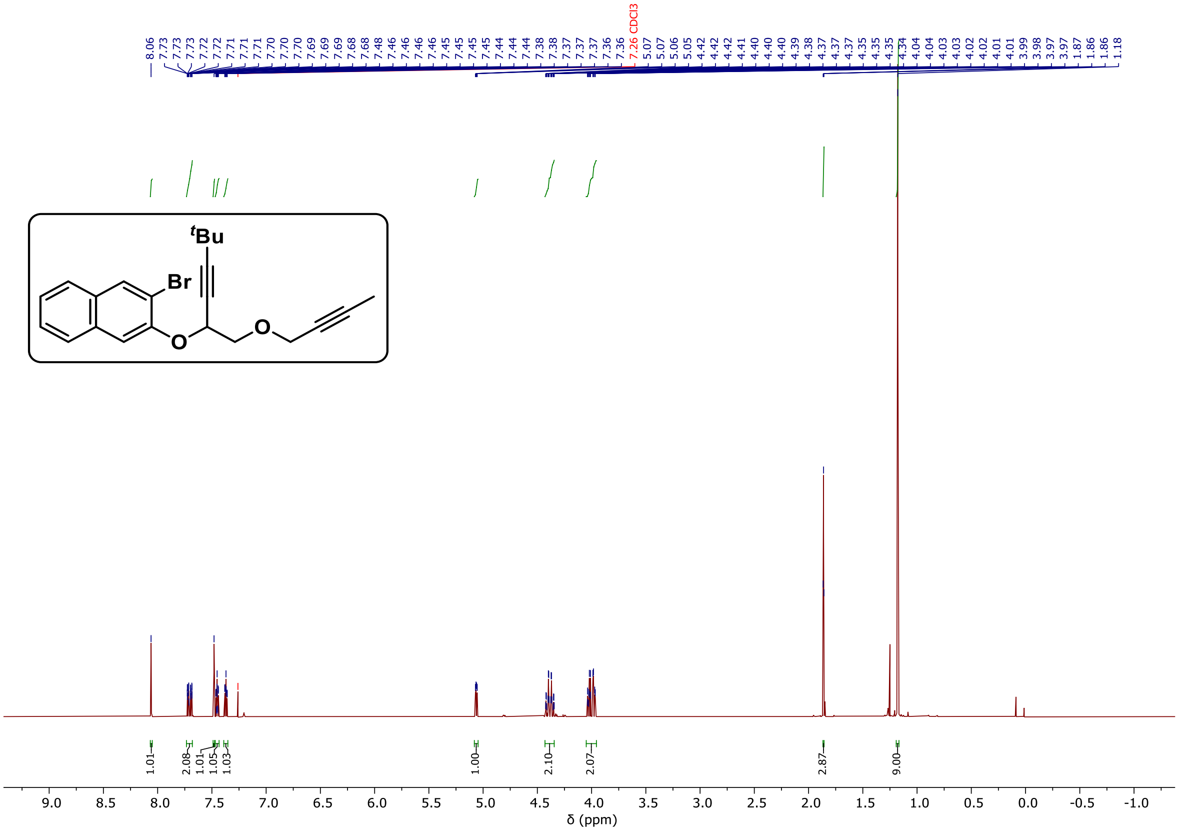

^{1}H-NMR (700 MHz, $CDCl_3$)

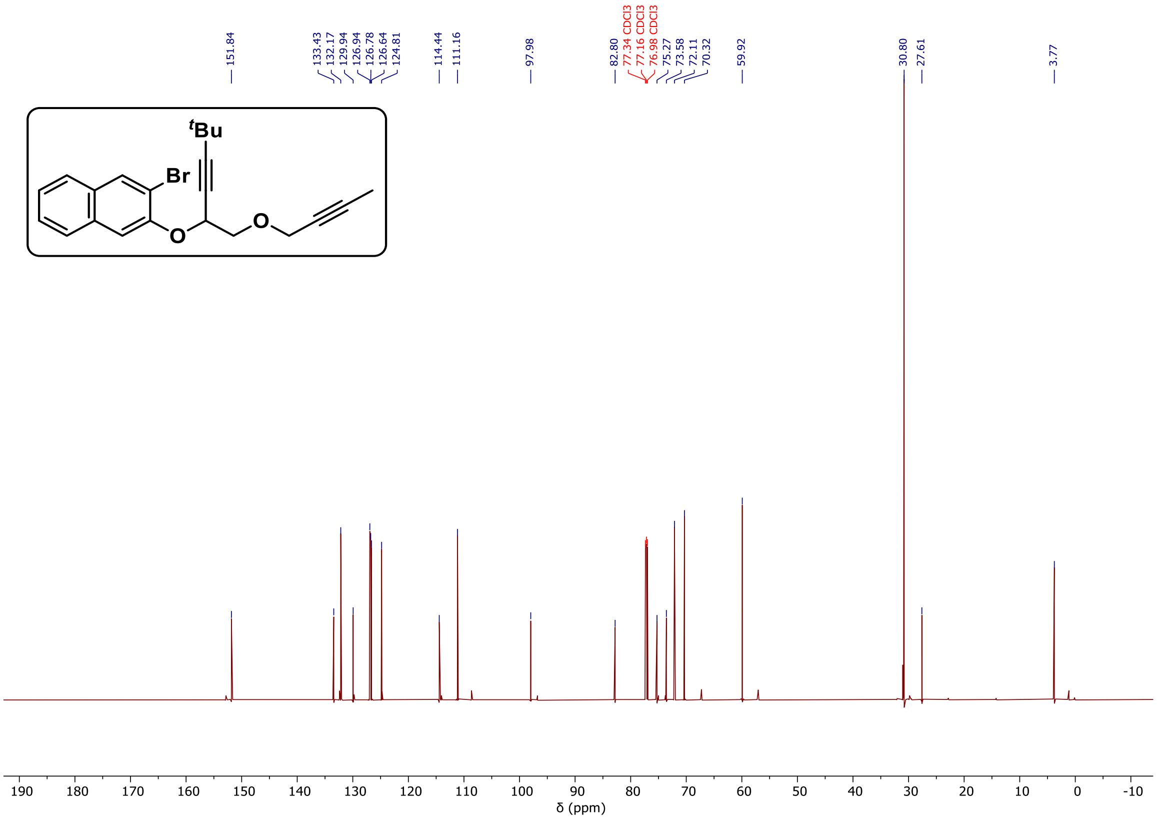

^{13}C-NMR (176 MHz, $CDCl_3$)

1-((5,5-dimethyl-1-(pent-2-yn-1-yloxy)hex-3-yn-2-yl)oxy)-2-iodobenzene (1t)

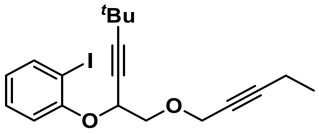

The alcohol **S18** (357 mg, 1.04 mmol, 1.0 eq.), 1-bromopent-2-yne (183 mg, 1.24 mmol, 1.2 eq.), TBAI (77 mg, 0.21 mmol, 20.0 mol%), and NaH (60% on mineral oil, 37.5 mg, 1.56 mmol, 1.5 eq.) in DMF (21 mL, 0.05 M) were reacted according to **GP2**. The reaction mixture was stirred at r.t. for 20 h. The crude product was purified using silica gel column chromatography (*n*-pentane:EtOAc = 80:1) to afford the domino precursor **1t** (186 mg, 0.45 mmol, 44%) as a colorless oil.

R_f = 0.36 (*n*-pentane:EtOAc = 80:1).

^{1}H-NMR (500 MHz, $CDCl_3$): δ = 7.77 – 7.75 (dd, *J* = 7.8, 1.6 Hz, 1H), 7.30 – 7.26 (dd, *J* = 6.9, 1.3 Hz, 1H), 7.09 – 7.07 (dd, *J* = 8.3, 1.4 Hz, 1H), 6.75 – 6.71 (m, 1H), 4.93 – 4.91 (dd, *J* = 7.4, 3.9 Hz, 1H), 4.41 – 4.32 (m, 2H), 3.97 – 3.89 (m, 2H), 2.26 – 2.21 (qt, *J* = 7.5, 2.2 Hz, 2H), 1.17 – 1.15 (t, *J* = 7.6 Hz, 3H, overlapped with 1.16), 1.16 (s, 9H).

^{13}C-NMR (126 MHz, $CDCl_3$): δ = 12.6, 13.9, 27.6, 30.8, 60.0, 70.5, 72.3, 73.7, 75.4, 87.9, 88.7, 97.6, 115.4, 123.4, 129.2, 139.5, 156.9.

IR (ATR): $\tilde{\nu}$ (cm^{-1}) = 2969, 2929, 2867, 2237, 1580, 1571, 1469, 1439, 1391, 1361, 1340, 1319, 1276, 1262, 1236, 1204, 1140, 1120, 1092, 1044, 1018.

HRMS (APCI, Q-TOF): calculated for $C_{19}H_{24}IO_2^+$ $[M+H]^+$: 411.0815, found 411.0822.

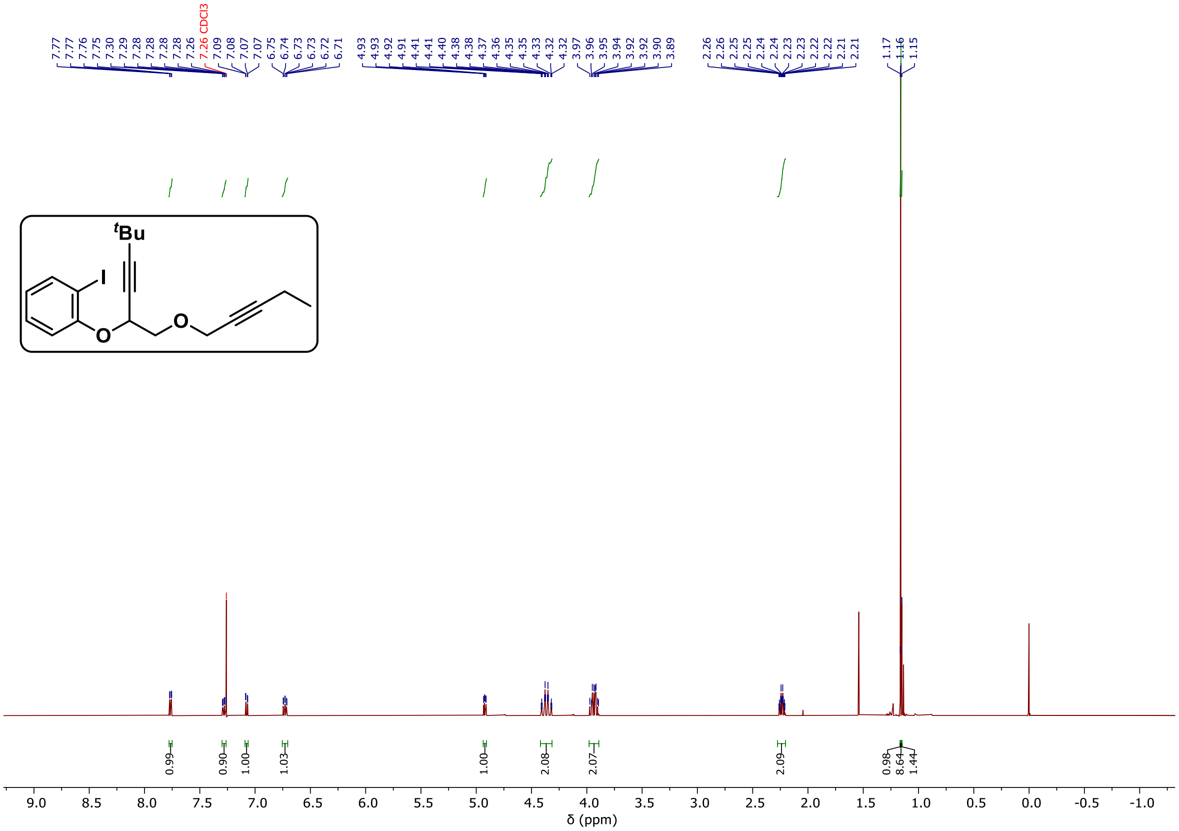

^{1}H-NMR (500 MHz, $CDCl_3$)

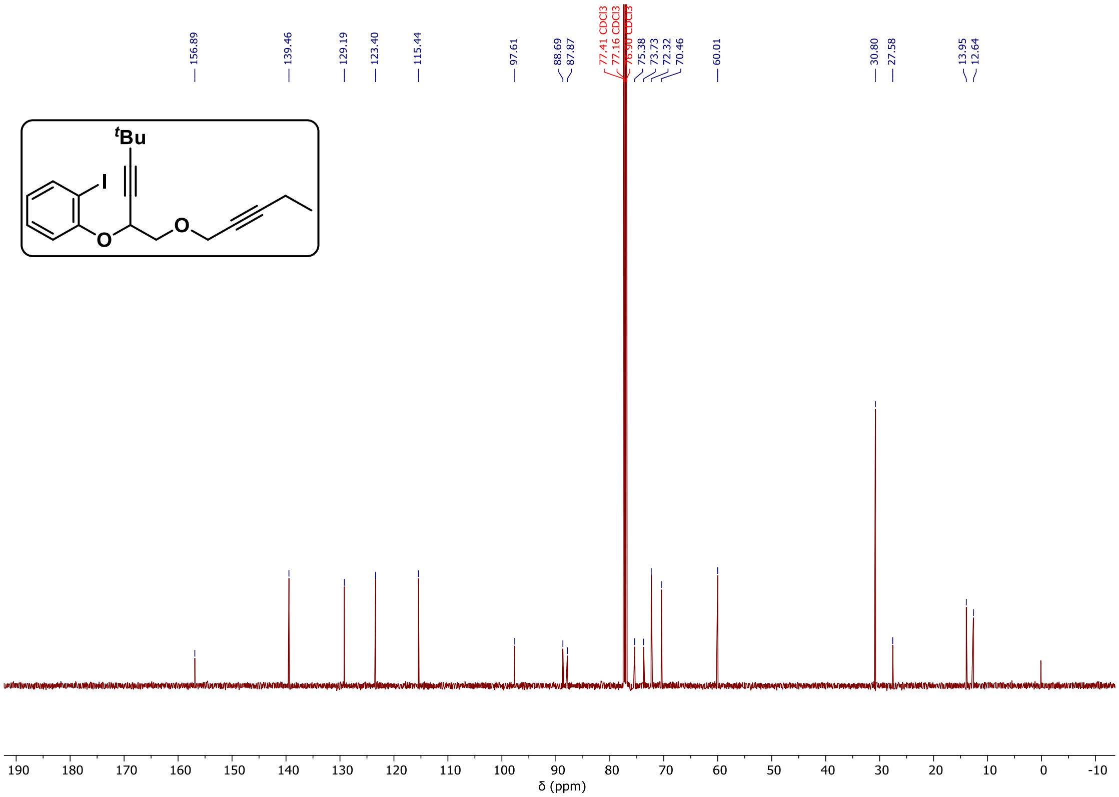

^{13}C-NMR (126 MHz, $CDCl_3$)

1-((5,5-dimethyl-1-((4-methylpent-2-yn-1-yl)oxy)hex-3-yn-2-yl)oxy)-2-iodobenzene (1u)

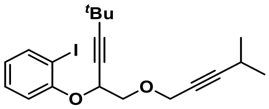

The alcohol **S18** (357 mg, 1.04 mmol, 1.0 eq.), 1-bromo-4-methylpent-2-yne (200.5 mg, 1.24 mmol, 1.2 eq.), TBAI (76.6 mg, 0.21 mmol, 20.0 mol%), and NaH (60% on mineral oil, 37.5 mg, 1.56 mmol, 1.5 eq.) in DMF (21 mL, 0.05 M) were reacted according to **GP2**. The reaction mixture was stirred at r.t. for 17 h. The crude product was purified using silica gel column chromatography (*n*-pentane:EtOAc = 80:1) to afford the domino precursor **1u** (173 mg, 0.41 mmol, 39 %) as a colorless oil.

R_f = 0.36 (*n*-pentane:EtOAc = 80:1).

^{1}H-NMR (700 MHz, $CDCl_3$): δ = 7.77 – 7.76 (dd, *J* = 7.8, 1.6 Hz, 1H), 7.29 – 7.26 (ddd, *J* = 8.2, 7.3, 1.6 Hz, 1H), 7.09 – 7.07 (dd, *J* = 8.3, 1.4 Hz, 1H), 6.74 – 6.72 (m, 1H), 4.93 – 4.92 (dd, *J* = 7.5, 3.8 Hz, 1H), 4.40 – 4.33 (m, 2H), 3.96 – 3.90 (m, 2H), 2.63 – 2.57 (dtt, *J* = 13.8, 6.9, 2.0 Hz, 1H), 1.18 (s, 3H), 1.17 (s, 3H), 1.16 (s, 9H).

^{13}C-NMR (176 MHz, $CDCl_3$): δ = 20.7, 23.0, 27.6, 29.5, 30.8, 60.0, 70.5, 72.3, 73.8, 75.2, 87.9, 92.8, 97.6, 115.5, 123.4, 129.2, 139.5, 156.9.

IR (ATR): $\tilde{\nu}$ (cm^{-1}) = 2968, 2928, 2868, 1580, 1571, 1469, 1439, 1361, 1341, 1319, 1276, 1263, 1237, 1204, 1184, 1162, 1127, 1097, 1044, 1018, 1007.

HRMS (APCI, Q-TOF): calculated for $C_{20}H_{26}IO_2^+$ $[M+H]^+$: 425.0972, found 425.0978.

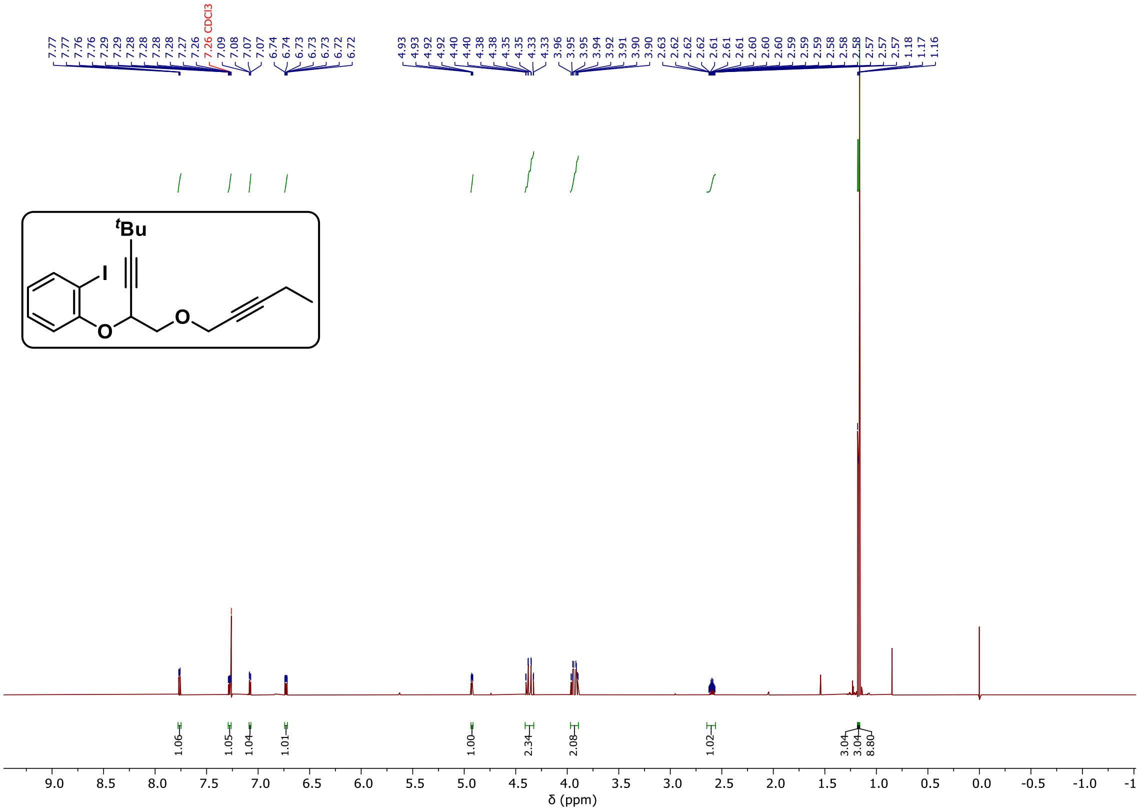

^{1}H-NMR (700 MHz, $CDCl_3$)

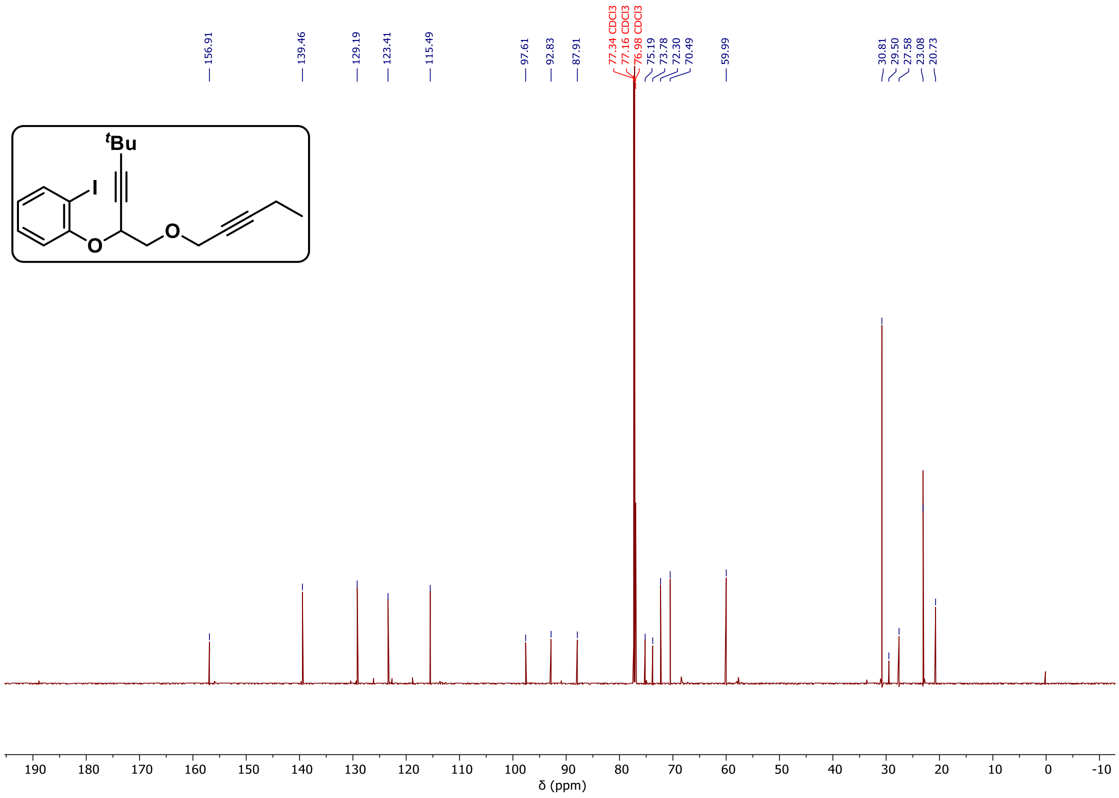

^{13}C-NMR (176 MHz, $CDCl_3$)

5. The Formation and Characterization of Cascade Products

Unsuccessful Examples

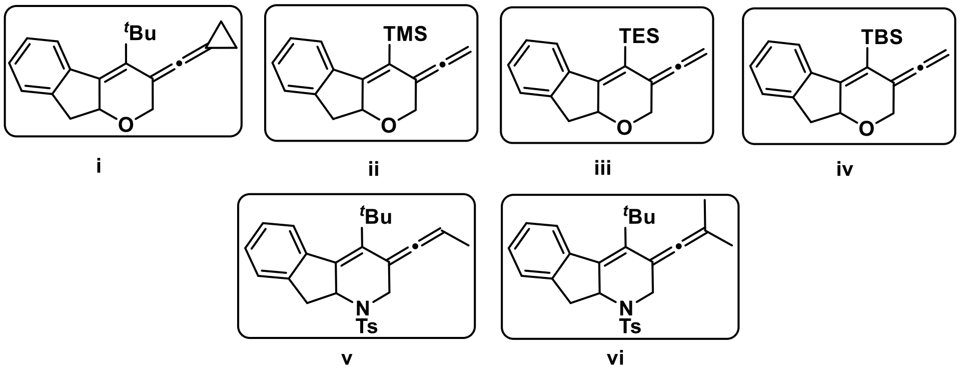

i, ii, iii, and iv: no allenes were formed.

v and vi: not easy to isolate the desired allenes.

4-(*tert*-butyl)-3-vinylidene-2,3,9,9a-tetrahydroindeno[2,1-*b*]pyran (2a)

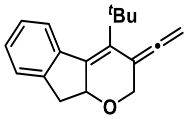

Domino precursor **1a** (35 mg, 0.10 mmol, 1.0 eq.), $Pd_2(dba)_3$ (4.6 mg, 5 µmol, 5.0 mol%), *tert*-Bu-DavePhos (3.4 mg, 10 µmol, 10.0 mol%), and KOAc (73.5 mg, 0.75 mmol, 7.5 eq.) in DMF (8 mL, 12 mM) were reacted according to **GP5**. Flash column chromatography (*n*-pentane:EtOAc = 40:1) afforded allene product **2a** (16.4 mg, 65 µmol, 62%) as yellow oil.

In the case of employing **1a'**, the domino product **2a** is obtained in 71% yield.

Large scale synthesis:

Domino precursor **1a'** (350 mg, 1.0 mmol, 1.0 eq.), $Pd_2(dba)_3$ (45.5 mg, 50 µmol, 5.0 mol%), *tert*-Bu-DavePhos (34 mg, 100 µmol, 10.0 mol%), and KOAc (0.73 g, 7.5 mmol, 7.5 eq.) in DMF (80 mL, 12 mM) were reacted according to **GP5**. Flash column chromatography (*n*-pentane:EtOAc = 40:1) afforded allene product **2a** (168.9 mg, 0.67 mmol, 67%) as yellow oil.

R_f = 0.28 (*n*-pentane:EtOAc = 40:1).

^{1}H-NMR (400 MHz, $CDCl_3$): δ = 7.62 – 7.58 (m, 1H), 7.24 – 7.16 (m, 3H), 4.99 (qd, *J* = 3.8, 1.1 Hz, 2H), 4.56 – 4.47 (m, 2H), 4.36 (dtd, *J* = 12.4, 3.5, 0.5 Hz, 1H), 3.19 (ddt, *J* = 15.2, 7.9, 0.6 Hz, 1H), 2.98 (ddt, *J* = 15.2, 7.8, 1.0 Hz, 1H), 1.42 (s, 9H).

^{13}C-NMR (101 MHz, $CDCl_3$): δ = 205.4, 143.5, 141.9, 138.5, 135.9, 128.2, 128.0, 126.1, 125.0, 99.4, 82.5, 79.1, 67.6, 37.9, 36.6, 30.9.

IR (ATR): $\tilde{\nu}$ (cm^{-1}) = 3414, 5958, 2923, 2862, 1936, 1716, 1667, 1603, 1462, 1395, 1363, 1267, 1200, 1124, 1029.

HRMS (APCI, Q-TOF): calculated for $C_{18}H_{21}O^+$ $[M+H]^+$: 253.1587, found: 253.1587.

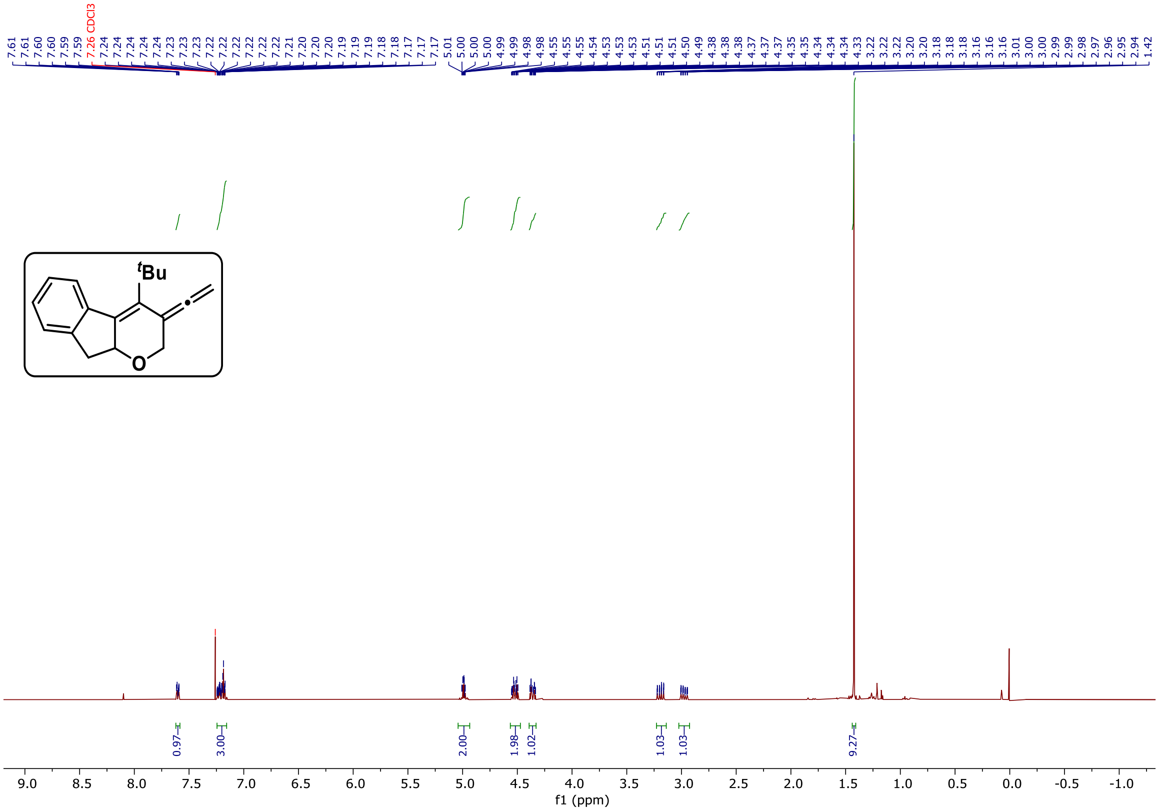

^{1}H-NMR (400 MHz, $CDCl_3$)

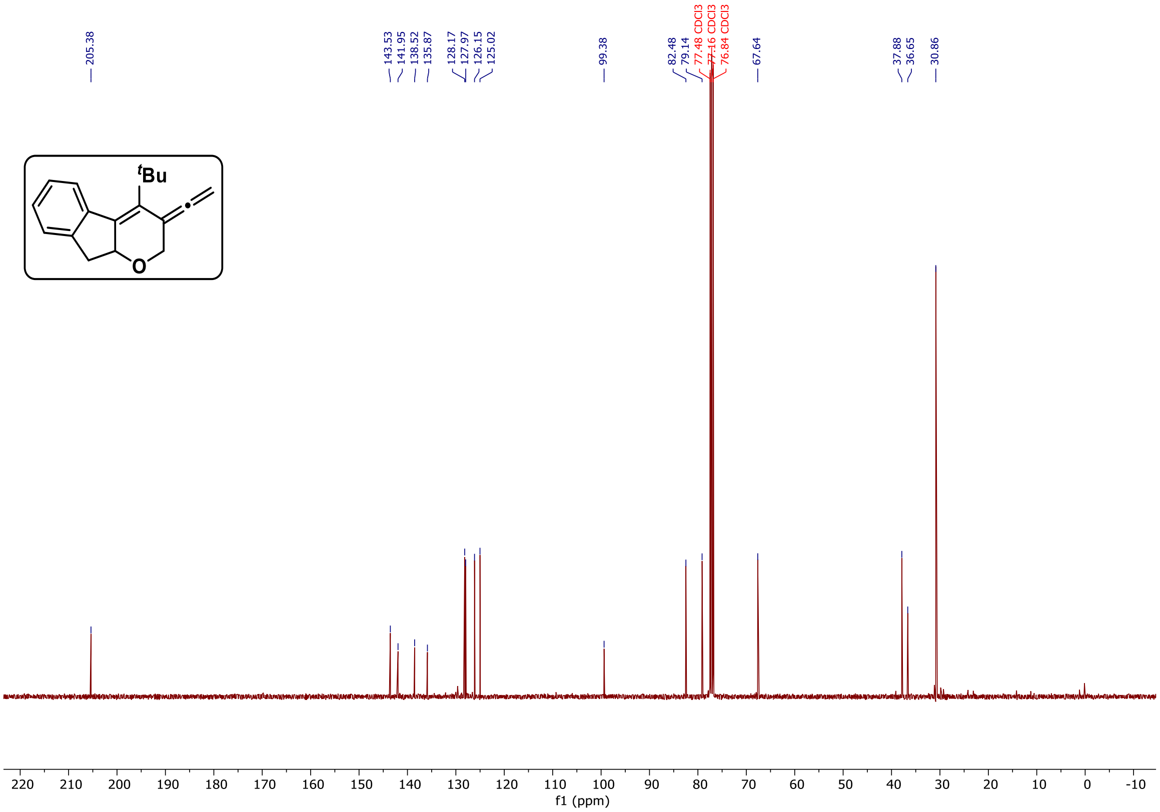

^{13}C-NMR (101 MHz, $CDCl_3$)

4-(*tert*-butyl)-3-((*R*/*S*)-prop-1-en-1-ylidene)-2,3,9,9a-tetrahydroindeno[2,1-*b*]pyran (2b)

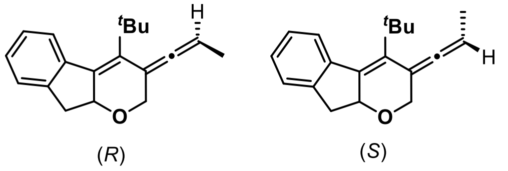

Domino precursor **1b** (35 mg, 0.10 mmol, 1.0 eq.), $Pd_2(dba)_3$ (4.6 mg, 5 µmol, 5.0 mol%), *tert*-Bu-DavePhos (3.4 mg, 10 µmol, 10.0 mol%), and KOAc (74.2 mg, 0.75 mmol, 7.5 eq.) in DMF (8 mL, 12 mM) were reacted according to **GP5**. Flash column chromatography (*n*-pentane:EtOAc = 80:1 and *n*-pentane:toluene = 1:1) afforded allene product **2b** in the ratio of 54% to 46% (17.4 mg, 65 µmol, 65%) as yellow oil.

$\mathbf{R_f}$ = 0.22 (*n*-pentane:EtOAc = 80:1).

^{1}H-NMR (500 MHz, $CDCl_3$): δ = 7.61 – 7.57 (m, 1H), 7.24 – 7.15 (m, 3H), 5.38 – 5.28 (m, 1H), 4.56 – 4.51 (m, 1H), 4.51 – 4.44 (m, 1H), 4.37 – 4.29 (m, 1H), 3.18 (ddd, *J* = 15.0, 8.0, 0.7 Hz, 1H), 2.97 (dddt, *J* = 14.9, 7.8, 2.1, 1.0 Hz, 1H), 1.73 (d, *J* = 7.1 Hz, 2H), 1.69 (d, *J* = 7.0 Hz, 1H), 1.40 (s, 5H), 1.40 (s, 4H).

^{13}C-NMR (126 MHz, $CDCl_3$): δ = 201.5, 200.9, 143.4, 143.3, 141.4, 141.3, 138.7, 138.7, 136.7, 136.7, 128.2, 128.1, 127.7, 126.0, 126.0, 124.9, 99.4, 99.0, 89.9, 89.0, 82.5, 82.5, 67.9, 37.9, 37.8, 36.6, 35.8, 30.8, 30.8, 14.5, 14.4.

IR (ATR): $\tilde{\nu}$ (cm^{-1}) = 2964, 2929, 1716, 1606, 1460, 1366, 1195, 1150, 1074, 1024.

HRMS (APCI, Q-TOF): calculated for $C_{19}H_{22}O^+$ $[M]^+$: 266.1665, found: 266.1667.

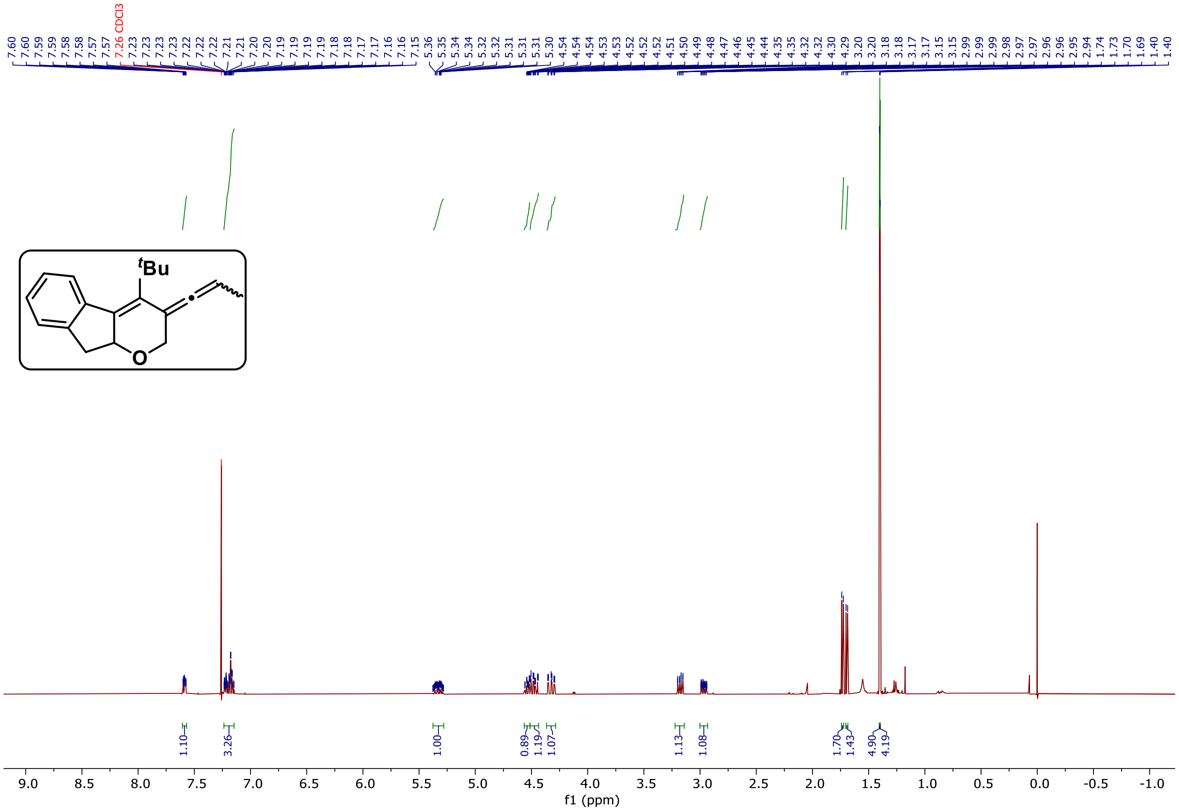

^{1}H-NMR (500 MHz, $CDCl_3$)

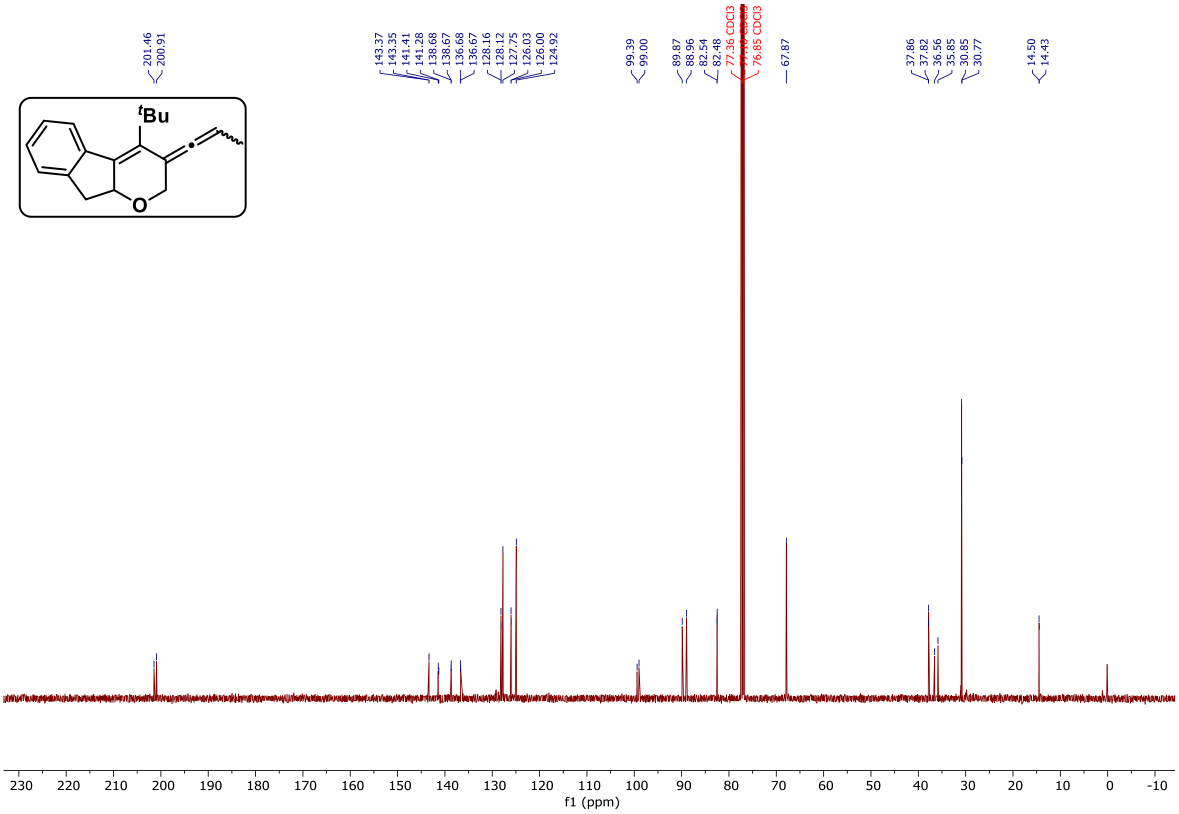

^{13}C-NMR (126 MHz, $CDCl_3$)

4-(*tert*-butyl)-3-(2-methylprop-1-en-1-ylidene)-2,3,9,9a-tetrahydroindeno[2,1-*b*]pyran (2c)

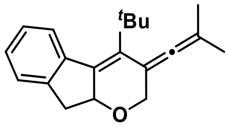

Domino precursor **1c** (36 mg, 0.10 mmol, 1.0 eq.), $Pd_2(dba)_3$ (4.5 mg, 5 µmol, 5.0 mol%), *tert*-Bu-DavePhos (3.4 mg, 10 µmol, 10.0 mol%), and KOAc (73.3 mg, 0.75 mmol, 7.5 eq.) in DMF (8 mL, 12 mM) were reacted according to **GP5**. Flash column chromatography (*n*-pentane:EtOAc = 60:1 to 20:1) afforded allene product **2c** (13.2 mg, 47 µmol, 47%) as brown oil.

R$_f$ = 0.29 (*n*-pentane:EtOAc = 60:1).

^{1}H-NMR (500 MHz, $CDCl_3$): δ = 7.52 (dq, *J* = 6.8, 0.9 Hz, 1H), 7.23 – 7.12 (m, 3H), 5.59 (hept, *J* = 1.4 Hz, 1H), 4.66 – 4.61 (m, 1H), 3.34 (ddd, *J* = 16.5, 1.8, 0.9 Hz, 1H), 3.22 (ddd, *J* = 10.0, 6.2, 1.9 Hz, 1H), 3.09 (ddt, *J* = 16.2, 9.9, 1.1 Hz, 1H), 1.83 (d, *J* = 1.5 Hz, 3H), 1.41 (s, 9H), 1.37 (d, *J* = 1.2 Hz, 3H).

^{13}C-NMR (126 MHz, $CDCl_3$): δ = 210.1, 182.9, 143.5, 143.1, 138.9, 138.4, 127.4, 126.4, 126.1, 125.8, 116.5, 53.1, 51.2, 35.9, 32.4, 29.6, 25.6, 20.3.

IR (ATR): $\tilde{\nu}$ (cm^{-1}) = 2962, 2918, 1701, 1477, 1446, 1396, 1376, 1364, 1320, 1306, 1228, 1188, 1103, 1085, 1028, 978,

HRMS (APCI, Q-TOF): calculated for $C_{20}H_{25}O^+$ $[M+H]^+$: 281.1900, found: 281.1900.

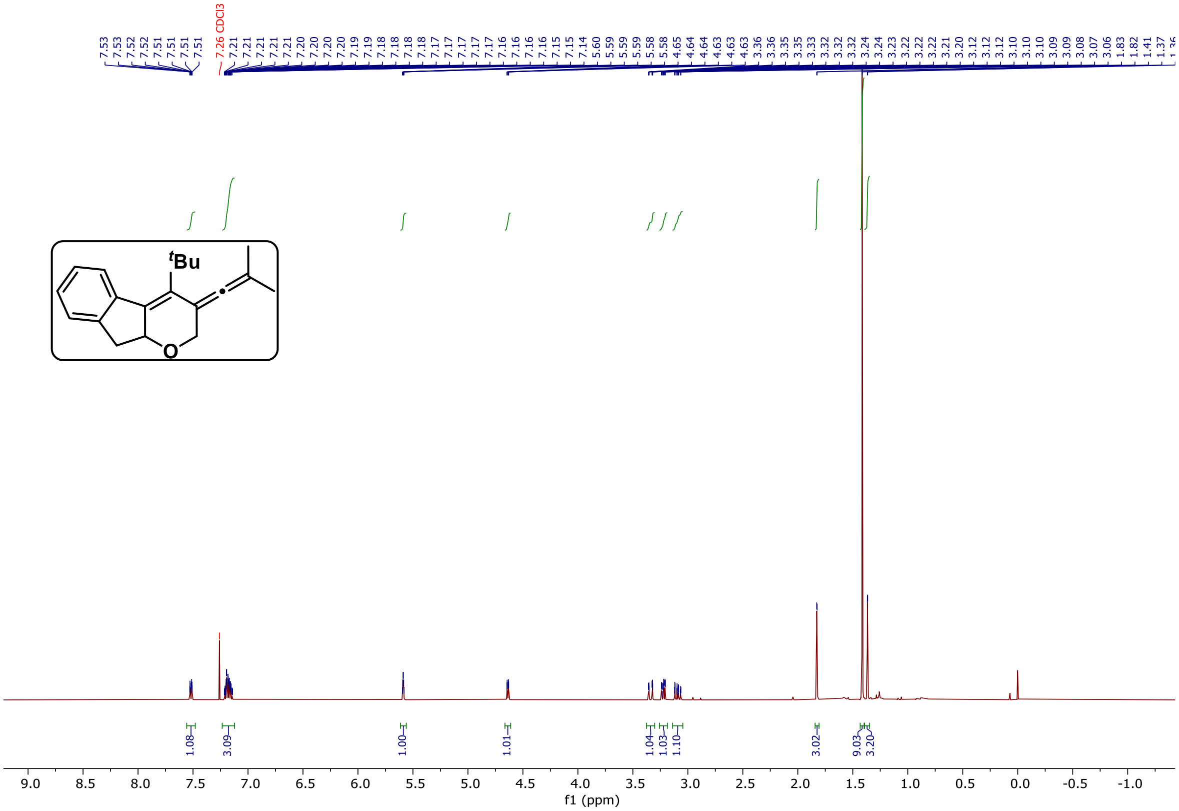

^{1}H-NMR (500 MHz, $CDCl_3$)

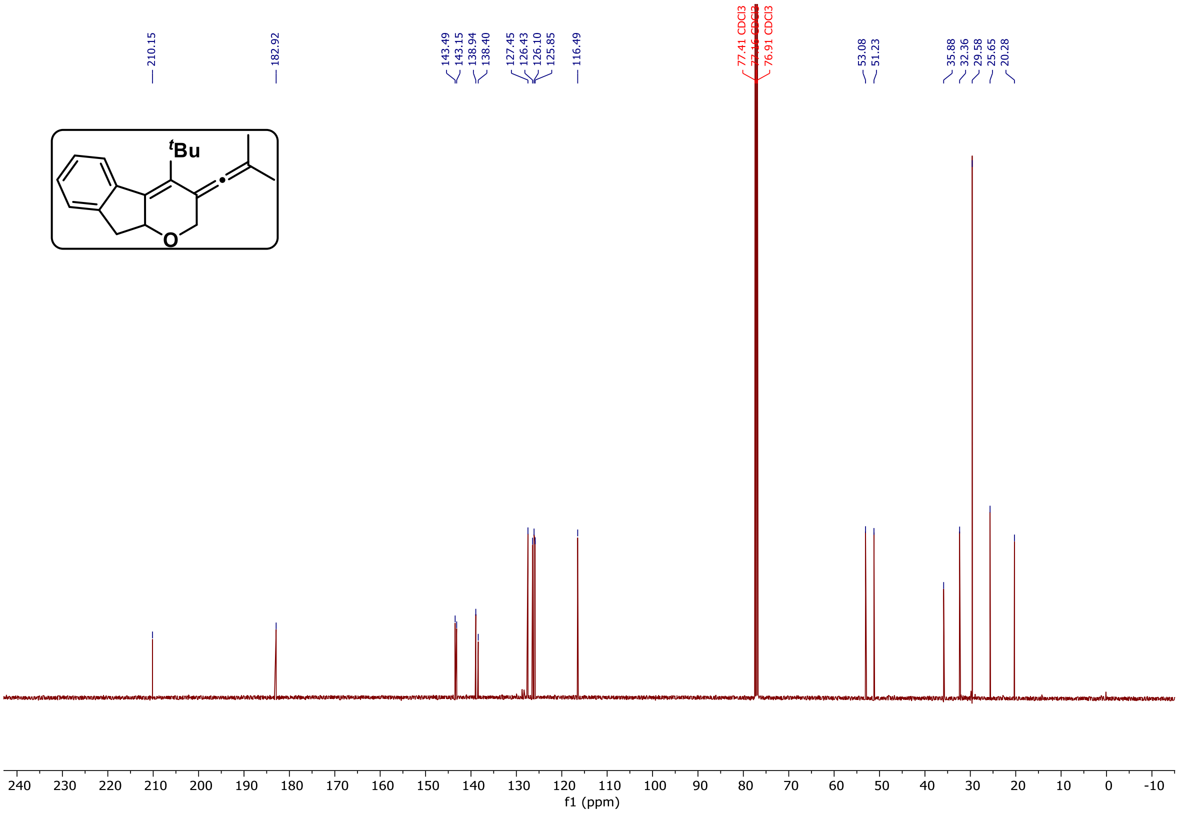

^{13}C-NMR (126 MHz, $CDCl_3$)

4-(*tert*-butyl)-1-tosyl-3-vinylidene-2,3,9,9a-tetrahydro-1*H*-indeno[2,1-*b*]pyridine (2d)

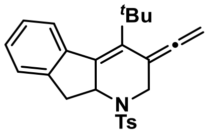

Domino precursor **1d** (52.5 mg, 0.10 mmol, 1.0 eq.), $Pd_2(dba)_3$ (4.5 mg, 5 µmol, 5.0 mol%), *tert*-Bu-DavePhos (3.3 mg, 10 µmol, 10.0 mol%), and KOAc (72.4 mg, 0.74 mmol, 7.5 eq.) in DMF (8 mL, 12 mM) were reacted according to **GP5**. Flash column chromatography (*n*-pentane:EtOAc = 90:1 to 60:1) afforded allene product **2d** (25.6 mg, 61 µmol, 62%) as yellow oil.

$\mathbf{R_f}$ = 0.34 (*n*-pentane:EtOAc = 60:1).

^{1}H-NMR (700 MHz, $CDCl_3$): δ = 7.70 – 7.66 (m, 2H), 7.60 – 7.57 (m, 1H), 7.28 – 7.21 (m, 3H), 7.21 – 7.14 (m, 2H), 4.77 (ddt, J = 11.4, 2.6, 0.8 Hz, 1H), 4.48 (ddt, J = 11.3, 2.1, 0.8 Hz, 1H), 4.20 – 4.12 (m, 2H), 3.88 (dt, J = 13.2, 2.4 Hz, 1H), 3.42 (dd, J = 14.9, 7.4 Hz, 1H), 3.12 (ddt, J = 14.9, 9.1, 1.2 Hz, 1H), 2.40 (s, 3H), 1.29 (s, 9H).

^{13}C-NMR (176 MHz, $CDCl_3$): δ = 207.2, 143.5, 143.2, 138.3, 138.2, 135.8, 135.7, 129.7, 128.2, 128.2, 125.8, 125.0, 96.5, 77.3, 62.6, 50.1, 40.9, 36.9, 30.9, 21.7.

IR (ATR): $\tilde{v}$ (cm^{-1}) = 2957, 2922, 1455, 1347, 1307, 1290, 1260, 1187, 1160, 1092, 1026.

HRMS (APCI, Q-TOF): calculated for $C_{25}H_{27}O_2NNaS^+$ [M+Na]$^+$: 428.1655, found: 428.1660.

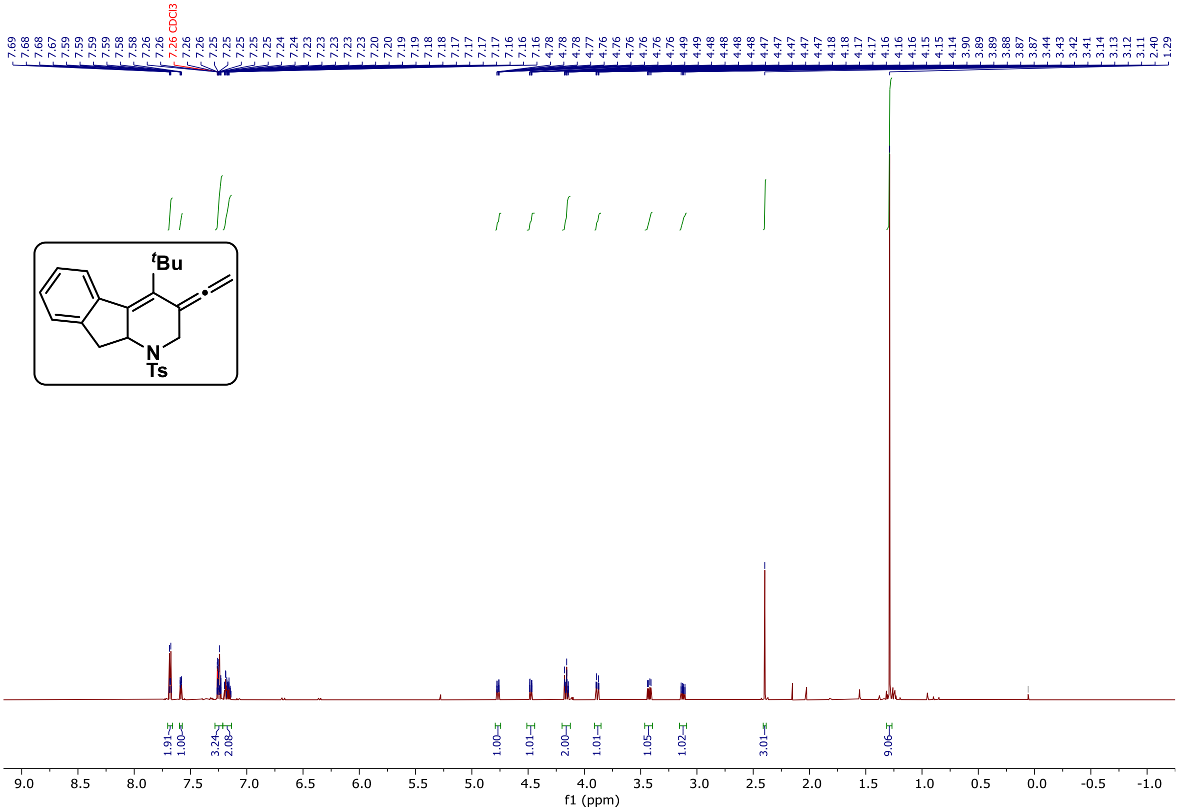

¹H-NMR (700 MHz, $CDCl_3$)

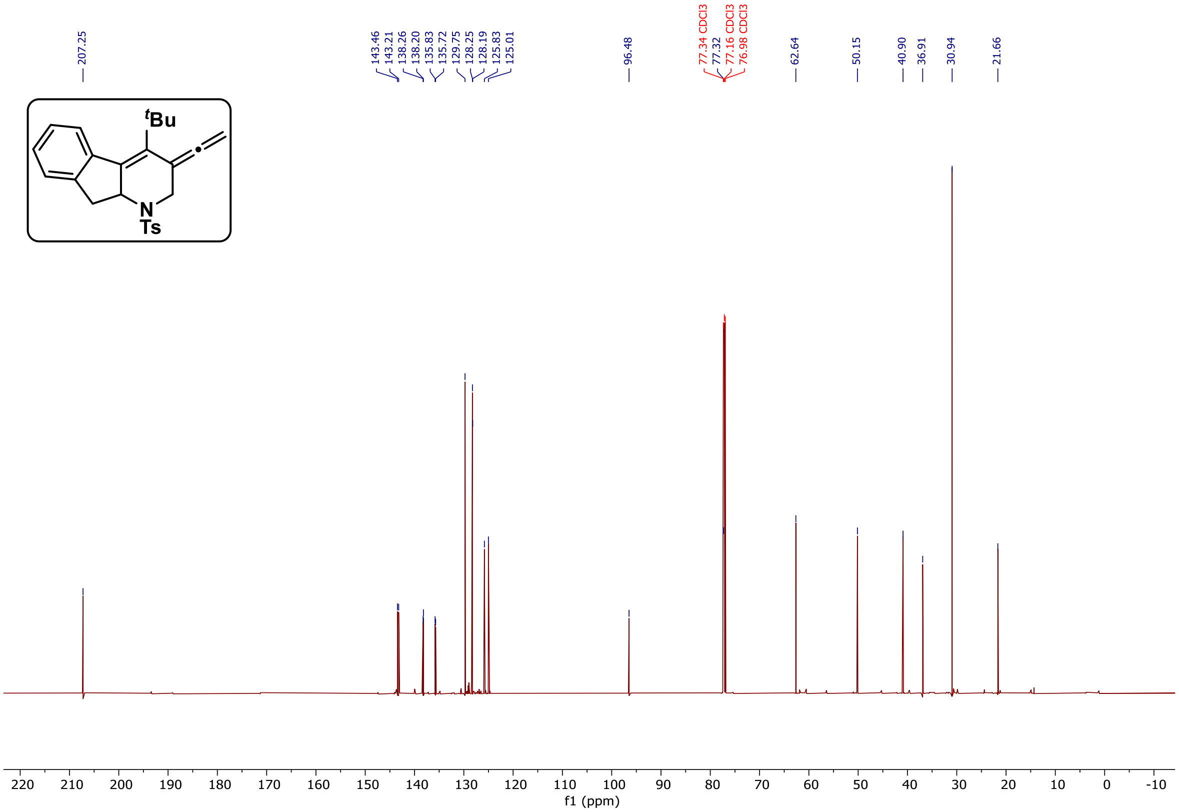

¹³C-NMR (176 MHz, $CDCl_3$)

1-(*tert*-butyl)-2-vinylidene-2,3,4,4a-tetrahydrodibenzo[*b*,*d*]furan (2e)

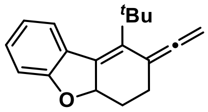

Domino precursor **1e** (39.2 mg, 0.10 mmol, 1.0 eq.), $Pd_2(dba)_3$ (4.7 mg, 5 µmol, 5.0 mol%), *tert*-Bu-DavePhos (3.5 mg, 10 µmol, 10.0 mol%), and KOAc (73.9 mg, 0.75 mmol, 7.5 eq.) in DMF (8 mL, 12 mM) were reacted according to **GP5**. Flash column chromatography (*n*-pentane:EtOAc = 20:1 and *n*-pentane:CH_2Cl_2 = 10:1) afforded allene product **2e** (16.9 mg, 67 µmol, 65%) as yellow oil.

R$_f$ = 0.42 (*n*-pentane:EtOAc = 30:1).

^{1}H-NMR (500 MHz, $CDCl_3$): δ = 7.67 (dd, J = 7.9, 1.3 Hz, 1H), 7.16 (ddd, J = 8.1, 7.4, 1.3 Hz, 1H), 6.91 (td, J = 7.6, 1.1 Hz, 1H), 6.88 – 6.83 (m, 1H), 4.82 (dd, J = 10.4, 4.9 Hz, 1H), 4.80 – 4.75 (m, 2H), 2.52 – 2.45 (m, 1H), 2.31 – 2.22 (m, 2H), 1.45 – 1.41 (m, 1H), 1.39 (s, 9H).

^{13}C-NMR (126 MHz, $CDCl_3$): δ = 207.3, 164.8, 136.4, 134.9, 129.3, 127.7, 123.7, 120.3, 110.4, 99.7, 84.7, 76.0, 35.9, 29.9, 27.1, 22.3.

IR (ATR): $\tilde{\nu}$ (cm^{-1}) = 1459, 1451, 1364, 1326, 1314, 1260, 1220, 1167, 1150, 1101, 1049, 1031, 1018, 963.

HRMS (APCI, Q-TOF): calculated for $C_{18}H_{21}O^+$ $[M+H]^+$: 253.1587, found: 253.1587.

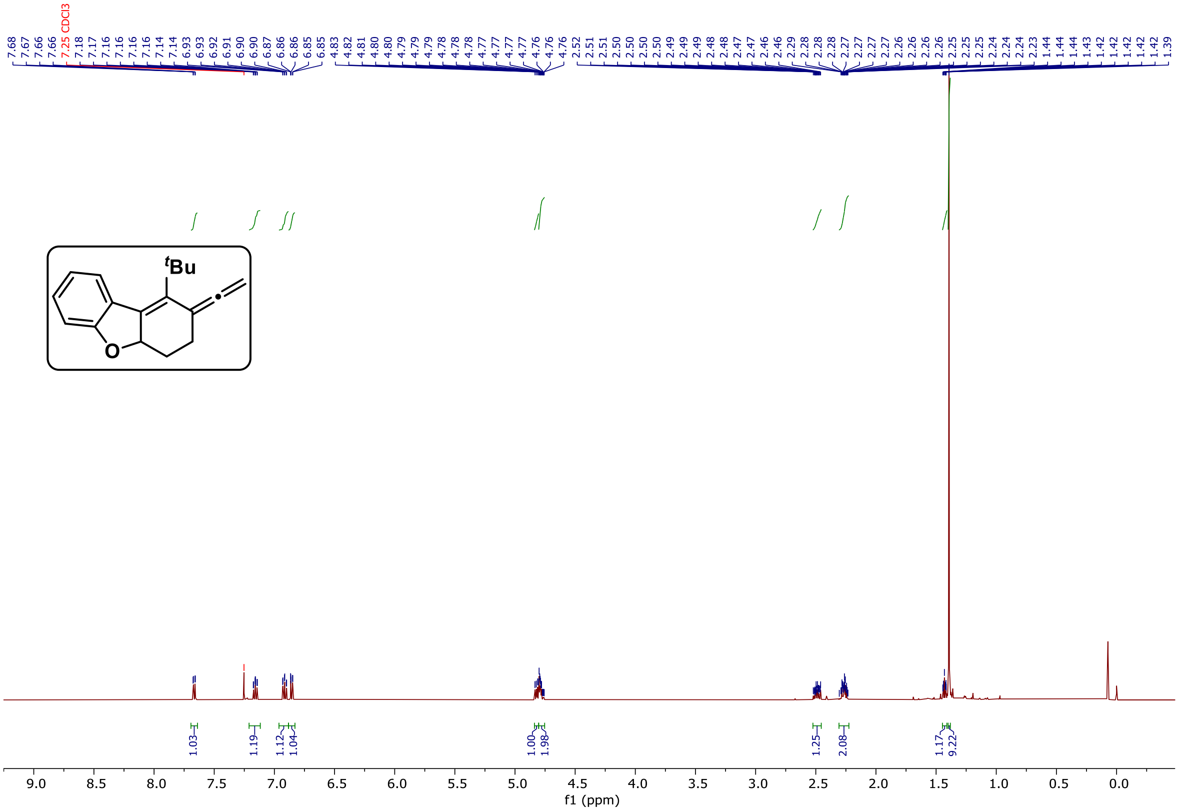

^{1}H-NMR (500 MHz, $CDCl_3$)

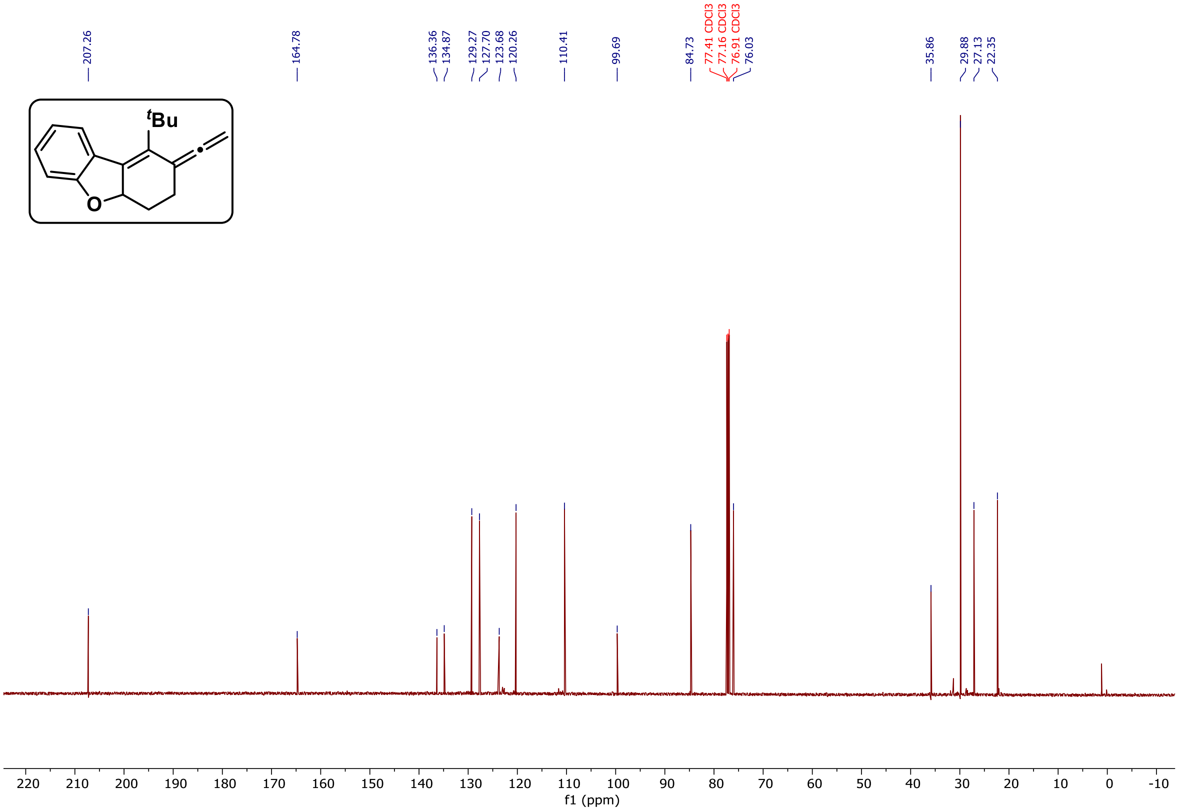

^{13}C-NMR (126 MHz, $CDCl_3$)

1,8-di-*tert*-butyl-2-vinylidene-2,3,4,4a-tetrahydrodibenzo[*b*,*d*]furan (2f)

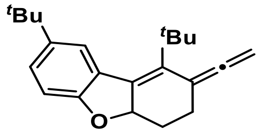

Domino precursor **1f** (49.5 mg, 0.11 mmol, 1.0 eq.), $Pd_2(dba)_3$ (5 mg, 5 µmol, 5.0 mol%), *tert*-Bu-DavePhos (3.8 mg, 11 µmol, 10.0 mol%), and KOAc (83.5 mg, 0.85 mmol, 7.5 eq.) in DMF (9 mL, 12 mM) were reacted according to **GP5**. Flash column chromatography (*n*-pentane:CH_2Cl_2 = 9:1) afforded allene product **2f** (25.5 mg, 83 µmol, 73%) as yellow oil.

R_f = 0.37 (*n*-pentane:CH_2Cl_2 = 9:1).

^{1}H-NMR (500 MHz, $CDCl_3$): δ = 7.75 (d, J = 2.1 Hz, 1H), 7.22 (dd, J = 8.4, 2.1 Hz, 1H), 6.80 (d, J = 8.4 Hz, 1H), 4.85 – 4.81 (m, 1H), 4.81 – 4.76 (m, 2H), 2.49 (dddd, J = 11.6, 8.8, 5.7, 3.2 Hz, 1H), 2.33 – 2.20 (m, 2H), 1.47 – 1.43 (overlapped with 1.42, m, 1H), 1.42 (s, 9H), 1.34 (s, 9H).

^{13}C-NMR (126 MHz, $CDCl_3$): δ = 207.3, 162.7, 142.9, 136.8, 134.2, 126.4, 124.9, 123.2, 109.5, 99.7, 85.0, 75.9, 35.8, 34.7, 31.9, 29.9, 27.1, 22.5.

IR (ATR): $\tilde{\nu}$ (cm^{-1}) = 2956, 2868, 1479, 1462, 1394, 1363, 1319, 1297, 1261, 1226, 1122, 1108, 1085, 1051, 1029.

HRMS (APCI, Q-TOF): calculated for $C_{22}H_{29}O^+$ $[M+H]^+$: 309.2213, found: 309.2215.

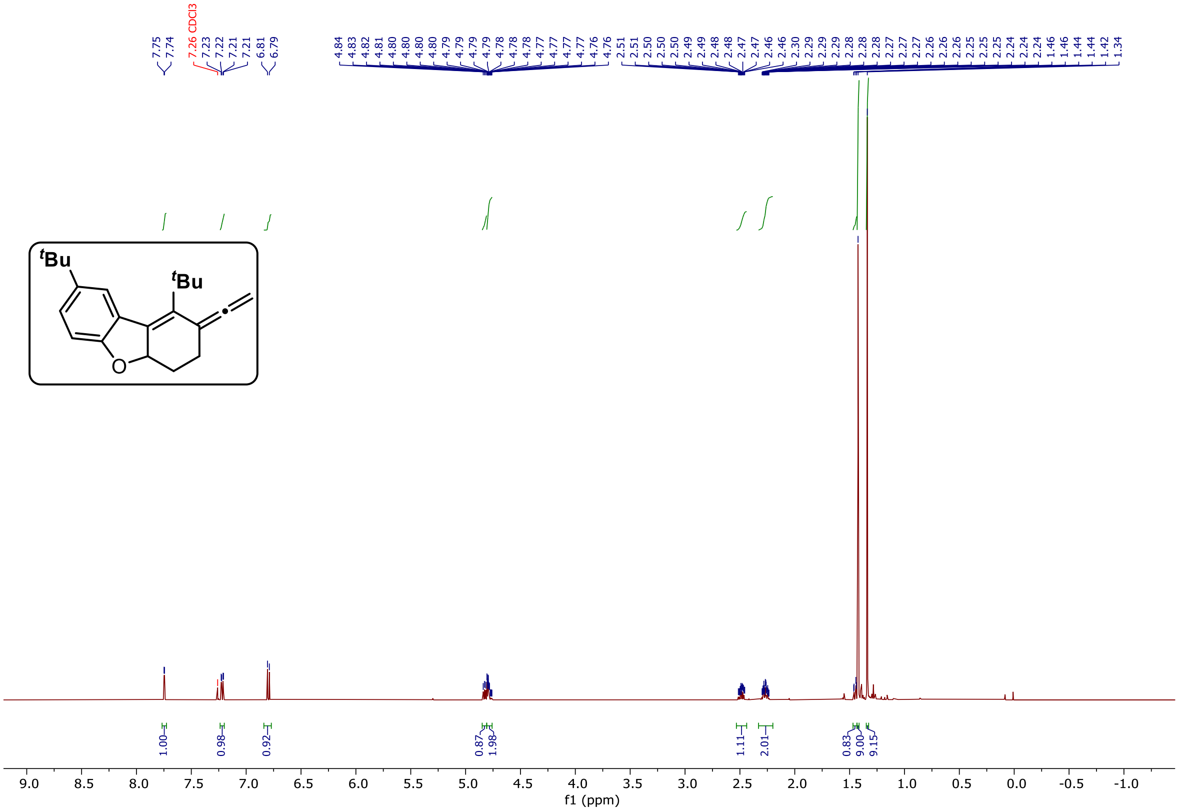

^{1}**H-NMR** (500 MHz, $CDCl_3$)

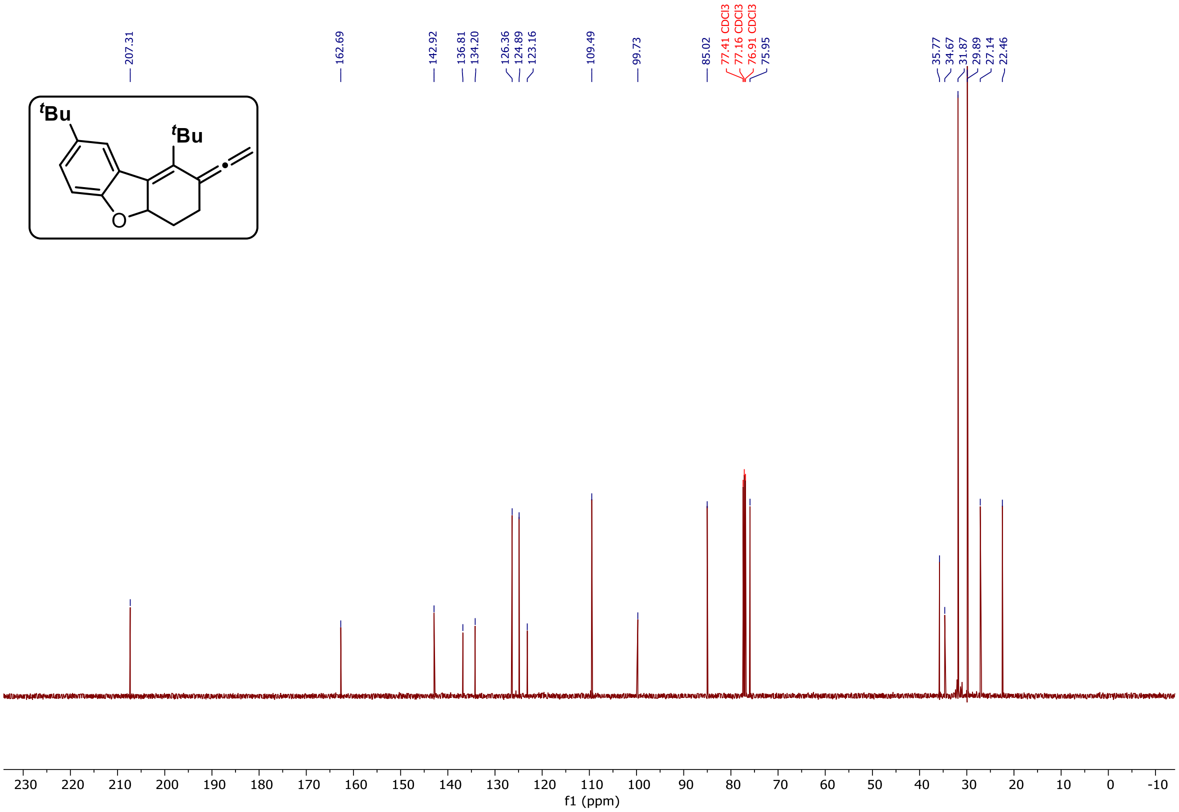

^{13}**C-NMR** (126 MHz, $CDCl_3$)

1,8-di-*tert*-butyl-2-vinylidene-2,3,4,4a-tetrahydrodibenzo[*b*,*d*]furan (2g)

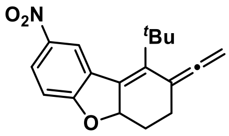

Domino precursor **1g** (42.5 mg, 0.10 mmol, 1.0 eq.), $Pd_2(dba)_3$ (5.0 mg, 5 µmol, 5.0 mol%), *tert*-Bu-DavePhos (3.8 mg, 11 µmol, 11.0 mol%), and KOAc (73.5 mg, 0.75 mmol, 7.5 eq.) in DMF (8 mL, 12 mM) were reacted according to **GP5**. Flash column chromatography (*n*-pentane:EtOAc = 90:1 and *n*-pentane:Et_2O = 100:1) afforded allene product **2g** (10.7 mg, 36 µmol, 36%) as bright yellow oil.

$\mathbf{R_f}$ = 0.2 (*n*-pentane:Et_2O = 100:1).

^{1}H-NMR (700 MHz, $CDCl_3$): δ = 8.55 (d, J = 2.4 Hz, 1H), 8.13 (dd, J = 8.9, 2.4 Hz, 1H), 6.88 (d, J = 8.9 Hz, 1H), 4.97 (dd, J = 11.3, 5.8 Hz, 1H), 4.85 (qd, J = 3.2, 0.6 Hz, 2H), 2.56 (dddd, J = 11.6, 7.2, 5.8, 4.7 Hz, 1H), 2.34 – 2.27 (m, 2H), 1.47 (tdd, J = 11.6, 10.1, 9.2 Hz, 1H), 1.42 (s, 9H).

^{13}C-NMR (176 MHz, $CDCl_3$): δ = 207.3, 169.4, 139.5, 133.3, 132.4, 127.1, 125.9, 123.4, 110.1, 99.2, 87.0, 76.7, 36.1, 29.6, 27.2, 22.0.

IR (ATR): $\tilde{\nu}$ (cm^{-1}) = 1583, 1517, 1462, 1364, 1329, 1256, 1233, 1181, 1157, 1116, 1076, 1049, 1024, 1004.

HRMS (APCI, Q-TOF): calculated for $C_{18}H_{20}NO_3^+$ $[M+H]^+$: 298.1438, found: 298.1440.

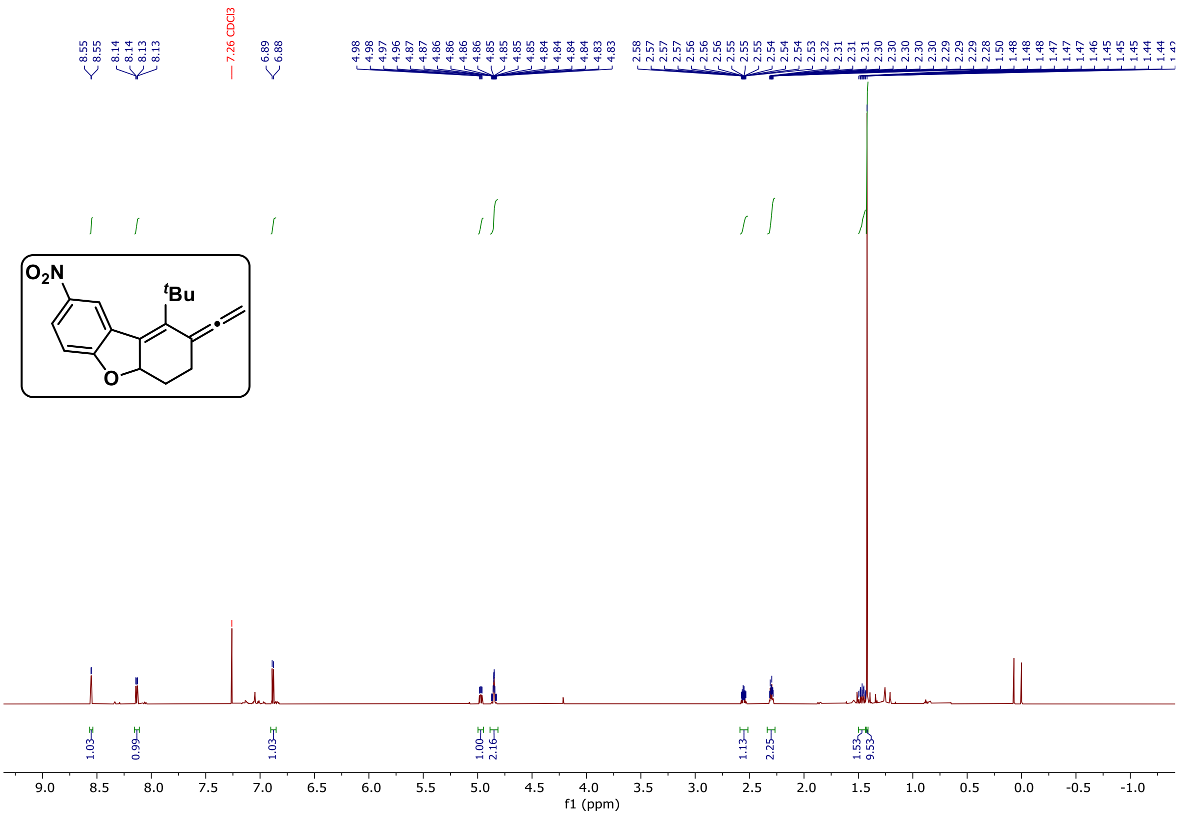

^{1}H-NMR (700 MHz, $CDCl_3$)

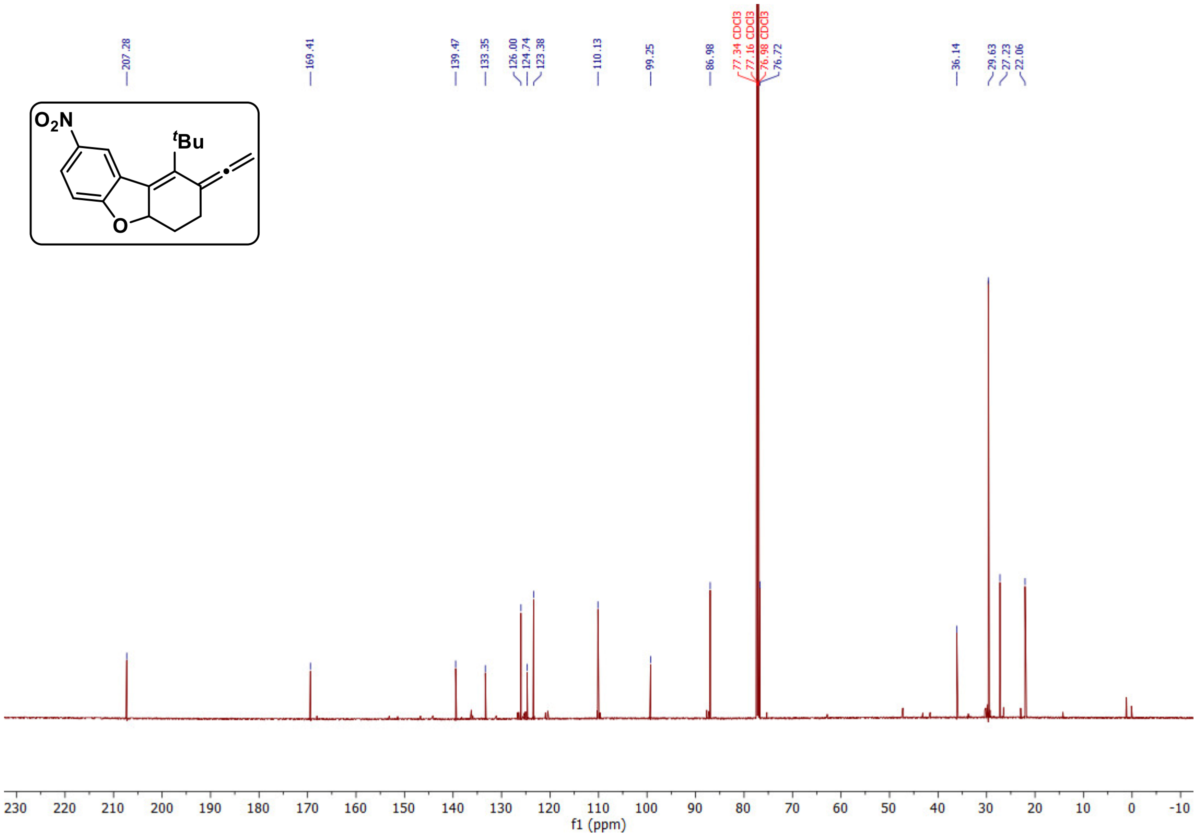

^{13}C-NMR (176 MHz, $CDCl_3$)

1-(*tert*-butyl)-8-(trifluoromethyl)-2-vinylidene-2,3,4,4a-tetrahydrodibenzo[*b*,*d*]furan (2h)

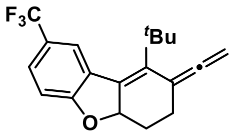

Domino precursor **1h** (44.9 mg, 0.10 mmol, 1.0 eq.), $Pd_2(dba)_3$ (4.7 mg, 5 µmol, 5.0 mol%), *tert*-Bu-DavePhos (3.5 mg, 10 µmol, 10.0 mol%), and KOAc (73.7 mg, 0.75 mmol, 7.5 eq.) in DMF (8 mL, 12 mM) were reacted according to **GP5**. Flash column chromatography (*n*-pentane:EtOAc = 90:1 to 60:1) afforded allene product **2h** (15 mg, 47 µmol, 47%) as yellow oil.

$\mathbf{R_f}$ = 0.45 (*n*-pentane:Et_2O = 60:1).

^{1}H-NMR (500 MHz, $CDCl_3$): δ = 7.88 (d, J = 1.9 Hz, 1H), 7.42 (ddt, J = 8.5, 2.0, 0.8 Hz, 1H), 6.90 (dt, J = 8.5, 0.6 Hz, 1H), 4.89 (dd, J = 11.3, 5.8 Hz, 1H), 4.86 – 4.77 (m, 2H), 2.56 – 2.46 (m, 1H), 2.32 – 2.25 (m, 2H), 1.46 – 1.40 (m, 1H), 1.39 (s, 9H).

^{13}C-NMR (126 MHz, $CDCl_3$): δ = 207.2, 166.9, 137.4, 134.6, 126.7 (q, J = 3.8 Hz), 124.7 (q, J = 4.0 Hz), 124.7 (q, J = 271.4 Hz), 124.3, 122.7 (q, J = 32.2 Hz), 110.4, 99.4, 85.8, 76.4, 35.9, 29.7, 27.2, 22.1.

^{19}F-NMR (471 MHz, $CDCl_3$): δ = -61.21.

IR (ATR): $\tilde{\nu}$ (cm^{-1}) = 1676, 1616, 1454, 1365, 1321, 1271, 1232, 1161, 1115, 1058, 1029, 1015, 1006.

HRMS (APCI, Q-TOF): calculated for $C_{19}H_{20}F_3O^+$ [M+H]$^+$: 321.1461, found: 321.1459.

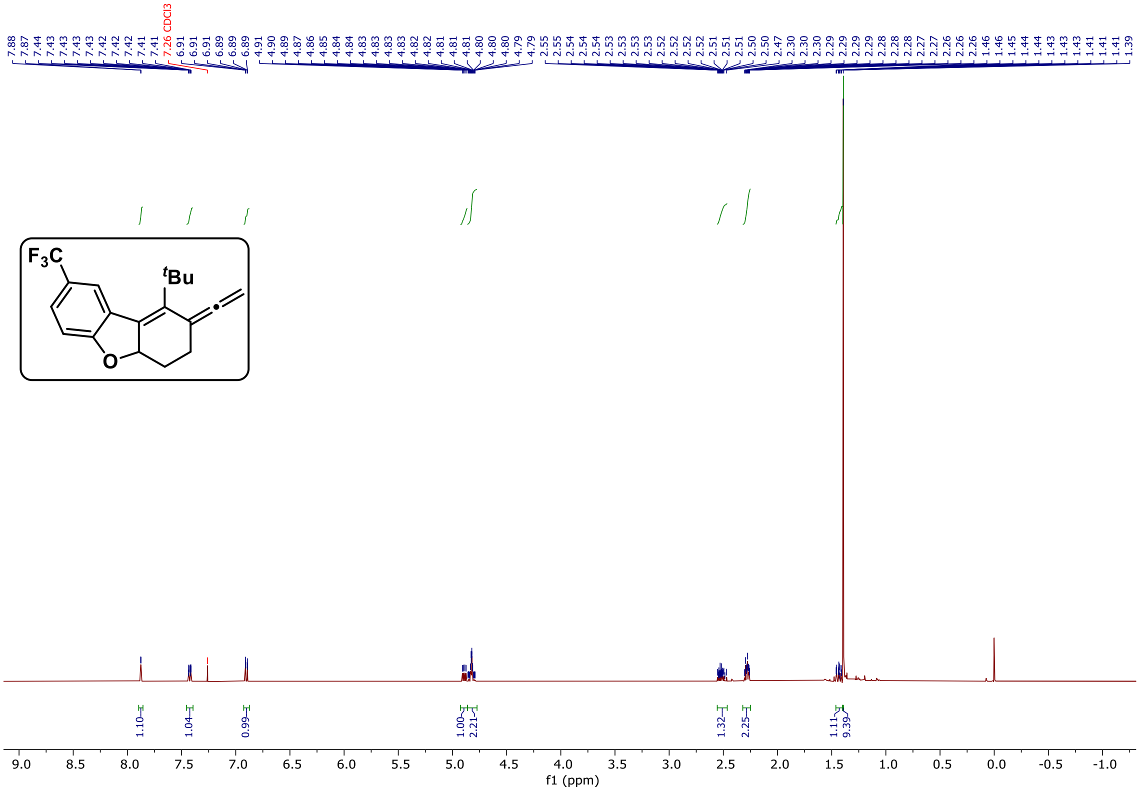

¹H-NMR (500 MHz, $CDCl_3$)

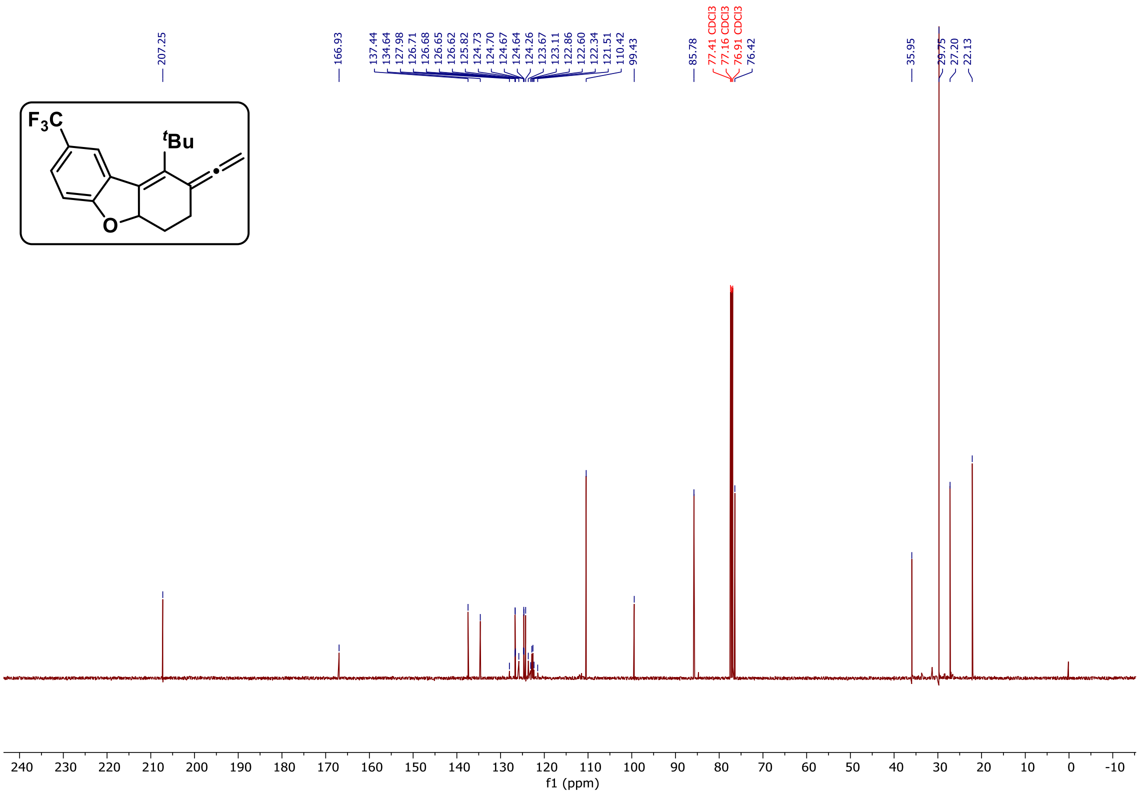

¹³C-NMR (126 MHz, $CDCl_3$)

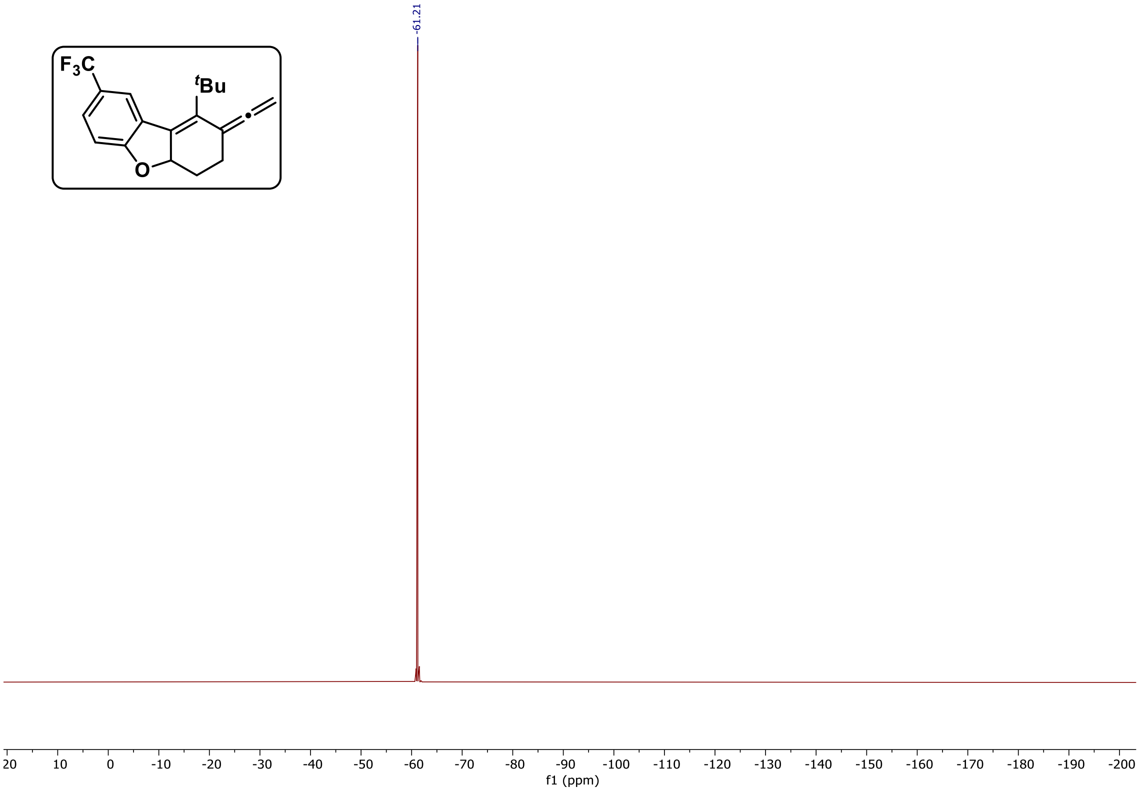

^{19}F-NMR (471 MHz, $CDCl_3$)

1-(*tert*-butyl)-7-methoxy-2-vinylidene-2,3,4,4a-tetrahydrodibenzo[*b*,*d*]furan (2i)

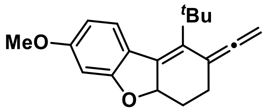

Domino precursor **1i** (41 mg, 0.10 mmol, 1.0 eq.), $Pd_2(dba)_3$ (4.7 mg, 5 µmol, 5.0 mol%), *tert*-Bu-DavePhos (3.6 mg, 10 µmol, 10.0 mol%), and KOAc (73.7 mg, 0.75 mmol, 7.5 eq.) in DMF (8 mL, 12 mM) were reacted according to **GP5**. Flash column chromatography (*n*-pentane:EtOAc = 60:1 and *n*-pentane:CH_2Cl_2 = 1:1) afforded allene product **2i** (14.2 mg, 50 µmol, 50%) as yellow oil.

$\mathbf{R_f}$ = 0.5 (*n*-pentane:Et_2O = 60:1).

^{1}H-NMR (700 MHz, $CDCl_3$): δ = 7.54 (d, *J* = 8.7 Hz, 1H), 6.50 (dd, *J* = 8.7, 2.5 Hz, 1H), 6.43 (d, *J* = 2.4 Hz, 1H), 4.83 (ddd, *J* = 11.3, 5.7, 0.6 Hz, 1H), 4.81 – 4.74 (m, 2H), 3.80 (s, 3H), 2.49 – 2.44 (m, 1H), 2.31 – 2.21 (m, 2H), 1.44 – 1.38 (m, 1H), 1.37 (s, 9H).

^{13}C-NMR (176 MHz, $CDCl_3$): δ = 207.4, 166.4, 161.3, 135.9, 131.8, 128.0, 116.6, 106.9, 99.6, 96.0, 85.7, 75.8, 55.6, 35.7, 29.8, 27.1, 22.6.

IR (ATR): $\tilde{\nu}$ (cm^{-1}) = 2935, 1704, 1613, 1495, 1444, 1397, 1365, 13334, 1283, 1194, 1153, 1113, 1071, 1033.

HRMS (APCI, Q-TOF): calculated for $C_{19}H_{23}O_2^+$ $[M+H]^+$: 283.1693, found: 283.1694.

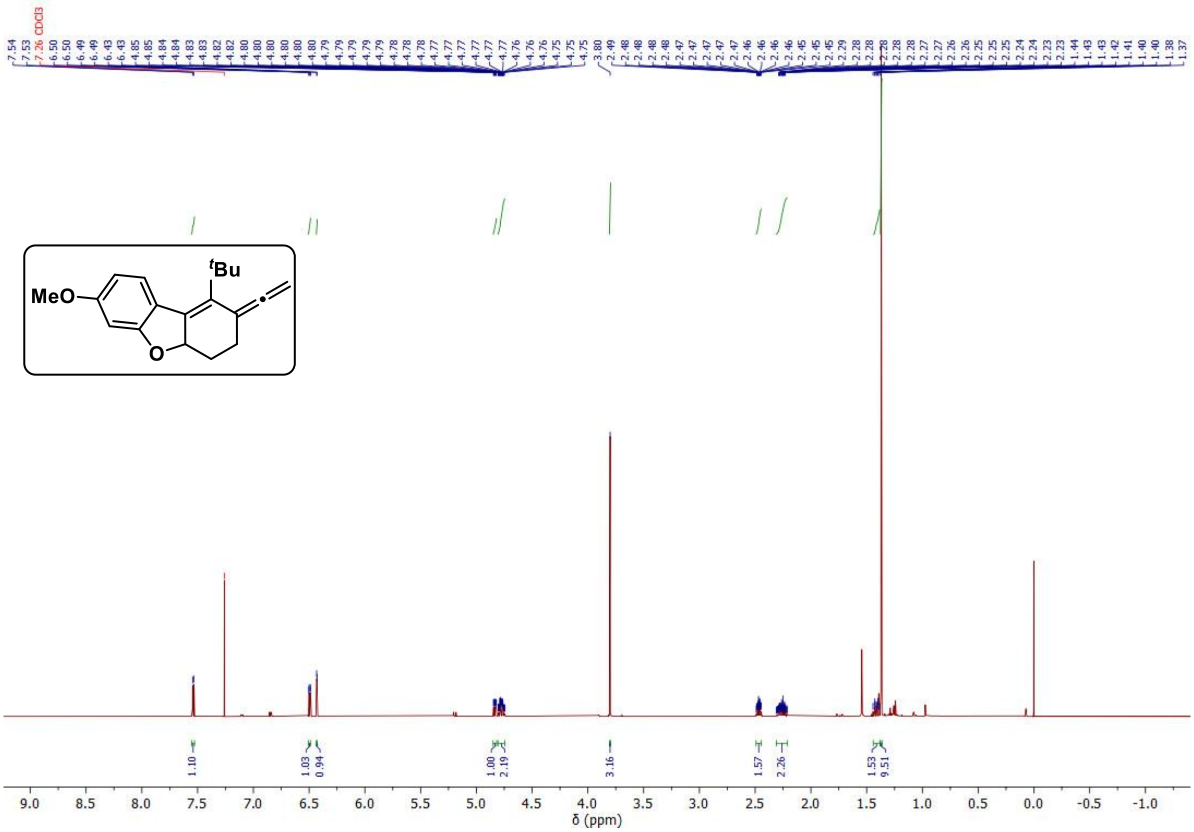

^{1}H-NMR (700 MHz, $CDCl_3$)

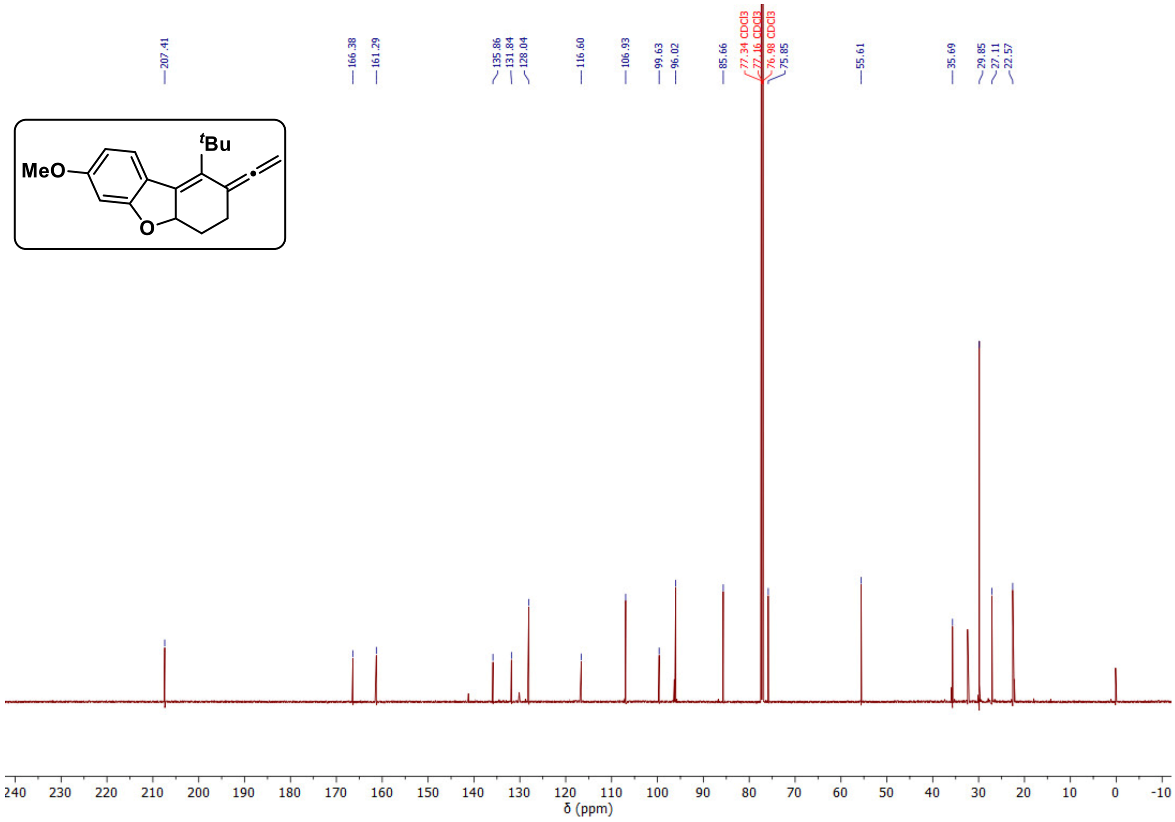

^{13}C-NMR (176 MHz, $CDCl_3$)

9-(*tert*-butyl)-8-vinylidene-5a,6,7,8-tetrahydrodibenzo[*b*,*d*]furan-3-carbaldehyde (2j)

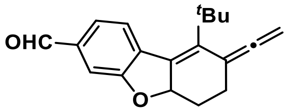

Domino precursor **1j** (81 mg, 0.20 mmol, 1.0 eq.), $Pd_2(dba)_3$ (4.7 mg, 10 µmol, 5.0 mol%), *tert*-Bu-DavePhos (7.1 mg, 20 µmol, 10.0 mol%), and KOAc (135 mg, 1.5 mmol, 7.5 eq.) in DMF (16 mL, 12 mM) were reacted according to **GP5**. Flash column chromatography (*n*-pentane:EtOAc = 80:1 and *n*-pentane:CH_2Cl_2 = 10:1) afforded allene product **2j** (17.4 mg, 62 µmol, 31%) as yellow oil.

$\mathbf{R_f}$ = 0.43 (*n*-pentane:CH_2Cl_2 = 10:1).

^{1}H-NMR (500 MHz, $CDCl_3$): δ = 9.94 (s, 1H), 7.79 (d, *J* = 8.0 Hz, 1H), 7.44 (dd, *J* = 8.1, 1.5 Hz, 1H), 7.31 (dd, *J* = 1.5, 0.4 Hz, 1H), 4.89 (dd, *J* = 11.3, 5.7 Hz, 1H), 4.84 (qd, *J* = 3.1, 0.6 Hz, 2H), 2.59 – 2.48 (m, 1H), 2.34 – 2.25 (m, 2H), 1.47 – 1.42 (m, 1H), 1.41 (s, 9H).

^{13}C-NMR (126 MHz, $CDCl_3$): δ = 207.2, 191.6, 165.1, 139.9, 136.9, 135.3, 130.1, 127.7, 123.2, 109.6, 99.7, 85.5, 76.6, 36.3, 29.9, 27.3, 22.1.

IR (ATR): $\tilde{\nu}$ (cm^{-1}) = 1689, 1621, 1430, 1394, 1364, 1276, 1239, 1141, 1101, 1049, 1029, 1006, 958.

HRMS (APCI, Q-TOF): calculated for $C_{19}H_{21}O_2^+$ [M+H]$^+$: 281.1536, found: 281.1538.

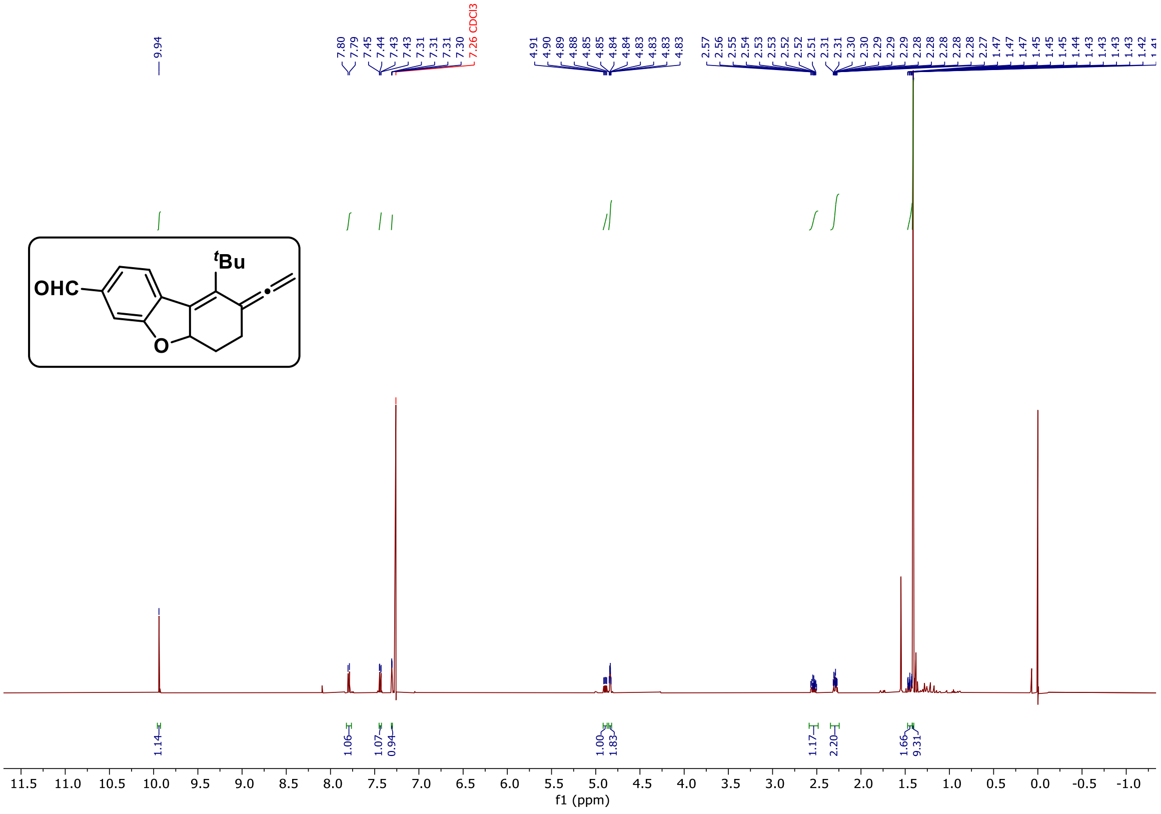

^{1}H-NMR (500 MHz, $CDCl_3$)

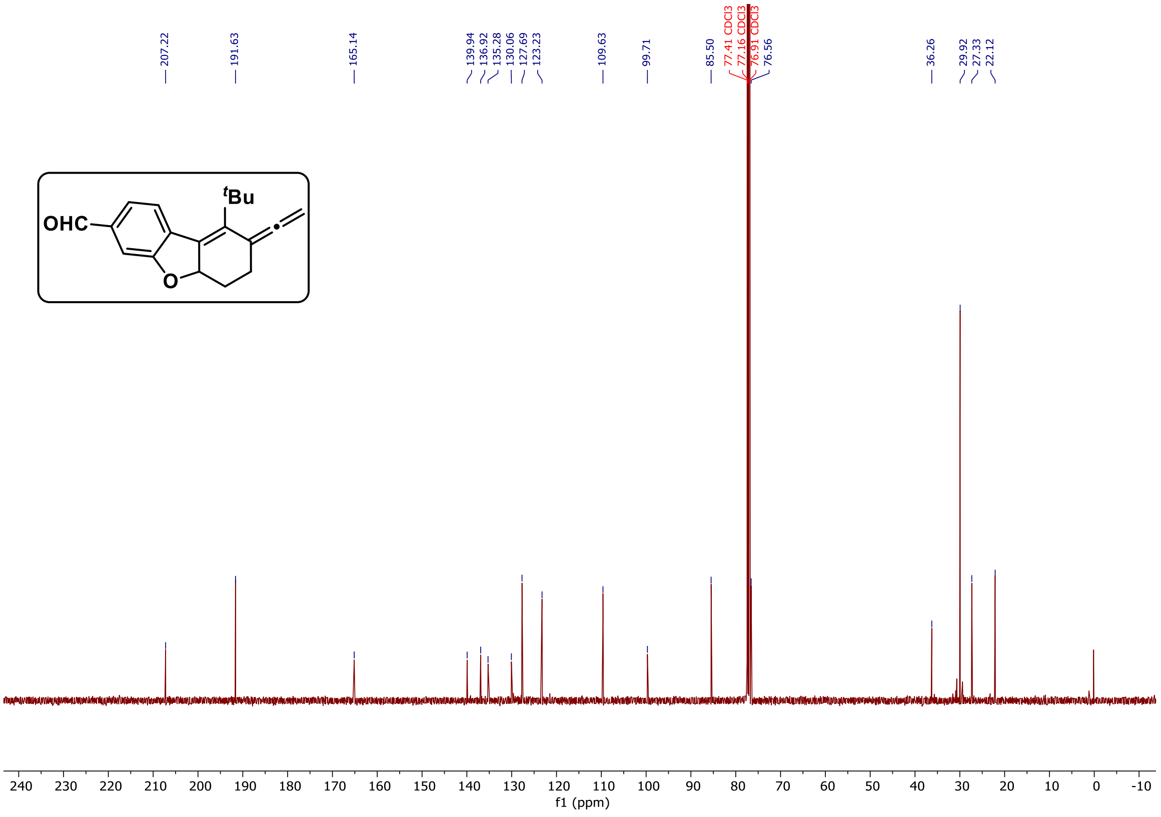

^{13}C-NMR (126 MHz, $CDCl_3$)

9-(*tert*-butyl)-8-vinylidene-5a,6,7,8-tetrahydrodibenzo[*b*,*d*]furan-3-carbonitrile (2k)

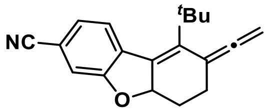

Domino precursor **1k** (40.5 mg, 0.10 mmol, 1.0 eq.), $Pd_2(dba)_3$ (4.6 mg, 5 µmol, 5.0 mol%), *tert*-Bu-DavePhos (3.5 mg, 10 µmol, 10.0 mol%), and KOAc (73.1 mg, 0.75 mmol, 7.5 eq.) in DMF (8 mL, 12 mM) were reacted according to **GP5**. Flash column chromatography (*n*-pentane:EtOAc = 60:1 and *n*-pentane:CH_2Cl_2 = 10:1) afforded allene product **2k** (11.1 mg, 40 µmol, 40%) as colorless oil.

R_f = 0.38 (*n*-pentane:CH_2Cl_2 = 10:1).

^{1}H-NMR (500 MHz, $CDCl_3$): δ = 7.70 (d, J = 8.1 Hz, 1H), 7.20 (dd, J = 8.1, 1.5 Hz, 1H), 7.07 – 7.05 (m, 1H), 4.87 (dd, J = 11.3, 5.7 Hz, 1H), 4.84 (q, J = 2.8 Hz, 2H), 2.53 (dq, J = 11.6, 5.7 Hz, 1H), 2.31 – 2.25 (m, 2H), 1.48 – 1.40 (m, 1H), 1.38 (s, 9H).

^{13}C-NMR (126 MHz, $CDCl_3$): δ = 207.2, 164.3, 140.1, 134.5, 128.7, 127.9, 124.5, 119.1, 113.3, 111.7, 99.5, 85.6, 76.7, 36.2, 29.8, 27.3, 22.0.

IR (ATR): $\tilde{\nu}$ (cm^{-1}) = 1624,1480, 1425, 1365, 1317, 1265, 1246, 1219, 1190, 1146, 1086, 1050, 1030, 1006, 962.

HRMS (APCI, Q-TOF): calculated for $C_{19}H_{20}NO^+$ $[M+H]^+$: 276.1394, found: 276.1396.

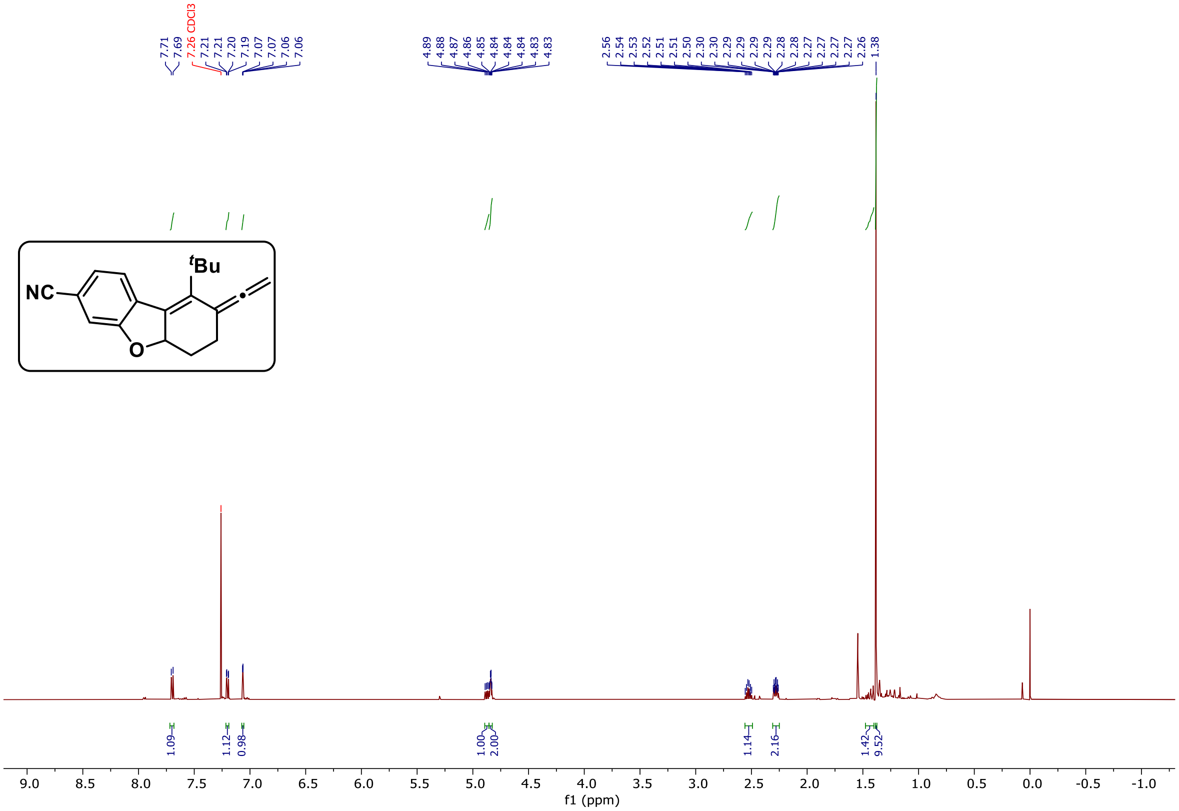

^{1}**H-NMR** (500 MHz, CDCl$_3$)

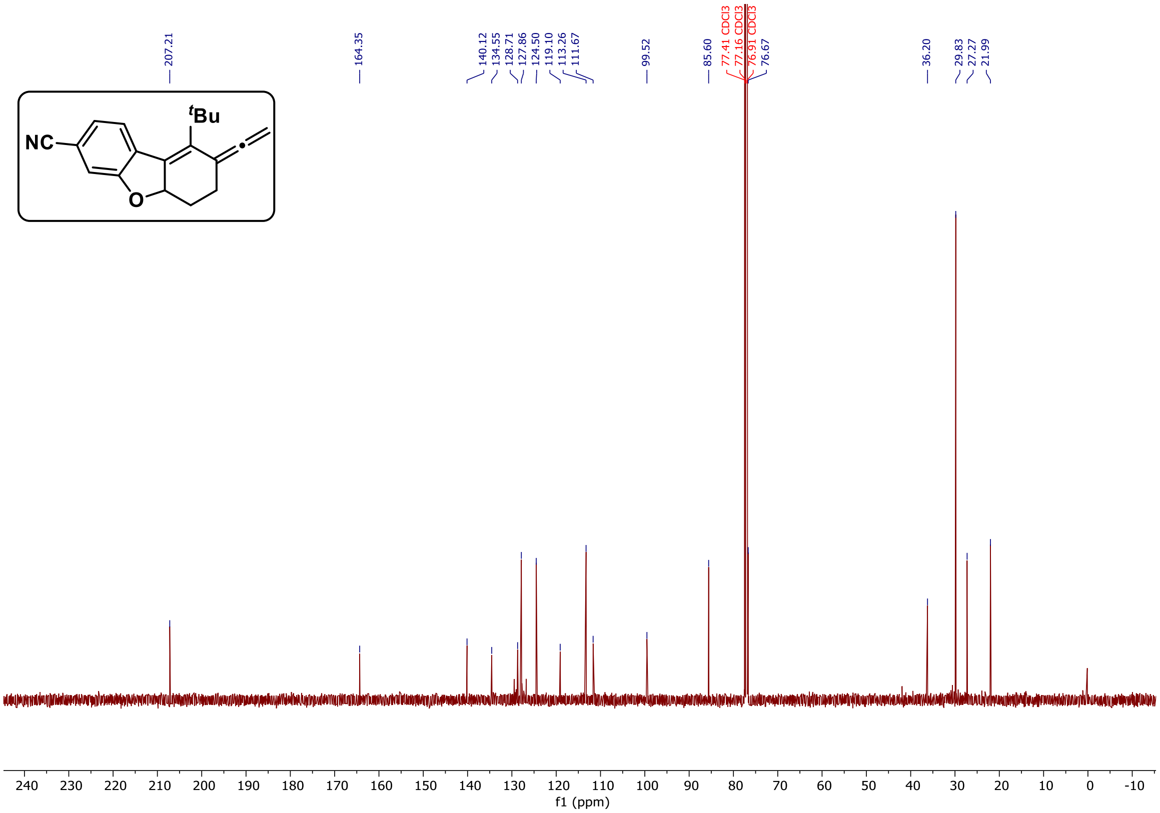

^{13}**C-NMR** (126 MHz, CDCl$_3$)

1-(*tert*-butyl)-2-vinylidene-2,3,4,4a-tetrahydronaphtho[2,3-*b*]benzofuran (2l)

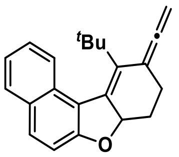

Domino precursor **1l** (30 mg, 0.08 mmol, 1.0 eq.), $Pd_2(dba)_3$ (4.2 mg, 4 µmol, 5.0 mol%), *tert*-Bu-DavePhos (2.8 mg, 8 µmol, 10.0 mol%), and KOAc (57.6 mg, 0.59 mmol, 7.5 eq.) in DMF (6.6 mL, 12 mM) were reacted according to **GP5**. Flash column chromatography (*n*-pentane:CH_2Cl_2 = 10:1) afforded allene product **2l** (14 mg, 46 µmol, 59%) as yellow oil.

R_f = 0.26 (*n*-pentane:CH_2Cl_2 = 10:1).

^{1}H-NMR (700 MHz, $CDCl_3$): δ = 8.05 (dq, J = 8.5, 0.9 Hz, 1H), 7.74 (ddt, J = 8.2, 1.3, 0.6 Hz, 1H), 7.68 (d, J = 8.7 Hz, 1H), 7.42 (ddd, J = 8.3, 6.7, 1.3 Hz, 1H), 7.28 – 7.26 (overlapped with $CDCl_3$ peak, m, 1H), 7.08 (d, J = 8.7 Hz, 1H), 5.08 (ddt, J = 10.9, 5.1, 1.5 Hz, 1H), 4.93 (ddt, J = 5.0, 4.1, 1.3 Hz, 2H), 2.55 – 2.47 (m, 2H), 2.35 – 2.28 (m, 1H), 1.72 – 1.65 (m, 1H), 1.33 (s, 9H).

^{13}C-NMR (176 MHz, $CDCl_3$): δ = 208.0, 162.4, 136.7, 135.9, 131.8, 131.2, 129.6, 128.7, 126.2, 125.8, 122.9, 120.5, 112.3, 101.4, 88.3, 77.2, 38.5, 31.2, 27.7, 23.0.

IR (ATR): $\tilde{\nu}$ (cm^{-1}) = 2957, 2923, 1455, 1362, 1352, 1262, 1235, 1208, 1133, 1110, 1072, 1044, 1001, 981.

HRMS (APCI, Q-TOF): calculated for $C_{22}H_{23}O^+$ $[M+H]^+$: 303.1743, found: 303.1748.

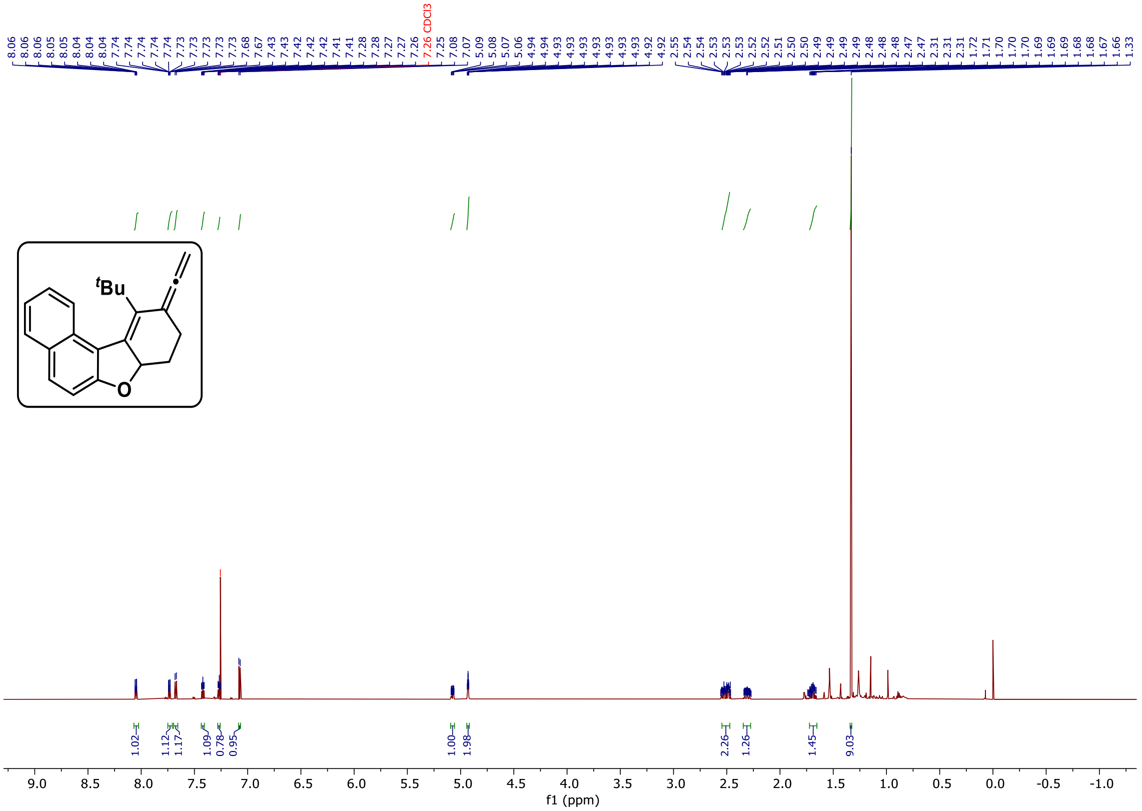

^{1}H-NMR (700 MHz, $CDCl_3$)

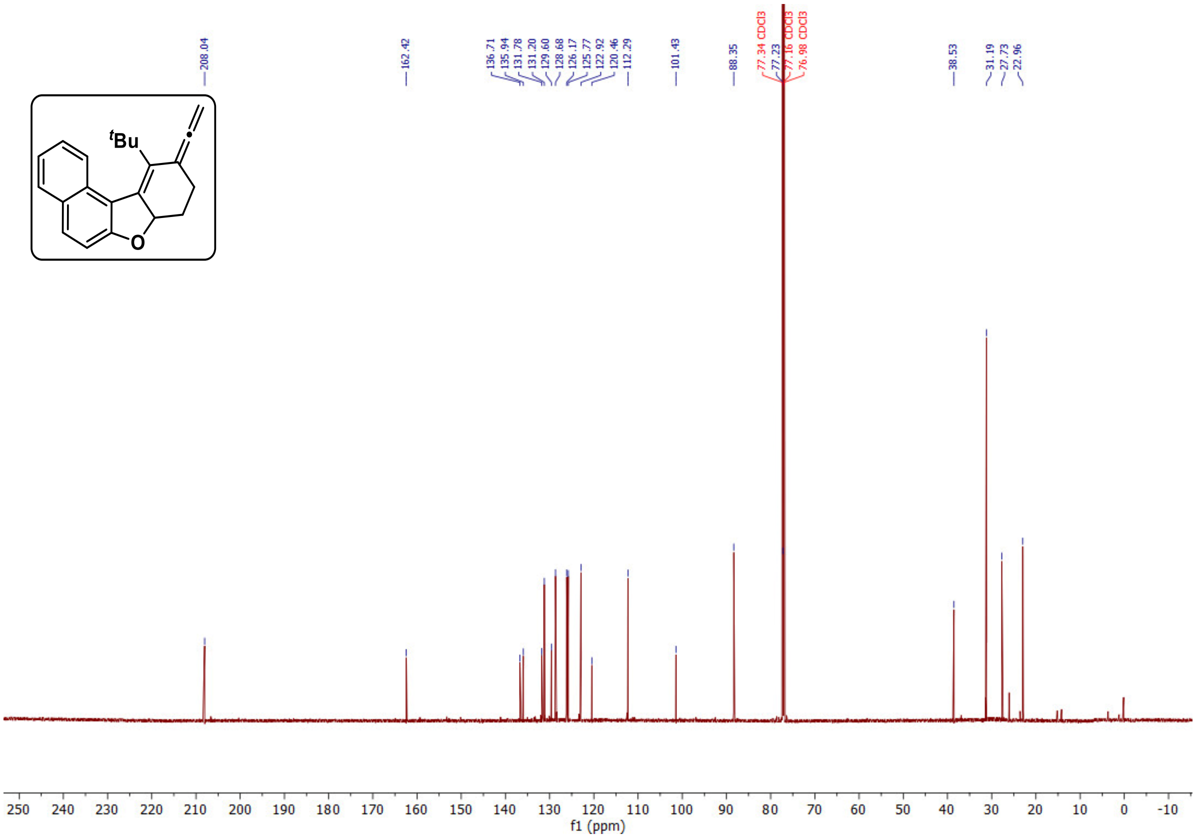

^{13}C-NMR (176 MHz, $CDCl_3$)

1-(*tert*-butyl)-2-vinylidene-2,3,4,4a-tetrahydroanthra[2,3-*b*]benzofuran (2m)

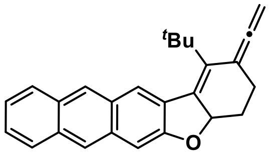

Domino precursor **1m** (43.4 mg, 0.1 mmol, 1.0 eq.), $Pd_2(dba)_3$ (4.6 mg, 5 µmol, 5.0 mol%), *tert*-Bu-DavePhos (3.4 mg, 10 µmol, 10.0 mol%), and KOAc (73.6 mg, 0.75 mmol, 7.5 eq.) in DMF (8 mL, 12 mM) were reacted according to **GP5**. Flash column chromatography (*n*-pentane:CH_2Cl_2 = 10:1 to 8:1) afforded allene product **2m** (21.5 mg, 61 µmol, 61%) as yellow solid.

R_f = 0.4 (*n*-pentane:CH_2Cl_2 = 8:1).

^{1}H-NMR (700 MHz, $CDCl_3$): δ = 8.39 (d, J = 1.1 Hz, 1H), 8.23 – 8.17 (m, 2H), 7.94 (dd, J = 8.3, 1.0 Hz, 1H), 7.91 – 7.89 (m, 1H), 7.42 (ddd, J = 8.2, 6.5, 1.3 Hz, 1H), 7.37 (ddd, J = 7.9, 6.5, 1.2 Hz, 1H), 7.21 (s, 1H), 4.92 (dd, J = 11.4, 5.7 Hz, 1H), 4.87 (qdd, J = 11.1, 4.8, 1.9 Hz, 2H), 2.56 (dddd, J = 11.5, 9.1, 5.7, 2.3 Hz, 1H), 2.42 – 2.33 (m, 2H), 1.53 (s, 9H), 1.52 – 1.50 (overlapped with 1.53, m, 1H).

^{13}C-NMR (176 MHz, $CDCl_3$): δ = 207.3, 161.6, 139.8, 135.2, 132.7, 132.6, 130.5, 128.9, 128.3, 127.8, 127.7, 127.6, 126.8, 125.8, 124.5, 123.5, 101.8, 99.9, 84.8, 76.5, 36.3, 29.5, 27.5, 23.0.

IR (ATR): $\tilde{\nu}$ (cm^{-1}) = 1636, 1438, 1363, 1326, 1288, 1264, 1192, 1172, 1159, 1086, 1047, 1029, 1005.

HRMS (APCI, Q-TOF): calculated for $C_{26}H_{25}O^+$ $[M+H]^+$: 353.1900, found: 353.1905.

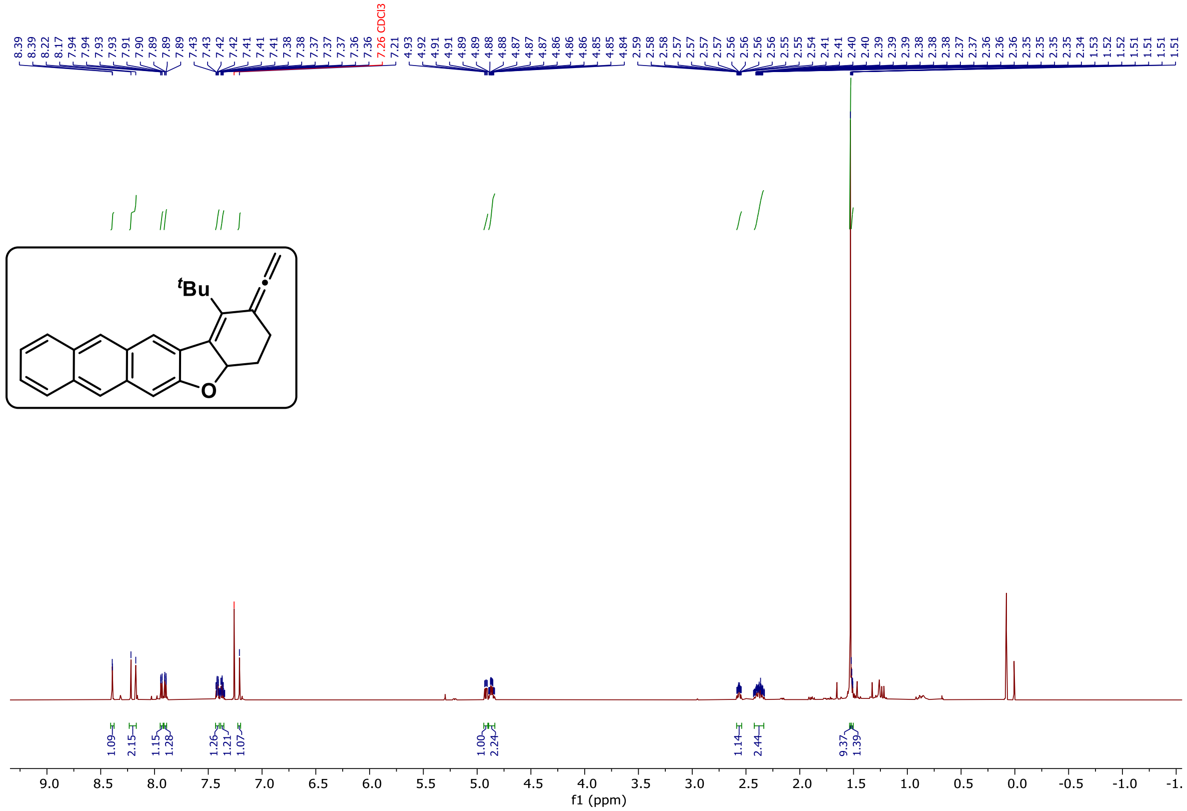

^{1}H-NMR (700 MHz, $CDCl_3$)

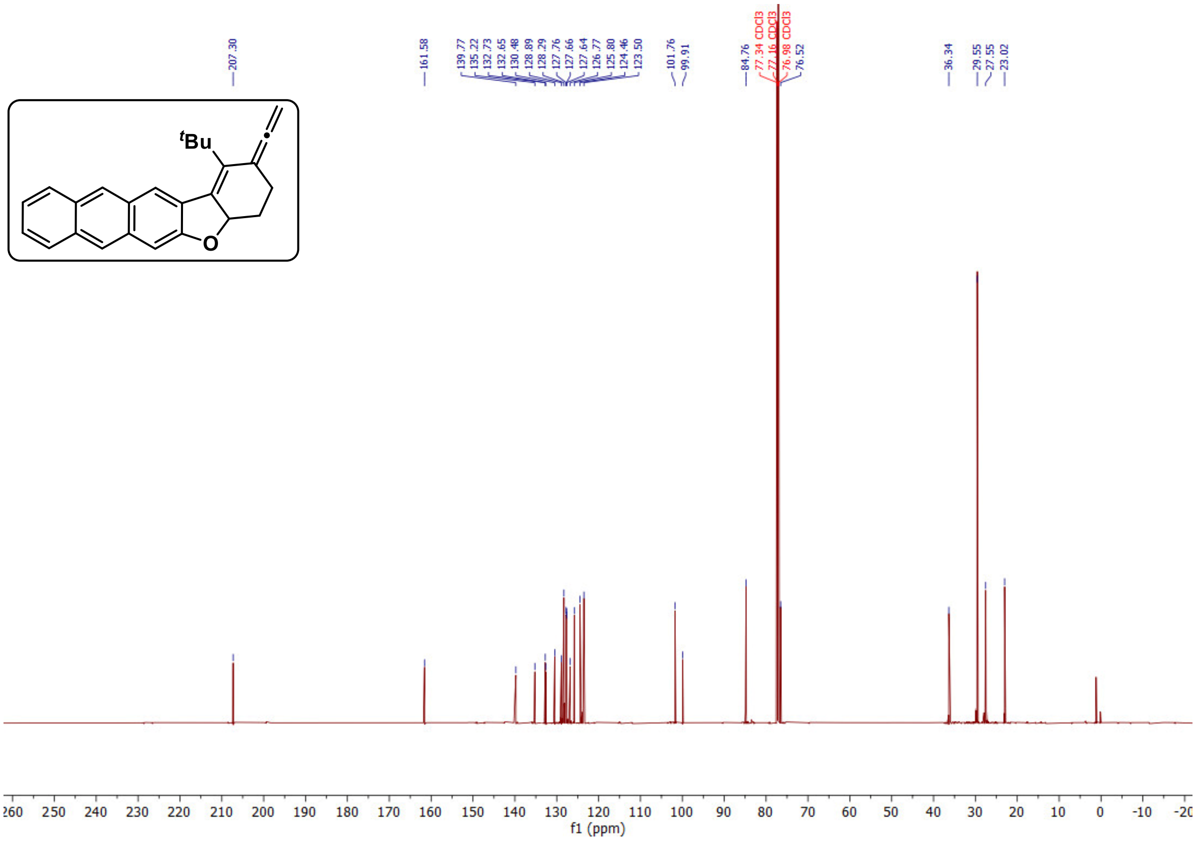

^{13}C-NMR (176 MHz, $CDCl_3$)

1-(*tert*-butyl)-2-vinylidene-2,4a,5,6-tetrahydro-3*H*-benzo[*f*]chromene (2n)

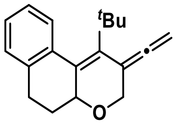

Domino precursor **1n'** (43.2 mg, 0.11 mmol, 1.0 eq.), $Pd_2(dba)_3$ (5.1 mg, 5 µmol, 5.0 mol%), *tert*-Bu-DavePhos (3.9 mg, 11 µmol, 10.0 mol%), and KOAc (80.6 mg, 0.82 mmol, 7.5 eq.) in DMF (9 mL, 12 mM) were reacted according to **GP5**. Flash column chromatography (*n*-pentane:EtOAc = 80:1 to 40:1) afforded allene product **2n** (16.3 mg, 61 µmol, 56%) as light-yellow oil.

In the case of employing **1n**, the domino product **2n** is obtained in 44% yield.

R_f = 0.28 (*n*-pentane:EtOAc = 60:1).

^{1}H-NMR (500 MHz, $CDCl_3$): δ = 7.21 – 7.17 (m, 2H), 7.14 – 7.08 (m, 2H), 5.03 (ddt, J = 11.9, 3.6, 1.9 Hz, 1H), 4.97 (ddt, J = 11.9, 3.6, 1.9 Hz, 1H), 4.34 (dt, J = 12.1, 1.8 Hz, 1H), 4.27 (dt, J = 12.1, 3.3 Hz, 1H), 4.17 (ddt, J = 7.6, 5.5, 2.0 Hz, 1H), 2.84 – 2.76 (m, 1H), 2.66 (ddd, J = 14.9, 6.6, 3.8 Hz, 1H), 2.13 – 2.06 (m, 1H), 1.93 – 1.85 (m, 1H), 1.19 (s, 9H).

^{13}C-NMR (126 MHz, $CDCl_3$): δ = 207.0, 139.8, 138.1, 137.3, 136.0, 130.7, 127.9, 126.7, 124.7, 99.3, 78.4, 76.7, 68.3, 38.3, 32.5, 30.0, 27.6.

IR (ATR): $\tilde{\nu}$ (cm^{-1}) = 1720, 1672, 1449, 1363, 1303, 1266, 1227, 1207, 1193, 1144, 1086, 1067, 967.

HRMS (APCI, Q-TOF): calculated for $C_{19}H_{23}O^+$ $[M+H]^+$: 267.1743, found: 267.1739.

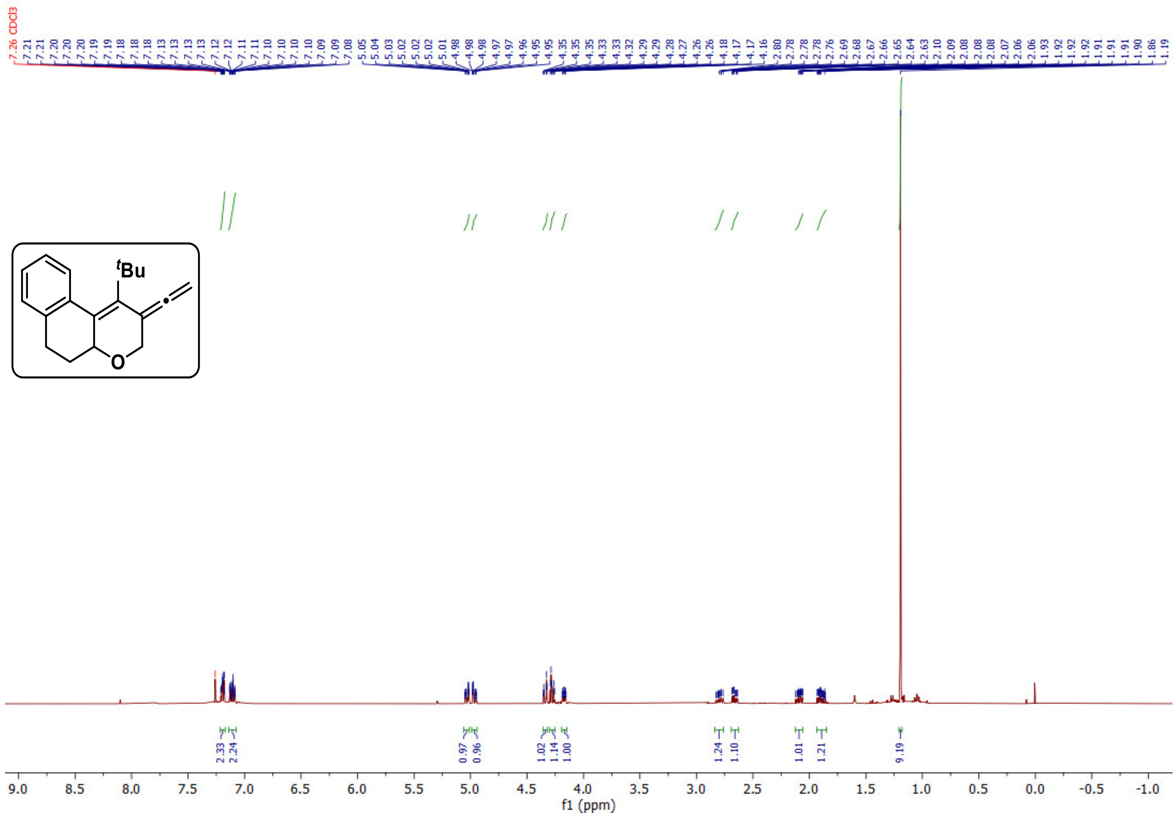

^{1}H-NMR (500 MHz, $CDCl_3$)

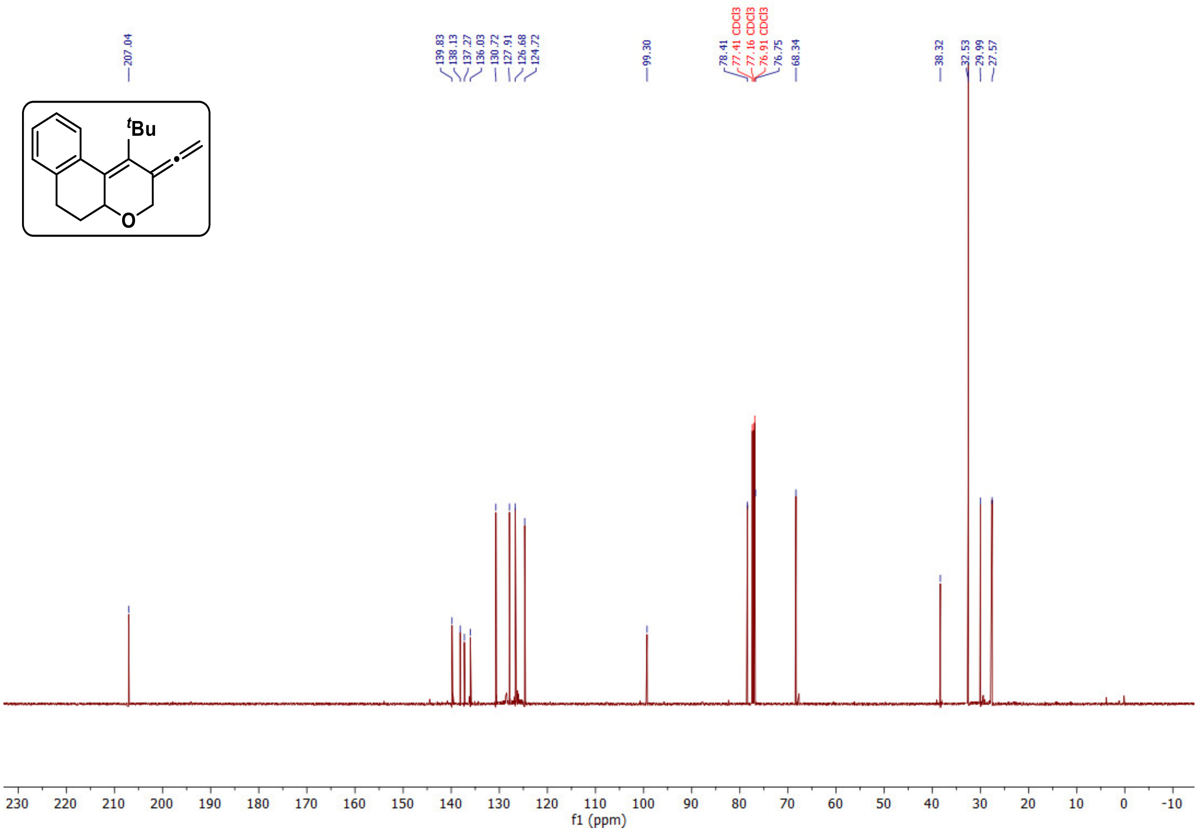

^{13}C-NMR (126 MHz, $CDCl_3$)

5-(*tert*-butyl)-4-vinylidene-1,3,4,10a-tetrahydrooxepino[3,4-*b*]benzofuran (2o)

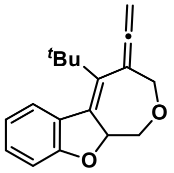

Domino precursor **1o** (69 mg, 0.17 mmol, 1.0 eq.), $Pd_2(dba)_3$ (8 mg, 5 µmol, 5.0 mol%), *tert*-Bu-DavePhos (6 mg, 17 µmol, 10.0 mol%), and KOAc (125.6 mg, 1.28 mmol, 7.5 eq.) in DMF (13.5 mL, 13 mM) were reacted according to **GP5**. Flash column chromatography (*n*-pentane to *n*-pentane:EtOAc = 100:1) afforded allene product **2o** (28 mg, 0.10 mmol, 60%) as yellow oil.

$\mathbf{R_f}$ = 0.35 (*n*-pentane:EtOAc = 100:1).

^{1}H-NMR (400 MHz, $CDCl_3$): δ = 7.68 (dd, *J* = 8.0, 1.3 Hz, 1H), 7.16 (ddd, *J* = 8.1, 7.4, 1.3 Hz, 1H), 6.91 (ddd, *J* = 8.0, 7.4, 1.2 Hz, 1H), 6.80 (dd, *J* = 8.0, 1.1 Hz, 1H), 5.64 (dd, *J* = 9.0, 3.4 Hz, 1H), 4.93 – 4.81 (m, 2H), 4.29 (dd, *J* = 11.3, 0.8 Hz, 1H), 3.98 (ddd, *J* = 11.5, 3.4, 0.6 Hz, 1H), 3.88 (dt, *J* = 11.3, 1.7 Hz, 1H), 3.57 (dd, *J* = 11.5, 9.0 Hz, 1H), 1.37 (s, 9H).

^{13}C-NMR (101 MHz, $CDCl_3$): δ = 207.4, 164.6, 137.9, 137.5, 129.8, 128.3, 123.0, 120.5, 110.6, 104.2, 86.5, 75.6, 73.0, 70.3, 36.0, 29.9.

IR (ATR): $\tilde{\nu}$ (cm^{-1}) = 2957, 1737, 1458, 1366, 1320, 1306, 1241, 1226, 1213, 1176, 1152, 1103, 1065, 1034, 1017, 999.

HRMS (APCI, Q-TOF): calculated for $C_{18}H_{21}O_2^+$ [M+H]$^+$: 269.1536, found: 269.1535.

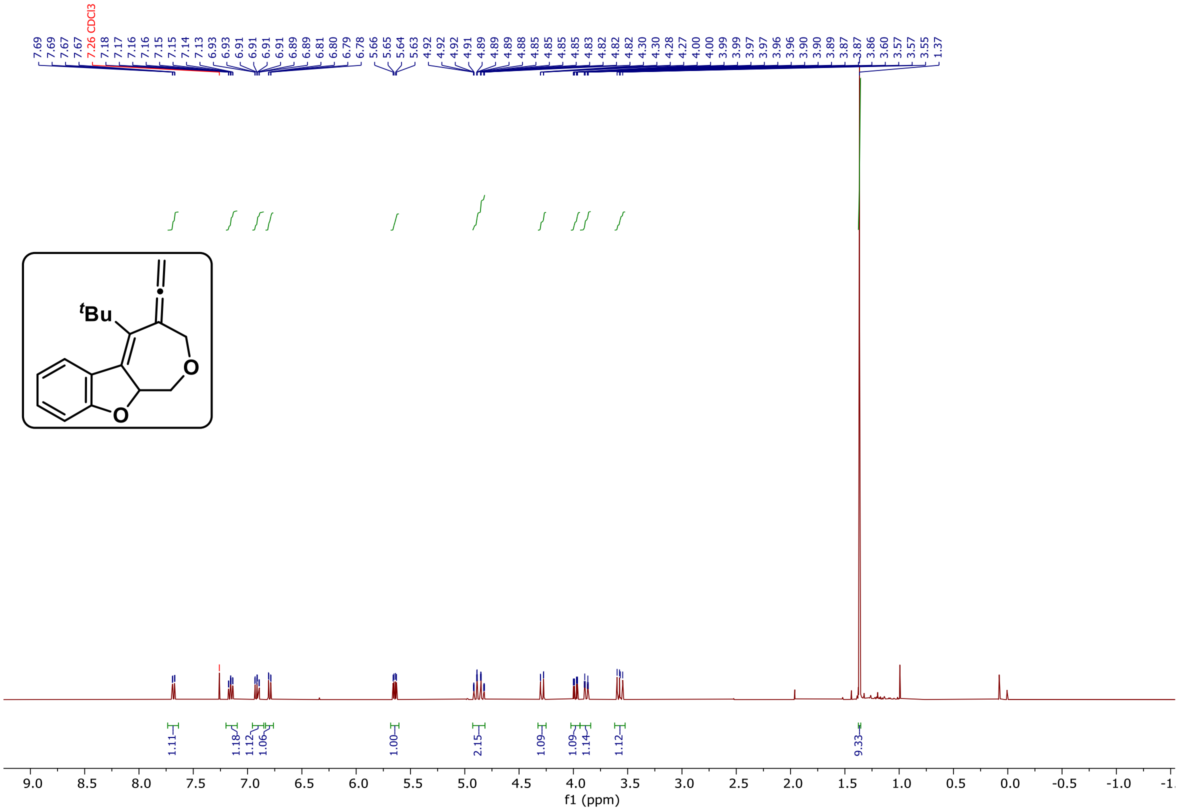

^{1}H-NMR (400 MHz, $CDCl_3$)

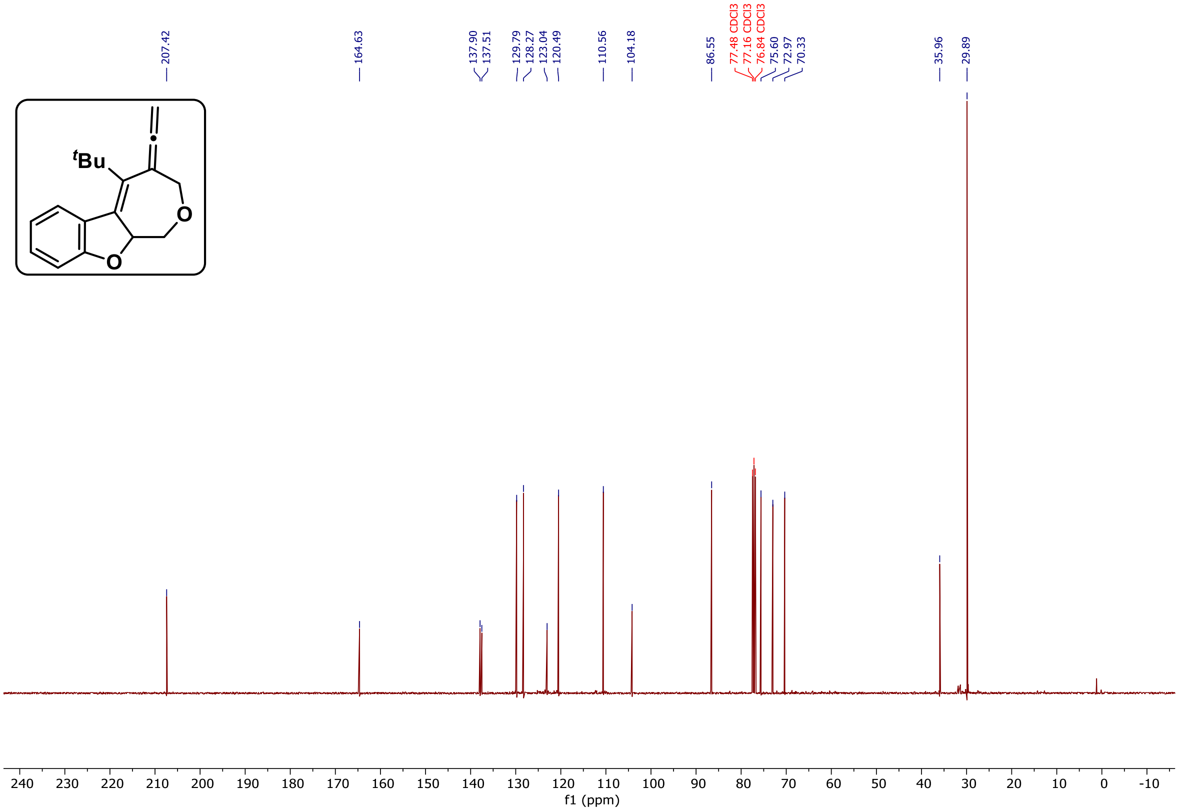

^{13}C-NMR (101 MHz, $CDCl_3$)

5,7-di-*tert*-butyl-4-vinylidene-1,3,4,10a-tetrahydrooxepino[3,4-*b*]benzofuran (2p)

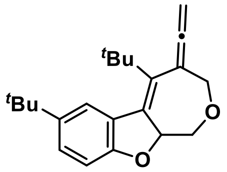

Domino precursor **1p** (55 mg, 0.12 mmol, 1.0 eq.), $Pd_2(dba)_3$ (5.6 mg, 6 µmol, 5.0 mol%), *tert*-Bu-DavePhos (4.2 mg, 12 µmol, 10.0 mol%), and KOAc (89.5 mg, 0.9 mmol, 7.5 eq.) in DMF (9.5 mL, 13 mM) were reacted according to **GP5**. Flash column chromatography (*n*-pentane to *n*-pentane:EtOAc = 100:1) afforded allene product **2p** (26 mg, 80 µmol, 66%) as yellow oil.

$\mathbf{R_f}$ = 0.38 (*n*-pentane:EtOAc = 100:1).

^{1}H-NMR (400 MHz, $CDCl_3$): δ = 7.75 (d, J = 2.0 Hz, 1H), 7.20 (dd, J = 8.5, 2.1 Hz, 1H), 6.73 (d, J = 8.5 Hz, 1H), 5.63 (dd, J = 9.0, 3.4 Hz, 1H), 4.92 – 4.80 (m, 2H), 4.29 (dd, J = 11.3, 0.7 Hz, 1H), 3.98 (dd, J = 11.5, 3.4 Hz, 1H), 3.88 (dt, J = 11.3, 1.7 Hz, 1H), 3.57 (dd, J = 11.5, 9.1 Hz, 1H), 1.39 (s, 9H), 1.33 (s, 9H).

^{13}C-NMR (101 MHz, $CDCl_3$): δ = 207.5, 162.5, 143.2, 137.9, 137.2, 126.9, 125.5, 122.5, 109.6, 104.2, 86.8, 75.5, 73.0, 70.4, 35.9, 34.7, 31.8, 29.9.

IR (ATR): $\tilde{\nu}$ (cm^{-1}) = 2957, 1481, 1464, 1364, 1294, 1262, 1227, 1211, 1122, 1106, 1084, 1065, 1027, 999.

HRMS (APCI, Q-TOF): calculated for $C_{22}H_{29}O_2^+$ $[M+H]^+$: 325.2162, found: 325.2165.

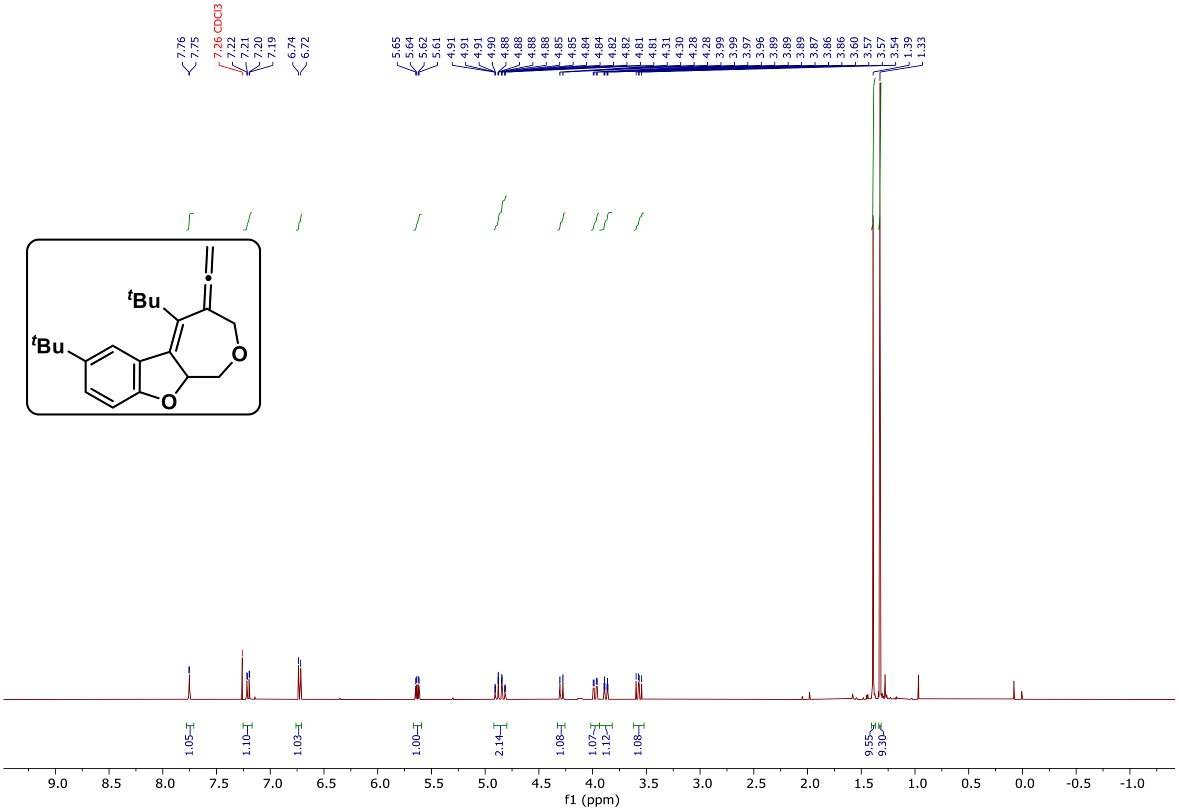

^{1}H-NMR (400 MHz, $CDCl_3$)

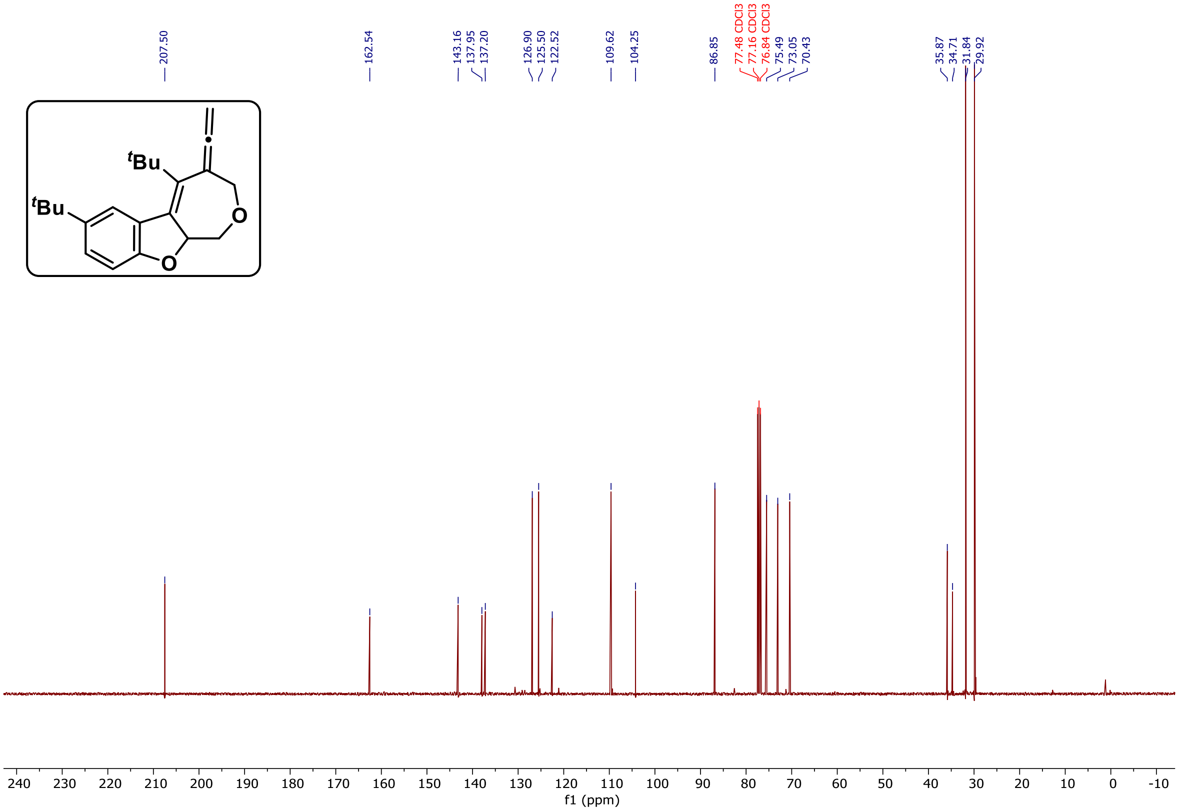

^{13}C-NMR (101 MHz, $CDCl_3$)

5-(*tert*-butyl)-7-nitro-4-vinylidene-1,3,4,10a-tetrahydrooxepino[3,4-*b*]benzofuran (2q)

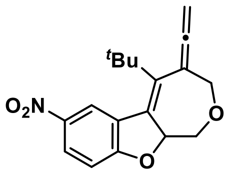

Domino precursor **1q** (38 mg, 0.09 mmol, 1.0 eq.), $Pd_2(dba)_3$ (4 mg, 4 µmol, 5.0 mol%), *tert*-Bu-DavePhos (3.1 mg, 9 µmol, 10.0 mol%), and KOAc (64.3 mg, 0.66 mmol, 7.3 eq.) in DMF (7.5 mL, 12 mM) were reacted according to **GP5**. The reaction was completed in 2h. Flash column chromatography (*n*-pentane:EtOAc = 70:1 to 50:1) afforded allene product **2q** (12.6 mg, 40 µmol, 47%) as yellow oil.

$\mathbf{R_f}$ = 0.37 (*n*-pentane:EtOAc = 50:1).

^{1}H-NMR (700 MHz, $CDCl_3$): δ 8.05 – 8.03 (m, 2H), 6.86 (dd, *J* = 8.5, 1.0 Hz, 1H), 5.13 (ddt, *J* = 12.8, 3.3, 1.6 Hz, 1H), 5.05 (ddt, *J* = 12.9, 3.5, 1.7 Hz, 1H), 4.56 (dd, *J* = 10.2, 5.1 Hz, 1H), 4.43 (ddt, *J* = 10.5, 5.2, 1.9 Hz, 1H), 4.37 (dt, *J* = 12.3, 1.4 Hz, 1H), 4.33 (dt, *J* = 12.4, 3.3 Hz, 1H), 3.94 (t, *J* = 10.3 Hz, 1H), 1.34 (s, 9H).

^{13}C-NMR (176 MHz, $CDCl_3$): *δ* = 207.9, 159.5, 140.2, 138.3, 129.9, 128.4, 125.2, 122.1, 116.6, 99.3, 79.5, 71.7, 70.5, 68.8, 38.4, 32.7.

IR (ATR): $\tilde{\nu}$ (cm^{-1}) = 1724, 1675, 1615, 1580, 1516, 1480, 1427, 1397, 1336, 1246, 1135, 1090, 1048, 1025.

HRMS (APCI, Q-TOF): calculated for $C_{18}H_{20}NO_4^+$ $[M+H]^+$: 314.1387, found: 314.1391.

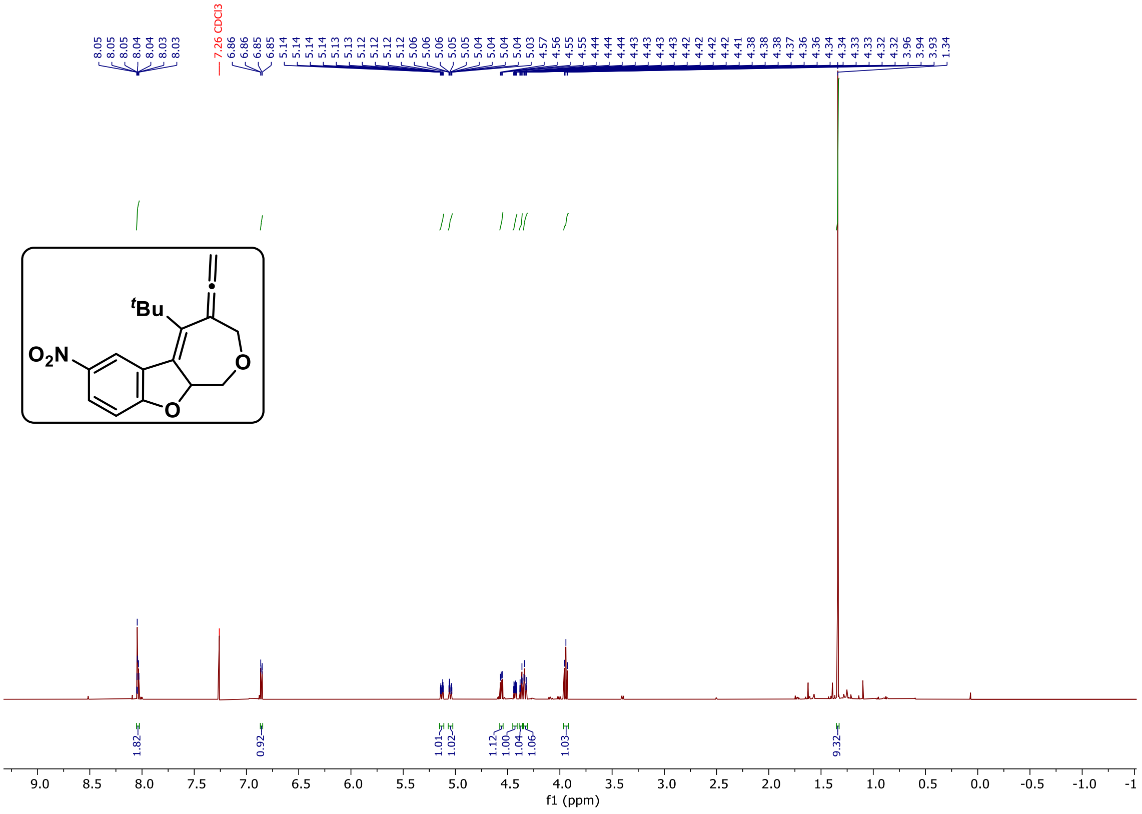

^{1}H-NMR (700 MHz, $CDCl_3$)

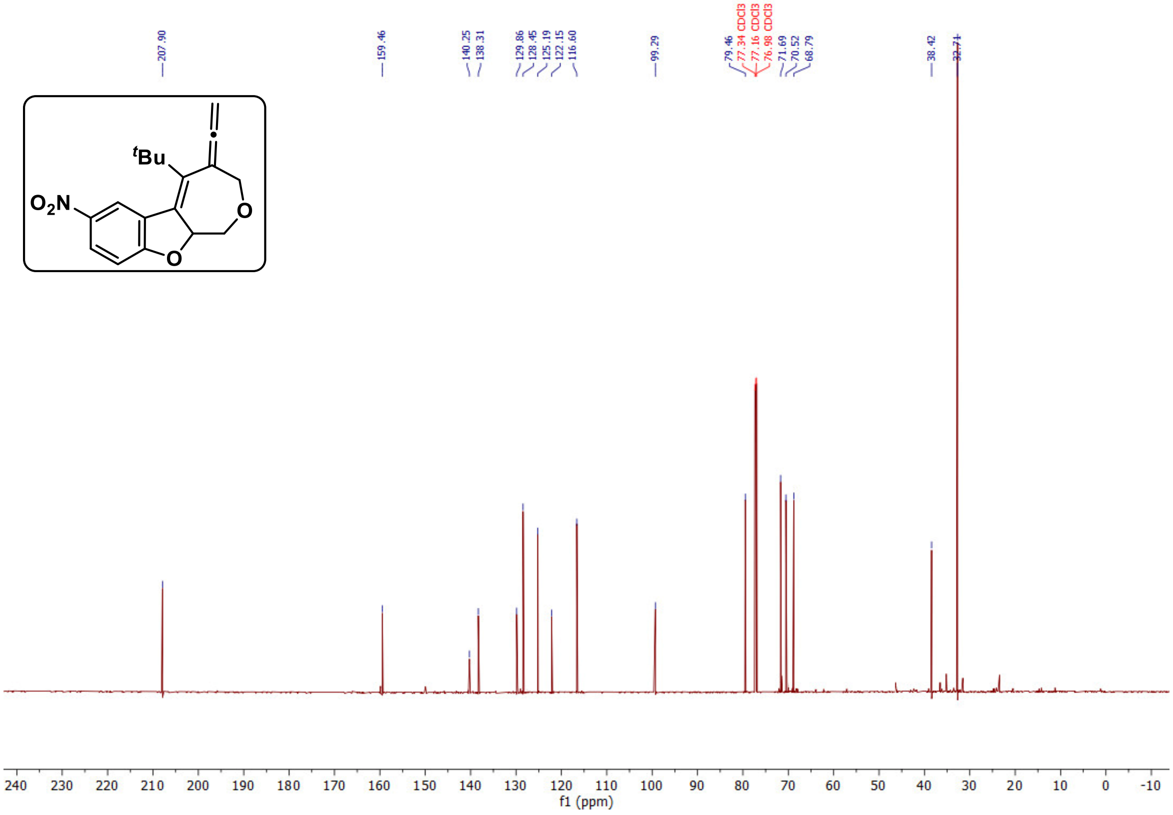

^{13}C-NMR (176 MHz, $CDCl_3$)

5-(*tert*-butyl)-8-methoxy-4-vinylidene-1,3,4,10a-tetrahydrooxepino[3,4-*b*]benzofuran (2r)

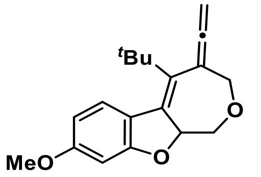

Domino precursor **1r** (45 mg, 0.11 mmol, 1.0 eq.), $Pd_2(dba)_3$ (4.7 mg, 5 µmol, 5.0 mol%), *tert*-Bu-DavePhos (3.6 mg, 10 µmol, 10.0 mol%), and KOAc (80.3 mg, 0.82 mmol, 7.5 eq.) in DMF (9 mL, 12 mM) were reacted according to **GP5**. Flash column chromatography (*n*-pentane:EtOAc = 60:1 to 40:1) afforded allene product **2r** (19.2 mg, 64 µmol, 61%) as yellow oil.

$\mathbf{R_f}$ = 0.4 (*n*-pentane:EtOAc = 40:1).

^{1}H-NMR (500 MHz, $CDCl_3$): δ 7.55 (d, J = 8.7 Hz, 1H), 6.48 (dd, J = 8.7, 2.5 Hz, 1H), 6.35 (d, J = 2.5 Hz, 1H), 5.64 (dd, J = 9.0, 3.3 Hz, 1H), 4.88 (ddd, J = 11.7, 1.5, 0.8 Hz, 1H), 4.81 (ddd, J = 11.6, 2.0, 0.7 Hz, 1H), 4.28 (dd, J = 11.3, 0.8 Hz, 1H), 3.97 (ddd, J = 11.4, 3.4, 0.6 Hz, 1H), 3.89 – 3.82 (m, 1H), 3.79 (s, 3H), 3.56 (dd, J = 11.6, 9.0 Hz, 1H), 1.33 (s, 9H).

^{13}C-NMR (126 MHz, $CDCl_3$): δ = 207.7, 166.2, 161.5, 137.0, 134.6, 128.7, 115.9, 107.3, 104.2, 95.9, 87.5, 75.3, 73.2, 70.4, 55.6, 35.8, 29.9.

IR (ATR): $\tilde{\nu}$ (cm^{-1}) = 2921, 1945, 1614, 1493, 1444, 1332, 1274, 1236, 1193, 1157, 1093, 1066, 1031.

HRMS (APCI, Q-TOF): calculated for $C_{19}H_{23}O_3^+$ $[M+H]^+$: 299.1642, found: 299.1643.

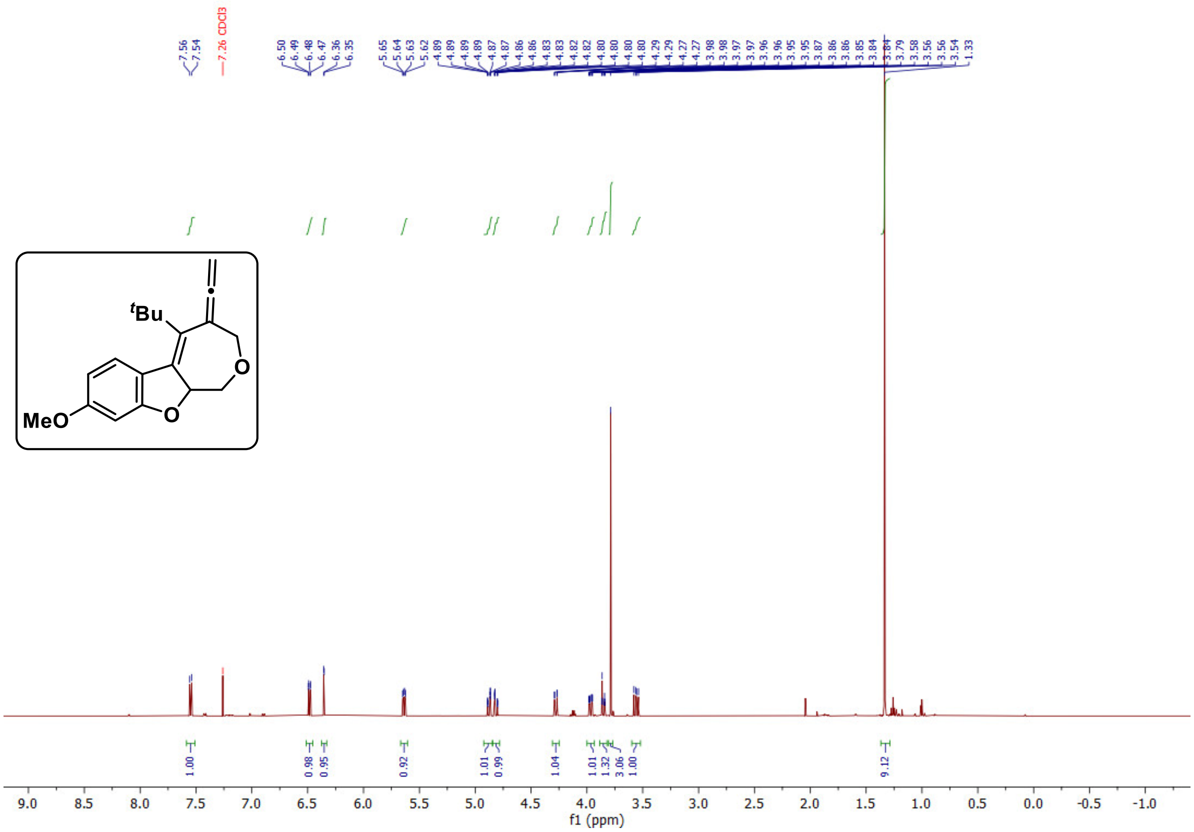

^{1}H-NMR (500 MHz, $CDCl_3$)

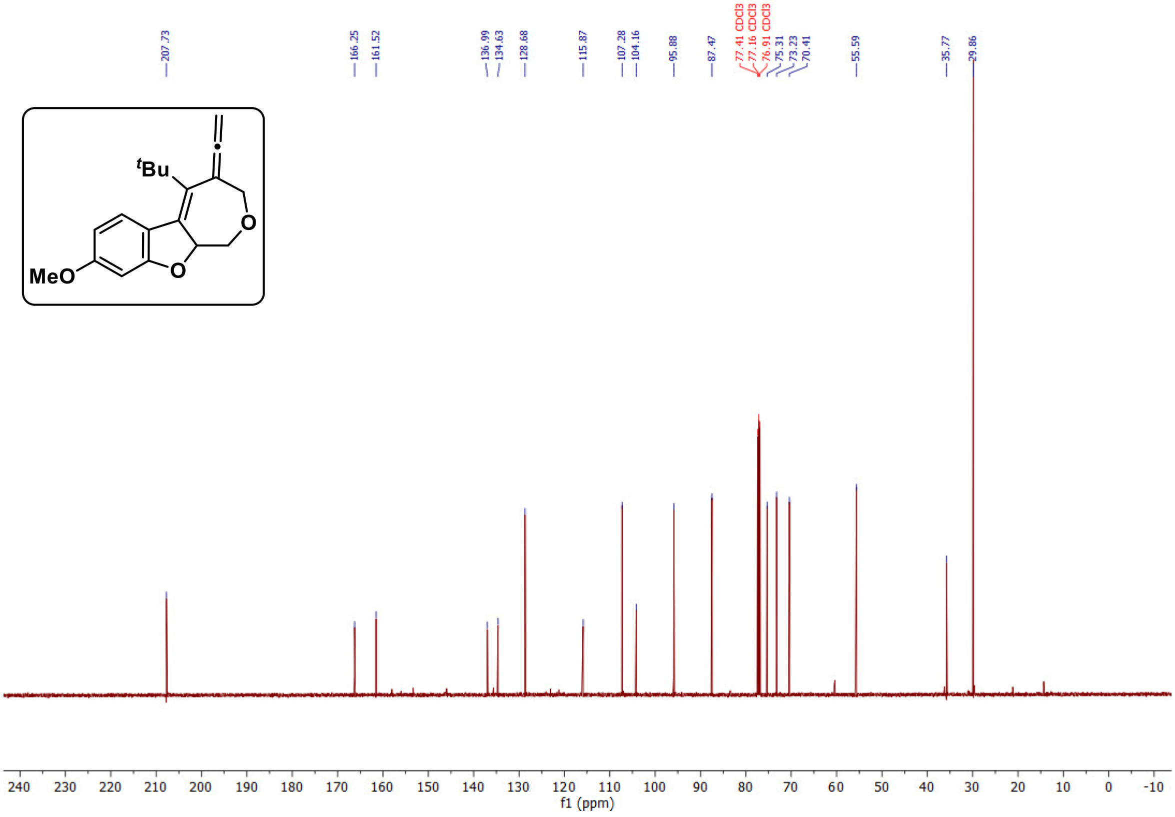

^{13}C-NMR (126 MHz, $CDCl_3$)

5-(*tert*-butyl)-4-vinylidene-1,3,4,12a-tetrahydronaphtho[2',3':4,5]furo[2,3-*c*]oxepine (2s)

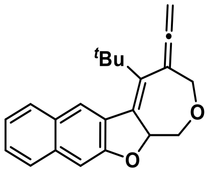

Domino precursor **1s** (40 mg, 0.1 mmol, 1.0 eq.), $Pd_2(dba)_3$ (4.6 mg, 5 µmol, 5.0 mol%), *tert*-Bu-DavePhos (3.5 mg, 10 µmol, 10.0 mol%), and KOAc (73.5 mg, 0.75 mmol, 7.5 eq.) in DMF (8 mL, 12 mM) were reacted according to **GP5**. Flash column chromatography (*n*-pentane:EtOAc = 80:1 to 40:1) afforded allene product **2s** (17.0 mg, 53 µmol, 53%) as yellow oil.

$\mathbf{R_f}$ = 0.41 (*n*-pentane:EtOAc = 60:1).

^{1}**H-NMR** (400 MHz, $CDCl_3$): δ = 7.73 (ddq, J = 8.2, 1.3, 0.7 Hz, 1H), 7.67 (ddq, J = 8.3, 1.3, 0.7 Hz, 1H), 7.58 (s, 1H), 7.40 (ddd, J = 8.2, 6.8, 1.3 Hz, 1H), 7.31 (ddd, J = 8.1, 6.8, 1.3 Hz, 1H), 7.18 (d, J = 0.8 Hz, 1H), 5.14 – 5.01 (m, 2H), 4.52 – 4.45 (m, 2H), 4.43 – 4.32 (m, 2H), 4.03 – 3.95 (m, 1H), 1.33 (s, 9H).

^{13}**C-NMR** (101 MHz, $CDCl_3$): δ = 207.6, 153.1, 136.8, 134.8, 132.5, 131.4, 128.0, 127.8, 126.8, 126.5, 125.0, 124.1, 110.7, 99.6, 79.0, 73.6, 70.6, 68.7, 38.3, 32.7.

IR (ATR): $\tilde{\nu}$ (cm^{-1}) = 1258, 1234, 1220, 1167, 1144, 1135, 1107, 1059, 1049, 1031, 1015, 997.

HRMS (APCI, Q-TOF): calculated for $C_{22}H_{23}O_2^+$ [M+H]$^+$: 319.1693, found: 319.1694.

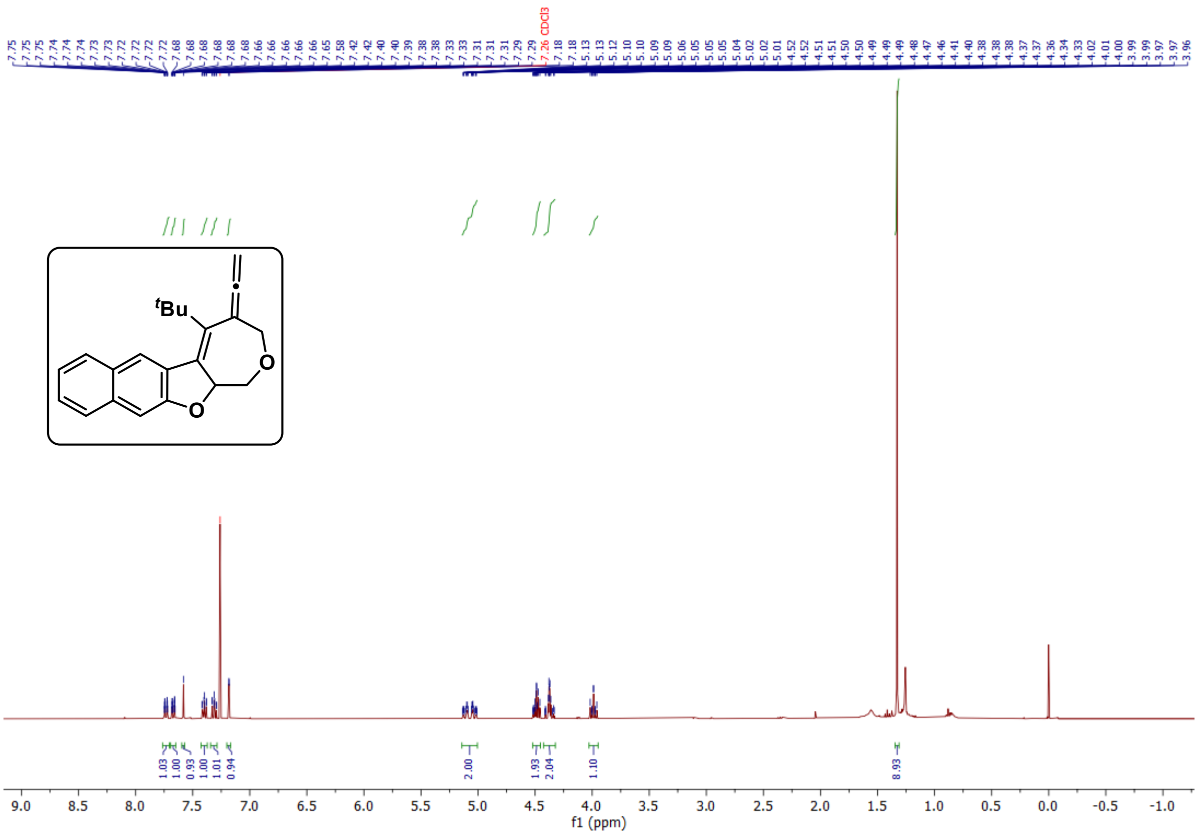

^{1}H-NMR (400 MHz, $CDCl_3$)

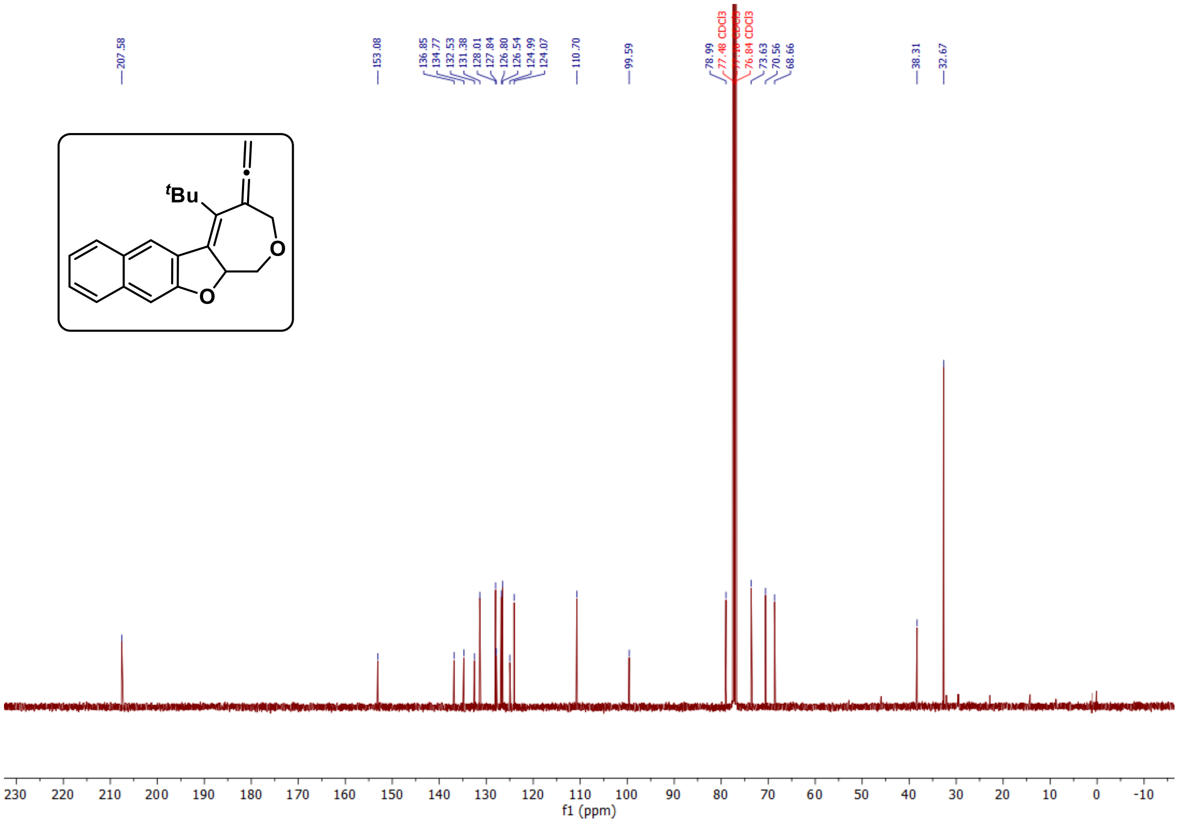

^{13}C-NMR (101 MHz, $CDCl_3$)

5-(*tert*-butyl)-4-((*R*/*S*)-prop-1-en-1-ylidene)-1,3,4,10a-tetrahydrooxepino[3,4-*b*]benzofuran (2t)

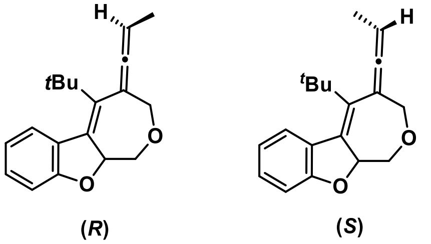

Domino precursor **1t** (61.5 mg, 0.15 mmol, 1.0 eq.), $Pd_2(dba)_3$ (6.9 mg, 7 µmol, 5.0 mol%), *tert*-Bu-DavePhos (5.1 mg, 15 µmol, 10.0 mol%), and KOAc (110.2 mg, 1.12 mmol, 7.5 eq.) in DMF (12 mL, 12 mM) were reacted according to **GP5**. Flash column chromatography (*n*-pentane:EtOAc = 80:1 to 60:1) afforded allene product **2t** (26.9 mg, 95 µmol, 64%) as yellow oil.

$\mathbf{R_f}$ = 0.25 (*n*-pentane:EtOAc = 80:1).

^{1}**H-NMR** (700 MHz, $CDCl_3$): δ = 7.67 (ddd, *J* = 7.9, 4.4, 1.3 Hz, 2H), 7.15 (dddd, *J* = 8.1, 7.3, 5.0, 1.3 Hz, 2H), 6.91 (dddd, *J* = 7.9, 7.3, 4.0, 1.2 Hz, 2H), 6.81 – 6.77 (m, 2H), 5.67 (dd, *J* = 8.9, 3.5 Hz, 1H), 5.62 (dd, *J* = 9.0, 3.5 Hz, 1H), 5.28 (qdd, *J* = 7.2, 1.4, 0.8 Hz, 1H), 5.20 (qdd, *J* = 7.0, 1.8, 0.7 Hz, 1H), 4.24 (dt, *J* = 11.4, 0.8 Hz, 1H), 4.22 – 4.20 (m, 1H), 4.00 – 3.94 (m, 2H), 3.85 (ddd, *J* = 11.3, 2.6, 1.6 Hz, 2H), 3.57 (ddd, *J* = 17.9, 11.6, 8.9 Hz, 2H), 1.73 (d, *J* = 7.0 Hz, 3H), 1.72 (d, *J* = 7.2 Hz, 3H), 1.37 (s, 9H), 1.35 (s, 9H).

^{13}**C-NMR** (176 MHz, $CDCl_3$): δ = 203.9, 203.4, 164.6, 164.6, 139.1, 138.9, 136.8, 136.8, 129.6, 129.6, 128.3, 128.2 123.2, 123.1, 120.5, 120.4, 110.5, 104.4, 103.8, 86.8, 86.6, 86.5, 86.0, 73.3, 73.3, 70.3, 70.2, 35.9, 34.9, 30.1, 29.9, 14.4, 13.6.

IR (ATR): $\tilde{\nu}$ (cm^{-1}) = 2919, 1592, 1458, 1365, 1225, 1104, 1065, 1033.

HRMS (APCI, Q-TOF): calculated for $C_{19}H_{23}O_2^+$ $[M+H]^+$: 283.1693, found: 283.1692.

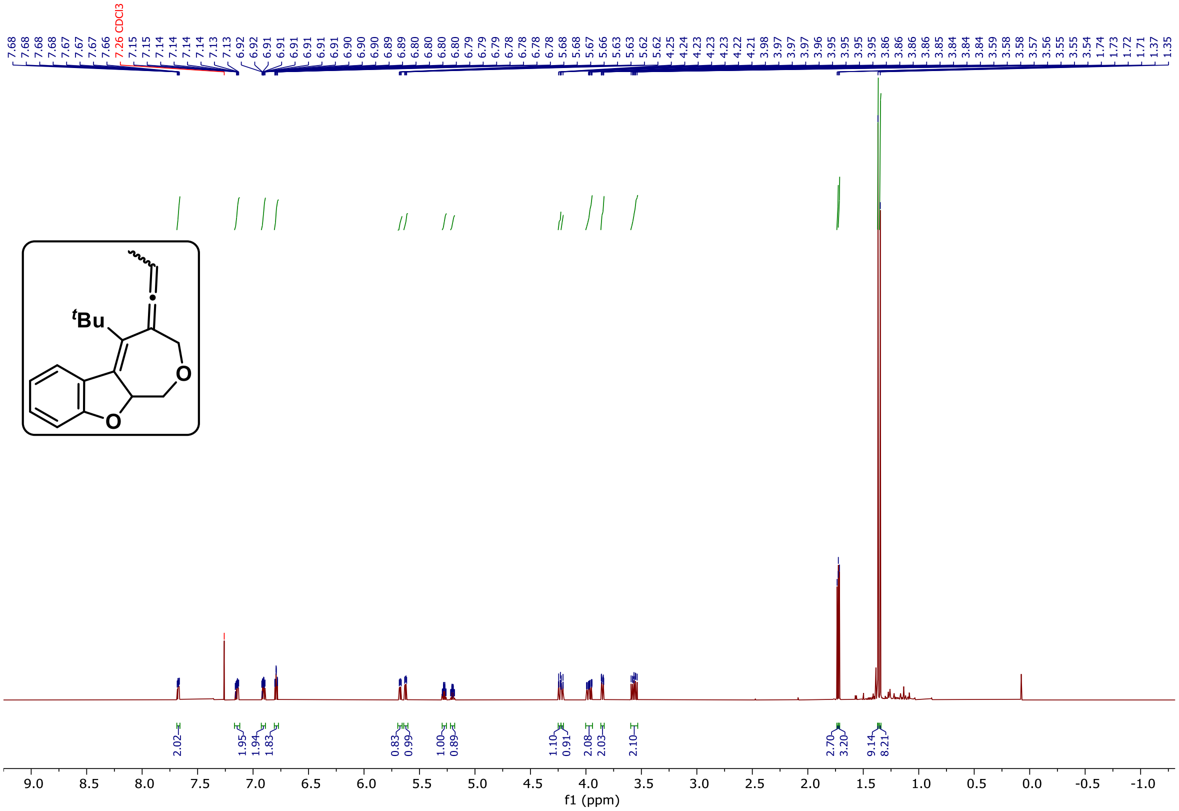

^{1}H-NMR (700 MHz, $CDCl_3$)

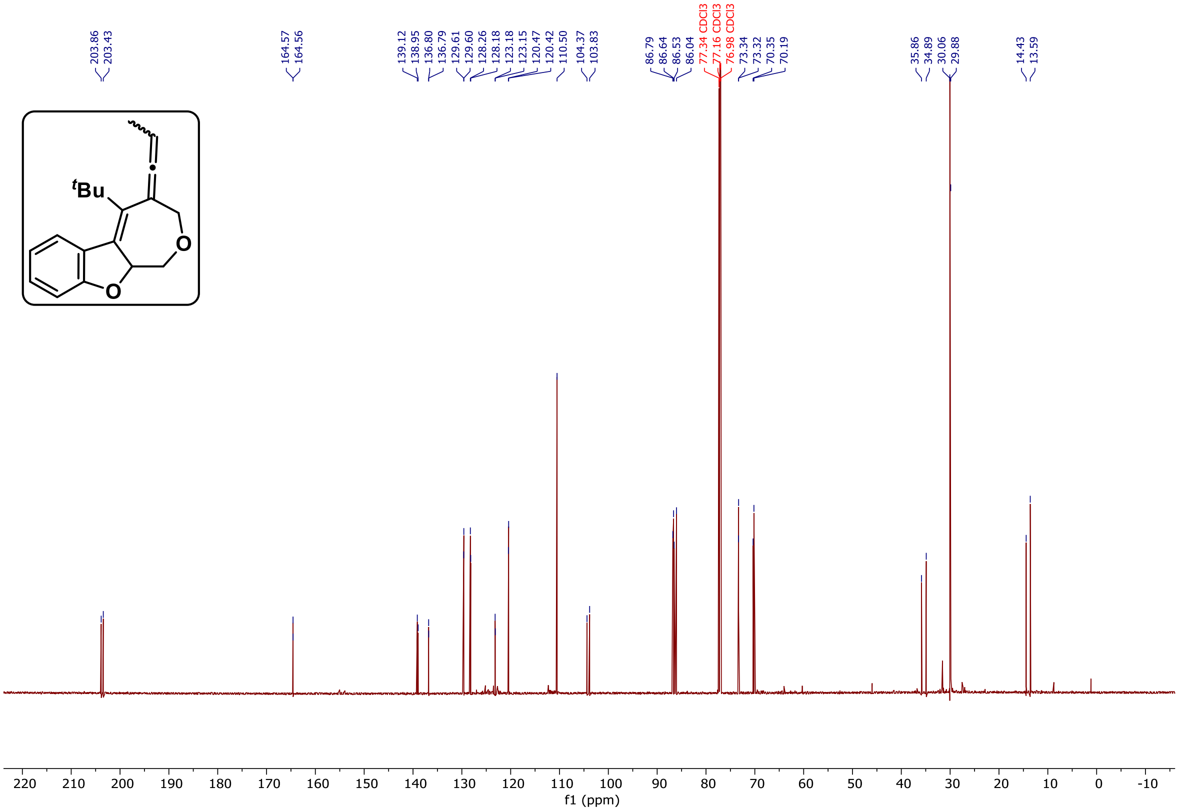

^{13}C-NMR (176 MHz, $CDCl_3$)

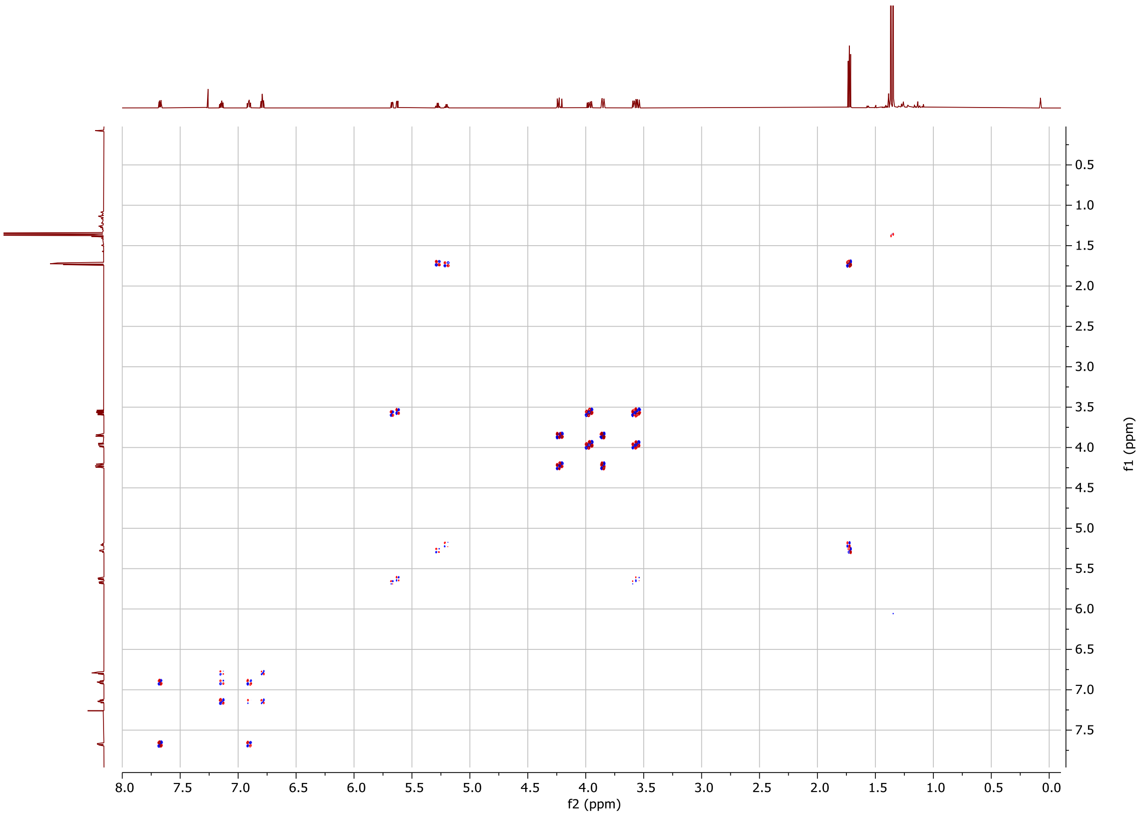

DQF-COSY for compound **2t**

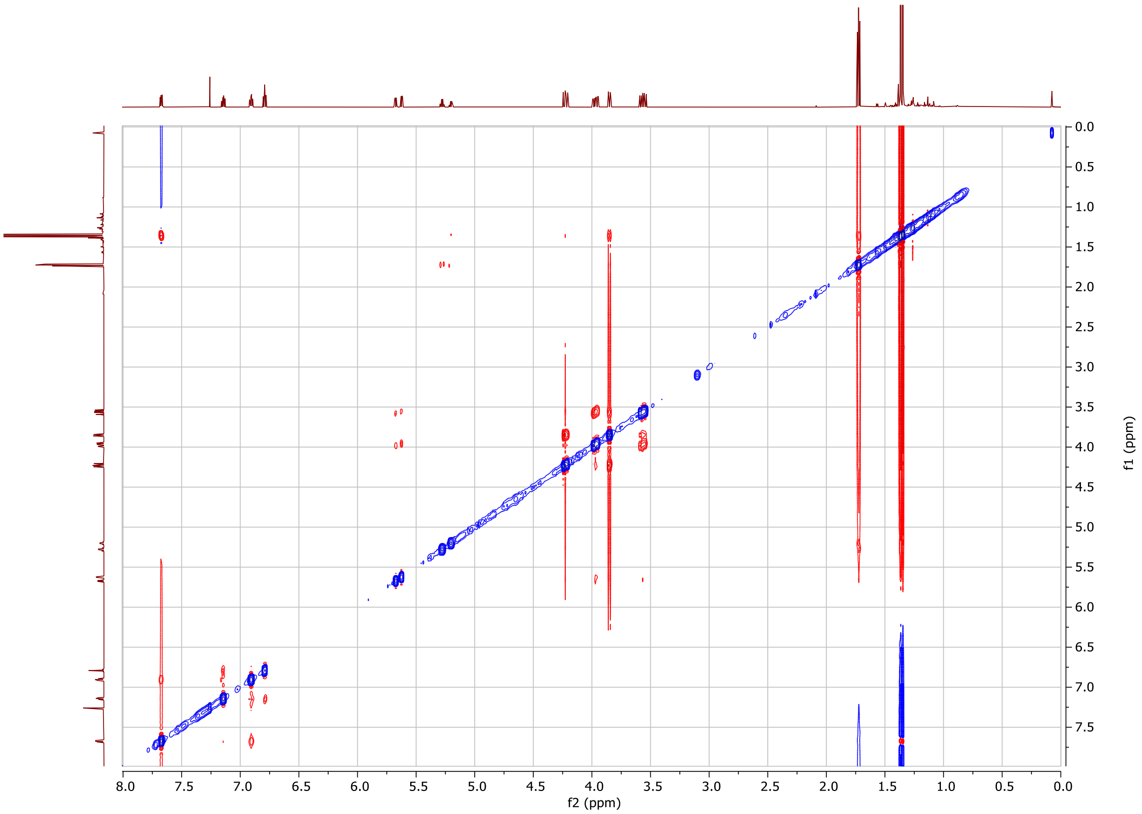

NOESY = 600msec for compound **2t**

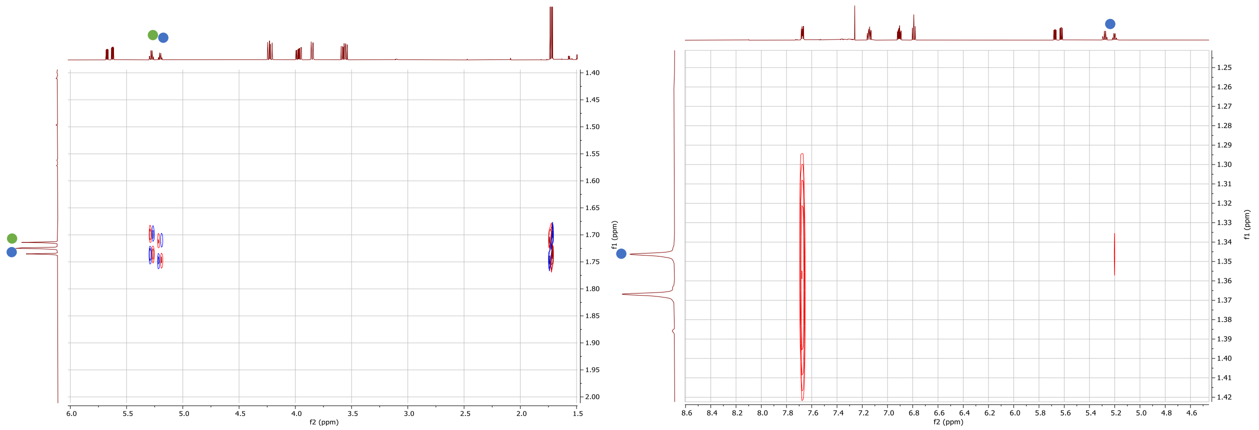

Closer view to DQF-COSY (left) and NOESY = 600msec (right).

According to DQF-COSY:

There is a correlation between **H** (δ = 5.28 ppm) and **CH_3** (δ = 1.72 ppm) in one isomer. Also, a correlation between **H** (δ = 5.20 ppm) and **CH_3** (δ = 1.73 ppm) in another isomer.

Based on NOESY = 600msec:

There is a spatial proximity of **H** (δ = 5.20 ppm) and **^{t}Bu** group (δ = 1.34 ppm) in one isomer, which cannot be found in another.

In addition, the ratio of (*R*)/(S) is 53% to 47%. This amount is measured based on the integration of allene hydrogens in two isomers.

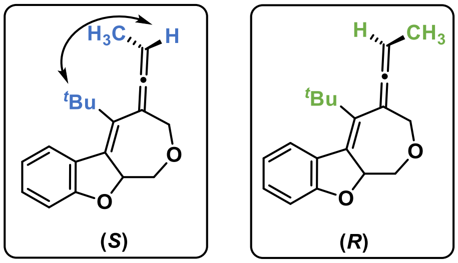

5-(*tert*-butyl)-4-(2-methylprop-1-en-1-ylidene)-1,3,4,10a-tetrahydrooxepino[3,4-*b*]benzofuran (2u)

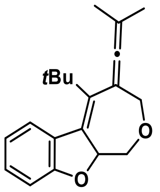

Domino precursor **1u** (63.6 mg, 0.15 mmol, 1.0 eq.), $Pd_2(dba)_3$ (6.9 mg, 7 µmol, 5.0 mol%), *tert*-Bu-DavePhos (5.1 mg, 15 µmol, 10.0 mol%), and KOAc (110.2 mg, 1.12 mmol, 7.5 eq.) in DMF (12 mL, 12 mM) were reacted according to **GP5**. Flash column chromatography (*n*-pentane:EtOAc = 100:1 to 70:1) afforded allene product **2u** (26.4 mg, 89 µmol, 59%) as colorless oil.

$\mathbf{R_f}$ = 0.37 (*n*-pentane:EtOAc = 70:1).

^{1}H-NMR (700 MHz, $CDCl_3$): δ = 7.67 (dd, J = 7.9, 1.3 Hz, 1H), 7.14 (ddd, J = 8.0, 7.3, 1.3 Hz, 1H), 6.90 (ddd, J = 7.9, 7.3, 1.1 Hz, 1H), 6.79 (ddd, J = 8.0, 1.2, 0.4 Hz, 1H), 5.64 (dd, J = 8.8, 3.6 Hz, 1H), 4.19 – 4.15 (m, 1H), 3.98 – 3.93 (m, 1H), 3.82 (d, J = 11.2 Hz, 1H), 3.56 (dd, J = 11.6, 8.8 Hz, 1H), 1.76 (s, 3H), 1.74 (s, 3H), 1.35 (s, 9H).

^{13}C-NMR (176 MHz, $CDCl_3$): δ = 200.5, 164.5, 139.9, 136.2, 129.4, 128.2, 123.3, 120.4, 110.4, 102.6, 95.6, 86.9, 73.6, 70.2, 35.0, 30.0, 21.0, 19.7.

IR (ATR): $\tilde{\nu}$ (cm^{-1}) = 2911, 1582, 1458, 1363, 1304, 1227, 1211, 1152, 1106, 1053, 1032.

HRMS (APCI, Q-TOF): calculated for $C_{20}H_{25}O_2^+$ $[M+H]^+$: 297.1849, found: 297.1851.

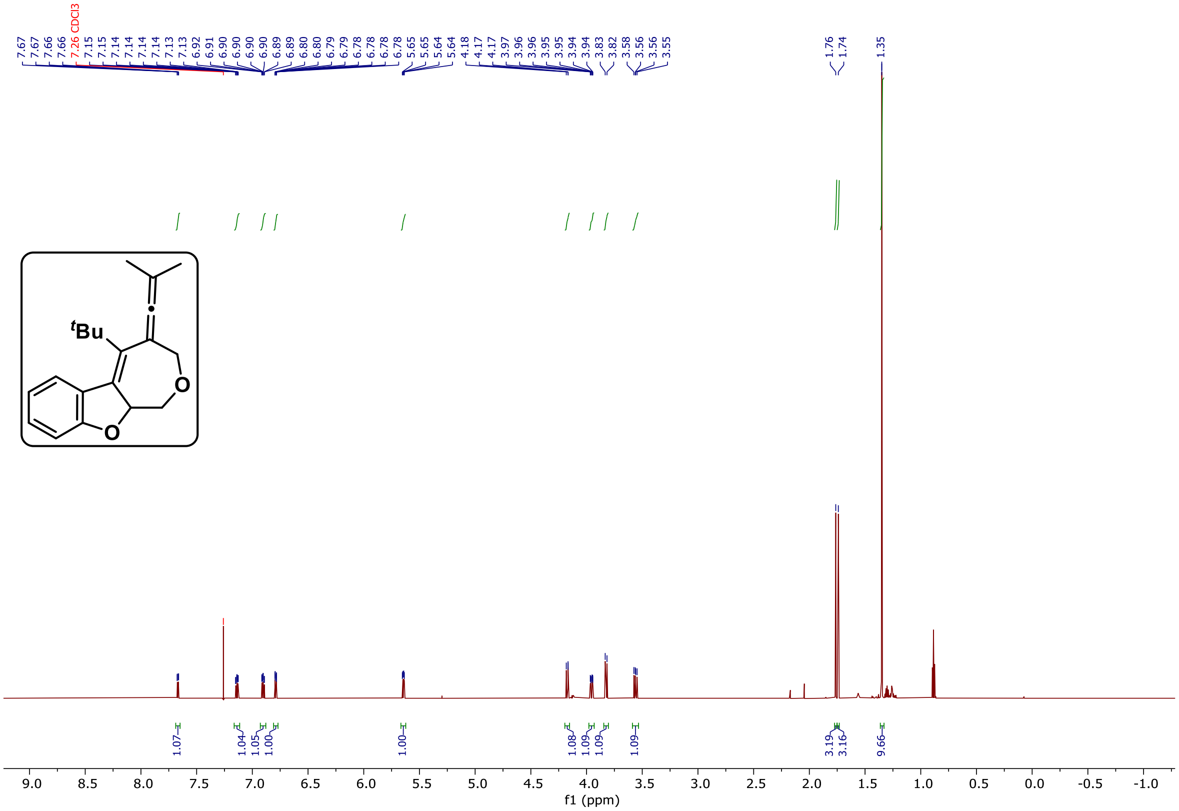

^{1}H-NMR (700 MHz, $CDCl_3$)

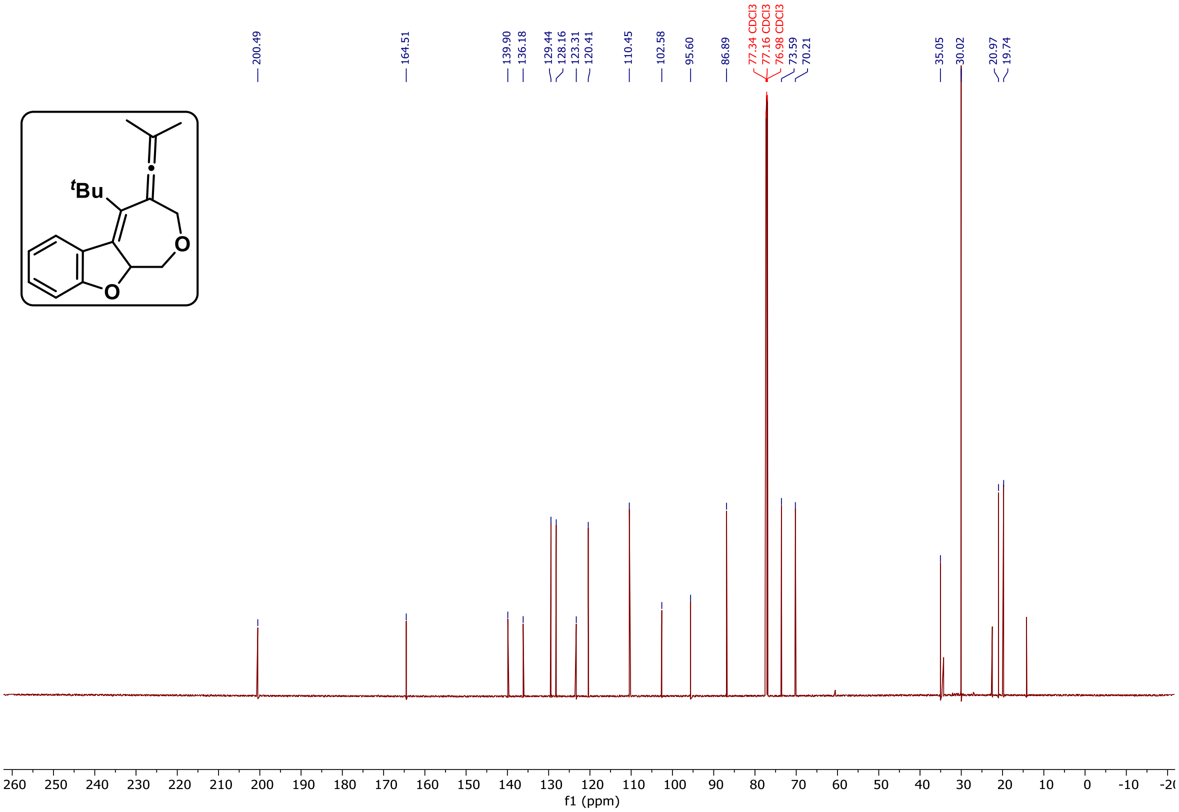

^{13}C-NMR (176 MHz, $CDCl_3$)

6. Supplementary Data for DFT Calculations

Methods

All calculations were performed using the Gaussian 16 software.[11] All structures were optimised using the hybrid PBE1PBE functional,[12] def2-SVP as basis set,[13] and D3 as dispersion correction.[14] All optimized structures were confirmed as minima by frequency analyses. Single point calculations were subsequently performed on the optimized structures at a PBE1PBE/def2-TZVP level of theory. Solvent effects were taken into account using the SMD implicit solvation model with *N*,*N*-dimethylformamide as solvent.[15]
For all thermochemical calculations, the temperature was set to 298°K.

Proposed Mechanism 1

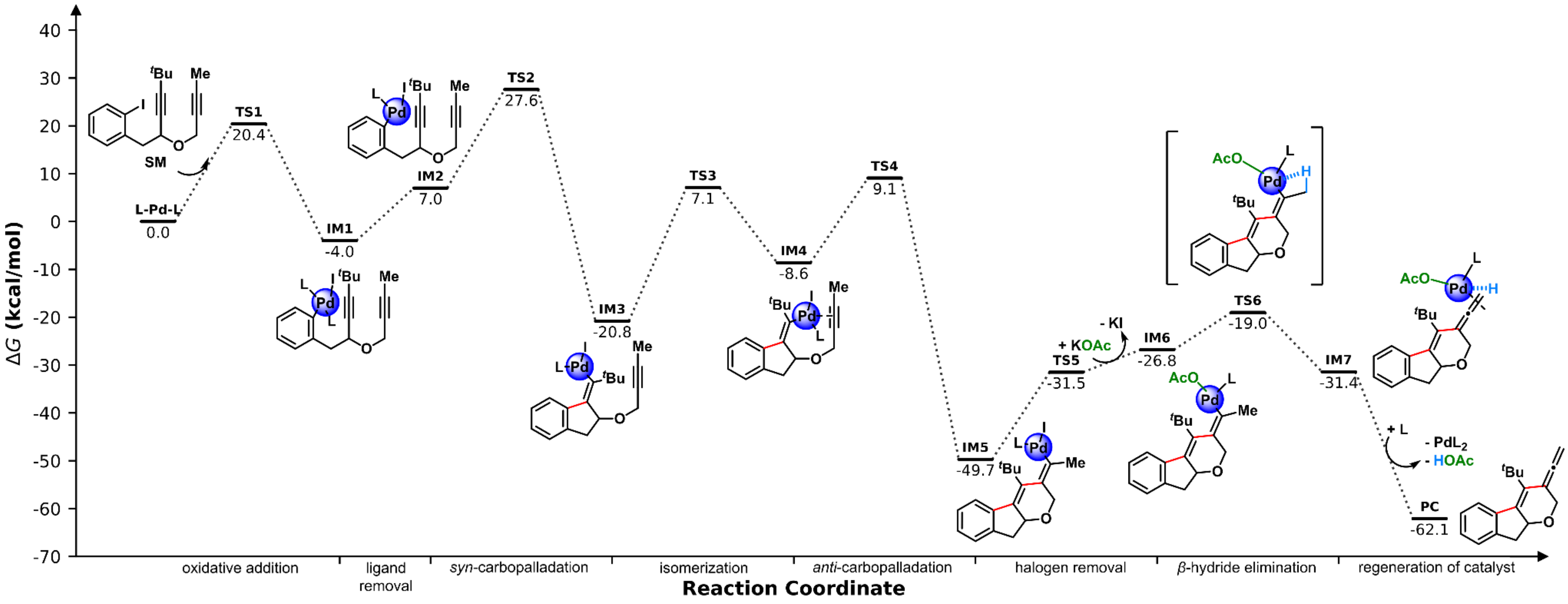

Proposed Mechanism 2

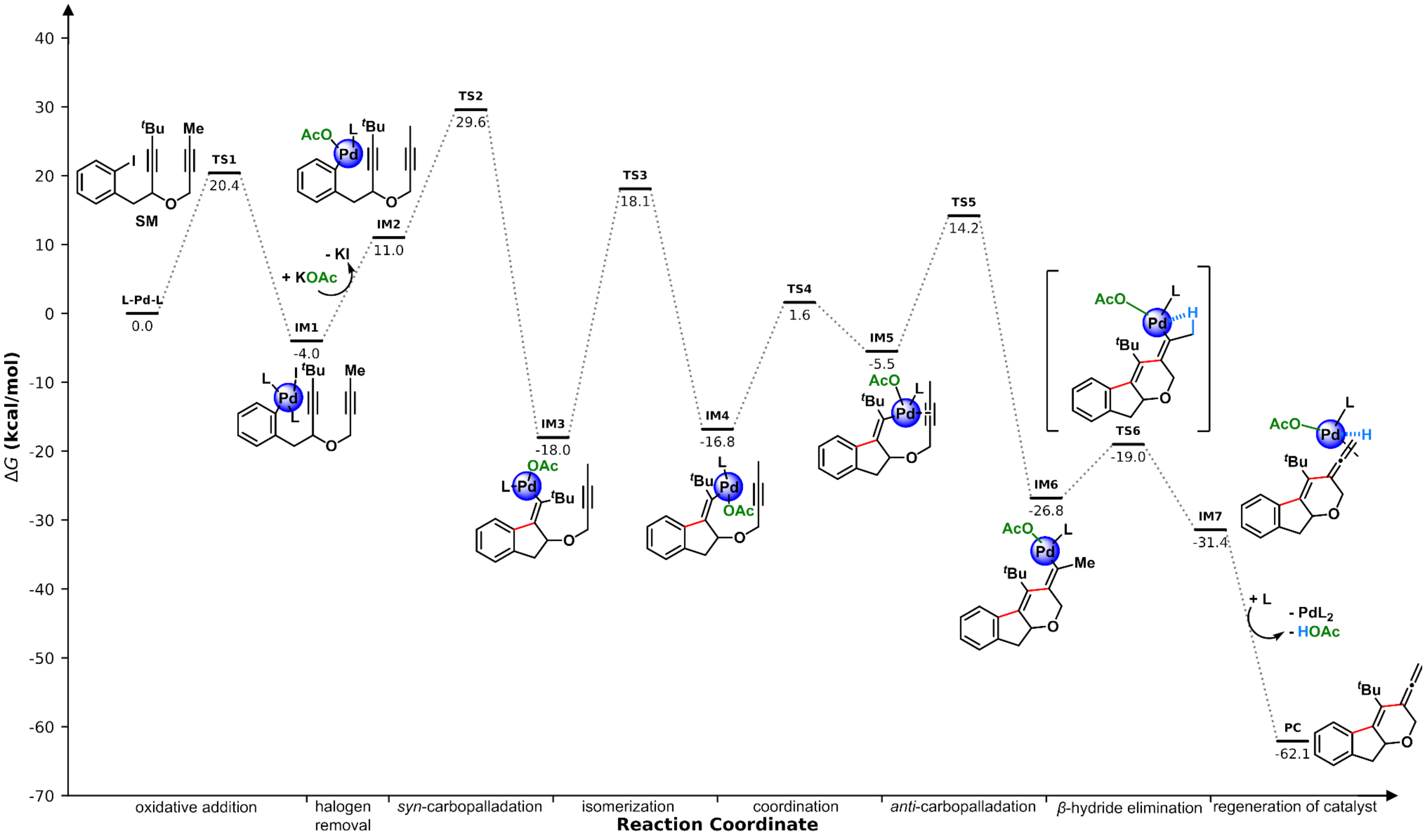

Proposed Mechanism 1	Correction Gibbs (Hartree)	SCF (Hartree)	Gibbs Energy (kcal/mol)	ΔG (kcal/mol)	ΔΔG (kcal/mol)
SM	0.2832	-1070.7816	-671747.9407	0.0	20.4
PdL_2	0.6693	-1756.7874	-1101980.7988	0.0	
TS1	0.6301	-2013.1555	-1262878.7893	20.4	
IM1	0.6344	-2013.1986	-1262903.1713	-4.0	
IM2	0.6389	-2013.1857	-1262892.2105	7.0	20.6
TS2	0.6370	-2013.1508	-1262871.5527	27.6	
IM3	0.6359	-2013.2270	-1262920.0291	-20.8	27.9
TS3	0.6362	-2013.1828	-1262892.1029	7.1	
IM4	0.6445	-2013.2171	-1262907.7449	-8.6	17.7
TS4	0.6442	-2013.1876	1262890.0778	9.1	
IM5	0.6444	-2013.2815	-1262948.9202	-49.7	18.2
TS5	0.6458	-2013.2538	-1262930.6494	-31.5	
IM6	0.6942	-1943.8319	-1219337.3420	-26.8	20.2
TS6	0.6904	-1943.8157	-1219329.5854	-19.0	
IM7	0.6904	-1943.8356	-1219342.0198	-31.4	
PC	0.2878	-772.5130	-484578.6442	-62.1	
L	0.3268	-814.3855	-510829.5594	-	
KOAc	0.0173	-828.1703	-519673.8682	-	
KI	-0.0256	-897.5903	-563262.4757	-	
HOAc	0.0349	-228.9443	-143642.8019	-	

Proposed Mechanism 2	Correction Gibbs (Hartree)	SCF (Hartree)	Gibbs Energy (kcal/mol)	ΔG (kcal/mol)	ΔΔG (kcal/mol)
SM	0.2832	-1070.7816	-671747.9407	0.0	20.4
PdL_2	0.6693	-1756.7874	-1101980.7988	0.0	
TS1	0.6301	-2013.1555	-1262878.7893	20.4	
IM1	0.6344	-2013.1986	-1262903.1713	-4.0	
IM2_OAc	0.6890	-1943.7764	-1219299.5308	11.0	18.6
TS2_OAc	0.6850	-1943.7328	-1219280.9926	29.6	
IM3_OAc	0.6904	-1943.8141	-1219328.5557	-18.0	36.1
TS3_OAc	0.6854	-1943.7515	-1219292.4238	18.1	
IM4_OAc	0.6948	-1943.8166	-1219327.3939	-16.8	18.4
TS4_OAc	0.6950	-1943.7875	-1219309.0093	1.6	
IM5_OAc	0.6943	-1943.7980	-1219316.0276	-5.5	19.7
TS5_OAc	0.6964	-1943.7687	-1219296.3277	14.2	
IM6	0.6942	-1943.8319	-1219337.3420	-26.8	20.2
TS6	0.6904	-1943.8157	-1219329.5854	-19.0	
IM7	0.6904	-1943.8356	-1219342.0198	-31.4	
PC	0.2878	-772.5130	-484578.6442	-62.1	
L	0.3268	-814.3855	-510829.5594	-	
KOAc	0.0173	-828.1703	-519673.8682	-	
KI	-0.0256	-897.5903	-563262.4757	-	
HOAc	0.0349	-228.9443	-143642.8019	-	

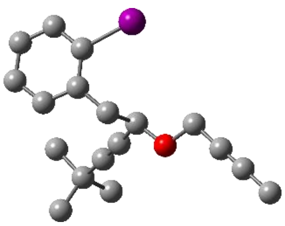

SM

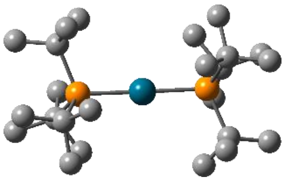

PdL$_2$

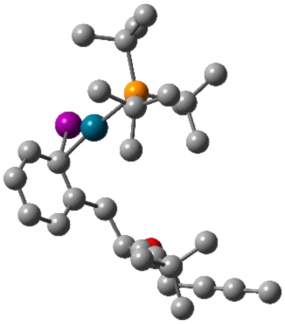

TS1

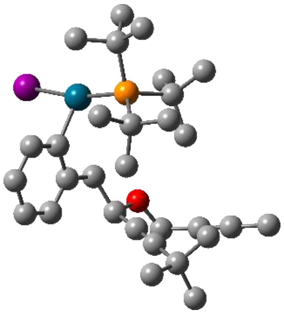

IM1

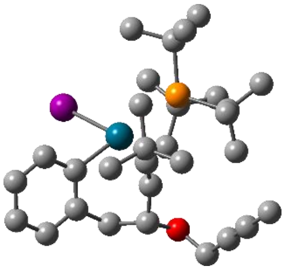

IM2

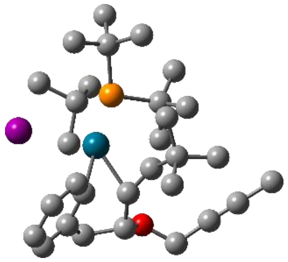

TS2

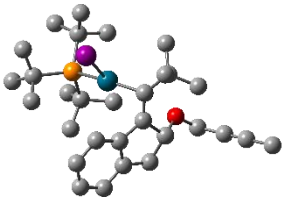

IM3

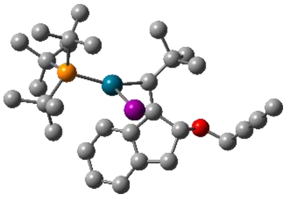

TS3

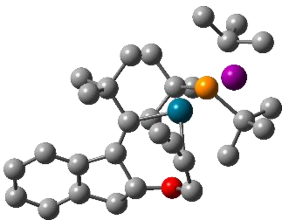

IM4

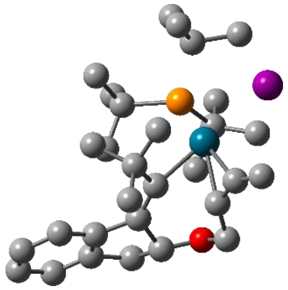

TS4

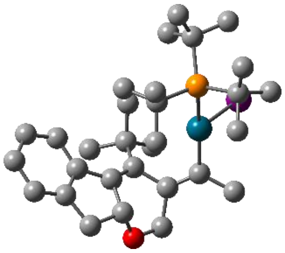

IM5

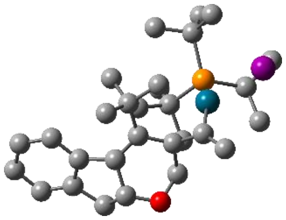

TS5

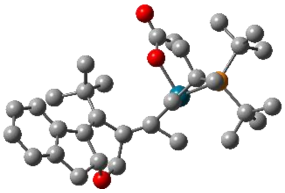

IM6

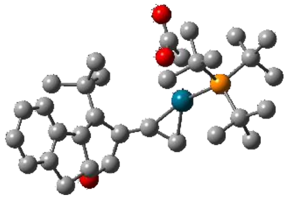

TS6

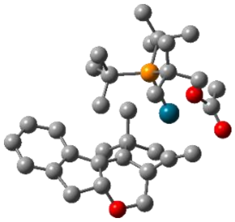

IM7

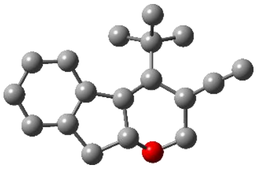

PC

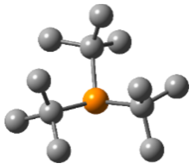

L

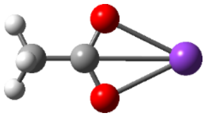

KOAc

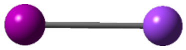

KI

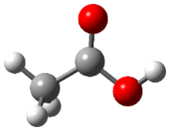

HOAc

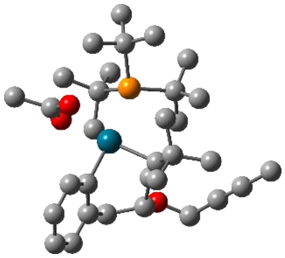

IM2_OAc

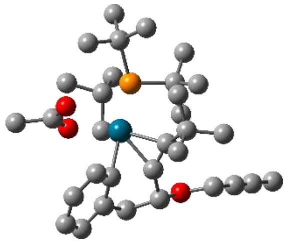

TS2_OAc

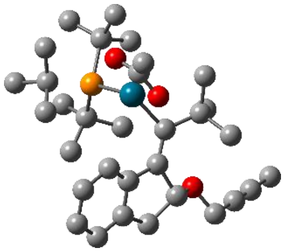

IM3_OAc

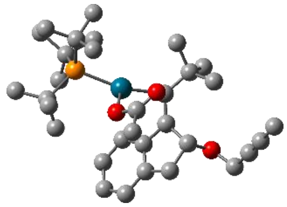

TS3_OAc

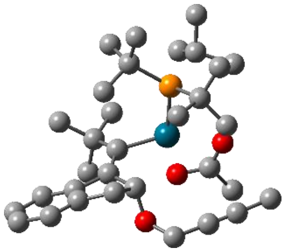

IM4_OAc

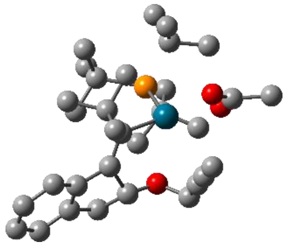

TS4_OAc

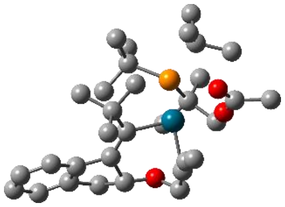

IM5_OAc

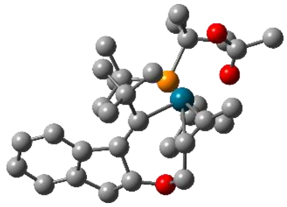

TS5_OAc

DFT optimized cartesian coordinates

SM

E(pbe1pbe) = -2013.19858825 a.u.

Lowest Freq. = 16.33 cm^{-1}

C	2.9361	-1.9509	1.4660
C	1.9551	-0.9770	1.6284
C	0.6796	-1.1037	1.0561
C	0.4291	-2.2663	0.3096
C	1.4019	-3.2535	0.1384
C	2.6595	-3.0929	0.7161
H	3.9175	-1.8172	1.9274
H	2.1716	-0.0810	2.2163
H	1.1768	-4.1489	-0.4447
H	3.4184	-3.8675	0.5801
C	-0.3150	0.0064	1.2211
H	-1.3258	-0.4057	1.3618
H	-0.0668	0.6039	2.1100
C	-0.3598	0.9613	0.0107
H	-0.5875	0.3653	-0.8954
O	-1.3569	1.9444	0.1937
C	-2.6306	1.5238	-0.2404
H	-2.9218	0.5650	0.2313
H	-2.6271	1.3442	-1.3342
C	-3.6275	2.5360	0.0867
C	-4.4691	3.3681	0.3566
C	-5.4777	4.3626	0.6797
H	-5.4223	5.2256	-0.0024
H	-6.4892	3.9335	0.6003
H	-5.3510	4.7336	1.7091
C	0.9308	1.6187	-0.1990
C	2.0345	2.1003	-0.3548
C	3.3828	2.6581	-0.5369
C	3.8371	3.3177	0.7721
C	4.3386	1.5133	-0.9019
C	3.3562	3.6975	-1.6651
H	3.1598	4.1366	1.0592
H	3.8627	2.5871	1.5951
H	4.8493	3.7348	0.6501
H	4.0306	1.0228	-1.8379
H	5.3583	1.9071	-1.0378
H	4.3660	0.7511	-0.1079
H	4.3638	4.1177	-1.8109
H	3.0315	3.2440	-2.6141
H	2.6701	4.5244	-1.4258
I	-1.4547	-2.6066	-0.5913

PdL$_2$

E(pbe1pbe) = -1756.78736760 a.u.

Lowest Freq. = 8.81 cm^{-1}

Pd	0.0001	-0.0158	0.0003
P	2.3151	-0.0021	-0.0004
P	-2.3149	-0.0019	-0.0002
C	2.9768	1.4224	1.1045
C	2.9906	0.2496	-1.7795
C	3.0027	-1.6620	0.6763
C	-2.9766	1.7553	-0.3989
C	-2.9944	-0.5283	1.7168
C	-2.9988	-1.2182	-1.3191
C	2.7379	2.7533	0.3846
H	1.6935	2.8518	0.0488
H	2.9357	3.5733	1.0954
H	3.4013	2.9039	-0.4768
C	4.4497	1.3305	1.5020
H	4.7179	2.2271	2.0874
H	4.6605	0.4589	2.1364
H	5.1212	1.2937	0.6341
C	2.1089	1.4770	2.3698
H	2.3906	2.3705	2.9533
H	1.0414	1.5586	2.1074
H	2.2297	0.6059	3.0235
C	2.1178	1.3081	-2.4691
H	2.2160	2.3097	-2.0354
H	2.4169	1.3762	-3.5293
H	1.0537	1.0238	-2.4293
C	4.4599	0.6576	-1.8831
H	4.7421	0.7095	-2.9491
H	4.6511	1.6497	-1.4517
H	5.1345	-0.0615	-1.4004
C	2.7744	-1.0416	-2.5745
H	1.7322	-1.3912	-2.5040
H	2.9837	-0.8339	-3.6375
H	3.4416	-1.8568	-2.2668
C	4.4783	-1.9406	0.3905
H	4.7641	-2.8875	0.8806
H	4.6848	-2.0597	-0.6819
H	5.1402	-1.1569	0.7817
C	2.7731	-1.7093	2.1900
H	2.9877	-2.7326	2.5416
H	3.4303	-1.0308	2.7490
H	1.7267	-1.4833	2.4492
C	2.1466	-2.7968	0.0952
H	2.2553	-2.9190	-0.9880
H	2.4513	-3.7464	0.5680
H	1.0785	-2.6314	0.3120
C	-2.1408	-2.4912	-1.2747
H	-2.4424	-3.1497	-2.1075
H	-1.0731	-2.2466	-1.3988
H	-2.2498	-3.0642	-0.3473
C	-2.7682	-0.6178	-2.7093
H	-1.7232	-0.2973	-2.8453
H	-2.9763	-1.3965	-3.4624
H	-3.4291	0.2303	-2.9295
C	-4.4737	-1.5937	-1.1791
H	-4.7586	-2.2436	-2.0248
H	-4.6777	-2.1576	-0.2590
H	-5.1373	-0.7191	-1.2003
C	-2.7805	-2.0351	1.8902
H	-2.9909	-2.2968	2.9410
H	-3.4490	-2.6408	1.2656
H	-1.7392	-2.3253	1.6793
C	-2.1224	0.1381	2.7910
H	-2.4221	-0.2499	3.7798
H	-1.0580	-0.1023	2.6348
H	-2.2205	1.2293	2.8225
C	-4.4643	-0.2036	1.9823
H	-5.1389	-0.6566	1.2439
H	-4.7446	-0.6032	2.9724
H	-4.6586	0.8772	2.0066
C	-2.1063	2.3430	-1.5190
H	-2.3996	3.3952	-1.6774
H	-1.0406	2.3212	-1.2389
H	-2.2141	1.8238	-2.4777
C	-4.4478	1.8381	-0.8042
H	-5.1219	1.4320	-0.0385
H	-4.7182	2.8982	-0.9517
H	-4.6520	1.3203	-1.7512
C	-2.7435	2.6567	0.8170
H	-2.9408	3.7002	0.5186
H	-3.4104	2.4295	1.6588
H	-1.7003	2.6041	1.1665

TS1

E(pbe1pbe) = -2013.15549599 a.u.

Lowest Freq. = -32.62 cm^{-1}

C	0.6130	-1.9124	3.4812
C	1.4096	-1.9456	2.3289
C	0.8693	-2.1402	1.0555
C	-0.5415	-2.3363	0.9763
C	-1.3542	-2.2910	2.1345
C	-0.7632	-2.0676	3.3869
H	1.0847	-1.7557	4.4543
H	2.4857	-1.7969	2.4372
H	-2.4169	-2.5347	2.0679
H	-1.3928	-2.0406	4.2801
C	1.7074	-2.1890	-0.1917
H	1.7068	-3.2197	-0.5868
H	1.2445	-1.5672	-0.9736
C	3.1674	-1.7723	-0.0699
H	3.6797	-2.4456	0.6473
O	3.7165	-1.9777	-1.3561
C	5.1153	-1.8407	-1.4500
H	5.4504	-2.5532	-2.2222
H	5.6053	-2.1449	-0.5058
C	5.5594	-0.4954	-1.8274
C	5.9346	0.6097	-2.1633
C	6.3677	1.9423	-2.5485
H	6.7330	2.5115	-1.6791
H	7.1807	1.8975	-3.2906
H	5.5362	2.5084	-2.9977
Pd	-1.3793	-0.5481	0.3691
I	-1.4276	-3.2260	-0.8257
P	-2.1649	1.5261	-0.2768
C	-4.0181	1.4335	-0.7547
C	-1.9496	2.8252	1.1157
C	-1.1605	2.0911	-1.8101
C	3.3564	-0.3984	0.4215
C	3.5904	0.7062	0.8696
C	3.9318	2.0220	1.4306
C	3.5295	3.1259	0.4448
C	3.1857	2.2042	2.7594
C	5.4473	2.0704	1.6726
H	4.0331	2.9939	-0.5251
H	2.4439	3.1266	0.2722
H	3.8144	4.1097	0.8500
H	3.4713	1.4254	3.4832
H	3.4309	3.1859	3.1945
H	2.0967	2.1539	2.6129
H	5.7270	3.0471	2.0983
H	5.7616	1.2846	2.3764
H	6.0024	1.9321	0.7322
C	-4.1462	0.6732	-2.0780
H	-3.7875	1.2470	-2.9420
H	-3.6135	-0.2904	-2.0461
H	-5.2135	0.4562	-2.2513
C	-4.7310	0.5657	0.2921
H	-4.7704	1.0228	1.2870
H	-5.7709	0.3988	-0.0371
H	-4.2406	-0.4169	0.3873
C	-4.7363	2.7769	-0.8794
H	-5.7724	2.5985	-1.2158
H	-4.7970	3.3112	0.0784
H	-4.2633	3.4422	-1.6138
C	-0.9587	0.8696	-2.7181
H	-0.4856	0.0421	-2.1635
H	-1.8857	0.4914	-3.1626
H	-0.2858	1.1539	-3.5452
C	0.2435	2.4946	-1.3514
H	0.8784	2.6259	-2.2436
H	0.2605	3.4428	-0.7996
H	0.7078	1.7130	-0.7298
C	-1.7701	3.2363	-2.6179
H	-1.9424	4.1357	-2.0120
H	-1.0721	3.5131	-3.4270
H	-2.7192	2.9567	-3.0951
C	-0.5890	2.5742	1.7807
H	0.2623	2.8082	1.1332
H	-0.5092	3.2137	2.6762
H	-0.4936	1.5232	2.0998
C	-2.9971	2.5599	2.2012
H	-2.7466	3.1746	3.0819
H	-4.0154	2.8321	1.8954
H	-2.9925	1.5061	2.5220
C	-2.0418	4.2870	0.6789
H	-1.9559	4.9327	1.5700
H	-1.2303	4.5730	-0.0039
H	-2.9979	4.5240	0.1937

IM1

E(pbe1pbe) = -2013.19858825 a.u.

Lowest Freq. = 16.33 cm^{-1}

C	0.1598	-1.9654	3.7157
C	0.7227	-2.2105	2.4630
C	0.1386	-1.7168	1.2880
C	-1.0345	-0.9490	1.4075
C	-1.6233	-0.7320	2.6552
C	-1.0231	-1.2353	3.8120
H	0.6426	-2.3573	4.6146
H	1.6390	-2.8037	2.4015
H	-2.5506	-0.1595	2.7339
H	-1.4840	-1.0498	4.7862
C	0.6980	-2.0170	-0.0735
H	0.1454	-2.8627	-0.5176
H	0.5010	-1.1598	-0.7345
C	2.1802	-2.3539	-0.1800
H	2.3859	-3.3064	0.3494
O	2.4051	-2.5461	-1.5649
C	3.7270	-2.8536	-1.9417
H	3.6613	-3.5548	-2.7907
H	4.2609	-3.3870	-1.1334
C	4.4962	-1.6770	-2.3578
C	5.1147	-0.6972	-2.7218
C	5.8468	0.4872	-3.1380
H	6.6233	0.7561	-2.4045
H	6.3390	0.3316	-4.1111
H	5.1671	1.3482	-3.2402
Pd	-1.9454	-0.3025	-0.1878
I	-3.2272	-2.6067	-0.6133
P	-1.1080	1.8713	-0.1880
C	-2.7690	2.7925	-0.4545
C	-0.1782	2.6576	1.2804
C	-0.0327	2.0186	-1.7719
C	3.0587	-1.3337	0.4095
C	3.8377	-0.5343	0.8886
C	4.8180	0.4118	1.4419
C	4.7059	1.7567	0.7108
C	4.5424	0.6021	2.9394
C	6.2245	-0.1704	1.2397
H	4.8600	1.6331	-0.3720
H	3.7189	2.2144	0.8690
H	5.4711	2.4518	1.0909
H	4.6307	-0.3517	3.4819
H	5.2698	1.3104	3.3666
H	3.5311	1.0011	3.1092
H	6.9785	0.5203	1.6495
H	6.3296	-1.1391	1.7518
H	6.4394	-0.3217	0.1706
C	-3.3555	2.3694	-1.8070
H	-2.8005	2.7719	-2.6636
H	-3.4214	1.2736	-1.9153
H	-4.3835	2.7626	-1.8692
C	-3.7653	2.3122	0.6119
H	-3.4742	2.5733	1.6347
H	-4.7382	2.7913	0.4116
H	-3.9114	1.2205	0.5636
C	-2.6781	4.3176	-0.4063
H	-3.6735	4.7296	-0.6444
H	-2.4049	4.6901	0.5899
H	-1.9696	4.7272	-1.1373
C	-0.5403	0.9974	-2.8006
H	-0.4802	-0.0317	-2.4120
H	-1.5683	1.1796	-3.1327
H	0.1092	1.0542	-3.6900
C	1.4157	1.6282	-1.4612
H	1.9602	1.5613	-2.4177
H	1.9403	2.3648	-0.8410
H	1.4930	0.6452	-0.9783
C	-0.0412	3.4122	-2.4037
H	0.3019	4.1960	-1.7168
H	0.6498	3.4023	-3.2635
H	-1.0297	3.6960	-2.7881
C	0.9123	1.7004	1.7759
H	1.6033	1.3753	0.9934
H	1.5006	2.2323	2.5413
H	0.4846	0.8072	2.2453
C	-1.1356	2.8746	2.4554
H	-0.5301	3.1673	3.3291
H	-1.8584	3.6811	2.2777
H	-1.6784	1.9589	2.7257
C	0.4628	4.0018	0.9220
H	0.8863	4.4310	1.8455
H	1.2891	3.9005	0.2072
H	-0.2561	4.7277	0.5221

IM2

E(pbe1pbe) = -2013.18568102 a.u.

Lowest Freq. = 21.48 cm^{-1}

C	5.2777	-1.1396	-0.1889
C	4.5479	-0.5035	-1.1917
C	3.1574	-0.3547	-1.0919
C	2.4947	-0.8613	0.0405
C	3.2290	-1.5005	1.0430
C	4.6148	-1.6358	0.9335
H	6.3613	-1.2473	-0.2843
H	5.0602	-0.1109	-2.0756
H	2.7208	-1.9078	1.9209
H	5.1742	-2.1357	1.7294
C	2.3837	0.3713	-2.1555
H	3.0189	0.6081	-3.0208
H	1.5442	-0.2496	-2.5088
C	1.7762	1.6830	-1.6376
H	2.5923	2.4163	-1.4937
O	0.8652	2.1631	-2.5948
C	0.9243	3.5524	-2.8272
H	0.2331	3.7476	-3.6620
H	1.9362	3.8384	-3.1733
C	0.5555	4.3885	-1.6825
C	0.2472	5.1019	-0.7490
C	-0.1496	5.9542	0.3579
H	0.6932	6.1557	1.0368
H	-0.5255	6.9212	-0.0124
H	-0.9552	5.4840	0.9437
Pd	0.5100	-0.5548	0.1862
P	-2.1485	-0.2259	0.0738
I	0.5425	-3.2262	-0.2622
C	-3.1310	-1.1183	1.4623
C	-2.7193	1.6133	0.0782
C	-2.7161	-0.9497	-1.6243
C	1.1948	1.4700	-0.2939
C	1.1631	1.4237	0.9512
C	1.4744	1.8217	2.3520
C	0.6678	1.0194	3.3715
C	2.9758	1.5746	2.5705
C	1.2028	3.3246	2.5065
H	-0.4050	1.2345	3.2859
H	0.8186	-0.0621	3.2319
H	0.9899	1.2866	4.3895
H	3.5802	2.1037	1.8184
H	3.2569	1.9485	3.5674
H	3.2204	0.5064	2.5199
H	1.5016	3.6420	3.5177
H	1.7876	3.9047	1.7773
H	0.1402	3.5667	2.3738
C	-1.5941	-0.6312	-2.6228
H	-1.3821	0.4398	-2.7222
H	-0.6578	-1.1308	-2.3308
H	-1.8826	-1.0116	-3.6177
C	-2.8482	-2.4754	-1.5808
H	-1.9462	-2.9680	-1.2001
H	-3.7123	-2.8158	-0.9967
H	-3.0002	-2.8265	-2.6156
C	-4.0502	-0.4201	-2.1582
H	-4.2641	-0.9409	-3.1071
H	-4.8852	-0.6324	-1.4773
H	-4.0466	0.6531	-2.3794
C	-2.2245	2.2861	-1.2060
H	-2.3861	3.3720	-1.1070
H	-1.1505	2.1284	-1.3715
H	-2.7611	1.9630	-2.1056
C	-4.2240	1.8514	0.2268
H	-4.4080	2.9335	0.1111
H	-4.8321	1.3347	-0.5236
H	-4.5918	1.5721	1.2231
C	-2.0208	2.3499	1.2234
H	-2.3038	1.9922	2.2183
H	-0.9345	2.2856	1.1214
H	-2.2957	3.4166	1.1670
C	-4.6161	-1.3433	1.1678
H	-5.0756	-1.8159	2.0530
H	-5.1605	-0.4114	0.9761
H	-4.7848	-2.0193	0.3207
C	-2.4645	-2.4701	1.7301
H	-2.4693	-3.1398	0.8629
H	-1.4208	-2.3436	2.0537
H	-3.0117	-2.9735	2.5452
C	-3.0140	-0.3284	2.7686
H	-3.6076	0.5945	2.7688
H	-3.3985	-0.9606	3.5864
H	-1.9705	-0.0811	3.0094

TS2

E(pbe1pbe) = -2013.15080673 a.u.

Lowest Freq. = -339.99 cm^{-1}

C	-3.4423	-3.9682	-0.0726
C	-2.7371	-3.6409	-1.2323
C	-1.6759	-2.7420	-1.1762
C	-1.3054	-2.1616	0.0578
C	-1.9755	-2.5507	1.2328
C	-3.0474	-3.4343	1.1577
H	-4.2825	-4.6652	-0.1231
H	-3.0027	-4.1001	-2.1887
H	-1.6880	-2.1281	2.1978
H	-3.5840	-3.7068	2.0704
C	-0.7126	-2.4492	-2.2754
H	-0.7971	-3.1377	-3.1267
H	-0.8154	-1.4154	-2.6470
C	0.6521	-2.5649	-1.5866
H	0.9036	-3.6337	-1.4478
O	1.6296	-1.9286	-2.3470
C	2.8609	-2.5933	-2.5180
H	3.2151	-2.3344	-3.5295
H	2.7095	-3.6881	-2.5037
C	3.9001	-2.2275	-1.5547
C	4.8230	-1.9281	-0.8239
C	5.9256	-1.5680	0.0499
H	6.0001	-2.2532	0.9086
H	6.8819	-1.6002	-0.4957
H	5.7978	-0.5463	0.4408
Pd	-0.5564	-0.2184	0.2131
P	0.4857	2.1075	-0.0146
I	-3.3147	0.5010	0.0071
C	0.1546	3.3342	1.4198
C	2.3834	2.0065	-0.2812
C	-0.2409	2.8667	-1.6345
C	0.4725	-1.9834	-0.1908
C	1.1279	-1.5993	0.8548
C	1.6388	-1.9581	2.2157
C	1.1157	-0.9769	3.2675
C	1.1308	-3.3776	2.5237
C	3.1735	-1.9969	2.2624
H	1.5216	0.0286	3.1003
H	0.0171	-0.9144	3.2496
H	1.4289	-1.3112	4.2689
H	1.5092	-4.0964	1.7811
H	1.4904	-3.6872	3.5179
H	0.0346	-3.4304	2.5269
H	3.4851	-2.3459	3.2595
H	3.5700	-2.6981	1.5150
H	3.6239	-1.0117	2.0928
C	-0.4410	1.7074	-2.6213
H	0.4894	1.1876	-2.8775
H	-1.1483	0.9649	-2.2212
H	-0.8679	2.1080	-3.5566
C	-1.6188	3.4952	-1.4071
H	-2.3262	2.8030	-0.9357
H	-1.5785	4.4195	-0.8178
H	-2.0310	3.7607	-2.3953
C	0.6286	3.9460	-2.2857
H	0.0913	4.3218	-3.1732
H	0.7992	4.8042	-1.6223
H	1.6002	3.5778	-2.6342
C	2.6674	1.2898	-1.6063
H	3.7470	1.0685	-1.6463
H	2.1371	0.3290	-1.6804
H	2.4317	1.8911	-2.4925
C	3.1431	3.3334	-0.2371
H	4.2019	3.1269	-0.4704
H	2.7865	4.0735	-0.9622
H	3.1207	3.7887	0.7621
C	2.9560	1.0933	0.8002
H	2.8478	1.4854	1.8165
H	2.4791	0.1060	0.7494
H	4.0330	0.9559	0.6092
C	0.3841	4.8098	1.0844
H	0.2062	5.4028	1.9980
H	1.4102	5.0161	0.7565
H	-0.3035	5.1890	0.3187
C	-1.2937	3.1324	1.8763
H	-2.0323	3.3494	1.0964
H	-1.4610	2.1011	2.2224
H	-1.4923	3.8100	2.7239
C	1.0316	2.9786	2.6227
H	2.0895	3.2296	2.4726
H	0.6780	3.5629	3.4886
H	0.9518	1.9161	2.8909

IM3

E(pbe1pbe) = -2013.22698985 a.u.

Lowest Freq. = 9.26 cm^{-1}

C	0.6877	-2.0091	4.2472
C	1.6262	-2.5331	3.3553
C	1.9873	-1.7929	2.2331
C	1.4082	-0.5353	1.9888
C	0.4961	0.0056	2.9065
C	0.1360	-0.7424	4.0254
H	0.3960	-2.5828	5.1307
H	2.0749	-3.5137	3.5362
H	0.0719	0.9988	2.7430
H	-0.5805	-0.3291	4.7400
C	2.9557	-2.1567	1.1511
H	3.9051	-2.5474	1.5459
H	2.5264	-2.9370	0.4995
C	3.1351	-0.8591	0.3351
H	4.0930	-0.3771	0.6099
O	3.0979	-1.0676	-1.0563
C	4.1742	-1.8174	-1.5681
H	3.9529	-1.9736	-2.6355
H	4.2178	-2.8210	-1.1044
C	5.4789	-1.1644	-1.4271
C	6.5561	-0.6161	-1.3071
C	7.8401	0.0485	-1.1665
H	8.4575	-0.4310	-0.3907
H	8.4037	0.0251	-2.1126
H	7.7022	1.1045	-0.8847
Pd	-0.3199	0.5655	-0.0463
P	-2.1226	-0.9307	-0.7236
I	-1.5462	2.7736	0.7643
C	-2.6979	-0.4812	-2.4883
C	-1.3357	-2.6801	-0.7524
C	-3.6408	-0.9725	0.4459
C	1.9757	0.0507	0.7539
C	1.5264	1.1509	0.0834
C	2.1948	2.2691	-0.6666
C	1.4329	2.6234	-1.9459
C	2.1970	3.4786	0.2892
C	3.6542	1.9520	-1.0158
H	1.4089	1.7681	-2.6397
H	0.3956	2.9154	-1.7256
H	1.9256	3.4649	-2.4587
H	2.7478	3.2494	1.2148
H	2.6948	4.3281	-0.2059
H	1.1762	3.7823	0.5597
H	4.1017	2.8318	-1.5048
H	4.2462	1.7328	-0.1151
H	3.7290	1.0962	-1.6967
C	-3.1242	-0.8280	1.8844
H	-2.4928	-1.6621	2.2091
H	-2.5532	0.1034	2.0099
H	-3.9918	-0.7853	2.5645
C	-4.5360	0.2446	0.1897
H	-3.9770	1.1894	0.2436
H	-5.0653	0.1970	-0.7704
H	-5.3033	0.2713	0.9816
C	-4.5044	-2.2301	0.3364
H	-5.3746	-2.1134	1.0049
H	-4.8908	-2.3895	-0.6793
H	-3.9767	-3.1389	0.6534
C	-1.1417	-3.1414	0.6966
H	-0.5132	-4.0475	0.6905
H	-0.6196	-2.3844	1.3008
H	-2.0822	-3.4039	1.1969
C	-2.1136	-3.7438	-1.5269
H	-1.5919	-4.7100	-1.4147
H	-3.1393	-3.8806	-1.1634
H	-2.1546	-3.5245	-2.6026
C	0.0726	-2.5731	-1.3509
H	0.0753	-2.3770	-2.4284
H	0.6750	-1.7922	-0.8669
H	0.5905	-3.5343	-1.1936
C	-3.9629	-1.1968	-2.9623
H	-4.1628	-0.9050	-4.0077
H	-3.8627	-2.2897	-2.9402
H	-4.8493	-0.9217	-2.3760
C	-2.9089	1.0373	-2.5447
H	-3.6957	1.3991	-1.8738
H	-1.9803	1.5742	-2.2990
H	-3.1966	1.3112	-3.5739
C	-1.5613	-0.7810	-3.4702
H	-1.4234	-1.8548	-3.6504
H	-1.8113	-0.3172	-4.4390
H	-0.6048	-0.3504	-3.1343

TS3

E(pbe1pbe) = -2013.18283154 a.u.

Lowest Freq. = -336.04 cm^{-1}

C	0.5843	4.6959	-0.2729
C	1.6580	4.0759	-0.9225
C	2.0618	2.8128	-0.5062
C	1.3844	2.1539	0.5361
C	0.3337	2.7937	1.2091
C	-0.0648	4.0637	0.7923
H	0.2639	5.6928	-0.5872
H	2.1727	4.5826	-1.7435
H	-0.1405	2.3079	2.0643
H	-0.8808	4.5745	1.3102
C	3.1687	1.9497	-1.0315
H	2.9191	1.5646	-2.0339
H	4.1164	2.5004	-1.1314
C	3.2723	0.7926	-0.0210
H	3.3937	-0.1685	-0.5441
O	4.3263	0.9652	0.9127
C	5.6274	0.8624	0.3844
H	6.3106	1.0640	1.2245
H	5.8185	1.6486	-0.3707
C	5.9406	-0.4437	-0.2004
C	6.1759	-1.5335	-0.6826
C	6.4335	-2.8479	-1.2441
H	6.5715	-2.7994	-2.3357
H	7.3377	-3.2987	-0.8054
H	5.5879	-3.5242	-1.0397
Pd	-0.4590	-0.1425	0.3964
P	-2.7405	-0.2718	0.0535
I	0.3768	-0.8400	-1.9732
C	-3.3368	-2.0849	0.0062
C	-3.4022	0.5565	1.6586
C	-3.3994	0.6908	-1.4624
C	1.9534	0.8202	0.7706
C	1.3268	-0.1546	1.5288
C	1.9928	-1.4129	2.0600
C	0.9106	-2.3966	2.5203
C	2.9147	-2.1557	1.0832
C	2.8143	-0.9733	3.2834
H	0.2218	-1.9227	3.2374
H	0.3116	-2.7443	1.6626
H	1.3558	-3.2828	3.0036
H	3.8280	-1.5892	0.8655
H	3.2268	-3.1195	1.5197
H	2.4002	-2.3714	0.1330
H	3.2661	-1.8500	3.7783
H	3.6183	-0.2872	2.9780
H	2.1815	-0.4571	4.0239
C	-2.5182	1.9328	-1.6517
H	-2.5708	2.6358	-0.8129
H	-1.4641	1.6586	-1.8047
H	-2.8635	2.4654	-2.5532
C	-3.2738	-0.1495	-2.7369
H	-2.2492	-0.5063	-2.9035
H	-3.9589	-1.0063	-2.7542
H	-3.5418	0.4982	-3.5879
C	-4.8662	1.1108	-1.3266
H	-5.1636	1.6019	-2.2683
H	-5.5371	0.2548	-1.1776
H	-5.0385	1.8331	-0.5200
C	-3.2289	2.0746	1.5586
H	-3.4243	2.5042	2.5546
H	-2.2065	2.3526	1.2753
H	-3.9290	2.5476	0.8597
C	-4.8664	0.2467	1.9736
H	-5.1505	0.8352	2.8625
H	-5.5502	0.5272	1.1628
H	-5.0329	-0.8095	2.2215
C	-2.5321	0.1038	2.8406
H	-2.6109	-0.9653	3.0622
H	-1.4672	0.3412	2.6822
H	-2.8619	0.6555	3.7367
C	-4.8199	-2.2227	-0.3497
H	-5.0920	-3.2877	-0.2591
H	-5.4781	-1.6571	0.3205
H	-5.0356	-1.9219	-1.3830
C	-2.5034	-2.8772	-1.0051
H	-2.5546	-2.4827	-2.0258
H	-1.4470	-2.9188	-0.7065
H	-2.8911	-3.9092	-1.0254
C	-3.0838	-2.7392	1.3682
H	-3.7609	-2.3809	2.1534
H	-3.2587	-3.8220	1.2583
H	-2.0443	-2.6046	1.7022

IM4

E(pbe1pbe) = -2013.21705572 a.u.

Lowest Freq. = 23.09 cm^{-1}

C	-6.80222	0.549885	0.197789
C	-6.00522	1.075747	-0.81996
C	-4.66259	0.719879	-0.8874
C	-4.07284	-0.14531	0.061995
C	-4.89803	-0.69174	1.055194
C	-6.24756	-0.34005	1.117935
H	-7.86017	0.817318	0.26153
H	-6.4309	1.750275	-1.56801
H	-4.51963	-1.40304	1.785152
H	-6.87482	-0.77833	1.898571
C	-3.66984	1.100094	-1.93638
H	-4.09786	1.164391	-2.94682
H	-3.18143	2.062893	-1.70817
C	-2.65599	-0.02899	-1.80101
H	-3.10321	-0.93115	-2.26906
O	-1.47939	0.28396	-2.47706
C	-0.78588	-0.83304	-2.9581
H	0.095656	-0.46062	-3.49986
H	-1.40511	-1.41195	-3.67156
C	-0.39883	-1.71372	-1.84886
C	-0.34568	-2.66055	-1.03804
C	-0.46713	-4.00838	-0.49215
H	-0.23485	-4.73899	-1.28307
H	0.218485	-4.17964	0.34804
H	-1.49865	-4.18795	-0.15306
Pd	0.339617	-0.7738	-0.12278
P	1.445469	1.629239	0.142463
C	2.007545	2.093656	-1.63803
C	0.29609	3.034039	0.777638
C	3.032155	1.665062	1.250699
C	-2.62587	-0.32683	-0.28526
C	-1.55987	-0.57589	0.506605
C	-1.59198	-0.95476	1.998908
C	-0.19273	-1.02711	2.624625
C	-2.35981	0.091871	2.822241
C	-2.1879	-2.36712	2.153098
H	0.464915	-1.74719	2.107542
H	0.298309	-0.04965	2.642897
H	-0.28219	-1.37325	3.66706
H	-1.89416	1.080421	2.71951
H	-3.41284	0.194487	2.544986
H	-2.3206	-0.18911	3.887043
H	-2.47541	-2.54015	3.202929
H	-3.06416	-2.54773	1.519468
H	-1.43708	-3.12395	1.888426
C	2.754878	0.851294	2.518719
H	1.938204	1.256188	3.127617
H	2.5289	-0.19699	2.277394
H	3.664571	0.859663	3.142358
C	4.211853	0.979405	0.555434
H	3.963384	-0.02931	0.204364
H	4.619551	1.558901	-0.28079
H	5.02031	0.875094	1.2985
C	3.503	3.067248	1.650076
H	4.442635	2.954316	2.217225
H	3.721898	3.705663	0.785075
H	2.800376	3.596438	2.304285
C	0.14683	2.917652	2.297624
H	-0.68317	3.571301	2.613113
H	-0.10485	1.897482	2.611918
H	1.038355	3.237395	2.849725
C	0.743687	4.458813	0.440543
H	0.042762	5.156671	0.929806
H	1.749686	4.699851	0.801577
H	0.701052	4.668112	-0.63598
C	-1.09856	2.819581	0.184248
H	-1.11818	2.900391	-0.90768
H	-1.50585	1.839456	0.45521
H	-1.77212	3.593617	0.589137
C	3.027046	3.232328	-1.73177
H	3.210809	3.431716	-2.80149
H	2.664097	4.166032	-1.28576
H	3.997153	2.989311	-1.28299
C	2.604069	0.838537	-2.28621
H	3.53771	0.510293	-1.81608
H	1.902395	-0.00748	-2.25961
H	2.824055	1.061375	-3.34383
C	0.788873	2.495235	-2.47338
H	0.413986	3.494353	-2.21465
H	1.100986	2.535251	-3.53074
H	-0.03818	1.779342	-2.39368
I	2.90492	-2.50053	-0.06795

TS4

E(pbe1pbe) = -2013.18757131 a.u.

Lowest Freq. = - -273.32 cm^{-1}

C	6.443988	0.805809	-0.57031
C	5.734974	1.116249	0.589929
C	4.499256	0.521219	0.815933
C	3.924421	-0.3804	-0.11594
C	4.671472	-0.70588	-1.25851
C	5.915421	-0.11338	-1.47651
H	7.41794	1.264899	-0.75886
H	6.148933	1.812064	1.32489
H	4.318088	-1.42796	-1.99029
H	6.48162	-0.38305	-2.37183
C	3.639352	0.678657	2.032647
H	4.217543	0.68783	2.967546
H	3.035572	1.599482	1.999608
C	2.773534	-0.55665	1.894166
H	3.421984	-1.41788	2.173286
O	1.655243	-0.59514	2.705875
C	1.078741	-1.86294	2.642075
H	0.281107	-1.90205	3.397632
H	1.821933	-2.64688	2.887489
C	0.460973	-2.14794	1.316878
C	-0.45525	-2.87349	0.806711
C	-1.14866	-4.1528	0.673698
H	-2.0356	-4.18552	1.326348
H	-1.49946	-4.34879	-0.3505
H	-0.4606	-4.9621	0.966536
Pd	-0.65285	-0.82203	0.141091
P	-1.03368	1.744228	-0.10689
C	-1.2003	2.327659	1.714352
C	0.341461	2.80134	-0.9506
C	-2.66981	2.232885	-1.02335
C	2.574344	-0.76479	0.393009
C	1.432792	-1.18046	-0.22167
C	1.443022	-1.81841	-1.63848
C	0.0937	-2.30502	-2.19316
C	1.900681	-0.77492	-2.67567
C	2.33581	-3.07008	-1.57971
H	-0.32051	-3.15831	-1.64444
H	-0.65976	-1.50194	-2.23588
H	0.257874	-2.64177	-3.23034
H	1.052969	-0.12752	-2.93446
H	2.718091	-0.123	-2.35458
H	2.218718	-1.28513	-3.5989
H	2.467581	-3.49477	-2.58826
H	3.328297	-2.88644	-1.15155
H	1.852413	-3.83758	-0.955
C	-2.75672	1.389684	-2.30271
H	-1.99952	1.663382	-3.04596
H	-2.66228	0.315049	-2.09281
H	-3.74437	1.5512	-2.76729
C	-3.90479	1.922323	-0.16977
H	-3.91928	0.896021	0.210218
H	-4.02477	2.614755	0.672492
H	-4.79432	2.04227	-0.81128
C	-2.78851	3.713895	-1.40221
H	-3.78369	3.860226	-1.85588
H	-2.73165	4.380044	-0.53182
H	-2.05367	4.045692	-2.14434
C	0.243043	2.643294	-2.47225
H	1.180833	3.01603	-2.91688
H	0.134669	1.592986	-2.7753
H	-0.57526	3.21729	-2.92177
C	0.335108	4.291716	-0.59832
H	1.096998	4.78826	-1.22374
H	-0.61942	4.792855	-0.78986
H	0.61869	4.472282	0.44737
C	1.706627	2.241318	-0.56184
H	1.897447	2.285901	0.513828
H	1.817873	1.20382	-0.88299
H	2.487477	2.838388	-1.06216
C	-1.8338	3.705752	1.917029
H	-1.81963	3.933278	2.996895
H	-1.28191	4.506532	1.41045
H	-2.88132	3.752767	1.596475
C	-2.02498	1.275026	2.467209
H	-3.05388	1.181294	2.101173
H	-1.55044	0.281573	2.413264
H	-2.07496	1.564632	3.530715
C	0.183985	2.336695	2.365955
H	0.826364	3.145862	1.995908
H	0.050315	2.502077	3.448807
H	0.699226	1.374468	2.249237
I	-3.37188	-1.66452	-0.10733

IM5

E(pbe1pbe) = -2013.28151074 a.u.

Lowest Freq. = 21.55 cm^{-1}

C	5.5085	2.8408	0.9832
C	5.5236	2.2961	-0.3022
C	4.6957	1.2176	-0.5969
C	3.8472	0.6562	0.3834
C	3.8346	1.2223	1.6671
C	4.6632	2.3052	1.9579
H	6.1500	3.6925	1.2242
H	6.1740	2.7165	-1.0743
H	3.1723	0.8442	2.4442
H	4.6411	2.7425	2.9594
C	4.5612	0.5053	-1.9088
H	4.4775	1.1951	-2.7612
H	5.4361	-0.1404	-2.0931
C	3.3344	-0.3866	-1.7289
H	2.4711	0.0155	-2.2955
O	3.6262	-1.6959	-2.1772
C	2.5814	-2.6189	-1.9602
H	3.0368	-3.5464	-1.5665
H	2.1019	-2.8671	-2.9224
C	1.5550	-2.0877	-0.9744
C	0.2308	-2.1189	-1.2336
C	-0.4639	-2.8640	-2.3239
H	-1.2683	-2.2671	-2.7818
H	0.2268	-3.1754	-3.1253
H	-0.9338	-3.7775	-1.9223
Pd	-0.8089	-0.6503	-0.4374
P	-1.8220	1.5511	-0.0961
I	-2.4938	-2.3782	0.6044
C	-2.6848	1.9645	1.5560
C	-0.3023	2.6915	-0.3224
C	-3.0408	1.8483	-1.5376
C	3.0397	-0.4062	-0.2337
C	2.1845	-1.3794	0.1852
C	1.9742	-1.9070	1.6137
C	3.3337	-2.0632	2.3097
C	1.0556	-0.9877	2.4239
C	1.3419	-3.3035	1.5820
H	3.9677	-2.7790	1.7629
H	3.8894	-1.1215	2.3923
H	3.1878	-2.4526	3.3303
H	0.0817	-0.8818	1.9212
H	0.8770	-1.4083	3.4272
H	1.4734	0.0193	2.5503
H	1.2823	-3.6990	2.6085
H	0.3262	-3.2885	1.1659
H	1.9454	-4.0056	0.9853
C	-2.2632	1.8274	-2.8576
H	-1.6265	0.9322	-2.9436
H	-2.9914	1.7941	-3.6850
H	-1.6433	2.7203	-3.0083
C	-4.0020	0.6539	-1.6059
H	-3.4539	-0.2891	-1.7513
H	-4.6342	0.5433	-0.7184
H	-4.6692	0.7946	-2.4729
C	-3.8409	3.1480	-1.4515
H	-3.1989	4.0366	-1.3928
H	-4.4567	3.2449	-2.3622
H	-4.5281	3.1631	-0.5951
C	-2.8058	3.4604	1.8496
H	-3.3549	3.9996	1.0663
H	-3.3640	3.5895	2.7927
H	-1.8310	3.9476	1.9835
C	-4.0859	1.3451	1.5805
H	-4.7888	1.8406	0.8989
H	-4.0650	0.2690	1.3531
H	-4.4881	1.4584	2.6012
C	-1.8998	1.2732	2.6785
H	-1.8758	0.1837	2.5293
H	-0.8688	1.6296	2.7791
H	-2.4130	1.4696	3.6350
C	0.5127	2.7044	0.9744
H	0.7483	1.6874	1.3191
H	1.4695	3.2138	0.7733
H	0.0206	3.2489	1.7898
C	0.5950	2.0165	-1.3695
H	1.5748	2.5224	-1.3851
H	0.7957	0.9592	-1.1068
H	0.1897	2.0445	-2.3858
C	-0.5900	4.1307	-0.7438
H	-1.2262	4.6607	-0.0225
H	0.3663	4.6784	-0.8054
H	-1.0620	4.1945	-1.7332

TS5

E(pbe1pbe) = -2013.25382828 a.u.

Lowest Freq. = -19.41 cm^{-1}

C	-6.7788	1.536436	0.739857
C	-6.22146	1.53184	-0.5401
C	-5.13111	0.712694	-0.80924
C	-4.56801	-0.1249	0.190041
C	-5.14381	-0.1058	1.470362
C	-6.23842	0.718739	1.73303
H	-7.6377	2.174808	0.962425
H	-6.63482	2.167766	-1.32781
H	-4.76813	-0.73253	2.276309
H	-6.67789	0.715124	2.733889
C	-4.38926	0.585798	-2.10617
H	-5.04063	0.605624	-2.99148
H	-3.64362	1.395147	-2.20822
C	-3.68325	-0.75227	-1.89903
H	-4.4287	-1.54936	-2.08081
O	-2.61235	-1.04576	-2.74132
C	-1.41018	-0.59743	-2.19623
H	-1.46733	0.482023	-1.97828
H	-0.61526	-0.75631	-2.93725
C	-1.11071	-1.32259	-0.88279
C	-0.01097	-2.16809	-0.96142
C	0.269979	-3.41592	-1.65336
H	1.214303	-3.88293	-1.34307
H	-0.58175	-4.1119	-1.5928
H	0.370599	-3.13748	-2.7228
Pd	0.965958	-0.73144	-0.2329
P	1.620919	1.676891	0.084744
I	3.315876	-2.25248	0.155667
C	3.072876	2.060697	-1.1105
C	0.163896	2.848974	-0.35205
C	2.180944	2.074414	1.875293
C	-3.39877	-0.81006	-0.40242
C	-2.22467	-1.32188	0.085958
C	-2.04726	-1.82744	1.528441
C	-1.99838	-0.63719	2.494376
C	-3.19056	-2.80775	1.841357
C	-0.7619	-2.62771	1.749347
H	-1.08271	-0.05859	2.308401
H	-2.84769	0.048543	2.387251
H	-1.97068	-0.98793	3.538568
H	-3.05591	-3.73266	1.258106
H	-3.18061	-3.0794	2.909012
H	-4.18574	-2.4206	1.596881
H	-0.79425	-3.08135	2.752873
H	-0.65578	-3.45742	1.031361
H	0.142889	-2.00198	1.70845
C	0.330009	4.303552	0.091488
H	1.23342	4.773976	-0.31678
H	-0.53621	4.879682	-0.27677
H	0.345997	4.414661	1.18339
C	-0.05252	2.831271	-1.86876
H	0.710299	3.398321	-2.41676
H	-0.07926	1.811888	-2.27551
H	-1.02641	3.300267	-2.0866
C	-1.11053	2.277298	0.282729
H	-1.97714	2.851748	-0.08576
H	-1.2616	1.223772	0.010662
H	-1.11529	2.345794	1.375999
C	2.793889	1.307735	-2.41939
H	1.917089	1.682152	-2.95998
H	3.666289	1.420166	-3.08513
H	2.647841	0.231208	-2.23239
C	3.304988	3.542807	-1.40766
H	4.188418	3.631405	-2.06299
H	2.466191	4.012547	-1.93624
H	3.511393	4.125876	-0.50063
C	4.376958	1.47919	-0.55599
H	4.746407	2.012314	0.328812
H	4.285029	0.410773	-0.31519
H	5.149027	1.575112	-1.33785
C	2.982654	3.367926	2.030677
H	3.196478	3.518503	3.102823
H	3.949493	3.334165	1.512478
H	2.435035	4.251411	1.679247
C	3.012522	0.891778	2.384379
H	2.422301	-0.03687	2.384382
H	3.924139	0.710588	1.804518
H	3.314537	1.099507	3.424907
C	0.958306	2.145682	2.793824
H	1.315265	2.176223	3.836694
H	0.345661	3.04193	2.635479
H	0.317962	1.257888	2.694233

IM6

E(pbe1pbe) = -1943.83187482 a.u.

Lowest Freq. = 12.34 cm^{-1}

C	-6.6007	-1.6561	2.3714
C	-6.0474	-2.4553	1.3705
C	-5.0585	-1.9321	0.5434
C	-4.5965	-0.6040	0.6918
C	-5.1702	0.1872	1.7012
C	-6.1602	-0.3407	2.5291
H	-7.3754	-2.0581	3.0295
H	-6.3856	-3.4862	1.2339
H	-4.8589	1.2168	1.8631
H	-6.5915	0.2884	3.3121
C	-4.3750	-2.6367	-0.5882
H	-3.9494	-3.6040	-0.2816
H	-5.0798	-2.8373	-1.4114
C	-3.3174	-1.6497	-1.0700
H	-2.2973	-2.0362	-0.8753
O	-3.4761	-1.4269	-2.4609
C	-2.6524	-0.3966	-2.9634
H	-3.2955	0.3267	-3.4992
H	-1.9453	-0.8162	-3.6992
C	-1.9001	0.3193	-1.8540
C	-0.5619	0.3706	-1.9118
C	0.3701	-0.2745	-2.8449
H	0.1277	-1.3162	-3.1055
H	0.6343	0.3125	-3.7369
H	1.4192	-0.4458	-2.3376
Pd	1.0874	0.4433	-0.8247
P	3.0343	-0.5789	0.2977
C	2.1761	-1.4373	1.7876
C	3.7718	-1.9297	-0.8493
C	4.4745	0.4938	0.9585
C	-3.5375	-0.3506	-0.3041
C	-2.7983	0.7130	-0.7302
C	-2.9283	2.1747	-0.2886
C	-4.3746	2.6407	-0.5162
C	-2.5033	2.3280	1.1775
C	-2.0320	3.0840	-1.1304
H	-4.6267	2.5992	-1.5879
H	-5.1161	2.0350	0.0198
H	-4.4908	3.6852	-0.1834
H	-1.4342	2.0966	1.2840
H	-2.6669	3.3647	1.5151
H	-3.0569	1.6637	1.8525
H	-2.1172	4.1223	-0.7724
H	-0.9841	2.7747	-1.0564
H	-2.3172	3.0665	-2.1939
O	0.6430	1.7144	0.7142
C	0.9812	2.9666	0.7289
O	0.7842	3.6798	1.7037
C	1.5956	3.5562	-0.5300
H	2.1322	2.8096	-1.1348
H	0.7870	3.9717	-1.1527
H	2.2684	4.3842	-0.2674
C	2.6936	-2.9850	-1.1197
H	1.7477	-2.5390	-1.4608
H	3.0543	-3.6488	-1.9228
H	2.4865	-3.6170	-0.2469
C	4.1241	-1.3073	-2.2080
H	4.4070	-2.1211	-2.8963
H	3.2755	-0.7765	-2.6624
H	4.9686	-0.6120	-2.1621
C	5.0193	-2.6317	-0.3080
H	5.2992	-3.4351	-1.0107
H	5.8817	-1.9559	-0.2345
H	4.8580	-3.0952	0.6729
C	2.9406	-2.6149	2.3924
H	2.3886	-2.9674	3.2806
H	3.0189	-3.4670	1.7041
H	3.9515	-2.3431	2.7215
C	0.7886	-1.9208	1.3362
H	0.8184	-2.6688	0.5360
H	0.2867	-2.3842	2.2026
H	0.1569	-1.0826	1.0002
C	1.9160	-0.3946	2.8814
H	2.8307	-0.0747	3.3968
H	1.3947	0.4872	2.4799
H	1.2638	-0.8584	3.6407
C	3.8999	1.8087	1.4961
H	3.4605	2.4000	0.6849
H	3.1449	1.6844	2.2777
H	4.7298	2.3985	1.9207
C	5.3184	-0.1725	2.0467
H	6.1636	0.4949	2.2877

H	4.7573	-0.3289	2.9770
H	5.7396	-1.1353	1.7320
C	5.3903	0.8879	-0.2045
H	6.0761	1.6741	0.1529
H	6.0114	0.0581	-0.5647
H	4.8261	1.3059	-1.0524

TS6

E(pbe1pbe) = -1943.81572794 a.u.

Lowest Freq. = -371.65 cm^{-1}

C	-6.4383	-2.3846	1.9230
C	-6.2696	-2.5325	0.5455
C	-5.3338	-1.7445	-0.1168
C	-4.5573	-0.7883	0.5751
C	-4.7299	-0.6611	1.9622
C	-5.6658	-1.4554	2.6234
H	-7.1671	-3.0019	2.4548
H	-6.8621	-3.2646	-0.0102
H	-4.1353	0.0412	2.5438
H	-5.7881	-1.3501	3.7045
C	-5.0110	-1.7591	-1.5796
H	-4.8700	-2.7757	-1.9746
H	-5.8212	-1.2900	-2.1625
C	-3.7645	-0.8865	-1.6997
H	-2.8666	-1.5052	-1.9029
O	-3.9400	0.0541	-2.7412
C	-2.8397	0.9113	-2.8906
H	-3.2329	1.9119	-3.1439
H	-2.1965	0.5805	-3.7287
C	-1.9960	0.9813	-1.6234
C	-0.6733	0.9134	-1.7874
C	0.2570	0.5124	-2.7420
H	0.1038	-0.4300	-3.2842
H	0.8866	1.2400	-3.2720
H	1.3121	-0.6027	-1.8517
Pd	1.1030	0.4575	-0.7759
P	2.9352	-0.8088	0.1453
C	2.2914	-1.3245	1.8776
C	3.2479	-2.3951	-0.8921
C	4.5853	0.1341	0.3330
C	-3.6275	-0.1489	-0.3714
C	-2.7835	0.9202	-0.3587
C	-2.6975	2.0291	0.6967
C	-4.1126	2.5086	1.0515
C	-1.9406	1.5459	1.9418
C	-1.9550	3.2407	0.1286
H	-4.6290	2.8846	0.1539
H	-4.7426	1.7280	1.4946
H	-4.0513	3.3361	1.7767
H	-0.8721	1.4289	1.7095
H	-2.0273	2.2925	2.7491
H	-2.3222	0.5896	2.3243
H	-1.8866	4.0287	0.8949
H	-0.9399	2.9655	-0.1706
H	-2.4763	3.6601	-0.7469
O	1.0163	1.8362	0.8144
C	1.3972	3.0676	0.7838
O	1.4932	3.7649	1.7885
C	1.6901	3.6745	-0.5816
H	2.0395	2.9288	-1.3112
H	0.7595	4.1138	-0.9778
H	2.4271	4.4844	-0.4895
C	2.0128	-3.2971	-0.7994
H	1.0889	-2.7570	-1.0603
H	2.1317	-4.1165	-1.5273
H	1.8852	-3.7570	0.1883
C	3.4071	-2.0489	-2.3788
H	3.5554	-2.9927	-2.9290
H	2.5083	-1.5728	-2.7986
H	4.2648	-1.4045	-2.5958
C	4.4842	-3.1886	-0.4588
H	4.5115	-4.1295	-1.0337
H	5.4180	-2.6530	-0.6762
H	4.4764	-3.4538	0.6048
C	2.9912	-2.5364	2.4934
H	2.5814	-2.6894	3.5063
H	2.8172	-3.4644	1.9333
H	4.0745	-2.3910	2.5978
C	0.7874	-1.6125	1.7515
H	0.5527	-2.4496	1.0845
H	0.3992	-1.8677	2.7521
H	0.2345	-0.7268	1.4009
C	2.4182	-0.1426	2.8459
H	3.4580	0.0751	3.1218
H	1.9446	0.7627	2.4394
H	1.8899	-0.4171	3.7748
C	4.2780	1.5749	0.7526
H	3.7183	2.0975	-0.0332
H	3.7144	1.6584	1.6865
H	5.2366	2.1035	0.8872
C	5.5556	-0.4947	1.3351
H	6.4946	0.0843	1.3161

H	5.1773	-0.4613	2.3648
H	5.8066	-1.5347	1.0930
C	5.2745	0.2320	-1.0310
H	6.1076	0.9485	-0.9398
H	5.7026	-0.7211	-1.3665
H	4.5966	0.6141	-1.8100

IM7

E(pbe1pbe) = -1943.83555635 a.u.

Lowest Freq. = 22.36 cm^{-1}

C	-4.9546	-3.2718	1.1914
C	-5.1444	-2.7626	-0.0952
C	-4.4920	-1.5916	-0.4670
C	-3.6539	-0.9083	0.4401
C	-3.4578	-1.4376	1.7227
C	-4.1114	-2.6133	2.0903
H	-5.4567	-4.1952	1.4918
H	-5.7909	-3.2834	-0.8068
H	-2.7836	-0.9578	2.4314
H	-3.9514	-3.0271	3.0892
C	-4.5432	-0.8904	-1.7923
H	-4.4793	-1.5798	-2.6463
H	-5.4839	-0.3232	-1.8944
C	-3.3872	0.1082	-1.7283
H	-2.5161	-0.2696	-2.3013
O	-3.8040	1.3561	-2.2542
C	-2.8906	2.4213	-2.0780
H	-3.4499	3.2807	-1.6677
H	-2.4570	2.7220	-3.0482
C	-1.7892	1.9943	-1.1310
C	-0.5349	2.0238	-1.5370
C	0.5714	2.2715	-2.2682
H	0.8192	1.6699	-3.1509
H	1.1301	3.1941	-2.0683
H	0.4618	-0.2406	-1.5665
Pd	1.1093	0.8110	-0.6921
P	2.1016	-1.2284	-0.0466
C	0.8347	-2.6369	0.2604
C	3.1808	-1.6787	-1.5679
C	3.2189	-1.0502	1.4957
C	-3.0319	0.2282	-0.2497
C	-2.2778	1.3022	0.0943
C	-2.0296	1.9394	1.4666
C	-3.3275	1.9323	2.2836
C	-0.9119	1.2217	2.2258
C	-1.6167	3.4077	1.2939
H	-4.1185	2.4950	1.7631
H	-3.7074	0.9213	2.4761
H	-3.1559	2.4133	3.2599
H	0.0243	1.2500	1.6493
H	-0.7289	1.7190	3.1927
H	-1.1601	0.1724	2.4310
H	-1.5126	3.8747	2.2862
H	-0.6608	3.5180	0.7646
H	-2.3780	3.9763	0.7360
O	2.0949	2.1696	0.6362
C	2.0162	3.4476	0.6925
O	1.6463	4.2007	-0.2104
C	2.4073	4.0229	2.0432
H	3.3355	3.5621	2.4122
H	2.5217	5.1140	1.9937
H	1.6144	3.7818	2.7705
C	2.2935	-2.1903	-2.7073
H	1.4863	-1.4835	-2.9508
H	2.9249	-2.2922	-3.6053
H	1.8524	-3.1748	-2.5080
C	3.8387	-0.3918	-2.0860
H	4.4115	-0.6375	-2.9959
H	3.0814	0.3617	-2.3569
H	4.5319	0.0648	-1.3711
C	4.2661	-2.7201	-1.2877
H	4.7825	-2.9443	-2.2363
H	5.0277	-2.3568	-0.5857
H	3.8620	-3.6652	-0.9032
C	1.4455	-4.0356	0.1367
H	0.6774	-4.7720	0.4267
H	1.7511	-4.2772	-0.8893
H	2.3077	-4.1839	0.7987
C	-0.3406	-2.5105	-0.7161
H	-0.0477	-2.5597	-1.7711
H	-1.0354	-3.3451	-0.5233
H	-0.8920	-1.5739	-0.5538
C	0.2295	-2.4927	1.6591
H	0.9356	-2.7325	2.4636
H	-0.1750	-1.4862	1.8271
H	-0.6097	-3.2028	1.7397
C	2.4384	-0.2915	2.5783
H	2.1517	0.7046	2.2138
H	1.5499	-0.8222	2.9358
H	3.1098	-0.1588	3.4440
C	3.7351	-2.3707	2.0700
H	4.4298	-2.1353	2.8941

H	2.9331	-2.9887	2.4951
H	4.2874	-2.9744	1.3388
C	4.4102	-0.1559	1.1352
H	4.9442	0.0852	2.0697
H	5.1329	-0.6442	0.4692
H	4.0751	0.7929	0.6918

IM2_OAc

E(pbe1pbe) = -1943.76640484 a.u.

Lowest Freq. = 24.87 cm^{-1}

C	5.115286	-1.85753	-0.9097
C	4.450947	-0.89505	-1.66753
C	3.119394	-0.54681	-1.39755
C	2.443098	-1.18664	-0.3393
C	3.114378	-2.15387	0.414707
C	4.442392	-2.48759	0.135507
H	6.154043	-2.11255	-1.136
H	4.96962	-0.39815	-2.49334
H	2.590265	-2.65709	1.229492
H	4.949342	-3.24406	0.741852
C	2.44154	0.501366	-2.23553
H	3.107783	0.854066	-3.0355
H	1.540529	0.085727	-2.71262
C	1.988102	1.720457	-1.42838
H	2.879812	2.301052	-1.12475
O	1.170607	2.506856	-2.26054
C	1.34306	3.898758	-2.13369
H	0.753811	4.349801	-2.9476
H	2.40141	4.16924	-2.3142
C	0.917477	4.47066	-0.85386
C	0.573799	4.990075	0.189082
C	0.146966	5.619565	1.426338
H	0.947297	5.608362	2.182224
H	-0.13726	6.669052	1.249062
H	-0.7288	5.102179	1.848364
Pd	0.520996	-0.72056	0.006968
P	-2.08966	-0.23693	-0.16001
C	-3.26712	-1.19464	1.018258
C	-2.59223	1.615226	-0.06834
C	-2.45508	-0.83394	-1.9552
C	1.343646	1.283114	-0.16948
C	1.244353	1.008586	1.042826
C	1.475523	1.078224	2.509654
C	0.453633	0.243307	3.280691
C	2.887448	0.532318	2.770846
C	1.409818	2.548616	2.938822
H	-0.55529	0.6631	3.175543
H	0.441649	-0.79513	2.914001
H	0.712631	0.239453	4.350648
H	3.643599	1.086062	2.193943
H	3.121544	0.637299	3.841801
H	2.959382	-0.53051	2.503508
H	1.642164	2.625211	4.012575
H	2.140219	3.156406	2.383841
H	0.408336	2.969857	2.774971
C	-1.24375	-0.44129	-2.81229
H	-1.02482	0.632578	-2.80826
H	-0.34091	-0.97507	-2.47744
H	-1.43562	-0.74045	-3.85694
C	-2.53416	-2.36405	-2.01363
H	-1.65591	-2.8302	-1.54791
H	-3.44747	-2.7654	-1.5563
H	-2.55293	-2.65721	-3.07742
C	-3.73009	-0.27532	-2.59057
H	-3.84594	-0.73621	-3.58674
H	-4.63162	-0.51784	-2.01363
H	-3.70008	0.810415	-2.74353
C	-1.97101	2.367596	-1.24791
H	-2.10655	3.448045	-1.07351
H	-0.89279	2.187314	-1.34447
H	-2.44743	2.13605	-2.20865
C	-4.09563	1.900348	-0.04626
H	-4.23508	2.994851	-0.07626
H	-4.6293	1.479552	-0.90681
H	-4.5815	1.543319	0.871112
C	-1.96652	2.220202	1.193703
H	-2.33184	1.772467	2.12384
H	-0.87454	2.136529	1.170574
H	-2.21685	3.29401	1.224437
C	-4.70322	-1.35297	0.512133
H	-5.28959	-1.85967	1.298101
H	-5.19633	-0.3963	0.303139
H	-4.76893	-1.97788	-0.38733
C	-2.67365	-2.58275	1.271713
H	-2.56951	-3.18443	0.362603
H	-1.68875	-2.51607	1.751219
H	-3.34734	-3.12654	1.955942
C	-3.31086	-0.50689	2.386151
H	-3.85516	0.445663	2.37764
H	-3.83936	-1.17538	3.086416
H	-2.30477	-0.33783	2.793962
H	1.047199	-5.29287	-0.38092
C	0.262329	-3.55154	0.581022
C	0.134559	-4.99981	0.163319

O	0.502827	-3.25155	1.75164
O	0.114631	-2.71605	-0.38403
H	0.023393	-5.64977	1.040842
H	-0.71385	-5.14203	-0.52189

TS2_OAc

E(pbe1pbe) = -1943.73284841 a.u.

Lowest Freq. = -346.61 cm^{-1}

C	-3.87958	-3.52988	-1.20604
C	-2.70241	-3.43673	-1.95401
C	-1.63255	-2.68225	-1.48402
C	-1.72996	-2.0035	-0.2466
C	-2.88444	-2.17081	0.537039
C	-3.95691	-2.91298	0.044059
H	-4.72195	-4.11533	-1.5832
H	-2.60619	-3.97514	-2.90121
H	-2.96866	-1.68172	1.507293
H	-4.86436	-3.00893	0.646606
C	-0.2562	-2.67305	-2.06357
H	-0.09486	-3.46035	-2.81164
H	-0.01077	-1.69864	-2.51882
C	0.644513	-2.85179	-0.84104
H	0.627928	-3.91191	-0.51123
O	1.944094	-2.46756	-1.1677
C	2.906076	-2.76396	-0.1821
H	2.816282	-3.82134	0.136933
H	2.7347	-2.14299	0.717274
C	4.246499	-2.51294	-0.69527
C	5.369554	-2.29285	-1.09996
C	6.712417	-2.02593	-1.58539
H	6.746956	-1.07719	-2.14398
H	7.422675	-1.9473	-0.7471
H	7.064514	-2.82766	-2.25342
Pd	-0.8655	-0.14589	0.134389
P	0.439314	2.044385	-0.04858
C	-0.17091	3.509512	1.020489
C	2.320917	1.808225	0.252979
C	0.249661	2.551206	-1.90264
C	0.025126	-2.02927	0.267494
C	0.274056	-1.42596	1.387532
C	0.386994	-1.59951	2.874802
C	-0.00039	-0.33236	3.635149
C	-0.59711	-2.71993	3.256271
C	1.800492	-2.04748	3.273388
H	0.733328	0.467692	3.485782
H	-0.98634	0.034539	3.311466
H	-0.049	-0.54972	4.713327
H	-0.36222	-3.65361	2.723612

H	-0.53392	-2.91147	4.339513
H	-1.6334	-2.43951	3.022205
H	1.834239	-2.20982	4.362613
H	2.069341	-2.99429	2.782003
H	2.559058	-1.29442	3.019031
C	0.251836	1.266811	-2.74466
H	1.170456	0.678276	-2.64414
H	-0.60604	0.631247	-2.48685
H	0.15567	1.547915	-3.80763
C	-1.10143	3.22875	-2.15792
H	-1.93099	2.605849	-1.80458
H	-1.17042	4.231264	-1.71755
H	-1.21059	3.350034	-3.24935
C	1.337161	3.496175	-2.42113
H	1.086257	3.761287	-3.46265
H	1.39161	4.433492	-1.85213
H	2.335943	3.04376	-2.44056
C	2.885308	0.882343	-0.83006
H	3.906011	0.591663	-0.53117
H	2.298072	-0.04229	-0.93058
H	2.963572	1.356378	-1.81571
C	3.156612	3.086797	0.325614
H	4.216553	2.798711	0.434855
H	3.078102	3.71509	-0.56884
H	2.899765	3.69785	1.201618
C	2.497694	1.046977	1.566091
H	2.189127	1.617793	2.448285
H	1.935945	0.104922	1.537783
H	3.56509	0.797501	1.688534
C	0.28111	4.897751	0.563351
H	-0.10393	5.641236	1.282683
H	1.372829	5.003398	0.538128
H	-0.11404	5.172552	-0.42257
C	-1.70222	3.450711	1.045351
H	-2.15709	3.657533	0.070032
H	-2.0715	2.469435	1.376621
H	-2.0692	4.216301	1.750453
C	0.290592	3.305657	2.465359
H	1.368839	3.464652	2.597643
H	-0.22705	4.044551	3.099809
H	0.028205	2.308029	2.842498
H	-5.03088	0.222374	-1.73365
C	-3.49688	0.843707	-0.37112
C	-4.75115	1.128536	-1.1722

O	-3.54522	0.765838	0.859959
O	-2.43831	0.683644	-1.07606
H	-5.58362	1.410764	-0.51427
H	-4.57242	1.9253	-1.91005

IM3_OAc

E(pbe1pbe) = -1943.81410002 a.u.

Lowest Freq. = 14.58 cm^{-1}

C	0.060483	4.516848	-2.23759
C	-1.21403	3.972759	-2.4271
C	-1.63027	2.923633	-1.61613
C	-0.78575	2.382073	-0.6269
C	0.464074	2.971917	-0.40865
C	0.883562	4.026268	-1.22192
H	0.400487	5.344632	-2.86567
H	-1.87985	4.374722	-3.19606
H	1.096896	2.62961	0.411583
H	1.863407	4.47993	-1.04939
C	-2.94672	2.218892	-1.59889
H	-3.8017	2.889935	-1.76612
H	-2.98047	1.430073	-2.37236
C	-2.96346	1.53621	-0.21698
H	-3.40557	2.239899	0.520519
O	-3.70674	0.349352	-0.22364
C	-5.09473	0.55327	-0.31339
H	-5.37676	0.970324	-1.2998
H	-5.42685	1.291672	0.444778
C	-5.79671	-0.70841	-0.10479
C	-6.38023	-1.75756	0.074939
C	-7.0726	-3.01609	0.292516
H	-7.88984	-2.89817	1.02154
H	-7.50457	-3.40328	-0.64373
H	-6.38024	-3.77755	0.685471
Pd	1.058852	0.23007	1.015943
P	1.589283	-1.19114	-0.76779
C	2.075556	-2.89982	-0.04529
C	0.356637	-1.44529	-2.21363
C	3.194256	-0.39866	-1.49836
C	-1.48039	1.311315	0.119638
C	-0.92139	0.343084	0.893455
C	-1.64735	-0.495	1.974208
C	-0.65525	-0.97258	3.050276
C	-2.67956	0.382921	2.700945
C	-2.34927	-1.751	1.439474
H	0.103497	-1.64962	2.629585
H	-0.13616	-0.13363	3.535636
H	-1.20626	-1.53195	3.824256
H	-3.53405	0.63245	2.061611
H	-3.07317	-0.16105	3.57523
H	-2.22494	1.318077	3.063303
H	-2.878	-2.24515	2.271635
H	-3.07701	-1.50573	0.659767
H	-1.6293	-2.47318	1.03646
C	2.977072	1.114645	-1.60495
H	2.108963	1.396075	-2.20991
H	2.860271	1.562323	-0.60865
H	3.870728	1.561427	-2.07271
C	4.382079	-0.58351	-0.5469
H	4.160091	-0.22682	0.466129
H	4.744989	-1.61739	-0.5007
H	5.211845	0.028234	-0.93966
C	3.601067	-0.943	-2.86958
H	4.561536	-0.47419	-3.14275
H	3.757395	-2.0291	-2.87152
H	2.886452	-0.6908	-3.66274
C	0.223146	-0.12224	-2.97538
H	-0.62356	-0.21497	-3.67536
H	0.00362	0.718286	-2.30285
H	1.109553	0.127493	-3.57164
C	0.74318	-2.562	-3.18733
H	0.021183	-2.54452	-4.02123
H	1.743895	-2.45179	-3.61833
H	0.670378	-3.55448	-2.7225
C	-1.03475	-1.7736	-1.66799
H	-1.07525	-2.72789	-1.13384
H	-1.41513	-0.98525	-1.01104
H	-1.72181	-1.85047	-2.52756
C	3.007392	-3.72599	-0.93554
H	3.166348	-4.70185	-0.44617
H	2.583063	-3.92425	-1.92733
H	3.995731	-3.26929	-1.06651
C	2.741978	-2.67681	1.318548
H	3.683907	-2.12246	1.26164
H	2.075366	-2.1369	2.006725
H	2.958666	-3.66362	1.760683
C	0.817784	-3.72761	0.220376
H	0.33308	-4.07893	-0.69939
H	1.111576	-4.62082	0.795969
H	0.084809	-3.17632	0.823607
H	4.112471	1.886029	4.091258
C	2.446401	1.523449	2.779651
C	3.175158	2.366965	3.782393

O	1.190218	1.659535	2.657048
O	3.074712	0.719992	2.044003
H	3.421756	3.332332	3.310799
H	2.542279	2.570296	4.656685

TS3_OAc

E(pbe1pbe) = -1943.75149310 a.u.

Lowest Freq. = -332.98 cm^{-1}

C	-0.60726	4.61078	-0.15155
C	-1.63051	4.031652	0.607983
C	-2.02816	2.729193	0.325692
C	-1.39684	1.991605	-0.69105
C	-0.39479	2.585605	-1.46949
C	-0.00268	3.895106	-1.19025
H	-0.29234	5.637183	0.055316
H	-2.11089	4.599305	1.410154
H	0.05299	2.031905	-2.2983
H	0.775158	4.369563	-1.79461
C	-3.07652	1.884046	0.986306
H	-2.71242	1.507448	1.957561
H	-4.00768	2.432237	1.193302
C	-3.27928	0.706218	0.016032
H	-3.46661	-0.22587	0.566069
O	-4.3332	0.934155	-0.90999
C	-5.62916	0.951082	-0.36349
H	-6.31137	1.13934	-1.20783
H	-5.76312	1.796482	0.33864
C	-6.01893	-0.29004	0.312175
C	-6.31742	-1.3246	0.874617
C	-6.65709	-2.57368	1.534
H	-6.82845	-2.42585	2.611925
H	-7.56742	-3.01861	1.102054
H	-5.83969	-3.30369	1.419332
Pd	0.534382	-0.23421	-0.33503
P	2.771002	-0.29002	0.094805
C	3.252124	-2.009	0.751458
C	3.52011	-0.00605	-1.64102
C	3.33768	1.077502	1.299251
C	-1.96751	0.634854	-0.7811
C	-1.32371	-0.4253	-1.37582
C	-1.95128	-1.75704	-1.75414
C	-0.89608	-2.86357	-1.65199
C	-3.18612	-2.20441	-0.96418
C	-2.35424	-1.59935	-3.23105
H	-0.00829	-2.62483	-2.25955
H	-0.57462	-2.9801	-0.60563
H	-1.29877	-3.82947	-2.00253
H	-4.0232	-1.50721	-1.09783
H	-3.52214	-3.19503	-1.31576
H	-2.95258	-2.28379	0.107748
H	-2.75349	-2.54997	-3.62502
H	-3.13213	-0.8279	-3.34686
H	-1.49287	-1.30785	-3.85367
C	2.482618	2.324105	1.035406
H	2.604171	2.731308	0.026145
H	1.417549	2.113837	1.204648
H	2.792652	3.107291	1.747198
C	3.074643	0.667937	2.752875
H	2.022574	0.402056	2.911173
H	3.717595	-0.15269	3.095014
H	3.303689	1.541845	3.385117
C	4.823484	1.416981	1.155037
H	5.074922	2.167909	1.922836
H	5.472904	0.547281	1.321757
H	5.072875	1.856231	0.180975
C	3.364645	1.469295	-2.02286
H	3.655846	1.576247	-3.08043
H	2.322149	1.806658	-1.93152
H	4.007015	2.140587	-1.44015
C	4.988013	-0.41172	-1.76642
H	5.328151	-0.15385	-2.78377
H	5.639799	0.115731	-1.05796
H	5.136742	-1.49225	-1.64033
C	2.683407	-0.79921	-2.65732
H	2.782971	-1.8847	-2.55729
H	1.609672	-0.5457	-2.59596
H	3.022427	-0.52305	-3.66954
C	4.694275	-2.07524	1.259185
H	4.904516	-3.11662	1.55565
H	5.427908	-1.79734	0.49169
H	4.86305	-1.44654	2.142679
C	2.283336	-2.39772	1.874782
H	2.310938	-1.71914	2.733793
H	1.247685	-2.45255	1.511206
H	2.570856	-3.39981	2.233986
C	3.065291	-3.05255	-0.35386
H	3.81041	-2.96932	-1.15511
H	3.187906	-4.04889	0.101561

H	2.057428	-3.0122	-0.79247
O	-1.12807	-1.74629	1.727759
H	-1.96184	-0.62648	3.905746
H	-0.23468	-0.80457	4.298213
C	-0.95027	-0.30235	3.626703
C	-0.64757	-0.73071	2.207344
H	-0.84383	0.781511	3.76416
O	0.190524	0.065542	1.617995

IM4_OAc

E(pbe1pbe) = -1943.81657049 a.u.

Lowest Freq. = 33.47 cm^{-1}

C	-6.13402	-1.06976	-1.03068
C	-5.14328	-0.65466	-1.92461
C	-3.8865	-0.31132	-1.44225
C	-3.57058	-0.38216	-0.06384
C	-4.58162	-0.79124	0.818452
C	-5.84786	-1.12847	0.332516
H	-7.12919	-1.33618	-1.39634
H	-5.35401	-0.59224	-2.99618
H	-4.41703	-0.84445	1.892347
H	-6.62277	-1.43745	1.039241
C	-2.70438	0.163211	-2.23327
H	-2.17655	-0.69086	-2.69114
H	-2.97292	0.851824	-3.04817
C	-1.83709	0.809387	-1.15359
H	-0.76028	0.823186	-1.38359
O	-2.28929	2.138647	-0.89699
C	-1.92515	3.094772	-1.85551
H	-2.63086	3.934749	-1.74471
H	-2.06064	2.713117	-2.8854
C	-0.55624	3.599532	-1.70895
C	0.57527	4.020732	-1.57329
C	1.942129	4.480107	-1.39629
H	1.975796	5.540138	-1.09899
H	2.432406	3.88516	-0.60927
H	2.524063	4.369129	-2.32495
Pd	0.616925	0.486792	0.783017
P	1.736553	-1.18763	-0.45196
C	3.418313	-1.34375	0.48452
C	1.005121	-2.94689	-0.65212
C	2.116014	-0.50052	-2.20565
C	-2.14146	0.012738	0.106169
C	-1.19582	-0.26999	1.038141
C	-1.46409	-0.8327	2.442674
C	-2.34348	0.192071	3.184618
C	-2.10619	-2.22806	2.38183
C	-0.19704	-0.97191	3.295073
H	-1.77794	1.126693	3.31266
H	-3.26734	0.444613	2.651164
H	-2.61526	-0.19064	4.182561
H	-1.36499	-2.97725	2.071388
H	-2.47016	-2.51731	3.381456
H	-2.94949	-2.29716	1.685621
H	-0.47376	-1.34467	4.295252
H	0.515975	-1.68267	2.862175
H	0.312709	-0.00557	3.423047
C	1.718903	-3.81059	-1.69629
H	2.792594	-3.92388	-1.50764
H	1.270839	-4.81794	-1.66089
H	1.582657	-3.43684	-2.71887
C	-0.4721	-2.83657	-1.0432
H	-1.05188	-2.29826	-0.28556
H	-0.63034	-2.33876	-2.00634
H	-0.88023	-3.85745	-1.1275
C	1.052287	-3.67685	0.692234
H	2.065701	-3.97834	0.98425
H	0.618696	-3.07771	1.502105
H	0.450772	-4.59649	0.604823
C	4.274572	-2.53903	0.059826
H	5.231095	-2.47801	0.606089
H	3.821891	-3.50487	0.316551
H	4.510883	-2.53927	-1.01119
C	4.252507	-0.06955	0.305453
H	4.662553	0.040829	-0.70574
H	3.691807	0.835997	0.569535
H	5.113098	-0.14103	0.991749
C	3.131471	-1.44891	1.987164
H	2.614706	-0.55145	2.357719
H	2.536868	-2.32779	2.259545
H	4.095988	-1.52519	2.516512
C	0.928581	-0.73667	-3.14293
H	0.811332	-1.78785	-3.43411
H	-0.01834	-0.38333	-2.71986
H	1.108507	-0.16213	-4.06652
C	2.282729	1.017706	-2.08463
H	1.37767	1.486549	-1.67544
H	3.127776	1.315034	-1.45567
H	2.450736	1.430865	-3.09346
C	3.359482	-1.09371	-2.87331
H	3.434267	-0.66366	-3.88648
H	4.290574	-0.84301	-2.3515
H	3.307828	-2.18349	-2.98594
O	0.1354	2.205134	1.985009
C	1.251902	2.774975	1.790446
C	1.462071	4.155482	2.335959

H	1.008878	4.248581	3.332762
H	2.52961	4.406673	2.376796
H	0.955374	4.874788	1.672326
O	2.152225	2.20156	1.124043

TS4_OAc

E(pbe1pbe) = -1943.78751679 a.u.

Lowest Freq. = -58.10 cm^{-1}

C	6.190075	-1.54055	-0.2644
C	5.247682	-1.67704	-1.28562
C	4.003623	-1.07026	-1.15473
C	3.653544	-0.32526	0.000886
C	4.625639	-0.17771	1.001485
C	5.87651	-0.78395	0.864545
H	7.172992	-2.00995	-0.35643
H	5.485474	-2.24713	-2.18824
H	4.439696	0.414639	1.894563
H	6.619219	-0.6534	1.65618
C	2.891165	-1.05086	-2.15876
H	3.240241	-0.9783	-3.19909
H	2.252497	-1.94629	-2.07
C	2.134563	0.185056	-1.68948
H	2.76112	1.053395	-1.97754
O	0.873386	0.3458	-2.27312
C	0.663134	1.626581	-2.82216
H	-0.32714	1.608726	-3.30109
H	1.404235	1.830248	-3.61867
C	0.720148	2.700047	-1.8281
C	0.846053	3.616189	-1.03723
C	0.983243	4.725533	-0.11198
H	0.914877	5.684844	-0.64957
H	0.179476	4.683448	0.637818
H	1.955955	4.695607	0.402812
Pd	-0.57343	0.839713	0.061705
P	-1.68629	-1.29055	0.026208
C	-2.08114	-1.76435	-1.7956
C	-0.85507	-2.82304	0.843002
C	-3.37833	-1.00503	0.923598
C	2.240119	0.140676	-0.15994
C	1.238006	0.361315	0.731523
C	1.471581	0.63337	2.232682
C	0.178783	0.971251	2.985691
C	2.118265	-0.54885	2.971733
C	2.326308	1.913712	2.325218
H	-0.36574	1.799274	2.5082
H	-0.49848	0.112842	3.049392
H	0.429407	1.270153	4.017537
H	1.391454	-1.3587	3.113477
H	2.983308	-0.97858	2.455771
H	2.450311	-0.22688	3.972674
H	2.601334	2.116518	3.373788
H	3.245227	1.877091	1.728463
H	1.74242	2.772354	1.960726
C	-3.11227	-0.1884	2.191923
H	-2.48334	-0.71308	2.920352
H	-2.64054	0.776239	1.9538
H	-4.0793	0.016753	2.68114
C	-4.32932	-0.16643	0.06184
H	-3.85414	0.756788	-0.29066
H	-4.73075	-0.71867	-0.79676
H	-5.18989	0.107217	0.695371
C	-4.12222	-2.28971	1.301426
H	-5.08867	-1.99443	1.743948
H	-4.34403	-2.92421	0.43457
H	-3.60201	-2.89536	2.052365
C	-0.89191	-2.67514	2.36552
H	-0.23428	-3.44494	2.801418
H	-0.5156	-1.70023	2.692519
H	-1.89001	-2.82241	2.794256
C	-1.49186	-4.1682	0.478238
H	-0.99138	-4.94481	1.081071
H	-2.56328	-4.22317	0.701298
H	-1.3392	-4.43613	-0.57461
C	0.621291	-2.87684	0.439477
H	0.770112	-3.01632	-0.63686
H	1.156273	-1.96981	0.737173
H	1.08485	-3.73615	0.951983
C	-3.2443	-2.74677	-1.9635
H	-3.33196	-2.97494	-3.03948
H	-3.08977	-3.69835	-1.44264
H	-4.20845	-2.33099	-1.64762
C	-2.40443	-0.48557	-2.572
H	-3.3045	0.024461	-2.21631
H	-1.57398	0.225426	-2.51499
H	-2.55917	-0.75609	-3.6301
C	-0.83951	-2.37269	-2.45275
H	-0.60186	-3.37701	-2.08116
H	-1.04485	-2.46913	-3.53223
H	0.032438	-1.71782	-2.34331
O	-2.26696	1.950296	-0.67634
C	-3.18236	4.114835	-0.3542
H	-4.14876	3.720955	-0.69963

H	-3.33385	4.820469	0.473267
H	-2.7269	4.660839	-1.19685
C	-2.25041	2.999597	0.049043
O	-1.48878	3.136046	1.022194

IM5_OAc

E(pbe1pbe) = -1943.79797915 a.u.

Lowest Freq. = 22.58 cm^{-1}

C	-6.43152	1.040506	0.127898
C	-5.57075	1.416715	-0.90497
C	-4.29108	0.875748	-0.95883
C	-3.82176	-0.03445	0.019386
C	-4.71466	-0.42524	1.028163
C	-6.00253	0.11321	1.077364
H	-7.4417	1.454812	0.181125
H	-5.90024	2.120558	-1.6745
H	-4.43732	-1.15649	1.783913
H	-6.68175	-0.20679	1.872074
C	-3.25598	1.089996	-2.01803
H	-3.67472	1.18146	-3.03057
H	-2.64635	1.987559	-1.81478
C	-2.40967	-0.16165	-1.83507
H	-2.99716	-1.00931	-2.25001
O	-1.21145	-0.08452	-2.54284
C	-0.70279	-1.33199	-2.91531
H	0.169409	-1.14983	-3.5611
H	-1.4461	-1.90206	-3.50723
C	-0.30097	-2.13916	-1.75347
C	-0.07492	-3.04299	-0.93571
C	0.108083	-4.32772	-0.27224
H	0.392876	-5.08037	-1.02525
H	0.903701	-4.27073	0.482774
H	-0.82403	-4.66452	0.205855
Pd	0.53732	-0.97827	-0.17082
P	1.544428	1.377626	0.148539
C	1.85482	2.073675	-1.61759
C	0.548582	2.716528	1.113327
C	3.264589	1.23421	1.01531
C	-2.40435	-0.39375	-0.3099
C	-1.34802	-0.70798	0.478754
C	-1.4588	-1.15372	1.954664
C	-0.08749	-1.44149	2.582111
C	-2.12625	-0.10419	2.858096
C	-2.21703	-2.49406	1.990034
H	0.509266	-2.16683	2.004784
H	0.512773	-0.53039	2.687222
H	-0.22886	-1.85773	3.593906
H	-1.46764	0.760943	2.997654
H	-3.08126	0.273395	2.48007
H	-2.30926	-0.54127	3.853517
H	-2.4543	-2.76958	3.031011
H	-3.14928	-2.48873	1.412822
H	-1.58712	-3.29245	1.575106
C	3.106766	0.265518	2.192223
H	2.416817	0.633198	2.96103
H	2.767778	-0.72647	1.860215
H	4.092178	0.139479	2.672782
C	4.30716	0.622606	0.071099
H	3.928624	-0.27367	-0.42996
H	4.661612	1.332854	-0.68619
H	5.183067	0.339527	0.678997
C	3.849533	2.554428	1.525732
H	4.851897	2.33785	1.933306
H	3.978196	3.298151	0.729313
H	3.27026	3.00995	2.337214
C	0.618186	2.423012	2.615166
H	-0.13155	3.051084	3.123914
H	0.382027	1.378513	2.847803
H	1.592202	2.660348	3.058568
C	1.005677	4.161407	0.890933
H	0.412064	4.806435	1.561229
H	2.062186	4.329718	1.128153
H	0.818524	4.510068	-0.13249
C	-0.92622	2.623728	0.711151
H	-1.10141	2.887301	-0.33743
H	-1.33176	1.620516	0.874043
H	-1.50047	3.332374	1.331206
C	2.887382	3.201133	-1.69967
H	2.927374	3.54402	-2.74787
H	2.626493	4.069999	-1.08449
H	3.900667	2.879351	-1.43144
C	2.309772	0.91979	-2.51588
H	3.271677	0.490251	-2.21979
H	1.572914	0.107491	-2.51836
H	2.407311	1.300885	-3.54646
C	0.538106	2.587992	-2.20538
H	0.195607	3.52142	-1.74196
H	0.708471	2.803761	-3.27395
H	-0.25181	1.829536	-2.14893
O	2.48137	-1.6928	-0.77838
C	4.280035	-3.20431	-0.62397
H	5.089503	-2.45607	-0.60512

H	4.596512	-4.07892	-0.04049
H	4.125811	-3.48961	-1.67514
C	3.022268	-2.58816	-0.04263
O	2.617078	-2.94282	1.070824

TS5_OAc

E(pbe1pbe) = -1943.76865844 a.u.

Lowest Freq. = -261.09 cm^{-1}

C	5.944943	1.248896	-0.74405
C	5.341504	1.344593	0.510238
C	4.208791	0.587698	0.786448
C	3.642111	-0.2898	-0.17583
C	4.269188	-0.37122	-1.43057
C	5.405664	0.390727	-1.70262
H	6.83586	1.839234	-0.97304
H	5.749721	2.012073	1.274153
H	3.899381	-1.02497	-2.2156
H	5.87747	0.305661	-2.68498
C	3.425045	0.593382	2.064138
H	4.058726	0.576006	2.962316
H	2.772217	1.478615	2.128764
C	2.621239	-0.6766	1.890665
H	3.330654	-1.51128	2.092961
O	1.545785	-0.82944	2.748238
C	1.044333	-2.13208	2.657017
H	0.350431	-2.27579	3.49786
H	1.862864	-2.87113	2.754562
C	0.286177	-2.35691	1.396018
C	-0.76966	-2.91266	0.950995
C	-1.7754	-3.96814	0.87124
H	-1.34807	-4.917	1.231539
H	-2.64751	-3.71674	1.49476
H	-2.13793	-4.107	-0.15711
Pd	-0.79368	-0.95248	0.209556
P	-1.17309	1.586018	-0.08506
C	-1.35466	2.186851	1.728954
C	0.148864	2.693572	-0.9469
C	-2.83201	1.961105	-0.99465
C	2.37778	-0.84045	0.390384
C	1.251114	-1.36359	-0.17651
C	1.275796	-2.07335	-1.555
C	0.044329	-2.93212	-1.88402
C	1.330463	-1.01726	-2.67136
C	2.449726	-3.0692	-1.57827
H	-0.02409	-3.8155	-1.23455
H	-0.91177	-2.38953	-1.82694
H	0.158852	-3.30117	-2.91733
H	0.337935	-0.55464	-2.77222
H	2.052708	-0.21113	-2.49941
H	1.577529	-1.49533	-3.63314
H	2.547526	-3.5182	-2.58016
H	3.419093	-2.64303	-1.30142
H	2.244038	-3.88441	-0.86655
C	-2.86108	1.07408	-2.24596
H	-2.08624	1.340687	-2.97454
H	-2.7489	0.008538	-2.00027
H	-3.83645	1.201879	-2.74676
C	-4.0455	1.589196	-0.13393
H	-3.9615	0.580777	0.282199
H	-4.21453	2.303314	0.682289
H	-4.93824	1.627384	-0.78124
C	-3.03192	3.423169	-1.40617
H	-4.02878	3.505831	-1.87252
H	-3.01943	4.107322	-0.54758
H	-2.3065	3.781243	-2.1452
C	0.06621	2.527989	-2.46902
H	0.968619	2.986179	-2.90742
H	0.061256	1.472187	-2.76926
H	-0.79872	3.023715	-2.92436
C	0.090253	4.183884	-0.60343
H	0.84124	4.703919	-1.22285
H	-0.88087	4.648364	-0.80845
H	0.354422	4.378565	0.444607
C	1.529762	2.172106	-0.55886
H	1.721884	2.215458	0.516586
H	1.660738	1.137358	-0.88355
H	2.296398	2.786632	-1.06001
C	-2.04062	3.541993	1.910737
H	-2.0266	3.793668	2.985285
H	-1.53184	4.354726	1.37928
H	-3.09231	3.53221	1.598554
C	-2.13687	1.121383	2.507902
H	-3.14855	0.951894	2.125915
H	-1.60905	0.155467	2.495315
H	-2.21794	1.451736	3.5575
C	0.03065	2.243794	2.375073
H	0.650733	3.066582	1.996777
H	-0.10091	2.408723	3.458029
H	0.567332	1.292914	2.254887
O	-2.92617	-1.17198	0.395061
C	-5.02473	-2.12084	-0.09487
H	-5.42981	-2.95353	-0.68555

H	-5.12596	-2.33165	0.979146
H	-5.6251	-1.22189	-0.3131
C	-3.58298	-1.84161	-0.47585
O	-3.15282	-2.22019	-1.56874

PC

E(pbe1pbe) = -772.512952086 a.u.

Lowest Freq. = 20.48 cm^{-1}

C	4.3702	0.6198	-0.4011
C	3.9103	-0.6494	-0.0431
C	2.5417	-0.8713	0.0621
C	1.6124	0.1684	-0.1630
C	2.0869	1.4270	-0.5586
C	3.4599	1.6451	-0.6688
H	5.4435	0.8059	-0.4928
H	4.6165	-1.4638	0.1398
H	1.3992	2.2351	-0.8066
H	3.8234	2.6275	-0.9809
C	1.8352	-2.1519	0.3863
H	2.3012	-3.0375	-0.0683
H	1.7986	-2.3128	1.4779
C	0.4108	-1.8930	-0.0991
H	0.2941	-2.2534	-1.1443
O	-0.5179	-2.5293	0.7329
C	-1.8276	-2.3132	0.3073
H	-2.4832	-2.5480	1.1610
H	-2.0943	-2.9988	-0.5208
C	-2.0705	-0.8711	-0.1371
C	-3.1745	-0.6114	-0.8076
C	-4.2757	-0.4112	-1.4854
H	-5.2164	-0.1385	-0.9884
H	-4.2989	-0.4989	-2.5797
C	0.2499	-0.3765	-0.0487
C	-1.0018	0.1347	0.1105
C	-1.3875	1.5754	0.4774
C	-0.3846	2.1703	1.4744
C	-1.4795	2.4396	-0.7881
C	-2.7460	1.6177	1.1953
H	-0.3589	1.5716	2.3986
H	0.6383	2.2337	1.0902
H	-0.6972	3.1915	1.7437
H	-2.2404	2.0447	-1.4787
H	-1.7641	3.4720	-0.5261
H	-0.5248	2.4786	-1.3320
H	-2.9189	2.6376	1.5727
H	-3.5861	1.3620	0.5378
H	-2.7656	0.9327	2.0577

L

E(pbe1pbe) = -814.385499605 a.u.

Lowest Freq. = 96.92 cm^{-1}

P	-0.0002	-0.0003	-0.6985
C	-1.5128	-0.9602	-0.0042
C	-0.0756	1.7897	-0.0040
C	1.5885	-0.8294	-0.0044
C	0.8425	2.6475	-0.8889
H	0.7159	3.7092	-0.6145
H	1.9060	2.4072	-0.7777
H	0.5807	2.5441	-1.9538
C	-1.4871	2.3472	-0.2167
H	-2.2329	1.9013	0.4542
H	-1.4696	3.4306	-0.0076
H	-1.8292	2.2198	-1.2556
C	0.3087	1.9798	1.4630
H	0.1854	3.0431	1.7356
H	-0.3233	1.3920	2.1421
H	1.3570	1.7178	1.6603
C	-2.7150	-0.5906	-0.8872
H	-3.5709	-1.2324	-0.6152
H	-3.0384	0.4501	-0.7711
H	-2.4945	-0.7608	-1.9530
C	-1.8693	-0.7268	1.4638
H	-2.1671	0.3112	1.6654
H	-2.7283	-1.3663	1.7346
H	-1.0443	-0.9824	2.1421
C	-1.2911	-2.4609	-0.2209
H	-2.2383	-2.9874	-0.0131
H	-1.0098	-2.6911	-1.2605
H	-0.5322	-2.8858	0.4489
C	1.5651	-1.2534	1.4639
H	0.8167	-2.0319	1.6651
H	2.5492	-1.6752	1.7354
H	1.3714	-0.4110	2.1415
C	1.8699	-2.0562	-0.8860
H	1.9027	-1.7828	-1.9526
H	2.8558	-2.4731	-0.6164
H	1.1331	-2.8585	-0.7653
C	2.7764	0.1138	-0.2222
H	2.8352	0.4712	-1.2623
H	2.7634	0.9844	0.4464
H	3.7065	-0.4420	-0.0132

KOAc

E(pbe1pbe) = -828.170296267 a.u.

Lowest Freq. = 20.35 cm^{-1}

C	-0.9860	0.0027	-0.0229
C	-2.5103	-0.0006	0.0205
H	-2.8355	-0.1017	1.0698
H	-2.9241	0.9385	-0.3732
H	-2.9171	-0.8592	-0.5334
O	-0.4077	1.1192	-0.0227
O	-0.4093	-1.1151	-0.0230
K	1.9048	-0.0012	0.0114

KI

E(pbe1pbe) = -897.590295890 a.u.

Lowest Freq. = 119.81 cm^{-1}

I	0.0000	0.0000	0.8699
K	0.0000	0.0000	-2.4264

HOAc

E(pbe1pbe) = -228.944299135 a.u.

Lowest Freq. = 79.58 cm^{-1}

C	0.0909	0.1183	0.0000
C	-1.3864	-0.1099	0.0000
H	-1.6730	-0.6969	0.8858
H	-1.9149	0.8500	0.0002
H	-1.6729	-0.6965	-0.8862
O	0.6385	1.1931	0.0000
O	0.7756	-1.0314	0.0000
H	1.7207	-0.8005	0.0000

7. References

(1) Ly, K. U.; Boussonnière, A.; Castanet, A.-S. Intramolecular *Anti*-Carbolithiation of Alkynes: Stereo-Directing Effect of Lithium-Coordinating Substituents. *Eur. J. Org. Chem.* **2022***, 2022*

(2) Reding, A.; Jones, P. G.; Werz, D. B. *trans*-Carbocarbonation of Internal Alkynes through a Formal *anti*-Carbopalladation/C-H Activation Cascade. *Angew. Chem. Int. Ed.* **2018***, 57*, 10610–10614.

(3) Tummatorn, J.; Dudley, G. B. Generation of medium-ring cycloalkynes by ring expansion of vinylogous acyl triflates. *Org. Lett.* **2011***, 13*, 1572–1575.

(4) Milde, B.; Reding, A.; Geffers, F. J.; Jones, P. G.; Werz, D. B. Intramolecular trans-Dicarbofunctionalization of Alkynes by a Formal *anti*-Carbopalladation/Stille Cascade. *Chem. Eur. J.* **2016***, 22*, 14544–14547.

(5) Dimirjian, C. A.; Castiñeira Reis, M.; Balmond, E. I.; Turman, N. C.; Rodriguez, E. P.; Di Maso, M. J.; Fettinger, J. C.; Tantillo, D. J.; Shaw, J. T. Synthesis of Spirobicyclic Pyrazoles by Intramolecular Dipolar Cycloadditions/[1s, 5s] Sigmatropic Rearrangements. *Org. Lett.* **2019***, 21*, 7209–7212.

(6) Woodin, K. S.; Jamison, T. F. Total synthesis of pumiliotoxins 209F and 251D via late-stage, nickel-catalyzed epoxide-alkyne reductive cyclization. *J. Org. Chem.* **2007***, 72*, 7451–7454.

(7) Kalvani, P.; Werz, D. B. *anti*-Carbopalladation Cascades of Internal Alkynes Terminated by Intermolecular Suzuki and Sonogashira Reactions. *Org. Lett.* **2024***, 26*, 10404–10408.

(8) Hötling, S.; Haberlag, B.; Tamm, M.; Collatz, J.; Mack, P.; Steidle, J. L. M.; Vences, M.; Schulz, S. Identification and synthesis of macrolide pheromones of the grain beetle Oryzaephilus surinamensis and the frog Spinomantis aglavei. *Chem. Eur. J.* **2014***, 20*, 3183–3191.

(9) Schulz, S.; Yildizhan, S.; Stritzke, K.; Estrada, C.; Gilbert, L. E. Macrolides from the scent glands of the tropical butterflies Heliconius cydno and Heliconius pachinus. *Org. Biomol. Chem.* **2007***, 5*, 3434–3441.

(10) Pawliczek, M.; Milde, B.; Jones, P. G.; Werz, D. B. Intramolecular Formal *anti*-Carbopalladation/Heck Reaction: Facile Domino Access to Carbo- and Heterooligocyclic Dienes. *Chem. Eur. J.* **2015***, 21*, 12303–12307.

(11) Frisch, M. J.; Trucks, G. W.; Schlegel, H. B.; Scuseria, G. E.; Robb, M. A.; Cheeseman, J. R.; Scalmani, G.; Barone, V.; Petersson, G. A.; Nakatsuji, H. *Gaussian 16, Revision C.01;* Gaussian, Inc., Wallingford CT**,** 2016**.**

(12) Adamo, C.; Barone, V. Toward reliable density functional methods without adjustable parameters: The PBE0 model. *J. Chem. Phys.* **1999***, 110*, 6158–6170.

(13) Weigend, F.; Ahlrichs, R. Balanced basis sets of split valence, triple zeta valence and quadruple zeta valence quality for H to Rn: Design and assessment of accuracy. *Phys. Chem. Chem. Phys* **2005**, *7*, 3297–3305.

(14) (a) Grimme, S.; Antony, J.; Ehrlich, S.; Krieg, H. A consistent and accurate ab initio parametrization of density functional dispersion correction (DFT-D) for the 94 elements H-Pu. *J. Chem. Phys.* **2010**, *132*, 154104. (b) Grimme, S.; Ehrlich, S.; Goerigk, L. Effect of the damping function in dispersion corrected density functional theory. *J. Comput. Chem.* **2011**, *32*, 1456–1465.

(15) Marenich, A. V.; Cramer, C. J.; Truhlar, D. G. Universal solvation model based on solute electron density and on a continuum model of the solvent defined by the bulk dielectric constant and atomic surface tensions. *J. Phys. Chem. B* **2009**, *113*, 6378–6396.
